# Enantioselective Alkoxycarbonyl‐Lactonization of Alkenes via the Merger of Photoredox and Copper Catalysis

**DOI:** 10.1002/advs.202510918

**Published:** 2025-08-13

**Authors:** Yuping Xiong, Zhipeng Zong, Wenlin Xie, Jian‐Qiang Chen, Xiaoyu Zhou, Jie Wu

**Affiliations:** ^1^ School of Pharmaceutical and Chemical Engineering & Institute for Advanced Studies Taizhou University Taizhou 318000 China; ^2^ School of Chemistry and Chemical Engineering Hunan University of Science and Technology Xiangtan 411201 China; ^3^ State Key Laboratory of Organometallic Chemistry, Shanghai Institute of Organic Chemistry Chinese Academy of Sciences Shanghai 200032 China

**Keywords:** chiral γ‐butyrolactones, enantioselective alkoxycarbonylation, lactonization, photocatalysis, radical

## Abstract

The synthesis of ester derivatives represents one of the most significant and fundamental tasks in modern organic chemistry, and numerous methods have been developed in both academic and industrial contexts. Among these, alkoxycarbonyl radical‐mediated functionalization strategies have emerged as a highly efficient and versatile tool for the synthesis of these compounds. Despite the significant advancements in recent years, the radical‐induced enantioselective alkoxycarbonylation reaction remains largely unexplored. Herein, a highly enantioselective alkoxycarbonyl‐lactonization of alkenes is reported through a photoredox/copper dual‐catalyzed alkoxycarbonyl radical addition/cyclization reaction. With this approach, a diverse array of ester‐substituted chiral γ‐butyrolactones bearing a quaternary stereocenter can be readily obtained in high yields and with excellent enantioselectivities under mild reaction conditions. This versatile method shows good substrate tolerance for primary, secondary, and tertiary alcohol derivatives while maintaining exceptional stereochemical control.

## Graphical abstract

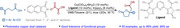

## 1 Introduction

**Figure 1:**
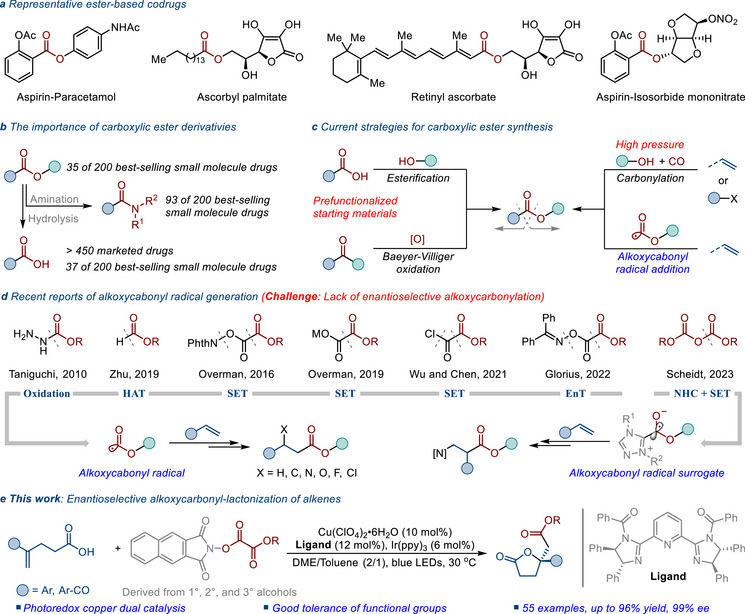
Ester‐containing bioactive compounds and synthesis of carboxylic esters. a) Representative ester‐based codrugs. b) The importance of carboxylic ester derivativies. c) Current strategies for carboxylic ester synthesis. d) Recent reports of alkoxycabonyl radical generation. e) This work: Enantioselective alkoxycarbonyl‐lactonization of alkenes.

Carboxylic esters are perhaps the most pivotal class of chemicals and have played a central role in the synthesis of diverse natural products, agrochemicals, bioactive compounds, and polymers. Their versatility in chemical transformations makes them indispensable intermediates in pharmaceuticals, materials science, and other industries. In the field of pharmaceuticals, esters exhibit exceptional versatility as carrier moieties, functioning as efficient prodrugs for both carboxyl and hydroxyl functional groups.^[^ ^1^, ^2^ ^]^ Moreover, the ester group can also serve as a biodegradable linker, thereby facilitating the covalent conjugation of two or more pharmacologically active agents to generate a novel codrug (**Figure** **1**a).^[^ ^3^, ^4^, ^5^ ^]^ The esterified codrug can be readily hydrolyzed by esterases to provide two or more drugs. This strategy leverages enzymatic cleavage mechanisms to enable site‐specific release of targeted drugs. Carboxylic esters represent one of the most versatile starting materials for the step‐ and atom‐economical synthesis of carboxylic acids and amides. More than 450 drugs containing a carboxylic acid group have been commercialized globally, including widely used anticoagulants, antibiotics, and cholesterol‐lowering agents.^[^ ^6^ ^]^ Notably, ≈72% (144 out of 200) of the top‐selling small‐molecule drugs in 2023 contain a carboxylic acid, ester, or amide functional group (Figure 1b). Therefore, the synthesis of ester derivatives represents one of the most significant and fundamental tasks in modern organic chemistry, and numerous methods have been developed in both academic and industrial contexts (Figure 1c).

The general methods for accessing carboxylic ester derivatives include the esterification of carboxylic acids with alcohols or the Baeyer−Villiger oxidation of the corresponding ketones (Figure 1c, left). These methods require the pre‐introduction of a carbonyl group in the starting material. Compared with these classical methods, the transition‐metal‐catalyzed carbonylative esterification of alkenes or organic halides with CO and alcohols is another widely adopted and significant approach for introducing ester groups into substrates.^[^ ^7^, ^8^, ^9^, ^10^, ^11^ ^]^ The direct alkoxycarbonylation of readily available alkenes to produce carboxylic esters is a pivotal transformation for the chemical industry, with an annual production exceeding multimillion tons.^[^ ^10^, ^11^ ^]^ However, this process often uses high‐pressure, toxic, and flammable CO gas at high temperatures, which means that special equipment and safety measures are required (Figure 1c, right).

Recently, alkoxycarbonyl radical‐mediated functionalization strategies have emerged as a powerful tool for the synthesis of carboxylic ester derivatives in synthetic organic chemistry, owing to their mild operational conditions and exceptional functional group tolerance (Figure 1d).^[^ ^12^, ^13^, ^14^, ^15^, ^16^, ^17^, ^18^, ^19^, ^20^, ^21^, ^22^, ^23^, ^24^, ^25^ ^]^ In 2010, Taniguchi and co‐workers reported an iron‐catalyzed 1,2‐hydroxy alkoxycarbonylation of electron‐rich alkenes with carbazates to provide β‐hydroxy esters.^[^ ^12^ ^]^ In this seminal study, carbazates were utilized for the first time as precursors of alkoxycarbonyl radicals. Subsequently, Zhu and co‐workers reported a copper‐catalyzed 1,2‐hydroxy methoxycarbonylation of styrenes using methyl formate as an alkoxycarbonyl radical precursor.^[^ ^13^ ^]^ Futhermore, the group of Overman recently achieved the formation of alkoxycarbonyl radicals from alkyl *N*‐phthalimidoyl oxalates and alcohol‐derived oxalates through a photoredox‐catalyzed single‐electron transfer (SET) process.^[^ ^14^, ^15^, ^16^, ^17^ ^]^ Inspired by these discoveries, our group also reported a photoredox‐catalyzed alkoxycarbonylchlorination of alkenes using various alcohol‐derived alkyloxalyl chlorides as precursors of alkoxycarbonyl radicals.^[^ ^18^ ^]^ In 2022, Glorius and co‐workers developed a photoinduced intermolecular aminocarboxylation of alkenes, enabling the synthesis of β‐amino acid derivatives through radical‐mediated C‐C and C‐N bond formation under mild conditions.^[^ ^19^ ^]^ This reaction features a radical decarboxylation triggered by an energy transfer (EnT) process to release both alkoxycarbonyl and iminyl radicals. Very recently, Scheidt and co‐workers developed a new *N*‐heterocyclic carbene (NHC)/photoredox cooperative catalyzed aminocarboxylation of alkenes with dialkyl pyrocarbonate, enabling the efficient synthesis of β‐amino esters.^[^ ^20^ ^]^ In this reaction, nucleophilic addition of the NHC catalyst to dialkyl pyrocarbonate provides an ester‐substituted azolium, which can be readily reduced by a photocatalyst to generate the corresponding NHC‐stabilized alkoxycarbonyl radical.^[^ ^21^ ^]^ These methods have demonstrated significant potential in the synthesis of diverse high‐value carboxylic ester derivatives, particularly in pharmaceutical and natural product development. Despite the significant advancements in recent years, the radical‐induced enantioselective alkoxycarbonylation reaction remains largely unexplored.

Over the past decade, Buchwald,^[^ ^26^ ^]^ Bao,^[^ ^27^ ^]^ Liu,^[^ ^28^ ^]^ Chen,^[^ ^29^ ^]^ Yu,^[^ ^30^ ^]^ and Gong,^[^ ^31^ ^]^ along with their co‐workers, have reported a series of copper‐catalyzed radical‐mediated asymmetric C‐O bond cross‐coupling reactions for the synthesis of chiral carboxylic esters.^[^ ^32^ ^]^ Furthermore, chiral γ‐butyrolactone structures are widely present in many natural products and biologically active molecules.^[^ ^33^ ^]^ The integration of pharmacologically active ester motifs into chiral γ‐butyrolactone scaffolds would offer a pioneering approach for modern drug discovery. Based on our interest in photoredox‐catalyzed alkoxycarbonylation of alkenes, we herein report a photoredox/copper dual‐catalyzed enantioselective alkoxycarbonyl‐lactonization of alkenes (Figure 1e).^[^ ^18^, ^22^, ^23^ ^]^ By using this approach, a diverse range of ester‐substituted chiral γ‐butyrolactones bearing a quaternary stereocenter can be readily obtained in high yields and with excellent enantioselectivities under mild reaction conditions. This versatile method shows good substrate tolerance for primary, secondary, and tertiary alcohol derivatives while maintaining exceptional stereochemical control.

## 2 Results and Discussion

### 2.1 Reaction Conditions Optimization

Initially, we explored the proposed enantioselective alkoxycarbonyl‐lactonization reaction by employing *N*‐hydroxy 2,3‐naphthalimide‐derived redox‐active ester (RAE) **2a** as a precursor of the alkoxycarbonyl radical and 4‐benzoylpent‐4‐enoic acid (**1a**) as a radical trap (**Table** **1**). After a variety of examinations of the reaction parameters, we were pleased to find that a combination of Cu(ClO_4_)_2_•6H_2_O, the chiral pyridyl‐bis(imidazole) ligand **L5**,^[^ ^34^ ^]^ and Ir(ppy)_3_ as a photoredox catalyst under irradiation with blue light‐emitting diodes (LEDs) at 30 °C yielded the desired alkoxycarbonylative lactonization product **3a** in 93% yield and 94% enantiomeric excess (ee) (entry 1).^[^ ^35^ ^]^ The investigation of several ligand scaffolds revealed that previously widely employed bis(oxazoline), bis(imidazole), and pyridyl‐bis(oxazoline) ligands (**L1**−**L3**) exhibited no significant effectiveness in this transformation. In contrast, the chiral pyridyl‐bis(imidazole) (PyBim) ligand **L4** was found to be highly effective (84% yield and 88% ee). Further exploration of acyl‐substituted chiral PyBim ligands (**L5**−**L12**) revealed that benzoyl‐substituted PyBim ligand **L5** exhibited the best stereoselectivity. Increasing the steric hindrance from Ph (**L5**) to *^t^* Bu (**L11**) led to a significant decrease in yield and a slight reduction in enantioselectivity (71% yield and 91% ee). As shown in entries 2−4, other cationic copper precatalysts, such as CuOTf, [Cu(MeCN)_4_]PF_6_, and Cu(OTf)_2_, could also be used as the precatalysts for this enantioselective alkoxycarbonyl‐lactonization reaction. The examination of the solvents revealed that 1,2‐dimethoxyethane (DME) produced the highest yield but slightly lower enantioselectivity (entries 5–9). The use of MeCN and DMF led to a noticeable decrease in both yield and enantioselectivity (entries 8 and 9). Meanwhile, when alkyl *N*‐phthalimidoyl oxalate **2a’** was employed as an alkoxycarbonyl radical precursor, the yield of the product **3a** decreased to 33% (entry 10). Lastly, control experiments have shown that all parameters, including the copper catalyst, visible light, and photoredox catalyst, are necessary for this photoredox/copper dual‐catalyzed enantioselective alkoxycarbonyl‐lactonization reaction (entries 11 and 12). Reducing the amount of photocatalyst and increasing the reaction temperature could lead to a moderate decline in both yield and enantioselectivity (Table S3, Supporting Information, entries 9–13). Notably, the absolute configuration of the corresponding chiral γ‐butyrolactone **3a** was determined by single‐crystal X‐ray diffraction analysis.^[^ ^36^ ^]^

**Table 1:** Initial Studies and the Reaction Optimization.

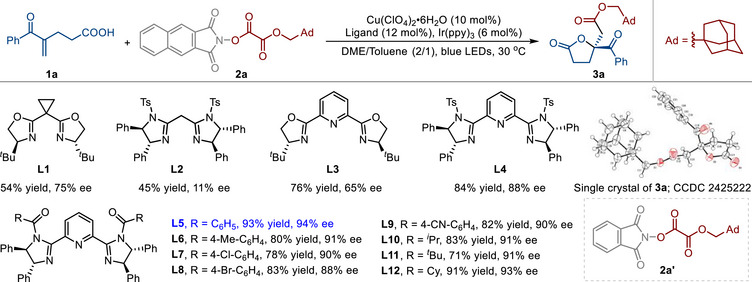			
Entry^a)^	Deviation from standard conditions	Yield of 3a [%]^b)^	ee of 3a [%]^c)^
1	none	93	94
2	CuOTf instead of Cu(ClO_4_)_2_•6H_2_O	79	91
3	[Cu(MeCN)_4_]PF_6_ instead of Cu(ClO_4_)_2_•6H_2_O	89	78
4	Cu(OTf)_2_ instead of Cu(ClO_4_)_2_•6H_2_O	88	93
5	Toluene instead of DME/Toluene	72	91
6	DME instead of DME/Toluene	95	88
7	1,4‐Dioxane instead of DME/Toluene	68	91
8	MeCN instead of DME/Toluene	32	69
9	DMF instead of DME/Toluene	22	61
10	**2a’** instead of **2a**	33	94
11	Without Cu(ClO_4_)_2_•6H_2_O and **L5**	NR	–
12	Without light or Ir(ppy)_3_	NR	–

^a)^ Reaction conditions: **1a** (0.1 mmol), **2a** (0.3 mmol), Ir(ppy)_3_ (0.006 mmol), Cu(ClO_4_)_2_•6H_2_O (0.01 mmol), ligand (0.012 mmol), DME/toluene = 2/1 (3 mL), blue LEDs, 30 °C, 96 h, under a N_2_ atmosphere. DME: 1,2‐Dimethoxyethane;

^b)^ Determined by ^1^H NMR analysis using 1,3,5‐trimethoxybenzene as an internal standard;

^c)^ Determined by HPLC analysis on a chiral stationary phase.

### 2.2 Substrates Scope

With the optimal conditions in hand, we subsequently explored this photoredox/copper dual‐catalyzed asymmetric alkoxycarbonyl‐lactonization reaction. As shown in **Table** **2**a, a series of α,β‐unsaturated enones bearing different aryl groups (**3a**‐**3q**) underwent the desired transformation to afford the corresponding ester‐substituted γ‐butyrolactones in good to excellent yields with excellent enantiomeric excesses. We initially examined the electronic effects of substituents located at the *para*‐position of the phenyl group in the substrates (**3a**‐**3k**). Both electron‐donating groups (‐Me, ‐OMe, ‐*^t^* Bu, ‐NMe_2_) and electron‐withdrawing ones (‐F, ‐Cl, ‐Br, ‐CN, ‐CF_3_) were tolerated well, affording the desired ester‐substituted γ‐butyrolactones in moderate to high yields with excellent enantioselectivities (85−96% ee). Additionally, *meta*‐ and *ortho*‐substituted substrates underwent the transformations smoothly, without any significant reduction in enantioselectivity being observed (**3l**‐**3n**, 91−98% ee). Futhermore, 2‐naphthyl‐substituted α,β‐unsaturated enone could be employed as a suitable substrate, providing the desired product **3o** in 81% yield and 94% ee. Substrates bearing electron‐rich heteroaryl substituents, such as 2‐thienyl (product **3p**, 99% ee) and 2‐furyl (product **3q**, 98% ee) groups, exhibited excellent enantioselectivity. Electron‐rich aryl‐substituted starting materials generally gave better results than their electron‐deficient counterparts. Furthermore, several alkenoic acids with different aryl groups at the alkene moiety were also suitable for this asymmetric alkoxycarbonyl‐lactonization reaction, affording the products in acceptable yields with moderate enantioselectivities (**3r**‐**3s**, 65‐71% ee).

**Table 2:** Substrate Scope of the Enantioselective Alkoxycarbonyl‐Lactonization Reaction.^a)^, ^b)^

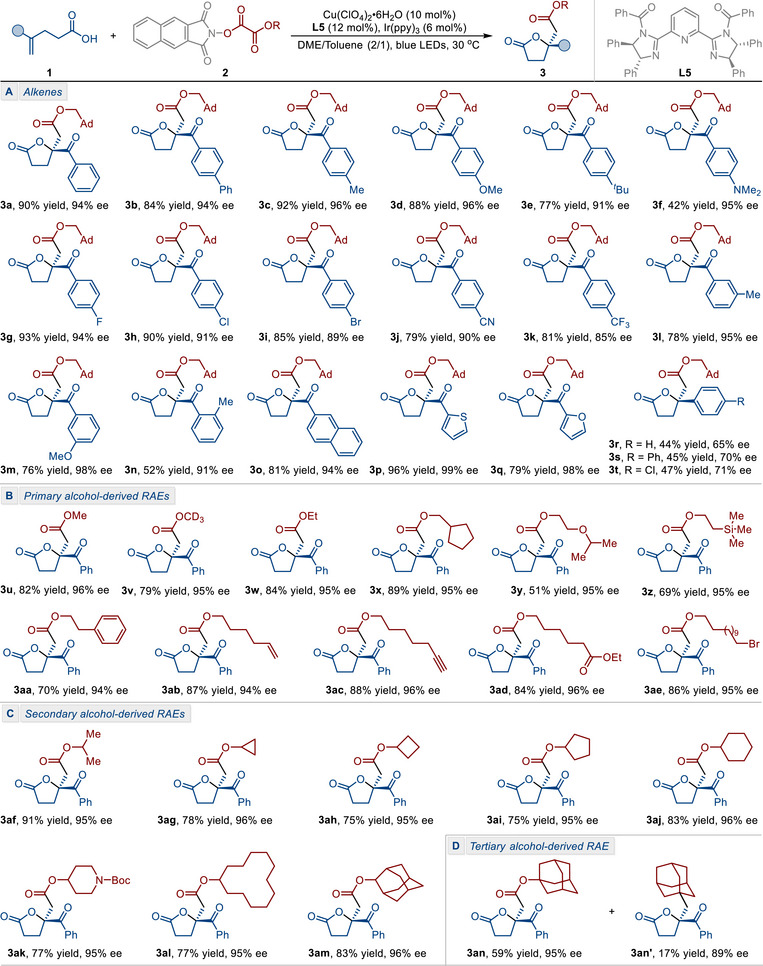

^a)^ Reaction conditions: **1** (0.1 mmol), **2** (0.3 mmol), Ir(ppy)_3_ (0.006 mmol), Cu(ClO_4_)_2_•6H_2_O (0.01 mmol), ligand **L5** (0.012 mmol), DME/toluene = 2/1 (3 mL), blue LEDs, 30 °C, 96 h, under a N_2_ atmosphere;

^b)^ Determined by HPLC analysis on a chiral stationary phase.

Having demonstrated the efficiency of α,β‐unsaturated enones in the asymmetric alkoxycarbonyl‐lactonization reaction, we next evaluated the conversion of various alcohol‐derived RAEs. A series of linear and branched primary alcohol‐derived RAEs were found to be effective for this reaction (Table 2b), affording the desired products **3u**−**3ae** in moderate to excellent yields (51−89%) with excellent enantioselectivities (94−96% ee). Notably, the D‐labeled γ‐butyrolactone **3v** was successfully obtained when a D‐labeled alkoxycarbonyl radical precursor was employed in this reaction. As illustrated in reactions **2ab** and **2ac** (products **3ab** and **3ac**, respectively), the radical‐sensitive terminal alkene and alkyne are fully preserved, thus facilitating further synthetic application of these functional groups. This reaction was also compatible with other alcohol‐derived RAEs bearing different functional groups, including alkoxy (product **3y**, 51% yield and 95% ee), trimethylsilyl (product **3z**, 69% yield and 95% ee), phenyl (product **3aa**, 70% yield and 94% ee), ester (product **3ad**, 84% yield and 96% ee), and bromo (product **3ae**, 86% yield and 95% ee), directly providing the corresponding ester‐substituted γ‐butyrolactones without any undesired by‐products.

We next investigated a variety of secondary alcohol‐derived RAEs. As illustrated in Table 2c, a series of carbocyclic and heterocyclic secondary alcohol derivatives, ranging in size from three‐ to twelve‐membered rings, could efficiently participate in this transformation to provide the corresponding alkoxycarbonyl‐lactonization products. Viable moieties in this transformation included cyclopropyl (product **3ag**, 78% yield and 96% ee), cyclobutyl (product **3ah**, 75% yield and 95% ee), cyclopentyl (product **3ai**, 75% yield and 95% ee), cyclohexyl (product **3aj**, 83% yield and 95% ee), piperidyl (product **3ak**, 77% yield and 95% ee), and cyclododecyl (product **3al**, 77% yield and 95% ee). The reaction proceeded with high efficiency when using 2‐adamantanol derivative (product **3am**, 83% yield and 96% ee) as an alkoxycarbonyl radical precursor. Generally, tertiary alcohol‐derived RAEs have been widely recognized as precursors of alkyl radicals due to their propensity for decarboxylation.^[^ ^37^ ^]^ Although this conversion is a competitive process, using a 1‐adamantanol derivative as the substrate furnished the desired product **3an** (59% yield and 95% ee), along with the carbolactonization product **3an’** (17% yield and 89% ee). (Table 2d).

In the field of pharmaceuticals, co‐drugs are biodegradable chemical derivatives of pharmacologically active compounds that can readily undergo enzymatic and/or chemical transformation in vivo, subsequently releasing two or more distinct active parent drugs. This strategic molecular modification can not only enhance the pharmacokinetic profiles but also preserve the synergistic therapeutic effects between the constituent agents.^[^ ^3^, ^4^, ^5^ ^]^ Ester groups are often explored as advantageous moieties in co‐drug development. We believe that the ester group can serve as a covalent linker, facilitating the integration of alcohol‐containing bioactive molecules into chiral γ‐butyrolactone scaffolds. With this in mind, we next investigated the transformations of various alcohol‐containing biologically active compound‐derived RAEs by employing 4‐benzoylpent‐4‐enoic acid (**1a**) as the substrate under the optimal conditions (**Table** **3**). Trans‐4‐(trans‐4‐propylcyclohexyl)cyclohexanol derivative could react with **1a**, leading to the desired product **3ao** in 82% yield with 95% ee. Cetyl alcohol, a long‐chain aliphatic alcohol, can function as an emulsifying and stiffening agent in pharmaceuticals.^[^ ^38^ ^]^ This method enables the introduction of this structure into the chiral γ‐butyrolactone scaffold, providing the desired product **3ap** in 81% yield with 94% ee. Notably, RAEs derived from other alcohol‐containing bioactive natural products, including (*S*)‐(‐)‐*β*‐citronellol (product **3aq**, 70% yield and 95% de), (‐)‐borneol (product **3ar**, 95 yield and 95% de), (+)‐fenchol (product **3as**, 84 yield and 97% de), (‐)‐menthol (product **3at**, 88 yield and 96% de), and (+)‐menthol (product **3au**, 75 yield and 96% de), were workable as well. Substrates derived from sterically bulky alcohols, such as (‐)‐borneol and (+)‐fenchol (products **3ar** and **3as**), exhibited good yields and excellent stereoselectivities, indicating a minimal influence of steric hindrance on this reaction. Other alcohol‐containing bioactive compounds, such as *D*‐galactopyranose (product **3av**, 75% yield and 98% de), diacetonefructose (product **3aw**, 74% yield and 95% de), and *D*‐ribofuranose (product **3ax**, 82% yield and 95% de), were efficiently incorporated into this strategy. To further validate the versatility of this approach in complex settings, we applied this reaction to the derivatization of steroidal compounds. A diverse array of steroidal moieties, including cholesterol (product **3ay**), dihydrocholesterol (product **3az**), diosgenin (product **3ba**), pregnenolone (product **3bb**), and epiandrosterone (product **3bc**), could also be efficiently incorporated into chiral esters.

**Table 3:** Substrate Scope with Respect to Different Alcohol Derivatives.^a)^, ^b)^

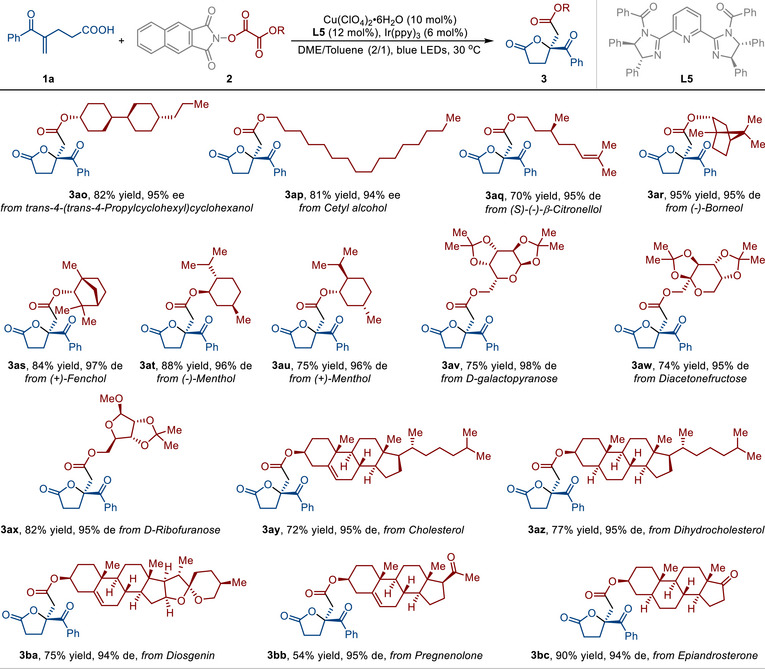

^a)^ Reaction conditions: **1** (0.1 mmol), **2** (0.3 mmol), Ir(ppy)_3_ (0.006 mmol), Cu(ClO_4_)_2_•6H_2_O (0.01 mmol), ligand **L5** (0.012 mmol), DME/toluene = 2/1 (3 mL), blue LEDs, 30 °C, 96 h, under a N_2_ atmosphere;

^b)^ Determined by HPLC analysis on a chiral stationary phase.

**Figure 2:**
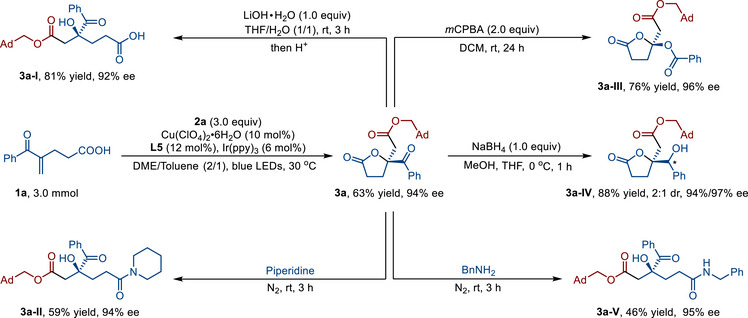
Synthetic applications.

To further demonstrate the practical utility of the photoredox/copper dual‐catalyzed enantioselective alkoxycarbonyl‐lactonization of alkenes, a scale‐up experiment (3.0 mmol) was conducted, affording the corresponding chiral γ‐butyrolactone **3a** in 63% yield with 94% ee (**Figure** **2**). Subsequent hydrolysis of this chiral γ‐butyrolactone **3a** under basic conditions (LiOH•H_2_O) afforded an ester‐substituted γ‐hydroxybutyric acid (**3a‐I**, 81% yield and 92% ee) in good yield. The reaction of **3a** with piperidine enabled the synthesis of the chiral γ‐hydroxybutyramide **3a‐II**, achieving moderate yield (59%) while maintaining excellent enantioselectivity (94% ee). Furthermore, Baeyer‐Villiger oxidation of compound **3a** provided a versatile and efficient methodology to access the chiral ketal derivative **3a‐III** (76% yield and 96% ee). Treatment of γ‐butyrolactone **3a** with sodium borohydride in THF yielded the chiral hydroxylactone **3a‐IV** in excellent yield (88% yield). Similarly, when reacted with benzylamine, ester‐substituted γ‐butyrolactone **3a** afforded the corresponding chiral γ‐hydroxyamide **3a‐V** with an extra benzyl group in moderate yield and with excellent enantioselectivity (46% yield and 95% ee).

### 2.3 Mechanistic Studies

**Figure 3:**
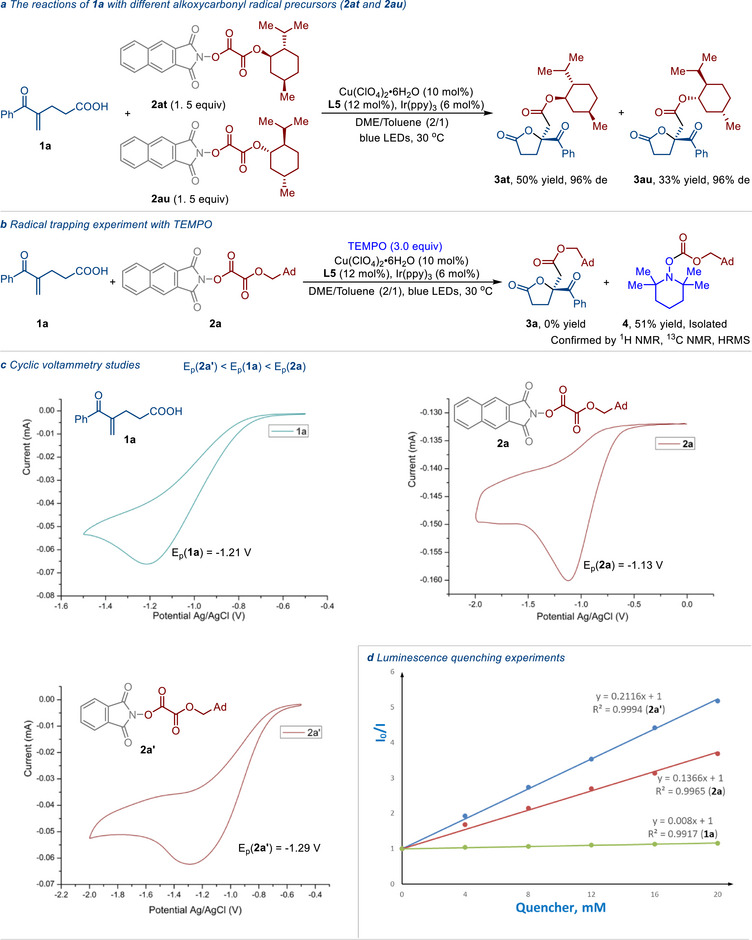
Mechanistic studies. a) The reactions of **1a** with different alkoxycarbonyl radical precursors (**2at** and **2au**). b) Radical trapping experiment with TEMPO. c) Cyclic voltammetry studies. d) Luminescence quenching experiments.

A comparative experiment was designed and conducted to confirm the influence of the chiral alkoxycarbonyl radical in the current reaction (**Figure** **3**a). When the mixture of (‐)‐menthol‐ and (+)‐menthol‐derived alkoxycarbonyl radical precursors (**2at** and **2au**) was treated with **1a** in the presence of the catalyst system, the alkoxycarbonyl‐lactonization products **3at** and **3au** were obtained in different yields but with the same diastereoselectivity. This result indicates that the chiral alkoxycarbonyl radical has no significant effect on the stereoselective formation of the C−O bond. When the radical scavenger 2,2,6,6‐tetramethylpiperidinyloxy (TEMPO) was added under standard conditions, the formation of compound **3a** was completely inhibited. Additionally, the radical‐trapping adduct **4** was isolated in 51% yield (Figure 3b). It is noteworthy that the *N*‐hydroxy 2,3‐naphthalimide‐derived RAE **2a** was crucial for the success of this transformation (**3a**, 93% yield and 94% ee), while the use of *N*‐phthalimidoyl oxalate **2a’** provided only a small amount of **3a** (33% yield and 94% ee). The reduction potential of **2a** measured by cyclic voltammetry in DME is E_p_ (**2a**) = − 1.13 V vs. Ag/AgCl^[^ ^39^ ^]^ and is slightly less negative than that of 4‐benzoylpent‐4‐enoic acid E_p_ (**1a**) = −1.21 V vs. Ag/AgCl.^[^ ^40^ ^]^ In contrast, the reduction potential of **2a’** [E_p_ (**2a’**) = −1.29 V vs Ag/AgCl] is more negative than that of **1a** (Figure 3c).^[^ ^15^ ^]^ It means that **1a** can be more readily reduced by the excited photocatalyst than **2a’**. Stern‐Volmer experiments revealed that both RAE **2a** and 4‐benzoylpent‐4‐enoic acid (**1a**) could quench the excited photocatalyst *Ir(ppy)_3_ (Figure 3d). Based on the Stern−Volmer equation, *I₀/I* = 1 + k_q_τ_0_[Q], the quenching rate constant for *Ir(ppy)_3_ with RAE **2a** was determined to be k_q_(**2a**) = 7.2 × 10⁷ M^−1^s^−1^. This value is ≈17 times higher than that of the quenching reaction between *Ir(ppy)_3_ and **1a**.

**Figure 4:**
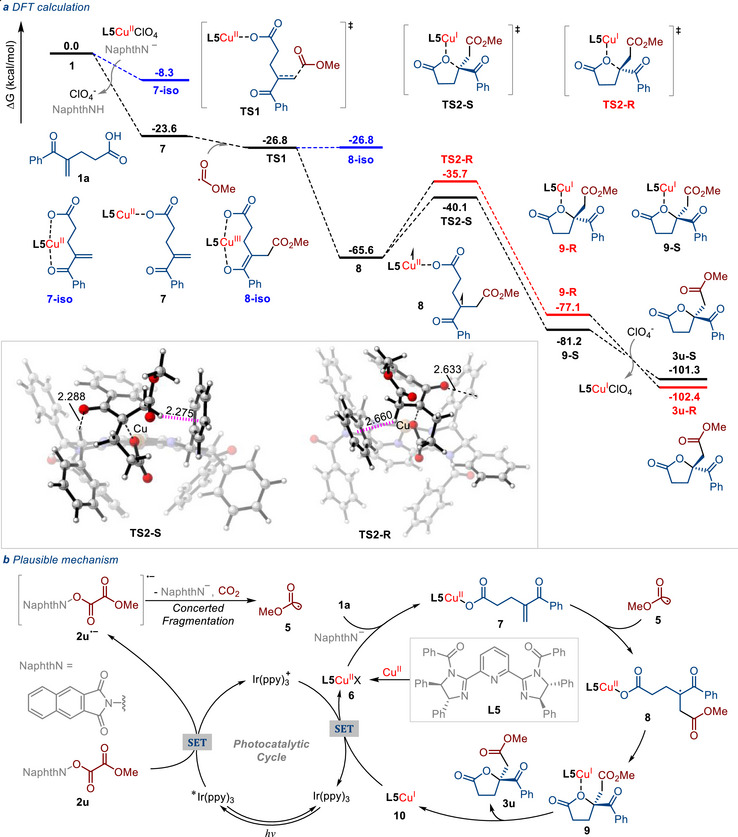
Plausible mechanism for photoredox/copper dual‐catalyzed enantioselective alkoxycarbonyl‐lactonization. a) DFT calculation. b) Plausible mechanism.

We conducted DFT calculation to investigate the photoredox and copper‐catalyzed mechanism for this enantioselective alkoxycarbonyl‐lactonization reaction (**Figure** **4**a). Under the 2,3‐naphthalimide anion (NaphthN^−^), the reactant 4‐benzoylpent‐4‐enoic acid **1a** undergoes the ligand change with **L5**Cu^II^ClO_4_ to form the intermediates **7** and **7‐iso**. The four‐coordinated intermediate **7** (−23.6 kcal mol^−1^) is favored compared to five‐coordinated **7‐iso** (−8.3 kcal mol^−1^). Then, the alkoxycarbonyl radical **5** additions to the C═C bond of intermediates **7** through the transition state **TS1** (−26.8 kcal mol^−1^), leading to the *α*‐carbonyl radical **8**. We calculated the Cu^III^ species **8‐iso** and *triplet‐state* Cu^II^ species **8**, whose free energy is significantly lower than that of Cu^III^ species **8‐iso** by 38.8 kcal mol^−1^. Following the alkoxycarbonyl radical addition, *triplet‐state* Cu^II^ species **8** proceeds via transition states **TS2‐R** (−35.7 kcal mol^−1^) and **TS2‐S** (−40.1 kcal mol^−1^). The favored **TS2‐S** leads to the major intermediate **9‐S**, which is 4.6 kcal/mol lower than of **TS2‐R**. Subsequently, intermediate **9‐S** (−81.2 kcal mol^−1^) undergoes the ligand change to generate major product **3u‐S** (−101.3 kcal mol^−1^). Additionally, the interaction analysis of transition states confirmed the effects of hydrogen bond and weak H∙∙∙π interaction, which are the driving factors for the enantiocontrol of the radical reduction.

Based on the aforementioned experimental results and relevant literature reports,^[^ ^14^, ^15^, ^16^, ^17^, ^18^, ^41^, ^42^, ^43^, ^44^ ^]^ a dual photoredox and copper‐catalyzed mechanism for this enantioselective alkoxycarbonyl‐lactonization reaction is proposed, as illustrated in Figure 4b. The reaction starts with a single‐electron reduction of the *N*‐hydroxy 2,3‐naphthalimide‐derived redox‐active ester **2u** by the excited‐state photocatalyst *Ir(ppy)_3_ [*E*_1/2_[Ir(ppy)_3_^+^/*Ir(ppy)_3_] = −1.73 V *vs*. saturated calomel electrode (SCE)] to generate the corresponding radical anion **2u** ^•−^ and oxidized photocatalyst Ir(ppy)_3_^+^. Then, **2u** ^•−^ can undergo concerted fragmentation to form the desired alkoxycarbonyl radical **5**, with concomitant release of 2,3‐naphthalimide anion and CO_2_. On the other hand, the generated 2,3‐naphthalimide anion can promote the ligand exchange reaction between the **L5**‐Cu^II^ complex **6** and reactant **1a**, leading to the formation of species **7**. Next, the corresponding alkoxycarbonyl radical **5** adds to species **7**, resulting in the formation of an *α*‐carbonyl radical **8**. Subsequently, species **8** can be converted into the **L5**‐Cu^II^ complex **9** via an intramolecular radical cyclization process. The dissociation of species **9** results in the formation of the desired product **3u**, accompanied by the generation of the **L5**‐Cu^I^ complex **10**. Finally, the single‐electron transfer (SET) from the **L5**‐Cu^I^ complex **10** [*E*_1/2_^red^ = −0.08 V vs SCE for Cu(I)] to the oxidized photocatalyst Ir(ppy)_3_^+^ (*E*_1/2_[Ir(ppy)_3_^+^/Ir(ppy)_3_] = + 0.77 V vs SCE) enables the regeneration of the ground‐state photocatalyst Ir(ppy)_3_ and **L5**‐Cu^II^ complex **6,** thereby preparing for the next catalytic cycle.

## 3 Conclusion

In summary, we have successfully developed a highly enantioselective alkoxycarbonyl‐lactonization of alkenes through a photoredox/copper dual‐catalyzed alkoxycarbonyl radical addition/ cyclization reaction. With this approach, a diverse range of ester‐substituted chiral γ‐butyrolactones bearing a quaternary stereocenter can be readily obtained in high yields and with excellent enantioselectivities under mild reaction conditions. This versatile method shows good substrate tolerance for primary, secondary, and tertiary alcohol derivatives while maintaining exceptional stereochemical control. Additionally, this strategy represents a significant approach for integrating a diverse range of alcohol‐containing bioactive molecules into chiral γ‐butyrolactone scaffolds. Chiral γ‐butyrolactone structures are widely present in many natural products and biologically active molecules. We believe that the integration of pharmacologically active ester motifs into chiral γ‐butyrolactone scaffolds can offer a pioneering approach for modern drug discovery.

## Conflict of Interest

The authors declare no conflict of interest.

## Author Contributions

J.‐Q.C. and J.W. conceived and supervised the whole project and wrote the paper with input from all authors. J.‐Q.C. and J.W. designed and discussed the experiments. Y.X., Z.Z., W.X., and J.‐Q.C. performed and analyzed the experiments. X.Z. performed the DFT calculations.

## Supporting information



Supporting Information

## Data Availability

The data that support the findings of this study are available in the supplementary material of this article.
