# Supplementary material for: Enantioselective Alkoxycarbonyl‐Lactonization of Alkenes via the Merger of Photoredox and Copper Catalysis
Source: Adv Sci (Weinh). 2025 Aug 13;12(42):e10918. doi: 10.1002/advs.202510918 (PMC12622489; doi:10.1002/advs.202510918)
Supplement: Supplementary file 1 — Supporting Information [file ADVS-12-e10918-s001.pdf]

Supplementary Information

Enantioselective Alkoxycarbonyl-Lactonization of Alkenes via the Merger of Photoredox and Copper Catalysis

Yuping Xiong[1,2], Zhipeng Zong[1], Wenlin Xie[2], Jian-Qiang Chen[1]*, Xiaoyu Zhou[1]*, and Jie Wu[1,3]*

[1]*School of Pharmaceutical and Chemical Engineering & Institute for Advanced Studies, Taizhou University, Taizhou 318000, China.*
[2]*School of Chemistry and Chemical Engineering, Hunan University of Science and Technology, Xiangtan 411201, China.*
[3]*State Key Laboratory of Organometallic Chemistry, Shanghai Institute of Organic Chemistry, Chinese Academy of Sciences, Shanghai 200032, China*

Contents

1. General information..2
2. General experimental procedure...3
3. Initial studies and the reaction optimization..8
4. Devices for the photocatalytic reactions..10
5. Mechanistic studies...12
6. X-ray data for compound **3a**..16
7. Computational details...17
8. Characterization of products..35
9. HPLC data and chromatograms...70
10. NMR spectra of compounds 132
11. Supplementary references...197

1. General information

All glassware was thoroughly oven-dried. Chemicals and solvents were either purchased from commercial suppliers (*Adamas-beta, Leyan, J&K Scientific, TCl, Aldrich, Energy Chemical, Alfa Aesar*) or purified by standard techniques. Thin-layer chromatography plates were visualized by exposure to ultraviolet light and/or staining with phosphomolybdic acid followed by heating on a hot plate. Flash chromatography was carried out using silica gel (200−300 mesh). ^{1}H NMR and ^{13}C NMR spectra were recorded on a Bruker AM-400 (400 MHz). The spectra were recorded in deuterochloroform ($CDCl_3$) as solvent at room temperature, ^{1}H and ^{13}C NMR chemical shifts are reported in ppm relative to the residual solvent peak. The residual solvent signals were used as references and the chemical shifts were converted to the TMS scale ($CDCl_3$: δ_H = 7.26 ppm, δ_C = 77.0 ppm). Data for ^{1}H NMR are reported as follows: chemical shift (δ ppm), multiplicity (s = singlet, d = doublet, t = triplet, m = multiplet, dd = doublet, br = broad), integration, coupling constant (Hz) and assignment. Data for ^{13}C NMR are reported as chemical shift. Electrospray–ionisation HRMS data were acquired on a Q–TOF mass spectrometer (Waters SYNAPT G2-Si) LC-MS TOF. All luminescence spectra were surveyed on a Cary Eclipse fluorescence spectrophotometer and equipped with a 1 cm quartz cell. Cyclic voltammetry (CV) studies were carried out on a CHI760E instrument. X-ray crystallography analysis of single crystal was performed on an Agilent SuperNova-CCD X-Ray diffractometer. Enantioselectivities were recorded on Agilent and Waters HPLC using CHIRALPAK columns. The chiral stationary phase was Daicel Chiralpak IA, IB, ID, IK, AD-H, AS-H, AY-H, AZ-H, OD-H, or OX-H column. Chiralpak IA, IB, ID, IK, AD-H, AS-H, AY-H, AZ-H, OD-H, or OX-H columns were purchased from Daicel Chemical Industries (Shanghai, China). UV absorption was monitored at 243-309 nm.

2. General experimental procedure[1]

*(a) General procedure for the synthesis of substrates **1**.*[1]

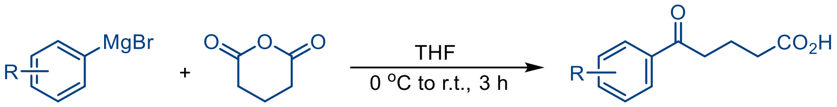

To the solution of glutaric anhydride (1.14 g, 10 mmol, 1.0 equiv.) in THF (30 mL) under a N_2 atmosphere was added dropwise the corresponding Grignard reagent (12 mmol, 1.2 equiv.) at 0 °C. The solution was warmed to room temperature and stirred for a further 3 hours. The reaction was quenched with 10% HCl, and THF was removed under vacuum. The resulting aqueous solution was extracted with DCM. The combined organic layers were washed with brine, dried (Na_2SO_4) and evaporated under vacuum to give a white solid and used without other purification.

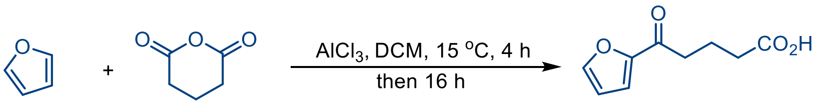

To a solution of glutaric anhydride (1.4 g, 12 mmol, 1.2 equiv.) in DCM (125 mL) in a 250 mL round-bottom flask fitted with a thermometer, and solvent addition funnel was added aluminum trichloride (2.3 g, 17 mmol, 1.7 equiv.). The reaction mass was cooled under stirring to 15 °C and a solution of furan (0.68 g, 10 mmol, 1.0 equiv.) in 10 mL of DCM was added dropwise and the reaction mixture was stirred for 16 h at rt. The reaction was poured in ice and 10 mL of concentrated hydrochloric acid was added under stirring at 0 °C. The organic layer was separated and the aqueous layer was extracted with DCM twice (2 × 50 mL). The combined organic layers were washed with water, dried over $MgSO_4$ and concentrated to give the expected compound and used without other purification.

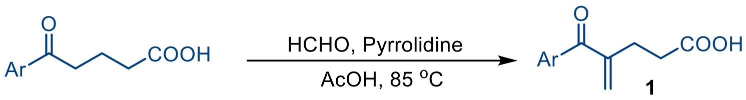

To a solution of keto acid (3.0 mmol, 1.0 equiv.) in acetic acid (6 mL) was added pyrrolidine (85.3 mg, 1.2 mmol, 0.4 equiv.) and formaldehyde solution (36.5-38% in H_2O, 13.2 mmol, 4.4 equiv.) at room temperature. The mixture was then stirred for 48 h at 85 °C. After evaporation of acetic acid, water and EtOAc were added. The organic

layer was washed with water, and dried over magnesium sulfate. Concentration of the organic layer offered the crude product that was further purified by flash column chromatography (hexane/EtOAc) to give the corresponding compound **1**.

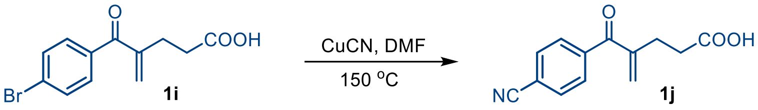

A 10 mL round-bottom flask charged with 4-(4-bromobenzoyl)pent-4-enoic acid (562 mg, 2.0 mmol, 1.00 equiv.) followed by addition of CuCN (269 mg, 3.0 mmol, 1.5 equiv.) and anhydrous DMF (2 mL). The resulting mixture was stirred at 150 °C for 14 hours. The mixture was allowed to cool and was acidified with 1M hydrochloric acid. The resulting solution was extracted with EA. The organic layers were washed with brine, dried (Na_2SO_4) and evaporated under vacuum to give the 4-(4-cyanobenzoyl)pent-4-enoic acid (348 mg, 76%).[1d]

*(b) General procedure for the synthesis of substrates **2**.*[2]

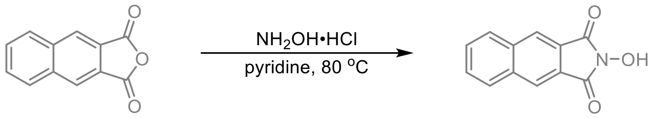

To a solution of substituted naphtho[2,3-c]furan-1,3-dione (6.9 g, 35.0 mmol, 1.0 equiv.) and hydroxylamine hydrochloride (4.8 g, 70.0 mmol, 2.0 equiv.) in pyridine (28 mL) was heated at 80 °C for 4 h and then cooled to room temperature. The mixture was added 130 mL water and acidified to pH = 2 with concentrated HCl. The precipitate was filtrated and washed by water. The filter was dried in vacuo and purified by recrystallization with EtOH.

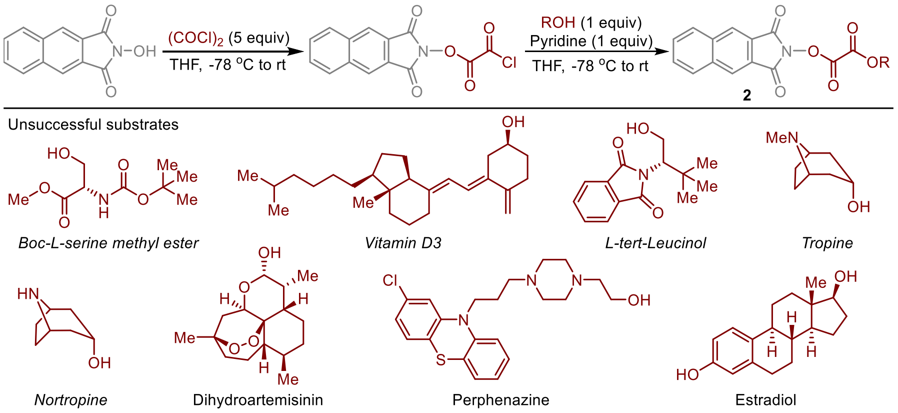

A round-bottom flask was charged with 2-hydroxy-1*H*-benzo[*f*]isoindole-1,3(2*H*)-dione (4.26 g, 20 mmol, 1 equiv.), followed by the addition of THF (200 mL). The resulting solution was then cooled to –78 °C and oxalyl chloride (12.6 g, 100 mmol,

5.0 equiv.) was added dropwise. The solution was then allowed to warm to room temperature and stirred for 20 h. The volatiles were removed under reduced pressure to yield a colorless solid. Then a round-bottom flask was charged with the colorless solid followed by the addition of THF (200 mL). The mixture was cooled to −78 °C and a solution of ROH (20 mmol, 1.0 equiv.), pyridine (1.6 g, 20 mmol, 1.0 equiv.) in THF (50 mL) was added dropwise. The resulting heterogeneous mixture was warmed to 0 ºC and allowed to stir for 1 h. The reaction was then allowed to warm to 23 ºC and stirred for another 30 min. The reaction mixture was concentrated under reduced pressure, and the resulting crude residue was dissolved in CH_2Cl_2 (100 mL) and washed with sat. aq. $CuSO_4$ (3 x 100 mL). The organic layer was dried over $MgSO_4$ and concentrated under reduced pressure. The resulting crude residue was purified by recrystallization with CH_2Cl_2/pentanes to afford the desired product **2**.

(c) General procedure for the synthesis of ***3****.*

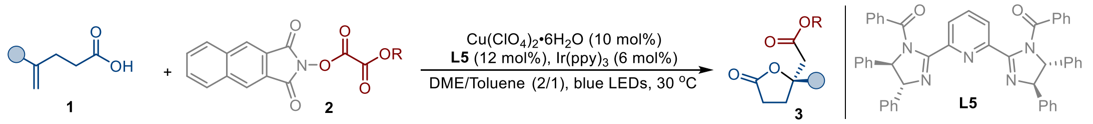

In the nitrogen glovebox, $Cu(ClO_4)_2$ • $6H_2O$ (3.7 mg, 0.010 mmol, 10 mol%) and L5 (8.7 mg, 0.012 mmol, 12 mol%) were dissolved in DME/Toluene (2:1, 3 mL) in a dried sealed tube under nitrogen atmosphere, and the mixture was stirred for 30 minutes. Then **1** (0.1 mmol, 1.0 equiv.), **2** (0.3 mmol, 3.0 equiv.) and Ir(ppy)$_3$ (3.9 mg, 0.006 mmol, 6 mol%) were added to the mixture. Sequentially, the heterogenous mixture was placed in the irradiation apparatus equipped with blue LEDs. The resulting mixture was stirred at 30 ºC for 96 h. Upon completion of the reaction, the mixture was concentrated under reduced pressure and purified by column chromatography on silica gel to afford the desired product **3**.

(d) Scale-up synthesis and further transformation.[3]

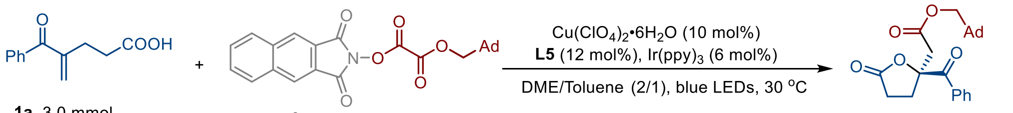

1a, 3.0 mmol **2a,** 9.0 mmol **3a**, 63% yield, 94% ee

In the nitrogen glovebox, $Cu(ClO_4)_2$ • $6H_2O$ (111.2 mg, 0.3 mmol, 10 mol%) and

L5 (261.8 mg, 0.36 mmol, 12 mol%) were dissolved in DME/Toluene (2:1, 90.0 mL) in a dried sealed tube under nitrogen atmosphere, and the mixture was stirred for 30 minutes. Then **1a** (612 mg, 3.0 mmol, 1.0 equiv.), **2a** (3.897 g, 9.0 mmol, 3.0 equiv.) and Ir(ppy)$_3$ (117.7 mg, 0.18 mmol, 6 mol%) were added to the mixture. Sequentially, the heterogenous mixture was placed in the irradiation apparatus equipped with blue LEDs. The resulting mixture was stirred at 30 ºC for 6 d. Upon completion of the reaction, the mixture was concentrated under reduced pressure and purified by column chromatography on silica gel to afford the desired product **3a** (748.4 mg, 63% yield, 94% ee).

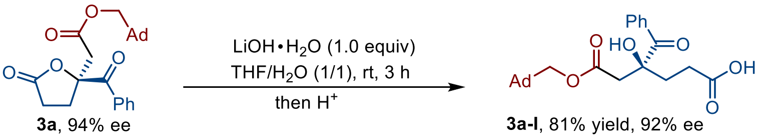

3a, 94% ee　　**3a-I**, 81% yield, 92% ee

The **3a** (39.6 mg, 0.1 mmol, 1.0 equiv.) was dissolved in THF (1 mL). A solution of LiOH·H$_2$O (4.2 mg, 0.1 mmol, 1.0 equiv.) in H$_2$O (1 mL) was added, and the mixture stirred at room temperature for 3 h. Then the mixture was acidified with 1M HCl (5 mL) and was extracted with ethyl acetate (3×10 mL). Then the combined organic layers were washed with brine, dried over anhydrous Na$_2$SO$_4$, and concentrated under reduced pressure. The residue was purified through column chromatography on silica gel (PE/EA = 1:1) to afford to afford **3a-I** as a colorless oil (33.6 mg, 81% yield, 92% ee).

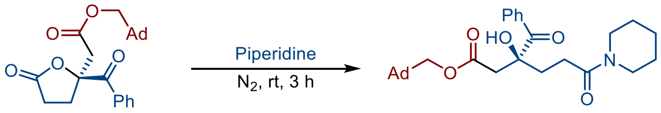

3a, 94% ee　　**3a-II**, 59% yield, 94% ee

Piperidine (0.5 mL, 5.05 mmol, 50.5 equiv.) was added dropwise to **3a** (39.6 mg, 0.1 mmol, 1.0 equiv.) under nitrogen atmosphere. The mixture was allowed to react at room temperature for 3 h. Then the additional piperidine was removed under reduced pressure and the mixture was purified by silica gel chromatography (PE/EA = 2:1) to afford **3a-II** as a colorless oil (56.8 mg, 59% yield, 94% ee).

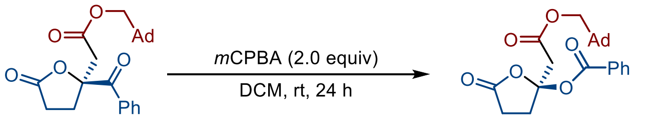

3a, 94% ee　　**3a-III**, 76% yield, 96% ee

To a flame dried 10 mL Schlenk tube equipped with a magnetic stir bar, were added

compound **3a** (39.6 mg, 0.1 mmol, 1.0 equiv.) and *m*CPBA (34.5 mg, 0.2 mmol, 2.0 equiv.) and DCM (2.0 mL, 0.05 M) under N_2 atmosphere. Resulting mixture was stirred at rt for 24 h. The reaction was then quenched with saturated $NaHCO_3$ solution (5.0 mL) and the aqueous phase was extracted with DCM (3x 10 mL). The combined organic layer was washed with water, dried over anhydrous Na_2SO_4, filtered and concentrated under reduced pressure. The crude product was purified by column chromatography (PE/EA = 4:1) to afford **3a-III** as a colorless oil (31.3 mg, 76% yield, 96% ee).

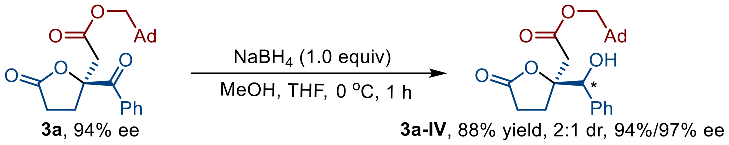

To a flame dried 10 mL Schlenk tube equipped with a magnetic stir bar, were added compound **3a** (39.6 mg, 0.1 mmol, 1.0 equiv.) and anhydrous THF (2.0 mL, 0.05 M) under N_2 atmosphere. The solution was cooled to 0 ºC. To the cold solution, MeOH (10 µL) and $NaBH_4$ (4.0 mg, 0.1 mmol, 1.0 equiv.) were added portion-wise. The resulting solution was allowed to stir at 0 ºC for 1 h. After complete consumption of **3a**, the mixture was warmed to room temperature and concentrated under reduced pressure. To the solid residue, EtOAc (5.0 mL) was added and the solution was neutralized with 2 N HCl (1.0 mL). The organic layer was separated and aqueous layer was extracted with EtOAc (3 x 10 mL). The combined organic layer was dried over anhydrous Na_2SO_4, filtered and concentrated under reduced pressure. The crude material was purified by column chromatography (PE/EA= 3:1) to afford **3a-IV** as a colorless oil (35.0 mg, 88% yield, 2:1 dr, 94%/97% ee).

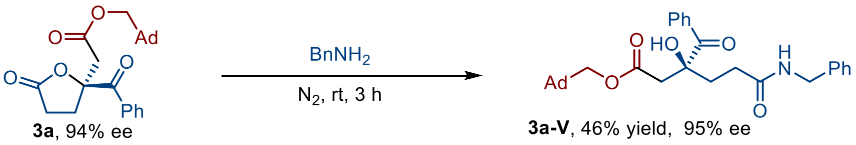

Benzylamine (0.4 mL) was added dropwise to **3a** (39.6 mg, 0.1 mmol, 1.0 equiv.) under nitrogen atmosphere. The mixture was allowed to react at room temperature for 3 h. After complete consumption of **3a**, the mixture was purified by silica gel chromatography (PE/EA= 2:1) to afford **3a-V** (23.2 mg, 46% yield, 95% ee).

3. Initial studies and the reaction optimization

Table S1. Initial studies for the reaction of **1a** with **2w'**.[a]

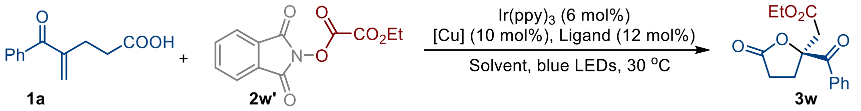

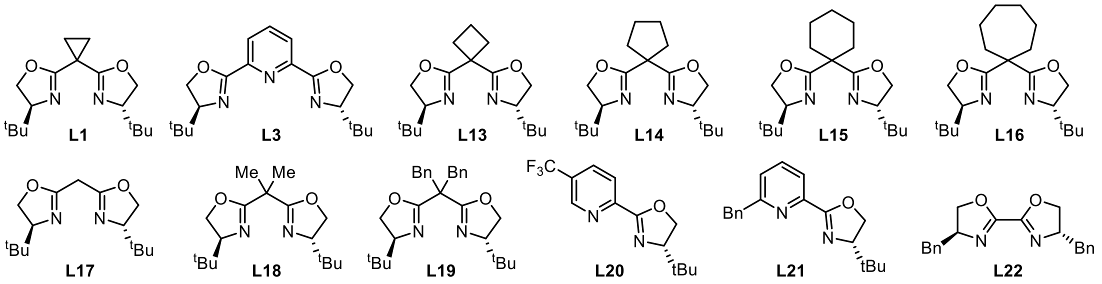

Entry	Ligand	[Cu]	Solvent	Yield (%)[b]	ee (%)[c]
1	**L1**	$Cu(OTf)_2$	DME	83	60
2	**L3**	$Cu(OTf)_2$	DME	48	34
3	**L13**	$Cu(OTf)_2$	DME	76	44
4	**L14**	$Cu(OTf)_2$	DME	74	39
5	**L15**	$Cu(OTf)_2$	DME	71	14
6	**L16**	$Cu(OTf)_2$	DME	78	31
7	**L17**	$Cu(OTf)_2$	DME	87	35
8	**L18**	$Cu(OTf)_2$	DME	89	34
9	**L19**	$Cu(OTf)_2$	DME	91	40
10	**L20**	$Cu(OTf)_2$	DME	92	5
11	**L21**	$Cu(OTf)_2$	DME	99	0
12	**L22**	$Cu(OTf)_2$	DME	81	0
13	**L1**	$[Cu(MeCN)_4]PF_6$	DME	72	53
14	**L1**	$[Cu(MeCN)_4]BF_4$	DME	51	59
15	**L1**	CuOTf	DME	78	60
16	**L1**	$Cu(OAc)_2$	DME	60	16
17	**L1**	$CuSO_4$	DME	Trace	-
18	**L1**	$CuBr_2$	DME	Trace	-
19	**L1**	$Cu(TFSI)_2$	DME	58	31
20	**L1**	$Cu(ClO_4)_2$•$6H_2O$	DME	85	63
21	**L1**	$Cu(ClO_4)_2$•$6H_2O$	MeCN	18	33
22	**L1**	$Cu(ClO_4)_2$•$6H_2O$	DCE	29	34
23	**L1**	$Cu(ClO_4)_2$•$6H_2O$	THF	44	58
24	**L1**	$Cu(ClO_4)_2$•$6H_2O$	Toluene	24	66
25[d]	**L1**	$Cu(ClO_4)_2$•$6H_2O$	DME/Toluene (1/1)	17	68
26[d]	**L1**	$Cu(ClO_4)_2$•$6H_2O$	DME/Toluene (2/1)	40	68

[a]Reaction conditions: **1a** (0.1 mmol), **2w'** (0.3 mmol), $Ir(ppy)_3$ (0.006 mmol), [Cu] (0.01 mmol), ligand (0.012 mmol), solvent (2 mL), blue LEDs, 30 °C, 72 h, under a N_2 atmosphere. DME: 1,2-Dimethoxyethane. [b]Determined by ^1H NMR analysis using 1,3,5-trimethoxybenzene as an internal standard. [c]Determined by HPLC analysis on a chiral stationary phase. [d]Solvent (3 mL).

Table S2. Initial studies for the reaction of **1a** with **2a**.[a]

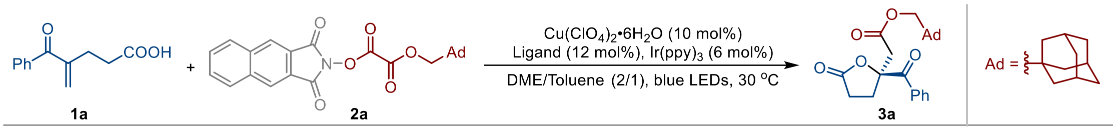

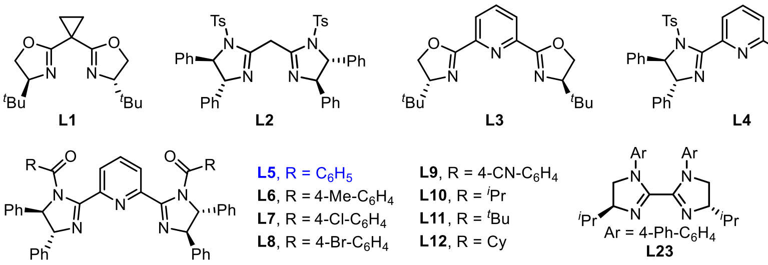

Entry	Ligand	[Cu]	Solvent	Yield (%)[b]	ee (%)[c]
1[d]	**L1**	$Cu(ClO_4)_2{\bullet}6H_2O$	DME/Toluene (2/1)	54	75
2	**L2**	$Cu(ClO_4)_2{\bullet}6H_2O$	DME/Toluene (2/1)	45	11
3	**L3**	$Cu(ClO_4)_2{\bullet}6H_2O$	DME/Toluene (2/1)	76	65
4	**L4**	$Cu(ClO_4)_2{\bullet}6H_2O$	DME/Toluene (2/1)	84	88
5	**L5**	$Cu(ClO_4)_2{\bullet}6H_2O$	DME/Toluene (2/1)	93	94
6	**L6**	$Cu(ClO_4)_2{\bullet}6H_2O$	DME/Toluene (2/1)	80	91
7	**L7**	$Cu(ClO_4)_2{\bullet}6H_2O$	DME/Toluene (2/1)	78	90
8	**L8**	$Cu(ClO_4)_2{\bullet}6H_2O$	DME/Toluene (2/1)	83	88
9	**L9**	$Cu(ClO_4)_2{\bullet}6H_2O$	DME/Toluene (2/1)	82	90
10	**L10**	$Cu(ClO_4)_2{\bullet}6H_2O$	DME/Toluene (2/1)	83	91
11	**L11**	$Cu(ClO_4)_2{\bullet}6H_2O$	DME/Toluene (2/1)	71	91
12	**L12**	$Cu(ClO_4)_2{\bullet}6H_2O$	DME/Toluene (2/1)	91	93
13	**L23**	$Cu(ClO_4)_2{\bullet}6H_2O$	DME/Toluene (2/1)	55	0
14	**L5**	CuOTf	DME/Toluene (2/1)	79	91
15	**L5**	$Cu(OTf)_2$	DME/Toluene (2/1)	88	93
16	**L5**	$[Cu(MeCN)_4]PF_6$	DME/Toluene (2/1)	89	78
17	**L5**	$[Cu(MeCN)_4]BF_4$	DME/Toluene (2/1)	87	75
18	**L5**	$Cu(ClO_4)_2{\bullet}6H_2O$	Toluene	72	91
19	**L5**	$Cu(ClO_4)_2{\bullet}6H_2O$	DME	95	88
20	**L5**	$Cu(ClO_4)_2{\bullet}6H_2O$	DMF	22	61
21	**L5**	$Cu(ClO_4)_2{\bullet}6H_2O$	MeCN	32	69
22	**L5**	$Cu(ClO_4)_2{\bullet}6H_2O$	DCM	51	88
23	**L5**	$Cu(ClO_4)_2{\bullet}6H_2O$	1,4-Dioxane	68	91
24	-	-	DME/Toluene (2:1)	NR	-
25[d]	**L5**	$Cu(ClO_4)_2{\bullet}6H_2O$	DME/Toluene (2:1)	NR	-
26[e]	**L5**	$Cu(ClO_4)_2{\bullet}6H_2O$	DME/Toluene (2:1)	NR	-

[a]Reaction conditions: **1a** (0.1 mmol), **2a** (0.3 mmol), $Ir(ppy)_3$ (0.006 mmol), [Cu] (0.01 mmol), ligand (0.012 mmol), solvent (3 mL), blue LEDs, 30 °C, 96 h, under a N_2 atmosphere. DME: 1,2-Dimethoxyethane. [b]Determined by ^1H NMR analysis using 1,3,5-trimethoxybenzene as an internal standard. [c]Determined by HPLC analysis on a chiral stationary phase. [d]Without $Ir(ppy)_3$. [e]In the dark.

Table S3. Initial studies and the reaction optimization.[a]

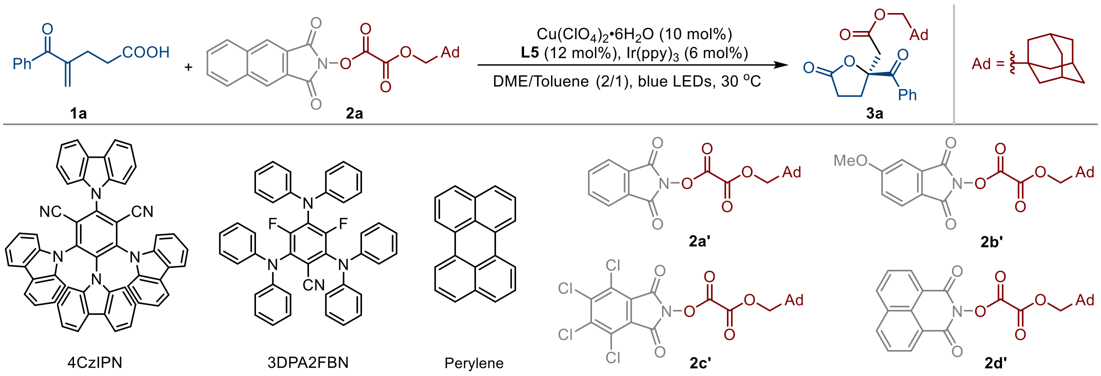

Entry	Deviation from standard conditions	Yield of **3a** (%)[b]	ee of **3a** (%)[c]
1	none	93	94
2	4CzIPN instead of $Ir(ppy)_3$	NR	-
3	3DPA2FBN instead of $Ir(ppy)_3$	NR	-
4[d]	Perylene instead of $Ir(ppy)_3$	NR	-
5	**2a'** instead of **2a**	33	94
6	**2b'** instead of **2a**	Trace	-
7	**2c'** instead of **2a**	NR	-
8	**2d'** instead of **2a**	NR	-
9	$Ir(ppy)_3$ (4 mol%)	91	89
10	$Ir(ppy)_3$ (2 mol%)	84	89
11	$Ir(ppy)_3$ (1 mol%)	64	89
12	40 °C, 72 h	72	91
13	50 °C, 72 h	65	90
14	$[Cu(MeCN)_4]PF_6$ instead of $Cu(ClO_4)_2$•$6H_2O$, without $Ir(ppy)_3$	NR	-
15	CuOTf instead of $Cu(ClO_4)_2$•$6H_2O$, without $Ir(ppy)_3$	NR	-

[a]Reaction conditions: **1a** (0.1 mmol), **2a** (0.3 mmol), $Ir(ppy)_3$ (0.006 mmol), $Cu(ClO_4)_2$•$6H_2O$ (0.01 mmol), ligand (0.012 mmol), DME/toluene = 2/1 (3 mL), blue LEDs, 30 °C, 96 h, under a N_2 atmosphere. DME: 1,2-Dimethoxyethane. [b]Determined by ^1H NMR analysis using 1,3,5-trimethoxybenzene as an internal standard. [c]Determined by HPLC analysis on a chiral stationary phase. [d]390 nm Kessil lamp. photocatalyst (0.01 mmol).

4. Devices for the photocatalytic reactions

Irradiation of visible light was performed with a 36 W Blue LED strip. All photocatalyzed alkoxycarbonylation/Cyanation reactions were carried out at room temperature (r.t.) with fan-assisted cooling to maintain a temperature of approximately 30 °C. The distance between tube and lamp was approximately 3 cm.

Manufacture of the light source: LED strip

Manufacturer: Greethink

Model: GT-5050-Blue

Wavelength of peak intensity: 460-470 nm

Material of the irradiation vessel: borosilicate glass

Distance of the irradiation vessel from the light source: approximately 3 cm.

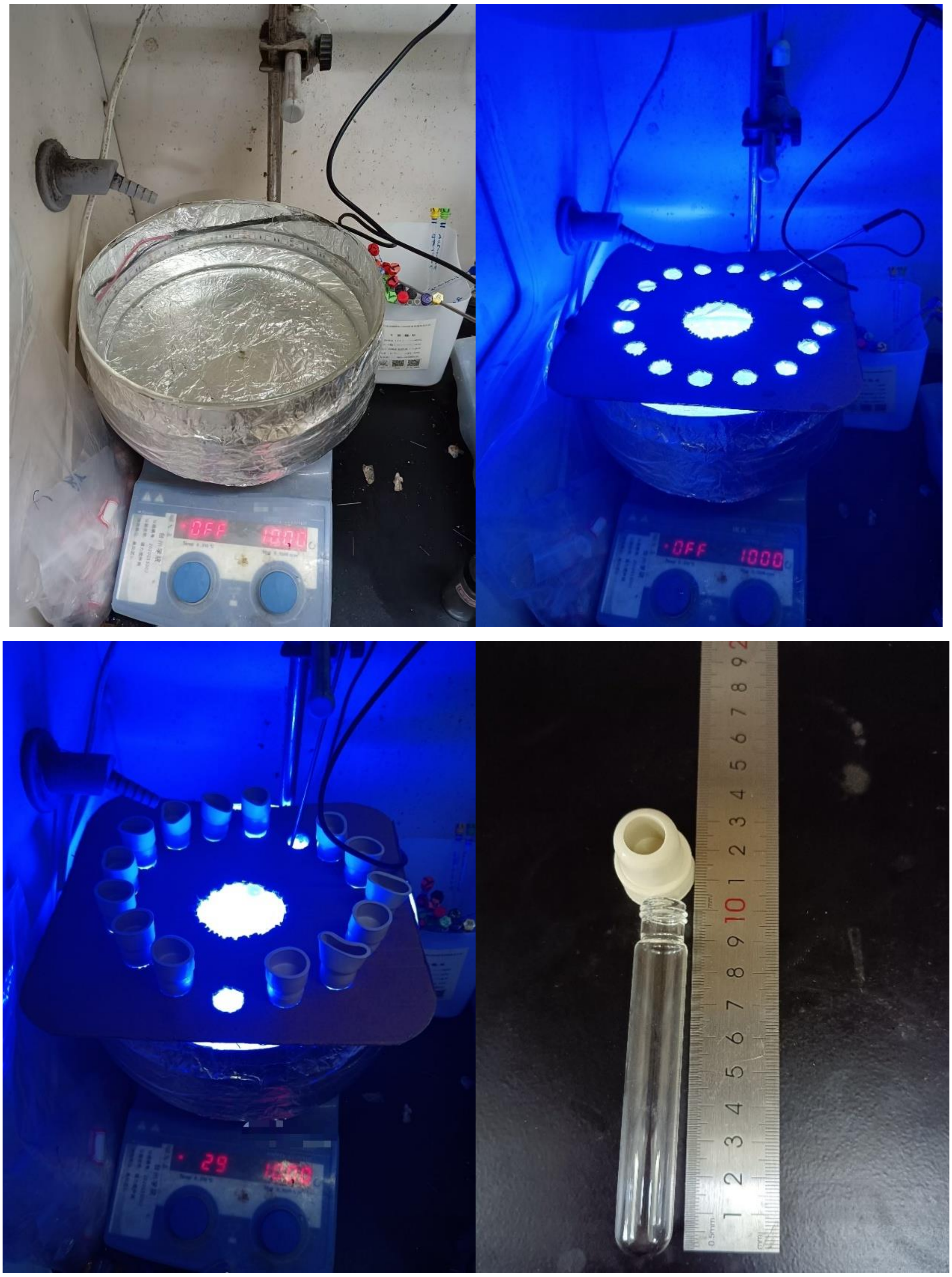

Supplementary Figure S1. Devices for the photocatalytic reactions

5. Mechanistic studies

*(a) The reactions of **1a** with different alkoxycarbonyl radical precursors (**2at** and **2au**).*

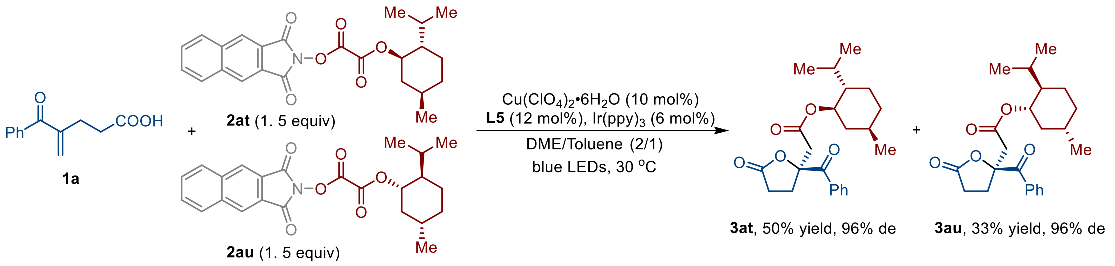

(b) Radical trapping experiment with TEMPO.

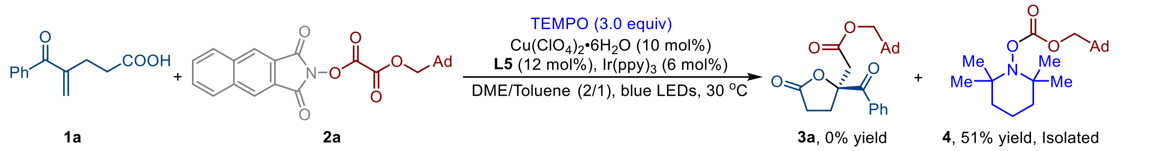

(c) Cyclic voltammetry studies

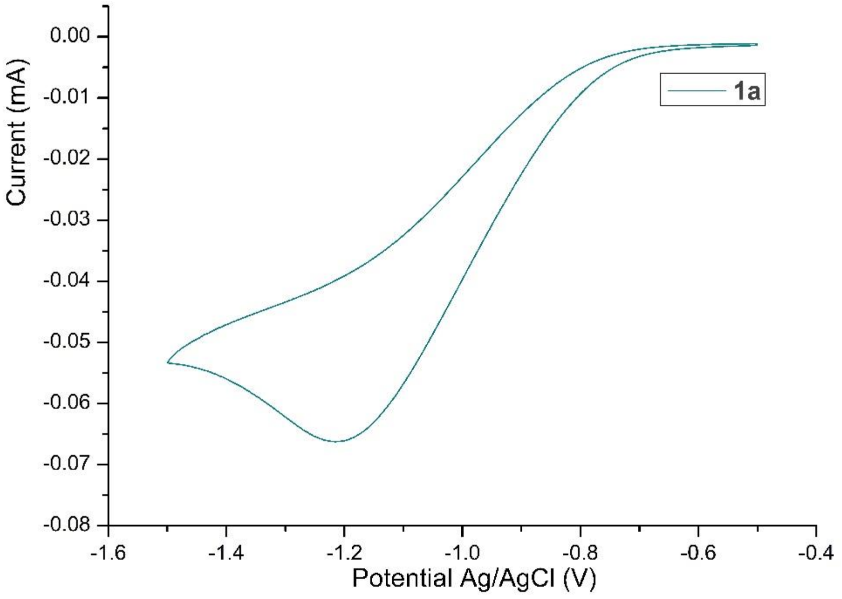

Supplementary Figure S2. Cyclic voltammogram of **1a** [0.02 M] in [0.1 M] $TBAPF_6$ in DME. Sweep rate: 100 mV/s. Glassy carbon working electrode, Ag/AgCl (satd. KCl) reference electrode, Pt wire auxiliary electrode. Irreversible reduction. E_p = -1.21 V.

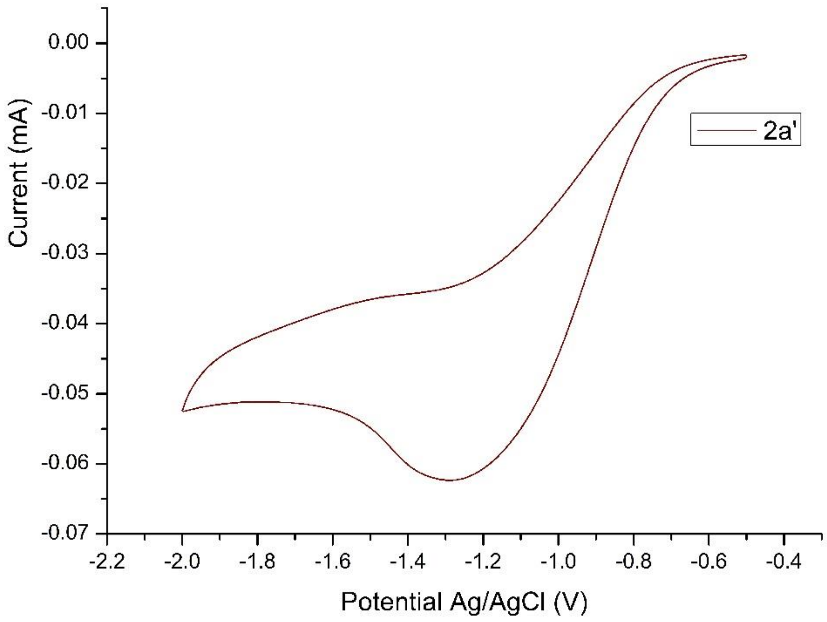

Supplementary Figure S3. Cyclic voltammogram of **2a'** [0.02 M] in [0.1 M] $TBAPF_6$ in DME. Sweep rate: 100 mV/s. Glassy carbon working electrode, Ag/AgCl (satd. KCl) reference electrode, Pt wire auxiliary electrode. Irreversible reduction. E_p = -1.29 V.

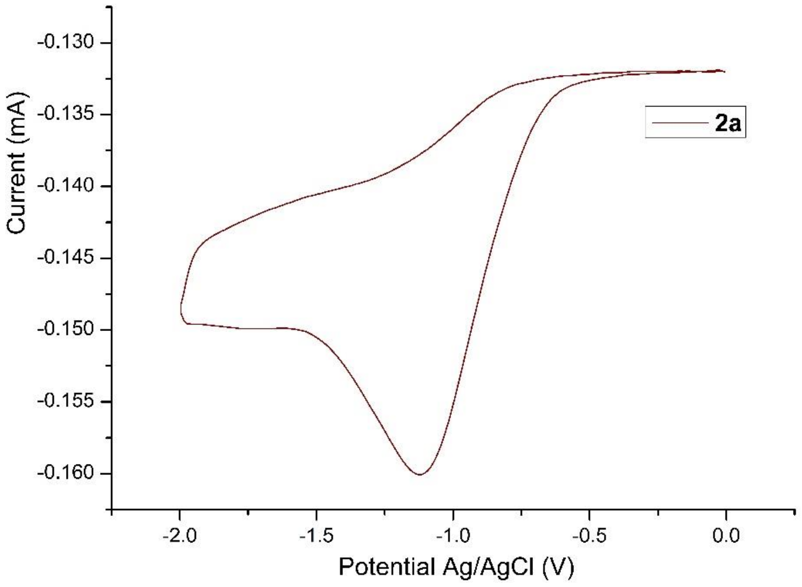

Supplementary Figure S4. Cyclic voltammogram of **2a** [0.02 M] in [0.1 M] $TBAPF_6$ in DME. Sweep rate: 63 mV/s. Glassy carbon working electrode, Ag/AgCl (satd. KCl) reference electrode, Pt wire auxiliary electrode. Irreversible reduction. E_p = -1.13 V;

(d) Stern-Volmer fluorescence quenching experiments

Stern-Volmer fluorescence quenching experiments were run with freshly prepared solutions of 0.1 mM Ir(ppy)$_3$ in degassed dry DME added with the appropriate amount of a quencher in a screw-top quartz cuvette at room temperature. The solutions were irradiated at 390 nm and fluorescence was measured from 450 nm to 700 nm.

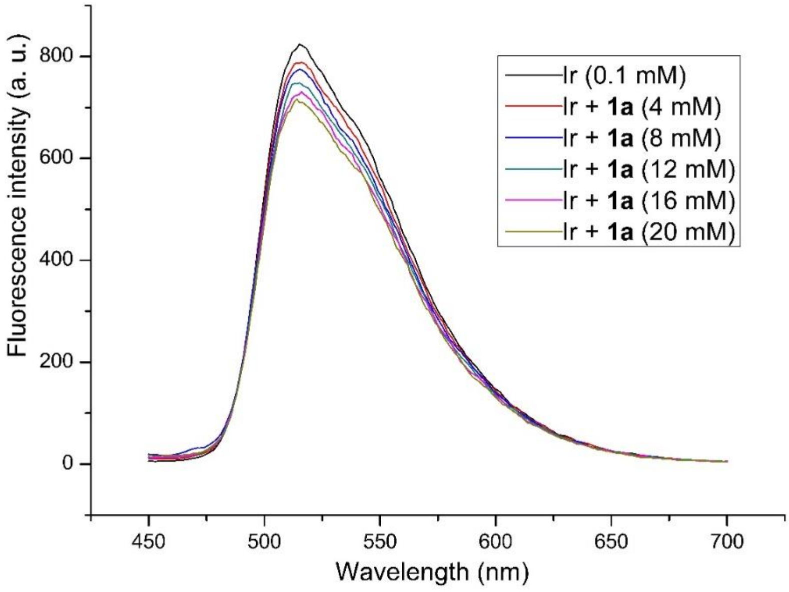

Supplementary Figure S5. Fluorescence quenching experiments of Ir(ppy)$_3$ and **1a**.

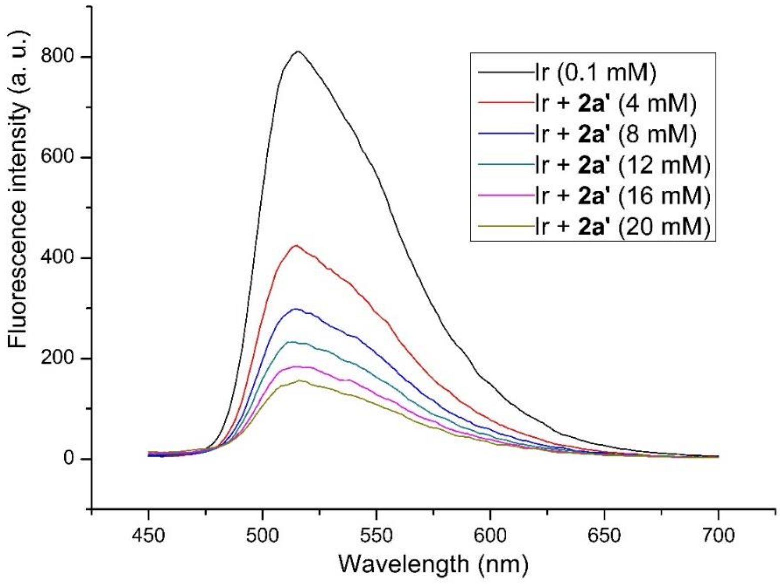

Supplementary Figure S6. Fluorescence quenching experiments of Ir(ppy)$_3$ and **2a'**.

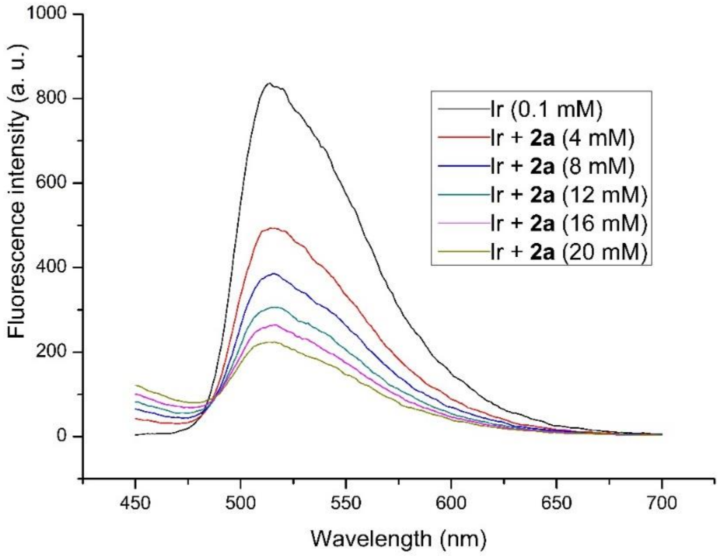

Supplementary Figure S7. Fluorescence quenching experiments of Ir(ppy)$_3$ and **2a**.

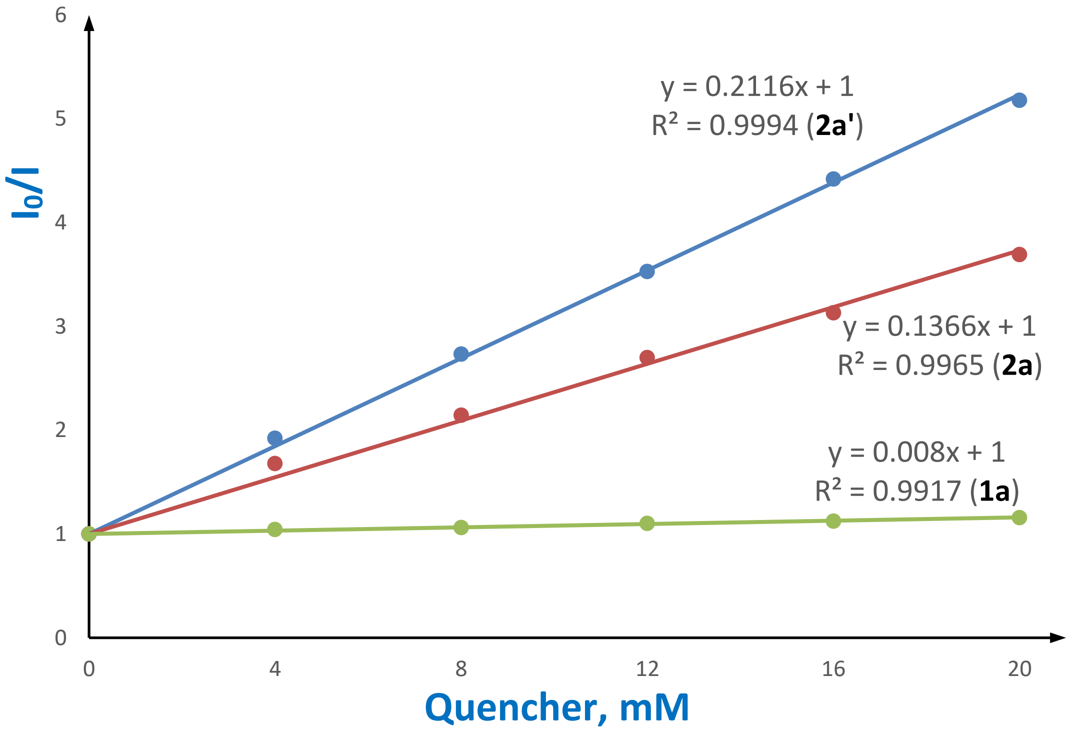

Supplementary Figure S8. Stern-Volmer plots of Ir(ppy)$_3$ with different quenchers.

6. X-ray data for compound 3a

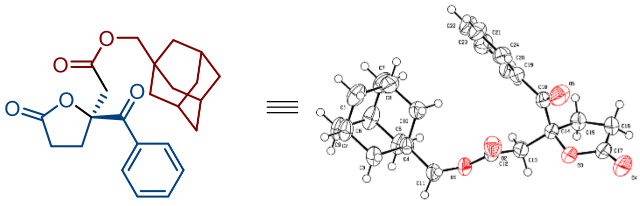

CCDC 2425222

Bond precision: C-C = 0.0031 A Wavelength= 1.54178

Cell: a= 6.1880(1) b= 11.6652(2) c= 14.1327(3)
alpha=90 beta=90.459(1) gamma=90

Temperature: 298 K

	Calculated	Reported
Volume	1020.13(3)	1020.13(3)
Space group	P 21	P 1 21 1
Hall group	P 2yb	P 2yb
Moiety formula	C24 H28 O5	C24 H28 O5
Sum formula	C24 H28 O5	C24 H28 O5
Mr	396.46	396.46
Dx,g cm-3	1.291	1.291
Z	2	2
Mu (mm-1)	0.725	0.725
F000	424.0	424.0
F000'	425.31	
h,k,lmax	7,14,17	7,14,17
Nref	3755[1978]	3555
Tmin,Tmax	0.853,0.865	0.458,0.753
Tmin'	0.853	

Correction method= # Reported T Limits: Tmin=0.458
Tmax=0.753 AbsCorr = MULTI-SCAN

Data completeness= 1.80/0.95 Theta(max)= 68.421

R(reflections)= 0.0321(3375) wR2(reflections)= 0.0809(3555)

S = 1.065 Npar= 262

7. Computational details

Gaussian 16 program was used for all the calculations.[4] The PBE0[5] density functional with D3BJ[6,7] dispersion correction and def2-SVP[8] basis sets were utilized for structures optimization and frequency calculations at 298.15 K and 1 atm. Frequency calculations were employed to confirm the minima (zero imaginary frequency) and transition state (only one imaginary frequency) at the same level of theory as above calculations. Intrinsic reaction coordinate (IRC)[9] calculations were performed for all the transition states. Further refining of single point energies with solvent effect was calculated at the PBE0-D3BJ and def2-TZVP[8,10] level of theory. The solvent effects were performed with SMD[11] model in DME and toluene mixed solvent.

Cartesian coordinates of related structures

L5$Cu^{I}ClO_4$

6	-2.76301	-2.87627	-2.05379
6	-2.33686	-1.84832	-1.21263
7	-1.05141	-1.54359	-1.10033
6	-0.11399	-2.14077	-1.82715
6	-0.44417	-3.18352	-2.69392
6	-1.78734	-3.5532	-2.78602
6	-3.16932	-0.85672	-0.50287
7	-4.48315	-1.00334	-0.11551
6	-5.04058	0.351677	0.039502
6	-3.73185	1.154421	0.289861
7	-2.68887	0.335691	-0.33195
6	1.171618	-1.41621	-1.69108
7	1.099957	-0.15368	-1.42049
6	2.455771	0.36506	-1.3253
6	3.326373	-0.70361	-2.04465
7	2.444857	-1.88039	-1.9193
29	-0.61551	0.340256	-0.32294
6	-3.46289	1.421241	1.753565
6	-2.59752	0.620086	2.497608
6	-2.37414	0.882626	3.848602
6	-3.03499	1.937384	4.47173
6	-3.9123	2.736952	3.736479
6	-4.11818	2.485837	2.383281
6	-5.82713	0.758886	-1.18391
6	-7.21598	0.598851	-1.19079
6	-7.96062	0.934744	-2.31898
6	-7.32381	1.428117	-3.45669
6	-5.93828	1.582465	-3.46143
6	-5.19355	1.251876	-2.33131
6	2.629299	1.744398	-1.89251
6	3.74115	2.505274	-1.5167
6	3.952329	3.764741	-2.07196
6	3.05012	4.280426	-3.00224
6	1.936965	3.529084	-3.37496
6	1.729296	2.264973	-2.8255
6	4.697221	-0.88813	-1.45851
6	4.859986	-1.24129	-0.11393
6	6.138131	-1.37721	0.421222
6	7.262292	-1.15469	-0.37444
6	7.103554	-0.8002	-1.71245
6	5.825201	-0.67348	-2.25186
1	-3.81888	-3.12969	-2.15075
1	0.313031	-3.68719	-3.29418
1	-2.07957	-4.35914	-3.46191
1	-5.69626	0.354383	0.919109
1	-3.78503	2.118946	-0.23614
1	2.710692	0.375678	-0.25088
1	3.420359	-0.447	-3.11401
1	-2.05371	-0.19613	2.022662
1	-1.6571	0.266424	4.395385
1	-2.85925	2.147039	5.529434
1	-4.42782	3.571426	4.217939
1	-4.79155	3.12695	1.806256
1	-7.70735	0.184383	-0.30786
1	-9.04577	0.808184	-2.30962
1	-7.90779	1.693372	-4.34121
1	-5.43252	1.967923	-4.3499
1	-4.10766	1.376525	-2.34848
1	4.443568	2.104379	-0.78118
1	4.823392	4.351085	-1.77006
1	3.212632	5.271461	-3.43262
1	1.223081	3.930543	-4.09847
1	0.85531	1.673266	-3.10728
1	3.985812	-1.40275	0.521543
1	6.254887	-1.65471	1.471389
1	8.263764	-1.25893	0.050014
1	7.97905	-0.62816	-2.34292
1	5.697772	-0.40961	-3.30457
6	2.899613	-3.17688	-2.17258
6	-5.17504	-2.12941	0.32277
8	3.842266	-3.35708	-2.90899
8	-6.37733	-2.08974	0.459626
6	2.211842	-4.28019	-1.44745
6	1.635942	-4.09526	-0.18426
6	2.214698	-5.54951	-2.03828
6	1.045542	-5.17666	0.464949
1	1.660115	-3.1224	0.313752
6	1.602227	-6.61849	-1.39443
1	2.705045	-5.67507	-3.00628
6	1.014385	-6.43101	-0.14125
1	0.614009	-5.02937	1.456276
1	1.591558	-7.6053	-1.86301
1	0.541253	-7.27334	0.369755
6	-4.35591	-3.32827	0.654508
6	-4.85257	-4.59322	0.32306
6	-3.14098	-3.21375	1.339754

6	-4.10723	-5.72972	0.620759
1	-5.8212	-4.66797	-0.17635
6	-2.40969	-4.35384	1.656525
1	-2.75012	-2.2415	1.643832
6	-2.88303	-5.61011	1.282695
1	-4.48455	-6.7164	0.342304
1	-1.46607	-4.23665	2.191482
1	-2.30062	-6.50486	1.515904
17	0.769585	-0.7789	2.121487
8	0.393509	0.536788	1.480408
8	0.950567	-0.55996	3.545685
8	-0.31075	-1.75433	1.865412
8	2.014594	-1.26064	1.49489

L5Cu^{II}ClO$_4$

6	-2.60283	-2.89577	-2.05247
6	-2.18353	-1.84644	-1.238
7	-0.90216	-1.48532	-1.22358
6	0.0443	-2.096	-1.93445
6	-0.29533	-3.16433	-2.76278
6	-1.63541	-3.54992	-2.81473
6	-2.99093	-0.90117	-0.42252
7	-4.27441	-1.00991	0.012058
6	-4.73256	0.356857	0.381049
6	-3.36424	1.062415	0.616836
7	-2.43746	0.241887	-0.15542
6	1.3355	-1.36709	-1.79628
7	1.248655	-0.14227	-1.3792
6	2.549757	0.520303	-1.50023
6	3.52724	-0.64131	-1.8145
7	2.586729	-1.76008	-2.1242
29	-0.47175	0.318428	-0.48915
6	-2.95515	1.15543	2.066516
6	-2.30209	0.103706	2.712735
6	-1.98512	0.194524	4.065367
6	-2.32164	1.338432	4.78406
6	-2.96824	2.394816	4.143928
6	-3.2803	2.30543	2.79106
6	-5.58689	0.959909	-0.70514
6	-6.97664	0.975981	-0.5585
6	-7.78449	1.513077	-1.55795
6	-7.21117	2.032786	-2.71697
6	-5.8255	2.016507	-2.87348
6	-5.01688	1.485583	-1.87204
6	2.449852	1.602833	-2.54762
6	2.324438	2.93549	-2.14701
6	2.186769	3.942215	-3.10101
6	2.169504	3.62381	-4.45766
6	2.287388	2.293743	-4.86225
6	2.424202	1.286833	-3.91066
6	4.414555	-1.0489	-0.66543
6	3.925658	-1.05854	0.648578
6	4.738428	-1.49346	1.69205
6	6.041844	-1.9192	1.435661
6	6.532571	-1.90799	0.131722
6	5.721062	-1.47617	-0.91565
1	-3.64929	-3.19221	-2.09988
1	0.453269	-3.6759	-3.36496
1	-1.93279	-4.36963	-3.47166
1	-5.30849	0.277815	1.311178
1	-3.38776	2.073888	0.187789
1	2.803392	0.984973	-0.53791
1	4.131028	-0.43404	-2.70758
1	-1.99717	-0.78297	2.15265
1	-1.45817	-0.62648	4.555853
1	-2.06661	1.414552	5.843126
1	-3.21963	3.300647	4.69959
1	-3.77498	3.141435	2.28922
1	-7.42679	0.549638	0.340407
1	-8.86901	1.525358	-1.42969
1	-7.84488	2.456514	-3.49925
1	-5.3712	2.429319	-3.77696
1	-3.93161	1.492125	-2.00565
1	2.330453	3.180241	-1.08215
1	2.097506	4.982653	-2.78092
1	2.067788	4.414574	-5.20436
1	2.278444	2.041436	-5.92501
1	2.518358	0.247504	-4.23992
1	2.915621	-0.70533	0.876897
1	4.354368	-1.48594	2.714512
1	6.679649	-2.253	2.257195
1	7.554376	-2.23461	-0.07309
1	6.101351	-1.47728	-1.93998
6	3.09977	-3.03253	-2.47823
6	-5.08315	-2.13033	0.281479
8	4.045776	-3.09885	-3.21864
8	-6.26451	-1.97842	0.454983
6	2.46527	-4.2104	-1.8368

6	1.940893	-4.14213	-0.5391
6	2.470672	-5.42801	-2.52838
6	1.392637	-5.28021	0.045353
1	2.003495	-3.21209	0.031938
6	1.898048	-6.55521	-1.94944
1	2.92678	-5.47088	-3.52014
6	1.354812	-6.48013	-0.66467
1	1.010546	-5.23482	1.067472
1	1.888489	-7.50236	-2.49304
1	0.920116	-7.37143	-0.20608
6	-4.40324	-3.4488	0.388368
6	-5.05587	-4.57047	-0.13589
6	-3.17542	-3.59817	1.045313
6	-4.44778	-5.82005	-0.06726
1	-6.03615	-4.4453	-0.60142
6	-2.58236	-4.85429	1.132845
1	-2.69678	-2.7382	1.519299
6	-3.20788	-5.96097	0.559712
1	-4.94836	-6.69278	-0.49214
1	-1.63345	-4.97137	1.660112
1	-2.73829	-6.94538	0.621244
17	0.775951	1.716474	1.571864
8	-0.06333	2.067184	0.312618
8	0.164799	2.331424	2.7201
8	0.712547	0.224881	1.620043
8	2.138435	2.167285	1.346696

1a

6	0.616979	2.778249	0.843059
6	0.160616	4.035884	0.752394
1	-0.88897	4.244331	0.53469
1	0.822132	4.898713	0.861707
6	2.050841	2.39831	1.069241
1	2.103422	1.769535	1.976175
1	2.3485	1.727031	0.248895
6	3.022679	3.560212	1.185884
1	2.767021	4.234803	2.014589
1	3.001201	4.156001	0.256499
6	4.450643	3.136347	1.402027
8	5.194238	3.595383	2.22778
8	4.831693	2.179636	0.542818
1	5.757234	1.978028	0.750021
6	-0.28654	1.612198	0.564664
8	0.170146	0.617889	0.035677
6	-1.7379	1.676102	0.919187
6	-2.61763	0.865285	0.189161
6	-2.23316	2.436373	1.986573
6	-3.97287	0.843125	0.495302
1	-2.20772	0.255457	-0.61862
6	-3.58935	2.398715	2.304057
1	-1.55092	3.042586	2.585182
6	-4.46122	1.611493	1.554013
1	-4.65431	0.219812	-0.08839
1	-3.96654	2.985023	3.145139
1	-5.52575	1.589723	1.799801

7

6	-3.75025	-3.65269	1.215632
6	-2.6393	-2.83123	1.395382
7	-2.77703	-1.50712	1.390123
6	-3.94698	-0.8931	1.221056
6	-5.10489	-1.64525	1.028275
6	-4.98679	-3.03627	1.027105
6	-1.24882	-3.20658	1.761281
7	-0.64609	-4.43367	1.729537
6	0.490564	-4.35517	2.692692
6	0.786627	-2.83733	2.629384
7	-0.5238	-2.28495	2.30398
6	-3.78467	0.575207	1.423119
7	-2.73818	0.940308	2.090775
6	-2.69504	2.390562	2.171645
6	-4.13629	2.820304	1.776911
7	-4.60235	1.601592	1.064678
29	-1.27814	-0.43684	2.199435
6	1.844996	-2.42667	1.63064
6	1.545521	-1.81424	0.413609
6	2.566655	-1.44164	-0.46125
6	3.895383	-1.6848	-0.12736
6	4.202385	-2.29059	1.092813
6	3.184943	-2.6516	1.969139
6	0.061213	-4.86321	4.059565
6	0.194276	-4.10086	5.224618
6	-0.23543	-4.60619	6.452728
6	-0.79255	-5.87816	6.535147
6	-0.90934	-6.65302	5.380347
6	-0.48533	-6.15173	4.15414
6	-2.2293	2.918955	3.499297
6	-1.68716	4.206213	3.565957

6	-1.28053	4.732831	4.789849	1	2.11941	3.429148	5.145602
6	-1.4011	3.972264	5.952758	1	1.158391	2.48088	6.424645
6	-1.92899	2.683805	5.889131	1	3.686236	1.94455	3.630336
6	-2.3479	2.163322	4.667338	1	3.111877	0.296568	3.329148
6	-4.19998	4.061842	0.935256	1	1.626854	2.893725	2.581331
6	-3.53378	4.123412	-0.29417	1	2.517358	1.873652	1.447907
6	-3.57977	5.283816	-1.06082	6	-5.80207	1.608389	0.319676
6	-4.28461	6.396662	-0.6004	6	-0.78463	-5.47018	0.796147
6	-4.94675	6.341464	0.623885	8	-6.68836	2.361918	0.629042
6	-4.90925	5.175307	1.386069	8	-0.27392	-6.54399	0.99662
1	-3.65813	-4.73762	1.235288	6	-5.85469	0.706992	-0.85979
1	-6.07619	-1.17137	0.896592	6	-4.70359	0.319808	-1.55888
1	-5.87991	-3.65002	0.89445	6	-7.11427	0.282969	-1.30133
1	1.313287	-4.96119	2.288971	6	-4.81211	-0.515	-2.66728
1	1.105312	-2.44216	3.601054	1	-3.7218	0.691651	-1.25632
1	-1.98477	2.725562	1.394668	6	-7.21653	-0.56455	-2.39897
1	-4.75471	2.949626	2.679993	1	-8.00204	0.629347	-0.76737
1	0.5124	-1.57354	0.157021	6	-6.06522	-0.96846	-3.0786
1	2.316818	-0.94715	-1.40233	1	-3.91586	-0.80263	-3.22094
1	4.695706	-1.39421	-0.81146	1	-8.19831	-0.90389	-2.73611
1	5.243836	-2.47191	1.367678	1	-6.14892	-1.62611	-3.94696
1	3.430026	-3.09677	2.937002	6	-1.56222	-5.1588	-0.43566
1	0.649777	-3.10934	5.2137	6	-2.44524	-6.12942	-0.9215
1	-0.12685	-3.98935	7.347843	6	-1.39092	-3.95746	-1.13494
1	-1.12595	-6.27231	7.497903	6	-3.20551	-5.86834	-2.05725
1	-1.32686	-7.66105	5.436203	1	-2.5309	-7.08187	-0.39341
1	-0.55962	-6.76806	3.255531	6	-2.1358	-3.71115	-2.28509
1	-1.58449	4.798983	2.652789	1	-0.64816	-3.22865	-0.80242
1	-0.86291	5.74124	4.834533	6	-3.05698	-4.65631	-2.73476
1	-1.0769	4.38375	6.911516	1	-3.90812	-6.61879	-2.42604
1	-2.00453	2.072133	6.791176	1	-1.98494	-2.78256	-2.83988
1	-2.75068	1.149362	4.621012	1	-3.64722	-4.45932	-3.63271
1	-2.97207	3.259107	-0.66084	6	1.564933	0.003763	5.352336
1	-3.06023	5.322869	-2.02074	8	1.985168	-1.03295	4.868958
1	-4.31802	7.308787	-1.20034	6	0.511772	-0.07729	6.416205
1	-5.50305	7.20869	0.985978	6	0.708858	0.375725	7.724453
1	-5.44085	5.12582	2.339278	6	-0.69149	-0.71103	6.084861
6	2.038017	1.329034	4.85872	6	-0.29113	0.20717	8.682006
6	2.782329	1.322631	3.546287	1	1.657208	0.839038	8.005763
6	1.761015	2.464352	5.516924	6	-1.69115	-0.87183	7.039142
6	1.939503	1.853139	2.383095	1	-0.83452	-1.06949	5.064047
6	0.676566	1.076611	2.116793	6	-1.49415	-0.40953	8.341292
8	0.145883	1.021869	0.996302	1	-0.12433	0.552736	9.704792
8	0.144517	0.465993	3.114605	1	-2.62683	-1.36776	6.769589

1	-2.27484	-0.53969	9.094216

7-iso

6	-3.95834	-3.44128	1.281551
6	-2.83199	-2.64621	1.487543
7	-2.9623	-1.33811	1.684192
6	-4.13425	-0.71289	1.616421
6	-5.31083	-1.43585	1.43159
6	-5.2051	-2.81671	1.273878
6	-1.42352	-3.06982	1.666894
7	-0.94627	-4.34791	1.574027
6	0.262768	-4.42206	2.415977
6	0.655109	-2.9173	2.483882
7	-0.58582	-2.21173	2.146743
6	-3.94241	0.741809	1.846623
7	-2.88756	1.075899	2.524454
6	-2.89695	2.512545	2.74719
6	-4.00456	3.041489	1.797431
7	-4.70825	1.779127	1.436749
29	-1.37667	-0.28071	2.392855
6	1.839786	-2.53843	1.624228
6	1.834894	-1.39865	0.82115
6	2.981474	-1.02879	0.116016
6	4.132982	-1.80607	0.192253
6	4.142273	-2.95108	0.991615
6	3.006533	-3.30987	1.709949
6	-0.01921	-5.06073	3.750898
6	0.609143	-6.25804	4.095251
6	0.372134	-6.85063	5.33491
6	-0.50872	-6.2557	6.235622
6	-1.14739	-5.06216	5.895058
6	-0.89884	-4.46484	4.663122
6	-3.10729	2.870214	4.200794
6	-2.97435	4.211606	4.578018
6	-3.15338	4.591034	5.904419
6	-3.46007	3.631485	6.870537
6	-3.58768	2.295437	6.50039
6	-3.41346	1.914705	5.169694
6	-3.47782	3.688699	0.540346
6	-2.32566	3.188273	-0.08546
6	-1.86409	3.764987	-1.26583
6	-2.54827	4.840631	-1.83309
6	-3.69449	5.338761	-1.21671
6	-4.15883	4.764926	-0.034
1	-3.87325	-4.51744	1.143471
1	-6.2818	-0.94428	1.413606
1	-6.10815	-3.4152	1.139822
1	1.013182	-5.01178	1.876791
1	0.890174	-2.66219	3.527458
1	-1.92622	2.916722	2.427129
1	-4.70971	3.702228	2.317351
1	0.958129	-0.75127	0.770186
1	2.95733	-0.10512	-0.46477
1	5.029409	-1.5181	-0.36171
1	5.044286	-3.5635	1.063407
1	3.039507	-4.18793	2.36084
1	1.281405	-6.73771	3.379499
1	0.870564	-7.78762	5.592976
1	-0.70173	-6.72337	7.203802
1	-1.83679	-4.59064	6.599532
1	-1.37841	-3.51147	4.424549
1	-2.72288	4.965912	3.826228
1	-3.04834	5.640599	6.188153
1	-3.59743	3.928161	7.912643
1	-3.82385	1.539714	7.252975
1	-3.49768	0.863915	4.886678
1	-1.76217	2.356393	0.351246
1	-0.9557	3.377869	-1.73298
1	-2.18176	5.296485	-2.75562
1	-4.23056	6.182773	-1.65622
1	-5.06255	5.14949	0.445053
6	-5.8407	1.837975	0.584132
6	-1.26014	-5.3843	0.683823
8	-6.63968	2.724609	0.733993
8	-0.88463	-6.50621	0.914041
6	-5.91673	0.829478	-0.5009
6	-4.76297	0.380237	-1.15637
6	-7.1773	0.378575	-0.90924
6	-4.87074	-0.55118	-2.1842
1	-3.78429	0.78495	-0.88504
6	-7.27693	-0.57201	-1.91978
1	-8.06795	0.775988	-0.41708
6	-6.12336	-1.04273	-2.55113
1	-3.97393	-0.89196	-2.70423
1	-8.25811	-0.94028	-2.22706
1	-6.20462	-1.78453	-3.34905
6	-1.98454	-5.00082	-0.55944
6	-2.8998	-5.9093	-1.1035

6	-1.69977	-3.80577	-1.23341
6	-3.57225	-5.59581	-2.27999
1	-3.0719	-6.85837	-0.59055
6	-2.35384	-3.51112	-2.42696
1	-0.93341	-3.12309	-0.85613
6	-3.30163	-4.39545	-2.94061
1	-4.29756	-6.29809	-2.69655
1	-2.10309	-2.59683	-2.96946
1	-3.81564	-4.16154	-3.87599
6	1.149995	1.356297	4.864711
6	0.300861	2.373882	4.158065
6	2.207663	1.70114	5.620949
6	0.854145	2.809373	2.80487
6	0.856735	1.780976	1.678886
8	1.722009	1.821075	0.828167
8	-0.14558	0.952007	1.629782
1	2.48704	2.752291	5.739481
1	2.803616	0.963175	6.161237
1	0.195021	3.259308	4.802003
1	-0.7055	1.954396	4.046169
1	1.881353	3.189841	2.900345
1	0.254392	3.658242	2.428892
6	0.726609	-0.06201	4.802193
8	-0.41313	-0.37292	4.448682
6	1.677072	-1.13775	5.189565
6	2.975498	-1.17848	4.661619
6	1.215963	-2.19596	5.985127
6	3.796737	-2.27139	4.927502
1	3.322767	-0.38046	4.002067
6	2.047255	-3.27413	6.264336
1	0.196366	-2.16532	6.373186
6	3.337144	-3.31404	5.73091
1	4.79611	-2.31297	4.489799
1	1.681613	-4.09719	6.881822
1	3.985275	-4.1684	5.939322

TS1

6	-2.01269	-3.3241	-0.64724
6	-1.432	-2.07678	-0.42063
7	-0.12159	-1.91699	-0.59106
6	0.705645	-2.89853	-0.94951
6	0.208968	-4.19155	-1.11007
6	-1.16598	-4.38344	-0.97073
6	-2.07499	-0.77832	-0.06975
7	-3.38503	-0.50811	0.198898
6	-3.58618	0.945056	-0.05085
6	-2.13142	1.462739	0.133763
7	-1.32982	0.279082	-0.15219
6	2.100251	-2.38138	-1.07265
7	2.271492	-1.12401	-0.82128
6	3.68971	-0.80736	-0.85267
6	4.352595	-2.20005	-0.70067
7	3.272109	-3.06221	-1.25909
29	0.635465	-0.08787	-0.29813
6	-1.86926	2.035928	1.507667
6	-1.33761	1.268628	2.546441
6	-1.18662	1.817057	3.819341
6	-1.56862	3.134917	4.062536
6	-2.08081	3.911981	3.023171
6	-2.22606	3.365974	1.75186
6	-4.19251	1.188215	-1.41049
6	-5.57025	1.399845	-1.51834
6	-6.1568	1.607317	-2.76431
6	-5.37318	1.599511	-3.91682
6	-3.99897	1.385063	-3.81905
6	-3.41078	1.182976	-2.57302
6	4.083843	-0.05608	-2.10529
6	5.392412	0.429075	-2.21097
6	5.8026	1.109761	-3.35344
6	4.905814	1.323881	-4.40168
6	3.598806	0.855182	-4.29718
6	3.190135	0.16719	-3.15404
6	4.685055	-2.59543	0.718588
6	3.993646	-2.06019	1.814325
6	4.309606	-2.47442	3.107255
6	5.312769	-3.41799	3.319988
6	6.003539	-3.95141	2.23244
6	5.690419	-3.54405	0.938663
1	-3.08572	-3.47828	-0.54974
1	0.86143	-5.02371	-1.36745
1	-1.58599	-5.37957	-1.12259
1	-4.25272	1.330194	0.730167
1	-1.90614	2.232456	-0.61809
1	3.925546	-0.1859	0.023427
1	5.23352	-2.31433	-1.34413
1	-0.9917	0.250062	2.360037
1	-0.75613	1.212054	4.620147
1	-1.44723	3.565578	5.058915

1	-2.3372	4.959678	3.188795
1	-2.59636	3.993336	0.937224
1	-6.18644	1.384905	-0.61706
1	-7.23327	1.778041	-2.83448
1	-5.83291	1.767055	-4.8934
1	-3.3794	1.387015	-4.71872
1	-2.33008	1.02888	-2.51312
1	6.097788	0.274376	-1.38934
1	6.827307	1.480993	-3.42467
1	5.227248	1.860173	-5.29708
1	2.889372	1.026585	-5.10995
1	2.159139	-0.18448	-3.07322
1	3.212016	-1.30498	1.687671
1	3.770819	-2.04272	3.953784
1	5.562678	-3.73348	4.335625
1	6.794763	-4.68731	2.39214
1	6.215885	-3.9755	0.084222
6	3.594326	-4.20837	-2.00069
6	-4.40671	-1.26844	0.794103
8	-5.54828	-0.89074	0.714866
8	4.659514	-4.74838	-1.83509
6	-4.0032	-2.47912	1.556342
6	-4.85878	-3.58708	1.527576
6	-2.84643	-2.50902	2.345477
6	-4.52223	-4.74	2.229307
1	-5.78168	-3.52845	0.946195
6	-2.5253	-3.65744	3.063886
1	-2.21358	-1.62262	2.430508
6	-3.35273	-4.7778	2.991938
1	-5.18107	-5.61038	2.194671
1	-1.63213	-3.67388	3.692058
1	-3.09668	-5.68047	3.551441
6	2.612521	-4.6545	-3.02394
6	1.895488	-3.74157	-3.80767
6	2.470886	-6.02983	-3.24474
6	1.00761	-4.20552	-4.77461
1	2.058232	-2.66709	-3.69002
6	1.565628	-6.48828	-4.19635
1	3.074727	-6.72516	-2.6574
6	0.829338	-5.5768	-4.95644
1	0.461413	-3.49502	-5.39877
1	1.441922	-7.56129	-4.3581
1	0.127673	-5.93912	-5.71128
6	1.896498	5.305343	2.008452
6	3.21215	5.638488	1.990574
1	3.532617	6.67328	2.119647
1	3.995678	4.877905	1.986221
6	1.37465	3.928426	1.739009
1	0.550504	3.713697	2.436406
1	0.885493	3.965943	0.751029
6	2.381205	2.786307	1.759609
1	2.710599	2.538136	2.777663
1	3.264853	3.051717	1.157696
6	1.782446	1.562839	1.127919
8	1.688057	0.467197	1.7081
8	1.345529	1.693916	-0.07786
8	3.824585	4.45994	-0.69441
8	2.713711	6.4037	-1.0438
6	3.412682	5.674632	-0.42385
6	0.833588	6.350668	2.008021
8	-0.2533	6.097848	1.513374
6	1.092523	7.697913	2.59481
6	1.884435	7.890102	3.734531
6	0.441435	8.7963	2.018159
6	2.034012	9.164405	4.277234
1	2.364601	7.034618	4.214145
6	0.608526	10.06998	2.548841
1	-0.19152	8.625931	1.145025
6	1.40566	10.25589	3.679789
1	2.642117	9.305817	5.173642
1	0.112725	10.92462	2.082891
1	1.532148	11.25614	4.10084
6	3.31295	3.819448	-1.88252
1	4.115984	3.179432	-2.26579
1	2.44829	3.203472	-1.59812
1	3.0297	4.578854	-2.62281

8

6	-1.91827	-3.48102	-1.79232
6	-1.75841	-2.13036	-1.49112
7	-0.53879	-1.60632	-1.39435
6	0.575745	-2.31528	-1.55036
6	0.50075	-3.67767	-1.83917
6	-0.76558	-4.24837	-1.96233
6	-2.78875	-1.06471	-1.38392
7	-4.14299	-1.18433	-1.24928
6	-4.73891	0.105249	-1.68319
6	-3.52226	1.056521	-1.4864

7	-2.38098	0.152629	-1.55133	1	-8.20816	-0.41317	-4.75774
6	1.762164	-1.41739	-1.53613	1	-6.67152	-0.27261	-6.71198
7	1.535466	-0.17971	-1.83008	1	-4.23065	0.054531	-6.34609
6	2.793631	0.551911	-1.91835	1	-3.33149	0.249253	-4.05658
6	3.887232	-0.50755	-1.57504	1	3.107311	3.201696	-2.47563
7	3.069923	-1.72669	-1.33498	1	3.423775	4.261763	-4.66001
29	-0.3649	0.393355	-1.40781	1	3.416744	2.87343	-6.74887
6	-3.56841	1.838164	-0.19378	1	3.106349	0.409354	-6.58725
6	-3.0535	1.324394	0.997869	1	2.814608	-0.66513	-4.37431
6	-3.1775	2.040739	2.187058	1	2.990564	0.348407	0.866326
6	-3.8212	3.276286	2.195816	1	4.339369	0.891435	2.857885
6	-4.32981	3.800084	1.006537	1	6.824054	0.75587	2.76754
6	-4.20034	3.086152	-0.18162	1	7.95067	0.030757	0.669317
6	-5.26418	0.019771	-3.0947	1	6.592071	-0.56596	-1.32672
6	-6.63263	-0.16826	-3.30862	6	1.870098	5.107751	-0.06426
6	-7.13629	-0.27127	-4.60312	6	0.410331	4.805813	0.006158
6	-6.27592	-0.19427	-5.69682	6	2.23386	6.465126	-0.584
6	-4.90866	-0.01197	-5.49225	6	-0.05974	3.598817	0.806947
6	-4.40496	0.097232	-4.19856	6	-0.04696	2.292063	0.065342
6	2.95264	1.195026	-3.27175	8	-0.24306	2.318935	-1.20402
6	3.11473	2.577311	-3.36961	8	0.037425	1.199728	0.660112
6	3.285957	3.179692	-4.61503	1	-0.08562	5.715046	0.390407
6	3.282976	2.403557	-5.7716	1	0.03582	4.698464	-1.03163
6	3.110076	1.020688	-5.68175	1	1.378585	6.862033	-1.15807
6	2.94729	0.41964	-4.43704	1	2.40999	7.194039	0.224422
6	4.708052	-0.16182	-0.36114	1	-1.11861	3.752755	1.079336
6	4.078047	0.238884	0.823704	1	0.489126	3.465446	1.748256
6	4.837553	0.562234	1.943655	6	3.668983	-2.9048	-0.84838
6	6.229528	0.491553	1.890168	6	-4.94515	-2.17554	-0.66476
6	6.860966	0.091819	0.714104	8	4.788082	-3.19136	-1.18976
6	6.101292	-0.23802	-0.40719	8	-6.13777	-2.15513	-0.84006
1	-2.90664	-3.92452	-1.89994	6	2.882521	-3.6925	0.137727
1	1.398559	-4.27668	-1.98264	6	2.021048	-3.07315	1.052745
1	-0.85668	-5.30804	-2.20863	6	3.07906	-5.07734	0.189566
1	-5.55775	0.345821	-0.99415	6	1.337133	-3.8428	1.989459
1	-3.46094	1.76451	-2.3247	1	1.9086	-1.98615	1.059425
1	2.776794	1.345473	-1.15622	6	2.372994	-5.84456	1.109905
1	4.546924	-0.69064	-2.4351	1	3.790086	-5.53525	-0.50194
1	-2.4999	0.384163	0.998141	6	1.498655	-5.22778	2.007582
1	-2.75499	1.634031	3.108197	1	0.687864	-3.35462	2.719338
1	-3.92022	3.836736	3.128102	1	2.515138	-6.927	1.140703
1	-4.82711	4.772605	1.003753	1	0.956859	-5.83094	2.740017
1	-4.59502	3.502428	-1.11264	6	-4.2687	-3.18057	0.199087
1	-7.30262	-0.2519	-2.4504	6	-4.72398	-4.50304	0.149503

6	-3.25083	-2.8255	1.092565
6	-4.1162	-5.47523	0.937567
1	-5.5514	-4.75418	-0.51796
6	-2.66292	-3.79696	1.897431
1	-2.93554	-1.78363	1.18361
6	-3.08115	-5.12389	1.806489
1	-4.45901	-6.51086	0.885238
1	-1.88081	-3.51468	2.604666
1	-2.61242	-5.88745	2.431522
6	2.869834	4.090528	0.204201
8	2.54398	2.899059	0.175563
6	4.294628	4.450866	0.458506
6	5.292794	3.589593	-0.01529
6	4.660582	5.583439	1.194955
6	6.633145	3.875899	0.212705
1	5.004427	2.693316	-0.56626
6	6.004056	5.861293	1.437442
1	3.893429	6.2419	1.607389
6	6.991686	5.01431	0.937395
1	7.402383	3.20201	-0.17032
1	6.279827	6.741027	2.023022
1	8.045499	5.236795	1.121246
6	3.413448	6.463879	-1.53097
8	3.605388	5.620459	-2.36983
8	4.193868	7.520392	-1.34708
6	5.358804	7.584942	-2.15898
1	5.092659	7.587244	-3.22536
1	5.867765	8.516593	-1.88927
1	6.008086	6.721022	-1.95495

8-iso

6	-3.69589	-3.42755	0.547982
6	-2.7289	-2.71749	1.257817
7	-3.00144	-1.49989	1.71033
6	-4.1646	-0.89098	1.518534
6	-5.18753	-1.53455	0.823042
6	-4.93259	-2.815	0.337254
6	-1.41251	-3.17502	1.760786
7	-0.84534	-4.40498	1.562695
6	0.030677	-4.65337	2.710085
6	0.371075	-3.18629	3.11467
7	-0.80046	-2.44176	2.639685
6	-4.17249	0.407092	2.23798
7	-3.44445	0.459132	3.309563
6	-3.46681	1.810592	3.830617
6	-4.56131	2.545973	2.988186
7	-4.81575	1.554049	1.918984
29	-1.68857	-0.5709	2.874374
6	1.680243	-2.72523	2.517691
6	1.747702	-2.02224	1.313915
6	2.984127	-1.70526	0.749012
6	4.164671	-2.08453	1.380694
6	4.104539	-2.79008	2.584674
6	2.870283	-3.1099	3.147009
6	-0.64501	-5.40494	3.830839
6	0.149405	-5.8729	4.883226
6	-0.42955	-6.49841	5.982907
6	-1.81317	-6.66724	6.042249
6	-2.60862	-6.213	4.993326
6	-2.02705	-5.58489	3.892358
6	-3.68109	1.88406	5.317759
6	-4.17015	0.793702	6.040069
6	-4.37982	0.898598	7.414766
6	-4.10901	2.095155	8.075374
6	-3.62312	3.187755	7.356908
6	-3.40547	3.081725	5.985845
6	-4.11006	3.867125	2.43066
6	-2.96627	3.926905	1.623191
6	-2.5436	5.147995	1.105867
6	-3.2499	6.315957	1.39728
6	-4.38516	6.258433	2.202797
6	-4.81801	5.03526	2.712642
1	-3.50082	-4.42968	0.169144
1	-6.1531	-1.05478	0.666546
1	-5.71184	-3.35102	-0.20766
1	0.92048	-5.19869	2.370172
1	0.416047	-3.11013	4.209038
1	-2.48221	2.255655	3.60335
1	-5.48145	2.675003	3.578525
1	0.841454	-1.69686	0.801668
1	3.016686	-1.15035	-0.19042
1	5.12946	-1.823	0.942217
1	5.025183	-3.09573	3.087419
1	2.829492	-3.66432	4.088038
1	1.235676	-5.75172	4.838521
1	0.202988	-6.86726	6.793523
1	-2.26903	-7.16397	6.901493
1	-3.69123	-6.35446	5.026859

1	-2.65975	-5.24868	3.068057
1	-4.38129	-0.14231	5.517996
1	-4.76144	0.040252	7.972876
1	-4.27576	2.177732	9.151693
1	-3.40647	4.127529	7.869924
1	-3.01634	3.937119	5.426198
1	-2.40444	3.013282	1.400023
1	-1.65661	5.190202	0.469293
1	-2.9158	7.273513	0.991358
1	-4.94443	7.169152	2.427859
1	-5.71961	4.984796	3.327912
6	-5.60136	1.883486	0.785678
6	-0.82037	-5.25841	0.442323
8	-6.50406	2.670846	0.907118
8	-0.44895	-6.39574	0.58909
6	-5.19977	1.255567	-0.49703
6	-3.8684	0.906264	-0.76256
6	-6.1864	1.047757	-1.4694
6	-3.53648	0.312034	-1.97647
1	-3.07474	1.079874	-0.02992
6	-5.85086	0.441244	-2.67511
1	-7.21154	1.361044	-1.25906
6	-4.52744	0.067114	-2.92603
1	-2.49491	0.040005	-2.15683
1	-6.62168	0.264378	-3.4285
1	-4.26925	-0.40866	-3.87535
6	-1.2003	-4.68264	-0.87406
6	-1.7465	-5.56089	-1.82043
6	-0.96688	-3.34452	-1.21903
6	-2.1013	-5.09294	-3.08038
1	-1.88332	-6.6096	-1.54774
6	-1.30229	-2.8878	-2.49039
1	-0.51029	-2.63056	-0.53119
6	-1.8797	-3.75502	-3.41611
1	-2.5394	-5.77613	-3.81121
1	-1.09206	-1.84809	-2.74746
1	-2.14513	-3.39328	-4.4124
6	1.491474	0.886452	3.405446
6	0.966154	1.860573	2.421516
6	2.964516	0.694238	3.505826
6	0.898826	1.379668	0.947476
6	-0.36793	0.583294	0.61771
8	-0.3308	-0.2222	-0.31239
8	-1.38906	0.845107	1.33944
1	3.206311	-0.35381	3.24899
1	3.281555	0.857287	4.549064
1	1.639273	2.733131	2.452303
1	-0.04518	2.173427	2.704561
1	1.791454	0.818354	0.647589
1	0.874438	2.282979	0.315725
6	0.563056	0.203309	4.252262
8	-0.67043	0.481768	4.054059
6	0.8407	-0.76266	5.347044
6	2.08989	-1.31006	5.685984
6	-0.28582	-1.19445	6.0789
6	2.197525	-2.2737	6.686009
1	2.997057	-1.02697	5.163692
6	-0.17638	-2.16166	7.067465
1	-1.25815	-0.75454	5.854621
6	1.068364	-2.71532	7.37088
1	3.179832	-2.68736	6.92499
1	-1.06852	-2.48558	7.607246
1	1.157598	-3.48055	8.14504
6	3.790955	1.584597	2.604637
8	4.083822	2.733137	3.209656
8	4.140502	1.299541	1.490747
6	4.865563	3.657709	2.459498
1	5.008773	4.530448	3.105277
1	4.343384	3.943455	1.535123
1	5.834607	3.213637	2.192294

TS2-S

6	-2.26729	-2.69815	-2.47169
6	-1.88859	-1.7633	-1.516
7	-0.58337	-1.54008	-1.26852
6	0.400699	-2.24982	-1.85887
6	0.084371	-3.19337	-2.82819
6	-1.26434	-3.40153	-3.14403
6	-2.7094	-0.86097	-0.70862
7	-4.05346	-0.91508	-0.441
6	-4.45245	0.400555	0.072522
6	-3.07726	0.969758	0.554617
7	-2.11	0.169793	-0.18355
6	1.701808	-1.80517	-1.33835
7	1.723171	-0.63709	-0.74866
6	3.046927	-0.40527	-0.18444
6	3.932889	-1.50144	-0.83841
7	2.907498	-2.44052	-1.34229

29	-0.10002	0.073515	-0.28873	1	5.276402	4.437263	-0.99986
6	-2.83169	0.92845	2.043551	1	4.112358	3.278576	-2.86841
6	-2.16877	-0.13661	2.656287	1	3.062646	1.059886	-2.49658
6	-1.94598	-0.13656	4.032335	1	3.385304	-2.73218	1.541056
6	-2.39328	0.928242	4.810973	1	4.990026	-3.80488	3.088356
6	-3.05628	1.997498	4.206567	1	7.421183	-3.82542	2.551984
6	-3.26827	2.000351	2.830625	1	8.234861	-2.77809	0.446169
6	-5.11564	1.269086	-0.97322	1	6.619712	-1.72825	-1.12451
6	-5.44836	2.58606	-0.63844	6	0.226753	3.536532	0.750051
6	-6.05206	3.423312	-1.57181	6	0.071076	3.62865	2.23553
6	-6.34097	2.94984	-2.85229	6	1.521701	3.927668	0.097584
6	-6.02586	1.634817	-3.18625	6	0.956203	2.565673	2.877241
6	-5.41499	0.799658	-2.25151	6	0.836945	1.307207	2.057841
6	3.602492	0.973602	-0.40541	8	0.38895	1.576926	0.842135
6	4.251879	1.633174	0.642028	8	1.11631	0.181353	2.406106
6	4.855502	2.87349	0.429853	1	0.356123	4.630522	2.589294
6	4.806875	3.46583	-0.8311	1	-0.9856	3.464174	2.480955
6	4.154763	2.815192	-1.88022	1	2.23552	3.091553	0.17972
6	3.560924	1.573247	-1.66952	1	1.384664	4.139269	-0.96727
6	4.903475	-2.15231	0.1067	1	0.687158	2.352143	3.920259
6	4.450677	-2.74319	1.292093	1	2.012599	2.883024	2.868518
6	5.351171	-3.34527	2.165719	8	2.542241	5.183117	1.894227
6	6.71369	-3.35569	1.865032	8	2.569645	6.003605	-0.18499
6	7.169762	-2.76778	0.687345	6	-4.93017	-2.00888	-0.36311
6	6.266194	-2.17313	-0.19138	6	3.323856	-3.66658	-1.91321
1	-3.31862	-2.87662	-2.69379	8	-6.12014	-1.81539	-0.35133
1	0.859977	-3.76239	-3.33759	8	4.392955	-3.71808	-2.46695
1	-1.53206	-4.12614	-3.9141	6	-4.32677	-3.36385	-0.23459
1	-5.14378	0.249204	0.914018	6	-4.95044	-4.43253	-0.88664
1	-3.00219	2.013704	0.228342	6	-3.21863	-3.59973	0.587847
1	2.970578	-0.57829	0.901629	6	-4.42936	-5.71756	-0.76857
1	4.480354	-1.08886	-1.7008	1	-5.84341	-4.23979	-1.48555
1	-1.78272	-0.95992	2.050846	6	-2.71889	-4.89045	0.727171
1	-1.40669	-0.96655	4.493089	1	-2.76434	-2.77877	1.1468
1	-2.21967	0.930014	5.889374	6	-3.31111	-5.94643	0.034523
1	-3.39907	2.841317	4.809633	1	-4.90658	-6.54875	-1.29215
1	-3.7496	2.858509	2.3547	1	-1.87129	-5.07358	1.39102
1	-5.23037	2.96881	0.362099	1	-2.91272	-6.95857	0.136895
1	-6.30463	4.449414	-1.29562	6	2.448998	-4.84658	-1.7106
1	-6.82143	3.603445	-3.58368	6	2.556825	-5.90309	-2.62349
1	-6.26129	1.251396	-4.18182	6	1.588115	-4.95734	-0.61151
1	-5.17819	-0.23036	-2.52165	6	1.772794	-7.04053	-2.46523
1	4.287128	1.166445	1.62989	1	3.262719	-5.81185	-3.45187
1	5.356996	3.380766	1.256662	6	0.820861	-6.10589	-0.4464

1	1.534309	-4.15426	0.12663
6	0.901899	-7.14038	-1.37863
1	1.84814	-7.85945	-3.18376
1	0.161933	-6.20017	0.418566
1	0.293899	-8.03883	-1.24955
6	2.245854	5.098167	0.730293
6	3.328436	7.115398	0.275763
1	4.287692	6.779542	0.695616
1	3.494588	7.754466	-0.59803
1	2.778593	7.66335	1.053677
6	-1.01392	3.610642	-0.06403
8	-2.031	4.073817	0.445366
6	-1.05591	3.006749	-1.4253
6	-0.04111	2.191425	-1.96571
6	-2.25811	3.134465	-2.13808
6	-0.24961	1.495438	-3.15556
1	0.924866	2.092925	-1.47061
6	-2.45744	2.449702	-3.3305
1	-3.04438	3.765214	-1.72068
6	-1.46028	1.613583	-3.83672
1	0.545599	0.859079	-3.55215
1	-3.40591	2.560131	-3.85906
1	-1.62182	1.06698	-4.76859

TS2S-R

6	-0.99502	-3.29137	-1.28175
6	-0.91546	-2.06943	-0.60477
7	0.169892	-1.28906	-0.79925
6	1.229757	-1.6633	-1.52725
6	1.215097	-2.86838	-2.21062
6	0.0681	-3.67362	-2.0931
6	-1.84387	-1.39713	0.28926
7	-3.05006	-1.81237	0.804819
6	-3.78325	-0.61153	1.217522
6	-2.59763	0.365681	1.485141
7	-1.56187	-0.15193	0.612544
6	2.22998	-0.5766	-1.53641
7	1.802228	0.606615	-1.19458
6	2.827901	1.600158	-1.4781
6	4.067214	0.764793	-1.94706
7	3.530618	-0.61504	-1.91151
29	0.182817	0.495068	-0.02398
6	-2.16491	0.415509	2.934001
6	-1.0184	-0.23817	3.390216
6	-0.64818	-0.16063	4.733484
6	-1.42008	0.572541	5.633175
6	-2.56789	1.227578	5.183628
6	-2.9357	1.148515	3.843308
6	-4.72889	-0.09477	0.160784
6	-5.75297	0.77498	0.547678
6	-6.60686	1.331657	-0.4007
6	-6.44855	1.019781	-1.7515
6	-5.43459	0.147338	-2.14303
6	-4.57855	-0.40587	-1.1917
6	2.357704	2.578703	-2.52757
6	2.689154	3.930257	-2.42387
6	2.277806	4.831176	-3.40419
6	1.531012	4.38839	-4.49346
6	1.199417	3.037627	-4.60488
6	1.613153	2.136448	-3.62775
6	5.283336	0.923747	-1.07555
6	5.224029	0.610362	0.288096
6	6.348927	0.771965	1.092137
6	7.538259	1.254923	0.544654
6	7.600217	1.567953	-0.81127
6	6.47731	1.397205	-1.61964
1	-1.86599	-3.93631	-1.1753
1	2.057655	-3.18564	-2.82294
1	0.016215	-4.6178	-2.63727
1	-4.34169	-0.83624	2.135327
1	-2.87885	1.375908	1.155236
1	3.072244	2.156423	-0.55959
1	4.321601	1.016802	-2.9861
1	-0.38771	-0.7878	2.688194
1	0.252154	-0.67659	5.075843
1	-1.12977	0.633959	6.684308
1	-3.17664	1.807215	5.881114
1	-3.82707	1.677969	3.493775
1	-5.89272	1.009934	1.606945
1	-7.41026	2.000321	-0.08342
1	-7.12389	1.446769	-2.49615
1	-5.31328	-0.11174	-3.19731
1	-3.79705	-1.09897	-1.5108
1	3.245986	4.287183	-1.55565
1	2.531982	5.888657	-3.3065
1	1.202625	5.097059	-5.25698
1	0.614714	2.685655	-5.45789
1	1.340473	1.081273	-3.71505

1	4.297312	0.23536	0.731765
1	6.298692	0.515192	2.153057
1	8.420137	1.382182	1.176496
1	8.530685	1.939068	-1.24632
1	6.530423	1.626289	-2.68682
6	0.75907	3.453572	2.17212
6	2.188467	3.473156	2.610257
6	-0.2801	3.259462	3.227787
6	2.584992	2.04952	2.994034
6	1.900142	1.099767	2.042283
8	0.851546	1.650449	1.46685
8	2.236829	-0.04608	1.811375
6	0.438733	4.069754	0.852947
1	2.289576	4.16182	3.464842
1	2.804135	3.850976	1.783245
1	0.021777	2.445552	3.904603
1	-1.24805	2.990678	2.800065
1	3.670154	1.880218	2.973399
1	2.246858	1.785709	4.010831
8	0.473332	4.953526	4.766825
8	-1.6333	5.025973	4.014833
6	4.401051	-1.71798	-2.08575
6	-3.56674	-3.07928	1.107909
8	5.317901	-1.62366	-2.8602
8	-4.74938	-3.19932	1.315475
6	4.16406	-2.90539	-1.22437
6	4.603476	-4.14738	-1.6989
6	3.590413	-2.80721	0.051536
6	4.423037	-5.29072	-0.92836
1	5.08403	-4.19579	-2.67856
6	3.431298	-3.95266	0.825679
1	3.278737	-1.84487	0.465271
6	3.832899	-5.19422	0.3333
1	4.753967	-6.26055	-1.30599
1	2.999757	-3.8687	1.825478
1	3.701373	-6.09188	0.942212
6	-2.60398	-4.20784	1.218985
6	-3.02323	-5.47201	0.789447
6	-1.33832	-4.05126	1.797275
6	-2.15709	-6.55764	0.877286
1	-4.03114	-5.5849	0.383993
6	-0.48269	-5.14374	1.900876
1	-1.02715	-3.07926	2.186179
6	-0.88423	-6.39221	1.425754

1	-2.47864	-7.54069	0.526562
1	0.501005	-5.02187	2.359047
1	-0.20779	-7.24685	1.49934
6	-0.41194	4.500078	4.092195
6	-1.86164	6.193269	4.800125
1	-1.18014	7.000503	4.497235
1	-2.90278	6.482098	4.620858
1	-1.70006	5.97642	5.865187
8	1.318475	4.616305	0.208528
6	-0.89123	3.766815	0.238634
6	-2.12945	4.099127	0.802824
6	-0.85797	3.082791	-0.98825
6	-3.30919	3.7271	0.158647
1	-2.18017	4.668096	1.733096
6	-2.04148	2.68129	-1.60555
1	0.10578	2.900649	-1.47104
6	-3.27178	2.998172	-1.0305
1	-4.27259	4.002302	0.594154
1	-1.99975	2.134901	-2.55079
1	-4.20152	2.681937	-1.50753

9-S

6	-2.16064	-3.01711	-2.37175
6	-1.89664	-1.95529	-1.50593
7	-0.65488	-1.64135	-1.15742
6	0.391464	-2.28074	-1.66203
6	0.229812	-3.37417	-2.51497
6	-1.07168	-3.74196	-2.85616
6	-2.84401	-0.93117	-1.00374
7	-4.20535	-1.04059	-0.84621
6	-4.74697	0.31887	-0.75639
6	-3.47141	1.089689	-0.30049
7	-2.37754	0.256559	-0.78274
6	1.642526	-1.53122	-1.38977
7	1.54791	-0.24218	-1.34385
6	2.885173	0.315793	-1.17959
6	3.857431	-0.89044	-1.36551
7	2.914699	-2.02037	-1.3023
29	-0.32466	0.466428	-0.73928
6	-3.39864	1.294454	1.194476
6	-2.74766	0.38575	2.033684
6	-2.78516	0.556052	3.418217
6	-3.4396	1.651979	3.97516
6	-4.07003	2.575	3.140931

6	-4.05752	2.389671	1.762153	1	8.306727	-0.62605	0.028789
6	-5.28799	0.843462	-2.06225	1	6.529955	-0.55276	-1.70734
6	-6.24472	1.861911	-2.02887	6	0.729459	2.420847	2.057139
6	-6.6992	2.445566	-3.20874	6	0.559468	2.04373	3.543043
6	-6.20266	2.013177	-4.43778	6	2.032915	3.16464	1.760611
6	-5.25483	0.992194	-4.4786	6	0.74417	0.53621	3.595577
6	-4.79891	0.411061	-3.29692	6	0.759254	0.089411	2.165642
6	3.142026	1.452112	-2.13385	8	0.694598	1.169878	1.339439
6	3.797497	2.610304	-1.70969	8	0.793073	-1.01806	1.712417
6	4.043652	3.651541	-2.60465	1	1.284159	2.578018	4.169348
6	3.64005	3.541838	-3.93328	1	-0.44237	2.35234	3.867868
6	2.991495	2.384472	-4.36634	1	2.088091	3.498407	0.713712
6	2.743656	1.346776	-3.47154	1	2.049179	4.090145	2.358684
6	4.934041	-0.96723	-0.31795	1	-0.05989	0.004196	4.120868
6	4.60467	-1.23305	1.015427	1	1.699094	0.227754	4.040044
6	5.600447	-1.29123	1.985719	8	3.316176	1.283288	2.564141
6	6.932783	-1.07156	1.635183	8	4.365547	3.052808	1.693027
6	7.264537	-0.79284	0.310012	6	-5.05216	-2.14641	-0.68621
6	6.268175	-0.74712	-0.66428	6	3.382976	-3.34617	-1.28973
1	-3.17724	-3.27093	-2.67081	8	-6.24096	-2.00532	-0.834
1	1.086265	-3.90861	-2.92635	8	4.455984	-3.59958	-1.7802
1	-1.23784	-4.58053	-3.53506	6	-4.4286	-3.43776	-0.29072
1	-5.53481	0.340525	0.007505	6	-4.95899	-4.61447	-0.83075
1	-3.4312	2.064318	-0.80231	6	-3.38472	-3.50579	0.639098
1	2.971863	0.684477	-0.14367	6	-4.40405	-5.84482	-0.49357
1	4.318951	-0.86448	-2.36537	1	-5.80357	-4.54592	-1.52004
1	-2.20723	-0.46172	1.604721	6	-2.84847	-4.74023	0.991925
1	-2.30875	-0.18209	4.0677	1	-3.00349	-2.59531	1.106341
1	-3.46208	1.787495	5.058605	6	-3.34395	-5.90749	0.412498
1	-4.57608	3.44295	3.568539	1	-4.80608	-6.76165	-0.93018
1	-4.56513	3.108184	1.112609	1	-2.04088	-4.79125	1.723856
1	-6.64773	2.193832	-1.06762	1	-2.9125	-6.87466	0.680546
1	-7.45534	3.232921	-3.16929	6	2.537781	-4.3522	-0.59565
1	-6.56385	2.463981	-5.36474	6	2.649238	-5.688	-1.00018
1	-4.87155	0.640195	-5.43915	6	1.714389	-4.01445	0.486443
1	-4.06353	-0.39566	-3.34485	6	1.908229	-6.67368	-0.35813
1	4.116635	2.706911	-0.66956	1	3.327184	-5.93413	-1.82045
1	4.553324	4.553713	-2.25905	6	0.996155	-5.01074	1.141744
1	3.832016	4.35733	-4.63404	1	1.635588	-2.9841	0.83981
1	2.676553	2.291626	-5.40837	6	1.080416	-6.3351	0.714438
1	2.223952	0.446232	-3.80928	1	1.98645	-7.7135	-0.68286
1	3.56174	-1.39048	1.301849	1	0.377711	-4.74604	2.001962
1	5.332535	-1.50116	3.023234	1	0.51141	-7.1132	1.229121
1	7.715044	-1.12138	2.396148	6	3.285003	2.381031	2.065399

6	5.623258	2.407691	1.89723
1	5.717653	1.533619	1.237419
1	6.387241	3.154179	1.65619
1	5.720369	2.075903	2.939497
6	-0.43145	3.313326	1.560565
8	-1.06586	3.944272	2.372954
6	-0.72332	3.419825	0.10167
6	0.070899	2.864053	-0.92009
6	-1.86809	4.148973	-0.25362
6	-0.29739	3.016195	-2.26002
1	1.030149	2.393589	-0.70193
6	-2.23855	4.286248	-1.58591
1	-2.45478	4.597068	0.549966
6	-1.46037	3.706523	-2.59185
1	0.348079	2.601027	-3.03661
1	-3.13854	4.847305	-1.84745
1	-1.75332	3.81278	-3.63862

9-R

6	-1.14274	-3.12738	-1.39555
6	-0.97561	-2.03182	-0.54816
7	0.166263	-1.35235	-0.51679
6	1.175862	-1.65137	-1.3223
6	1.116047	-2.74919	-2.1829
6	-0.06419	-3.49225	-2.20198
6	-1.99276	-1.35137	0.288937
7	-3.16224	-1.86293	0.807634
6	-3.99649	-0.71954	1.187289
6	-2.89294	0.352563	1.402527
7	-1.83374	-0.08513	0.505668
6	2.175552	-0.55407	-1.33949
7	1.71249	0.647269	-1.21429
6	2.788401	1.595595	-1.47772
6	4.041391	0.719082	-1.81608
7	3.513749	-0.63679	-1.60346
29	-0.04533	0.767802	-0.06507
6	-2.39278	0.466442	2.824955
6	-1.2335	-0.18276	3.260205
6	-0.81704	-0.0701	4.586732
6	-1.54614	0.701615	5.489272
6	-2.69602	1.365081	5.058886
6	-3.11506	1.246602	3.736103
6	-4.95686	-0.28004	0.107774
6	-5.99742	0.585615	0.459194
6	-6.83415	1.121577	-0.51584
6	-6.64134	0.793876	-1.85838
6	-5.61465	-0.07851	-2.21379
6	-4.77636	-0.61184	-1.2355
6	2.428367	2.554407	-2.58414
6	2.821236	3.89218	-2.51135
6	2.517095	4.771495	-3.54899
6	1.815021	4.32104	-4.66484
6	1.420074	2.985084	-4.74376
6	1.728051	2.105197	-3.70965
6	5.22794	1.045559	-0.95022
6	5.309688	0.587913	0.369081
6	6.379778	0.96516	1.177805
6	7.369201	1.814352	0.68182
6	7.288729	2.275229	-0.63072
6	6.225577	1.887696	-1.4445
1	-2.08069	-3.68039	-1.43655
1	1.948039	-3.0054	-2.83917
1	-0.15667	-4.34571	-2.87632
1	-4.54216	-0.9585	2.109234
1	-3.2627	1.330612	1.076155
1	2.985114	2.179793	-0.56372
1	4.319035	0.834398	-2.87435
1	-0.62814	-0.75239	2.551892
1	0.088511	-0.58769	4.91311
1	-1.21871	0.791705	6.527348
1	-3.26694	1.980156	5.757856
1	-4.01449	1.771906	3.401448
1	-6.15861	0.839239	1.511259
1	-7.64918	1.788807	-0.22636
1	-7.30078	1.207589	-2.62448
1	-5.46723	-0.35159	-3.26121
1	-3.98019	-1.29998	-1.52771
1	3.34633	4.254526	-1.62514
1	2.820581	5.818163	-3.47791
1	1.570981	5.012627	-5.47429
1	0.868835	2.628079	-5.6168
1	1.408055	1.061518	-3.76762
1	4.537721	-0.07359	0.769219
1	6.447936	0.583926	2.199887
1	8.209269	2.107484	1.315346
1	8.06546	2.931465	-1.02947
1	6.17204	2.238628	-2.47835
6	0.945732	3.11564	2.111788

6	2.385646	3.421527	2.530917
6	-0.02951	3.171868	3.275008
6	2.940918	2.059577	2.919012
6	2.10062	1.083207	2.143923
8	1	1.728311	1.671193
8	2.272303	-0.079	1.920537
6	0.53444	3.868213	0.813674
1	2.421298	4.163979	3.338146
1	2.921801	3.844143	1.67075
1	0.351125	2.587001	4.1239
1	-0.98475	2.700075	2.99637
1	4.001436	1.909554	2.674774
1	2.817436	1.823964	3.989302
8	-0.28303	5.520492	2.935076
8	-0.59887	4.679113	4.986078
6	4.34733	-1.75843	-1.75357
6	-3.60722	-3.16946	1.049661
8	5.317367	-1.68397	-2.4668
8	-4.7846	-3.36731	1.225365
6	4.011476	-2.96609	-0.95304
6	4.398035	-4.21179	-1.46135
6	3.412697	-2.88196	0.310868
6	4.145084	-5.36978	-0.73426
1	4.900542	-4.2514	-2.43032
6	3.189047	-4.04259	1.046033
1	3.133134	-1.91818	0.742165
6	3.539887	-5.28566	0.521192
1	4.435135	-6.34191	-1.13872
1	2.749113	-3.96889	2.042844
1	3.357615	-6.19479	1.099406
6	-2.58278	-4.2445	1.126467
6	-2.92212	-5.50782	0.629448
6	-1.33798	-4.04487	1.734387
6	-1.99793	-6.54612	0.682477
1	-3.91505	-5.65699	0.199429
6	-0.42621	-5.09346	1.807917
1	-1.08838	-3.076	2.172441
6	-0.74735	-6.33749	1.266601
1	-2.25657	-7.52698	0.27774
1	0.53952	-4.93986	2.291869
1	-0.02498	-7.1556	1.314417
6	-0.30352	4.584835	3.69819
6	-0.92997	5.980149	5.463378
1	-1.81426	6.370224	4.939741
1	-1.13646	5.868439	6.533008
1	-0.09313	6.674321	5.303486
8	1.365915	4.489968	0.204253
6	-0.80386	3.573514	0.20562
6	-2.03623	3.849582	0.811494
6	-0.78257	3.029365	-1.09115
6	-3.22253	3.556739	0.14084
1	-2.0713	4.354902	1.775284
6	-1.9755	2.69524	-1.73334
1	0.171009	2.917468	-1.61134
6	-3.19849	2.950064	-1.11597
1	-4.18015	3.801203	0.60641
1	-1.94169	2.25428	-2.73195
1	-4.13474	2.687461	-1.61428

3u-S

6	0.728875	2.343034	0.96849
6	0.458863	3.540769	1.906624
6	2.19858	2.16034	0.588697
6	0.662722	2.930076	3.281036
6	0.336326	1.4673	3.075418
8	0.365143	1.196143	1.74422
8	0.074321	0.632117	3.884359
1	1.118142	4.380698	1.665896
1	-0.5831	3.874149	1.786844
1	2.800904	2.202941	1.511829
1	2.332629	1.156401	0.166829
1	0.029405	3.345358	4.075292
1	1.706418	2.998185	3.629744
6	2.809868	3.125259	-0.3974
8	3.607994	2.812565	-1.23623
8	2.437072	4.395589	-0.17657
6	2.933377	5.346397	-1.1025
1	2.569888	5.119094	-2.11536
1	2.55664	6.322397	-0.77474
1	4.032832	5.345111	-1.11689
6	-0.14341	2.415562	-0.29669
8	0.402422	2.672071	-1.34754
6	-1.6328	2.300407	-0.23372
6	-2.32868	2.839836	-1.32801
6	-2.36247	1.702692	0.805895
6	-3.71609	2.806438	-1.37513
1	-1.74694	3.285578	-2.13691
6	-3.75384	1.664336	0.750429

1	-1.85449	1.231403	1.643911
6	-4.43335	2.218973	-0.33206
1	-4.24275	3.237696	-2.2295
1	-4.30981	1.186689	1.56023
1	-5.52507	2.18789	-0.36684

ClO_4^-

17	0.583692	0.327726	0.019448
8	-0.89265	0.31873	0.003996
8	1.05855	1.026225	1.230763
8	1.084892	-1.06122	0.024626
8	1.085294	1.02668	-1.18069

8. Characterization of products

((1s,3R)-adamantan-1-yl)methyl 2-((S)-2-benzoyl-5-oxotetrahydrofuran-2-yl)acetate (3a)

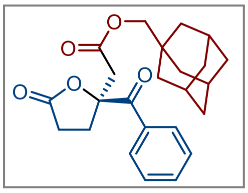

Purification by flash chromatography (PE/EA = 4/1) afforded **3a**. White solid; m.p. = 114−116 °C; 35.7 mg, 90% yield; Chiral HPLC conditions: ee = 94%; Chiralpak AD-H 95:5 Hexane/iPrOH, 1.0 mL/min, 35 min. t_R (minor) = 19.7 min. and t_R (major) = 23.3 min. λ = 245 nm.

$[\alpha]_D^{25}$ = +19.7 (c = 0.2, $CHCl_3$)

^{1}H NMR (400 MHz, $CDCl_3$) δ (ppm) = 8.03 (d, J = 7.6 Hz, 2H), 7.56 (t, J = 7.4 Hz, 1H), 7.45 (t, J = 7.6 Hz, 2H), 3.70 (d, J = 10.7 Hz, 1H), 3.64 (d, J = 10.7 Hz, 1H), 3.39 (d, J = 16.3 Hz, 1H), 3.05 (d, J = 16.3 Hz, 1H), 2.82−2.70 (m, 1H), 2.70−2.58 (m, 1H), 2.54−2.38 (m, 2H), 1.95 (s, 3H), 1.74−1.66 (m, 3H), 1.64−1.56 (m, 3H), 1.48−1.40 (m, 6H).

^{13}C NMR (100 MHz, $CDCl_3$) δ (ppm) = 198.9, 175.3, 168.6, 134.4, 133.1, 129.9, 128.4, 88.7, 74.8, 43.2, 39.0, 36.7, 32.9, 30.8, 27.8, 27.8.

HRMS (ESI) for $C_{24}H_{28}O_5Na$ $[M+Na]^+$ calcd. 419.1829, found 419.1834.

((1s,3R)-adamantan-1-yl)methyl 2-((S)-2-([1,1'-biphenyl]-4-carbonyl)-5-oxotetrahydrofuran-2-yl)acetate (3b)

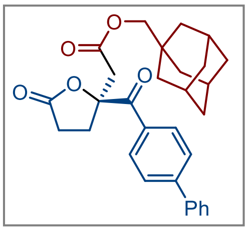

Purification by flash chromatography (PE/EA = 4/1) afforded **3b**. White solid; m.p. = 117−119 °C; 39.7 mg, 84% yield; Chiral HPLC conditions: ee = 94%; Chiralpak IA 95:5 Hexane/iPrOH, 1.0 mL/min, 40 min. t_R (minor) = 24.1 min. and t_R (major) = 32.1 min. λ = 286 nm.

$[\alpha]_D^{25}$ = +2.2 (c = 0.1, $CHCl_3$).

^{1}H NMR (400 MHz, $CDCl_3$) δ (ppm) = 8.14 (d, J = 8.5 Hz, 2H), 7.68 (d, J = 8.5 Hz, 2H), 7.64−7.57 (m, 2H), 7.47 (t, J = 7.4 Hz, 2H), 7.43−7.36 (m, 1H), 3.71 (d, J = 10.7 Hz, 1H), 3.65 (d, J = 10.7 Hz, 1H), 3.42 (d, J = 15.9 Hz, 1H), 3.07 (d, J = 15.9 Hz, 1H),

2.85−2.74 (m, 1H), 2.72−2.61 (m, 1H), 2.57−2.42 (m, 2H), 1.94 (s, 3H), 1.73−1.64 (m, 3H), 1.63−1.55 (m, 3H),1.50−1.41 (m, 6H).

^{13}C NMR (100 MHz, $CDCl_3$) δ (ppm) = 198.3, 175.3, 168.7, 145.8, 139.6, 133.0, 130.6, 128.9, 128.3, 127.2, 127.0, 88.8, 74.9, 43.3, 39.0, 36.7, 32.9, 30.9, 27.8, 27.8.

HRMS (ESI) for $C_{30}H_{32}O_5Na$ $[M+Na]^+$ calcd. 495.2142, found 495.2148.

((1s,3R)-adamantan-1-yl)methyl 2-((S)-2-(4-methylbenzoyl)-5-oxotetrahydrofuran-2-yl)acetate (3c)

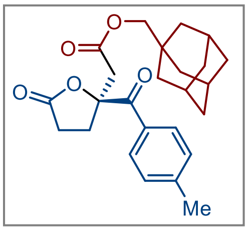

Purification by flash chromatography (PE/EA = 4/1) afforded **3c**. White solid; m.p. = 76−78 °C; 37.7 mg, 92% yield; Chiral HPLC conditions: ee = 96%; Chiralpak IA 95:5 Hexane/iPrOH, 1.0 mL/min, 40 min. t_R (minor) = 16.4 min. and t_R (major) = 18.4 min. λ = 256 nm.

$[α]_D^{25}$ = +7.9 (c = 0.1, $CHCl_3$).

^{1}H NMR (400 MHz, $CDCl_3$) δ (ppm) = 7.95 (d, *J* = 8.2 Hz, 2H), 7.26 (d, *J* = 8.2 Hz, 2H), 3.70 (d, *J* = 10.8 Hz, 1H), 3.64 (d, *J* = 10.8 Hz, 1H), 3.37 (d, *J* = 16.2 Hz, 1H), 3.04 (d, *J* = 16.2 Hz, 1H), 2.83−2.73 (m, 1H), 2.70−2.60 (m, 1H), 2.53−2.38 (m, 5H), 1.95 (s, 3H), 1.74−1.66 (m, 3H), 1.63−1.56 (m, 3H), 1.48−1.40 (s, 6H).

^{13}C NMR (100 MHz, $CDCl_3$) δ (ppm) = 198.2, 175.5, 168.7, 144.2, 131.7, 130.1, 129.2, 88.8, 74.8, 43.3, 39.0, 36.8, 33.0, 30.8, 27.9, 21.6.

HRMS (ESI) for $C_{25}H_{30}O_5Na$ $[M+Na]^+$ calcd. 433.1985, found 433.1993.

((1s,3R)-adamantan-1-yl)methyl 2-((S)-2-(4-methoxybenzoyl)-5-oxotetrahydrofuran-2-yl)acetate (3d)

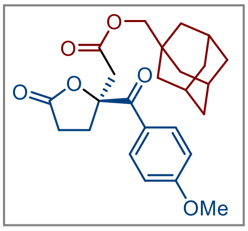

Purification by flash chromatography (PE/EA = 4/1) afforded **3d**. Colorless oil; 37.5 mg, 88% yield; Chiral HPLC conditions: ee = 96%; Chiralpak IA 95:5 Hexane/iPrOH, 1.0 mL/min, 40 min. t_R (minor) = 25.5 min. and t_R (major) = 29.9 min. λ = 278 nm.

$[α]_D^{25}$ = +8.4 (c = 0.1, $CHCl_3$).

^{1}H NMR (400 MHz, $CDCl_3$) δ (ppm) = 8.10 (d, J = 9.0 Hz, 2H), 7.56 (d, J = 9.0 Hz, 2H), 3.87 (s, 3H), 3.70 (d, J = 10.7 Hz, 1H), 3.64 (d, J = 10.7 Hz, 1H), 3.36 (d, J = 16.1 Hz, 1H), 3.01 (d, J = 16.1 Hz, 1H), 2.85−2.75 (m, 1H), 2.71−2.59 (m, 1H), 2.54−2.40 (m, 2H), 1.95 (s, 3H), 1.73−1.66 (m, 3H), 1.63−1.57 (m, 3H), 1.48−1.41 (m, 6H).

^{13}C NMR (100 MHz, $CDCl_3$) δ (ppm) = 196.8, 175.5, 168.7, 163.6, 132.6, 127.0, 113.7, 89.0, 74.8, 55.4, 43.3, 39.0, 36.8, 32.9, 30.9, 27.8, 27.8.

HRMS (ESI) for $C_{25}H_{30}O_6Na$ $[M+Na]^+$ calcd. 449.1935, found 449.1942.

((1s,3R)-adamantan-1-yl)methyl 2-((S)-2-(4-(tert-butyl)benzoyl)-5-oxotetrahydrofuran-2-yl)acetate (3e)

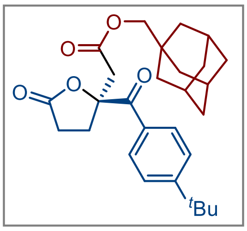

Purification by flash chromatography (PE/EA = 4/1) afforded **3e**. White solid; m.p. = 125−127 °C; 34.8 mg, 77% yield; Chiral HPLC conditions: ee = 91%; Chiralpak IA 95:5 Hexane/iPrOH, 1.0 mL/min, 40 min. t_R (minor) = 11.4 min. and t_R (major) = 15.1 min. λ = 257 nm.

$[\alpha]_D^{25}$ = +8.1 (c = 0.1, $CHCl_3$).

^{1}H NMR (400 MHz, $CDCl_3$) δ (ppm) = 8.01 (d, J = 8.6 Hz, 2H), 7.47 (d, J = 8.6 Hz, 2H), 3.69 (d, J = 10.7 Hz, 1H), 3.64 (d, J = 10.7 Hz, 1H), 3.38 (d, J = 16.2 Hz, 1H), 3.04 (d, J = 16.2 Hz, 1H), 2.83−2.72 (m, 1H), 2.71−2.59 (m, 1H), 2.56−2.40 (m, 2H), 1.94 (s, 3H), 1.73−1.64 (m, 3H), 1.62−1.55 (m, 3H), 1.47−1.40 (m, 6H), 1.33 (s, 9H).

^{13}C NMR (100 MHz, $CDCl_3$) δ (ppm) = 198.2, 175.5, 168.7, 157.1, 131.6, 130.1, 125.5, 88.9, 74.9, 43.3, 39.0, 36.8, 35.1, 33.0, 30.9, 30.9, 27.9.

HRMS (ESI) for $C_{28}H_{36}O_5Na$ $[M+Na]^+$ calcd. 475.2455, found 475.2462.

((1s,3R)-adamantan-1-yl)methyl 2-((S)-2-(4-(dimethylamino)benzoyl)-5-oxotetrahydrofuran-2-yl)acetate (3f)

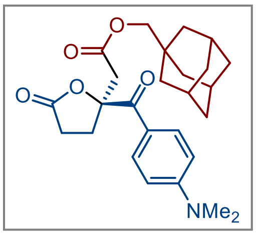

Purification by flash chromatography (PE/EA = 3/1) afforded **3f**. White solid; m.p. = 55−57 °C; 18.4 mg, 42% yield; Chiral HPLC conditions: ee = 95%; Chiralpak IA 95:5 Hexane/iPrOH, 1.0 mL/min, 80 min. t_R (minor) = 43.9 min. and t_R (major) = 56.4 min. λ = 336 nm.

$[\alpha]_D^{25}$ = -7.8 (c = 0.1, $CHCl_3$).

^{1}H NMR (400 MHz, $CDCl_3$) δ (ppm) = 8.06 (d, *J* = 9.2 Hz, 2H), 6.64 (d, *J* = 9.2 Hz, 2H), 3.69 (d, *J* = 10.7 Hz, 1H), 3.64 (d, *J* = 10.7 Hz, 1H), 3.32 (d, *J* = 15.9 Hz, 1H), 3.06 (s, 6H), 3.00 (d, *J* = 15.9 Hz, 1H), 2.92−2.83 (m, 1H), 2.70−2.60 (m, 1H), 2.55−2.43 (m, 2H), 1.95 (s, 3H), 1.73−1.67 (m, 3H), 1.63−1.57 (m, 3H), 1.49−1.42 (m, 6H).

^{13}C NMR (100 MHz, $CDCl_3$) δ (ppm) = 195.1, 175.9, 168.9, 153.5, 132.7, 121.3, 110.6, 89.3, 74.8, 43.3, 39.9, 39.0, 36.8, 33.0, 30.7, 28.0, 27.9.

HRMS (ESI) for $C_{26}H_{33}NO_5Na$ $[M+Na]^+$ calcd. 462.2251, found 462.2260.

((1s,3R)-adamantan-1-yl)methyl 2-((S)-2-(4-fluorobenzoyl)-5-oxotetrahydrofuran-2-yl)acetate (3g)

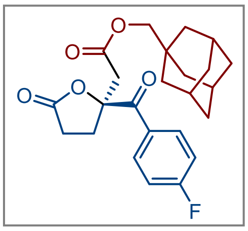

Purification by flash chromatography (PE/EA = 4/1) afforded **3g**. White solid; m.p. = 68−70 °C; 38.5 mg, 93% yield; Chiral HPLC conditions: ee = 94%; Chiralpak IA 95:5 Hexane/iPrOH, 1.0 mL/min, 40 min. t_R (minor) = 15.8 min. and t_R (major) = 18.3 min. λ = 248 nm.

$[\alpha]_D^{25}$ = +12.6 (c = 0.1, $CHCl_3$).

^{1}H NMR (400 MHz, $CDCl_3$) δ (ppm) = 8.17−8.06 (m, 2H), 7.19−7.09 (m, 2H), 3.70 (d, *J* = 10.7 Hz, 1H), 3.64 (d, *J* = 10.7 Hz, 1H), 3.39 (d, *J* = 16.3 Hz, 1H), 3.02 (d, *J* = 16.3 Hz, 1H), 2.79−2.59 (m, 2H), 2.54−2.38 (m, 2H), 1.95 (s, 3H), 1.75−1.65 (m, 3H), 1.64−1.57 (m, 3H),1.49−1.41 (m, 6H).

^{13}C NMR (100 MHz, $CDCl_3$) δ (ppm) = 197.7, 175.1, 168.7, 165.7 (d, *J* = 254.5 Hz), 133.0 (d, *J* = 9.1 Hz), 130.8 (d, *J* = 3.0 Hz), 115.6 (d, *J* = 21.5 Hz), 88.8, 74.9, 43.4,

39.0, 36.8, 33.0, 31.1, 27.8, 27.7.

^{19}F NMR (377 MHz, $CDCl_3$) δ (ppm) -104.1 – -104.2 (m).

HRMS (ESI) for $C_{24}H_{27}FO_5Na$ $[M+Na]^+$ calcd. 437.1735, found 437.1739.

((1s,3R)-adamantan-1-yl)methyl 2-((S)-2-(4-chlorobenzoyl)-5-oxotetrahydrofuran-2-yl)acetate (3h)

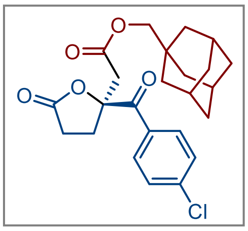

Purification by flash chromatography (PE/EA = 4/1) afforded **3h**. White solid; m.p. = 78−80 °C; 38.7 mg, 90% yield; Chiral HPLC conditions: ee = 91%; Chiralpak IA 95:5 Hexane/iPrOH, 1.0 mL/min, 40 min. t_R (minor) = 16.6 min. and t_R (major) = 19.8 min. λ = 255 nm.

$[\alpha]_D^{25}$ = +15.0 (c = 0.1, $CHCl_3$).

^{1}H NMR (400 MHz, $CDCl_3$) δ (ppm) = 8.01 (d, J = 8.5 Hz, 2H), 7.44 (d, J = 8.5 Hz, 2H), 3.70 (d, J = 10.7 Hz, 1H), 3.64 (d, J = 10.7 Hz, 1H), 3.39 (d, J = 16.4 Hz, 1H), 3.01 (d, J = 16.4 Hz, 1H), 2.77−2.59 (m, 2H), 2.53−2.37 (m, 2H), 1.96 (s, 3H), 1.74−1.66 (m, 3H), 1.64−1.57 (m, 3H),1.49−1.39 (m, 6H).

^{13}C NMR (100 MHz, $CDCl_3$) δ (ppm) = 198.2, 175.1, 168.7, 139.8, 132.9, 131.5, 128.8, 88.7, 74.9, 43.4, 39.0, 36.8, 33.0, 31.1, 27.9, 27.7.

HRMS (ESI) for $C_{24}H_{27}ClO_5Na$ $[M+Na]^+$ calcd. 453.1439, found 453.1444.

((1s,3R)-adamantan-1-yl)methyl 2-((S)-2-(4-bromobenzoyl)-5-oxotetrahydrofuran-2-yl)acetate (3i)

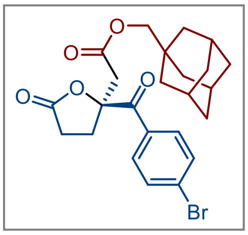

Purification by flash chromatography (PE/EA = 4/1) afforded **3i**. Colorless oil; 40.3 mg, 85% yield; Chiral HPLC conditions: ee = 89%; Chiralpak OD-H 95:5 Hexane/iPrOH, 1.0 mL/min, 30 min. t_R (minor) = 16.1 min. and t_R (major) = 24.6 min. λ = 261 nm.

$[\alpha]_D^{25}$ = +12.0 (c = 0.2, $CHCl_3$).

^{1}H NMR (400 MHz, $CDCl_3$) δ (ppm) = 7.92 (d, J = 8.6 Hz, 2H), 7.60 (d, J = 8.6 Hz, 2H), 3.70 (d, J = 10.7 Hz, 1H), 3.64 (d, J = 10.7 Hz, 1H), 3.39 (d, J = 16.4 Hz, 1H),

3.01 (d, J = 16.4 Hz, 1H), 2.77−2.59 (m, 2H), 2.53−2.37 (m, 2H), 1.96 (s, 3H), 1.73−1.67 (m, 3H), 1.63−1.57 (m, 3H), 1.50−1.41 (m, 6H).

^{13}C NMR (100 MHz, $CDCl_3$) δ (ppm) = 198.5, 175.1, 168.7, 133.3, 131.8, 131.6, 128.6, 88.7, 74.9, 43.4, 39.0, 36.8, 33.0, 31.1, 27.8, 27.7.

HRMS (ESI) for $C_{24}H_{27}BrO_5Na$ $[M+Na]^+$ calcd. 497.0934, found 497.0935.

((1s,3R)-adamantan-1-yl)methyl 2-((S)-2-(4-cyanobenzoyl)-5-oxotetrahydrofuran-2-yl)acetate (3j)

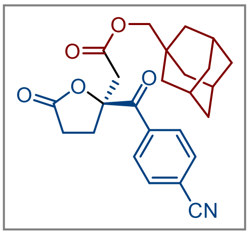

Purification by flash chromatography (PE/EA = 4/1) afforded **3j**. Colorless oil; 33.3 mg, 79% yield; Chiral HPLC conditions: ee = 90%; Chiralpak IA 95:5 Hexane/iPrOH, 1.0 mL/min, 60 min. t_R (minor) = 45.1 min. and t_R (major) = 50.9 min. λ = 250 nm.

$[\alpha]_D^{25}$ = +18.3 (c = 0.2, $CHCl_3$).

^{1}H NMR (400 MHz, $CDCl_3$) δ (ppm) = 8.11 (d, J = 8.2 Hz, 2H), 7.76 (d, J = 8.2 Hz, 2H), 3.71 (d, J = 10.7 Hz, 1H), 3.65 (d, J = 10.7 Hz, 1H), 3.45 (d, J = 16.6 Hz, 1H), 3.03 (d, J = 16.6 Hz, 1H), 2.71−2.61 (m, 2H), 2.53−2.38 (m, 2H), 1.96 (s, 3H), 1.74−1.68 (m, 3H), 1.64−1.57 (m, 3H), 1.50−1.42 (m, 6H).

^{13}C NMR (100 MHz, $CDCl_3$) δ (ppm) = 199.2, 174.7, 168.7, 138.2, 132.1, 130.4, 117.7, 116.2, 88.6, 75.0, 43.6, 39.0, 36.7, 33.0, 31.2, 27.8, 27.6.

HRMS (ESI) for $C_{25}H_{27}NO_5Na$ $[M+Na]^+$ calcd. 444.1781, found 444.1784.

((1s,3R)-adamantan-1-yl)methyl 2-((S)-5-oxo-2-(4-(trifluoromethyl)benzoyl) tetrahydrofuran-2-yl)acetate (3k)

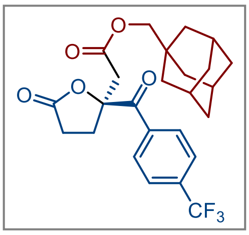

Purification by flash chromatography (PE/EA = 4/1) afforded **3k**. Colorless oil; 37.6 mg, 81% yield; Chiral HPLC conditions: ee = 85%; Chiralpak AD-H 95:5 Hexane/iPrOH, 1.0 mL/min, 60 min. t_R (minor) = 13.8 min. and t_R (major) = 19.1 min. λ = 238 nm.

$[\alpha]_D^{25}$ = +20.2 (c = 0.2, $CHCl_3$).

^{1}H NMR (400 MHz, $CDCl_3$) δ (ppm) = 8.14 (d, J = 8.2 Hz, 2H), 7.72 (d, J = 8.2 Hz,

2H), 3.71 (d, J = 10.7 Hz, 1H), 3.65 (d, J = 10.7 Hz, 1H), 3.44 (d, J = 16.6 Hz, 1H), 3.04 (d, J = 16.6 Hz, 1H), 2.74−2.60 (m, 2H), 2.53−2.38 (m, 2H), 1.96 (s, 3H), 1.74−1.68 (m, 3H), 1.64−1.57 (m, 3H), 1.50−1.42 (m, 6H).

^{13}C NMR (100 MHz, $CDCl_3$) δ (ppm) = 199.2, 174.9, 168.7, 137.7, 134.2 (q, J = 32.6 Hz), 130.3, 125.4 (q, J = 3.7 Hz), 123.4 (q, J = 271.0 Hz), 88.6, 75.0, 43.5, 39.0, 36.7, 33.0, 31.1, 27.8, 27.7.

^{19}F NMR (376 MHz, $CDCl_3$) δ (ppm) = -63.3 (s).

HRMS (ESI) for $C_{25}H_{27}F_3O_5Na$ $[M+Na]^+$ calcd. 487.1703, found 487.1704.

((1s,3R)-adamantan-1-yl)methyl 2-((S)-2-(3-methylbenzoyl)-5-oxotetrahydrofuran-2-yl)acetate (3l)

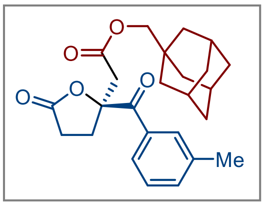

Purification by flash chromatography (PE/EA = 4/1) afforded **3l**. Colorless oil; 32.0 mg, 78% yield; Chiral HPLC conditions: ee = 95%; Chiralpak IA 95:5 Hexane/iPrOH, 1.0 mL/min, 40 min. t_R (minor) = 13.7 min. and t_R (major) = 16.0 min. λ = 249 nm.

$[\alpha]_D^{25}$ = +7.3 (c = 0.1, $CHCl_3$).

^{1}H NMR (400 MHz, $CDCl_3$) δ (ppm) = 7.89−7.75 (m, 2H), 7.42−7.30 (m, 2H), 3.69 (d, J = 10.6 Hz, 1H), 3.65 (d, J = 10.6 Hz, 1H), 3.38 (d, J = 16.3 Hz, 1H), 3.05 (d, J = 16.3 Hz, 1H), 2.82−2.71 (m, 1H), 2.70−2.59 (m, 1H), 2.54−2.36 (m, 5H), 1.95 (s, 3H), 1.74−1.66 (m, 3H), 1.64−1.56 (m, 3H),1.51−1.40 (m, 6H).

^{13}C NMR (100 MHz, $CDCl_3$) δ (ppm) = 198.9, 175.4, 168.7, 138.3, 134.4, 134.0, 130.3, 128.3, 127.1, 88.7, 74.8, 43.2, 39.0, 36.8, 33.0, 30.8, 27.8, 21.3.

HRMS (ESI) for $C_{25}H_{30}O_5Na$ $[M+Na]^+$ calcd. 433.1985, found 433.1993.

((1s,3R)-adamantan-1-yl)methyl 2-((S)-2-(3-methoxybenzoyl)-5-oxotetrahydrofuran-2-yl)acetate (3m)

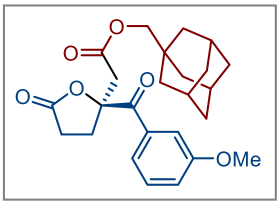

Purification by flash chromatography (PE/EA = 4/1) afforded **3m**. Colorless oil; 32.4 mg, 76% yield; Chiral HPLC conditions: ee = 98%; Chiralpak OD-H 95:5 Hexane/iPrOH, 1.0 mL/min,

60 min. t_R (minor) = 19.3 min. and t_R (major) = 25.3 min. λ = 309 nm.

[α]$_D$^{25} = +12.3 (c = 0.1, $CHCl_3$).

^{1}H NMR (400 MHz, $CDCl_3$) δ (ppm) = 7.64 (d, *J* = 7.8 Hz, 1H), 7.56−7.50 (m, 1H), 7.37 (t, *J* = 8.0 Hz, 1H), 7.15−7.07 (m, 1H), 3.85 (s, 3H), 3.70 (d, *J* = 10.7 Hz, 1H), 3.65 (d, *J* = 10.7 Hz, 1H), 3.39 (d, *J* = 16.3 Hz, 1H), 3.05 (d, *J* = 16.3 Hz, 1H), 2.82−2.72 (m, 1H), 2.71−2.60 (m, 1H), 2.56−2.39 (m, 2H), 1.95 (s, 3H), 1.75−1.66 (m, 3H), 1.64−1.57 (m, 3H), 1.49−1.41 (m, 6H).

^{13}C NMR (100 MHz, $CDCl_3$) δ (ppm) = 198.5, 175.3, 168.7, 159.5, 135.6, 129.5, 122.4, 119.8, 114.4, 88.7, 74.9, 55.4, 43.3, 39.0, 36.8, 33.0, 30.9, 27.9, 27.8.

HRMS (ESI) for $C_{25}H_{30}O_6Na$ $[M+Na]^+$ calcd. 449.1935, found 449.1944.

((1s,3R)-adamantan-1-yl)methyl 2-((S)-2-(2-methylbenzoyl)-5-oxotetrahydrofuran-2-yl)acetate (3n)

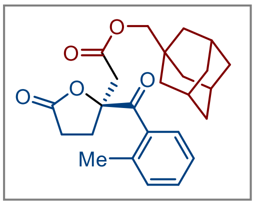

Purification by flash chromatography (PE/EA = 4/1) afforded **3n**. Colorless oil; 21.3 mg, 52% yield; Chiral HPLC conditions: ee = 91%; Chiralpak OD-H 95:5 Hexane/iPrOH, 1.0 mL/min, 40 min. t_R (minor) = 16.7 min. and t_R (major) = 19.1 min. λ = 245 nm.

[α]$_D$^{25} = +24.8 (c = 0.1, $CHCl_3$).

^{1}H NMR (400 MHz, $CDCl_3$) δ (ppm) = 7.74 (d, *J* = 7.6 Hz, 1H), 7.39−7.32 (m, 1H), 7.28−7.20 (m, 2H), 3.72 (d, *J* = 10.7 Hz, 1H), 3.67 (d, *J* = 10.7 Hz, 1H), 3.35 (d, *J* = 16.8 Hz, 1H), 3.06 (d, *J* = 16.8 Hz, 1H), 2.68−2.54 (m, 2H), 2.47−2.34 (m, 5H), 1.96 (s, 3H), 1.75−1.67 (m, 3H), 1.65−1.58 (m, 3H), 1.52−1.42 (m, 6H).

^{13}C NMR (100 MHz, $CDCl_3$) δ (ppm) = 202.4, 175.4, 169.1, 137.3, 135.1, 131.4, 131.1, 128.6, 125.3, 88.3, 74.7, 42.1, 39.1, 36.8, 33.1, 30.6, 27.9, 27.9, 20.2.

HRMS (ESI) for $C_{25}H_{30}O_5Na$ $[M+Na]^+$ calcd. 433.1985, found 433.1994.

((1s,3R)-adamantan-1-yl)methyl 2-((S)-2-(2-naphthoyl)-5-oxotetrahydrofuran-2-yl)acetate (3o)

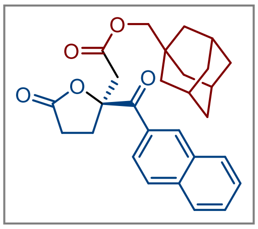

Purification by flash chromatography (PE/EA = 4/1) afforded **3o**. Colorless oil; 36.1 mg, 81% yield; Chiral HPLC conditions: ee = 94%; Chiralpak IA 95:5 Hexane/iPrOH, 1.0 mL/min, 40 min. t_R (minor) = 21.6 min. and t_R (major) = 27.8 min. λ = 252 nm.

[α]$_D$^{25} = -12.4 (c = 0.1, $CHCl_3$).

^{1}H NMR (400 MHz, $CDCl_3$) δ (ppm) = 8.66 (s, 1H), 8.04 (dd, *J* = 8.6, 1.7 Hz, 1H), 7.98 (d, *J* = 8.0 Hz, 1H), 7.91−7.83 (m, 2H), 7.64−7.52 (m, 2H), 3.70 (d, *J* = 10.7 Hz, 1H), 3.65 (d, *J* = 10.7 Hz, 1H), 3.45 (d, *J* = 16.3 Hz, 1H), 3.12 (d, *J* = 16.3 Hz, 1H), 2.91−2.80 (m, 1H), 2.74−2.61 (m, 1H), 2.57−2.43 (m, 2H), 1.92 (s, 3H), 1.71−1.64 (m, 3H), 1.61−1.54 (m, 3H), 1.47−1.39 (m, 6H).

^{13}C NMR (100 MHz, $CDCl_3$) δ (ppm) = 198.5, 175.4, 168.7, 135.4, 132.2, 132.0, 131.6, 129.9, 128.8, 128.2, 127.6, 126.8, 125.4, 88.9, 74.9, 43.4, 39.0, 36.7, 33.0, 30.9, 27.8.

HRMS (ESI) for $C_{28}H_{30}O_5Na$ $[M+Na]^+$ calcd. 469.1985, found 469.1994.

((1s,3R)-adamantan-1-yl)methyl 2-((S)-5-oxo-2-(thiophene-2-carbonyl)tetrahydrofuran-2-yl)acetate (3p)

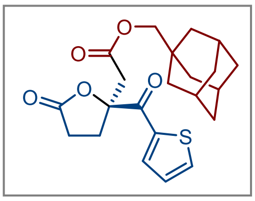

Purification by flash chromatography (PE/EA = 4/1) afforded **3p**. White solid; m.p. = 77−79 °C; 38.6 mg, 96% yield; Chiral HPLC conditions: ee = 99%; Chiralpak OD-H 95:5 Hexane/iPrOH, 1.0 mL/min, 40 min. t_R (minor) = 22.1 min. and t_R (major) = 24.9 min. λ = 287 nm.

[α]$_D$^{25} = +31.1 (c = 0.1, $CHCl_3$).

^{1}H NMR (400 MHz, $CDCl_3$) δ (ppm) = 8.12 (d, *J* = 3.9 Hz, 1H), 7.74 (d, *J* = 4.9 Hz, 1H), 7.17 (t, *J* = 4.4 Hz, 1H), 3.70 (d, *J* = 10.7 Hz, 1H), 3.63 (d, *J* = 10.7 Hz, 1H), 3.38 (d, *J* = 16.3 Hz, 1H), 2.98 (d, *J* = 16.3 Hz, 1H), 2.78−2.64 (m, 2H), 2.61−2.46 (m, 2H), 1.93 (s, 3H), 1.72−1.65 (m, 3H), 1.62−1.55 (m, 3H), 1.48−1.40 (m, 6H).

^{13}C NMR (100 MHz, $CDCl_3$) δ (ppm) = 191.3, 175.3, 168.6, 139.2, 135.8, 135.7, 128.6, 88.3, 74.9, 42.9, 39.0, 36.8, 32.9, 30.9, 27.9, 27.7.

HRMS (ESI) for $C_{22}H_{26}O_5SNa$ $[M+Na]^+$ calcd. 425.1393, found 425.1401.

((1s,3R)-adamantan-1-yl)methyl 2-((S)-2-(furan-2-carbonyl)-5-oxotetrahydrofuran-2-yl)acetate (3q)

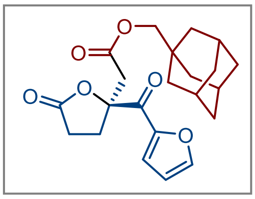

Purification by flash chromatography (PE/EA = 4/1) afforded **3q**. Colorless oil; 30.5 mg, 79% yield; Chiral HPLC conditions: ee = 98%; Chiralpak OX-H 95:5 Hexane/iPrOH, 1.0 mL/min, 85 min. t_R (minor) = 59.9 min. and t_R (major) = 73.0 min. λ = 271 nm.

$[\alpha]_D^{25}$ = +14.4 (c = 0.1, $CHCl_3$).

^{1}H NMR (400 MHz, $CDCl_3$) δ (ppm) = 7.68 (s, 1H), 7.55 (s, 1H), 6.59 (s, 1H), 3.80−3.57 (m, 2H), 3.37 (d, J = 16.5 Hz, 1H), 3.02 (d, J = 16.5 Hz, 1H), 2.78−2.45 (m, 4H), 1.95 (s, 3H), 1.75−1.66 (m, 3H), 1.64−1.55 (m, 3H), 1.51−1.37 (m, 6H).

^{13}C NMR (100 MHz, $CDCl_3$) δ (ppm) = 185.8, 175.7, 168.7, 149.4, 147.7, 122.2, 112.7, 87.2, 74.8, 42.2, 38.9, 36.7, 32.9, 30.4, 27.8, 27.7.

HRMS (ESI) for $C_{22}H_{26}O_6Na$ $[M+Na]^+$ calcd. 409.1622, found 409.1618.

((1s,3R)-adamantan-1-yl)methyl 2-((S)-5-oxo-2-phenyltetrahydrofuran-2-yl)acetate (3r)

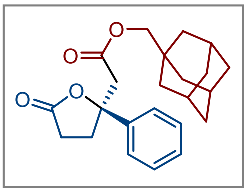

Purification by flash chromatography (PE/EA = 4/1) afforded **3a**. Colorless oil; 16.2 mg, 44% yield; Chiral HPLC conditions: ee = 65%; Chiralpak OX-H 95:5 Hexane/iPrOH, 1.0 mL/min, 35 min. t_R (minor) = 26.2 min. and t_R (major) = 28.8 min. λ = 208 nm.

$[\alpha]_D^{25}$ = +14.6 (c = 0.2, $CHCl_3$).

^{1}H NMR (400 MHz, $CDCl_3$) δ (ppm) = 7.44−7.34 (m, 4H), 7.33−7.28 (m, 1H), 3.65−3.57 (m, 2H), 3.04 (d, J = 15.4 Hz, 1H), 2.99 (d, J = 15.4 Hz, 1H), 2.93−2.84 (m, 1H), 2.73−2.57 (m, 2H), 2.54−2.44 (m, 1H), 1.93 (s, 3H), 1.72−1.66 (m, 3H), 1.62−1.56 (m, 3H), 1.45−1.35 (m, 6H).

^{13}C NMR (100 MHz, $CDCl_3$) δ (ppm) = 176.0, 169.1, 142.6, 128.6, 128.0, 124.5, 86.0, 74.5, 46.3, 39.0, 36.8, 33.3, 32.9, 28.6, 27.9.

HRMS (ESI) for $C_{23}H_{28}O_4Na$ $[M+Na]^+$ calcd. 391.1880, found 391.1888.

((1s,3R)-adamantan-1-yl)methyl 2-((S)-2-([1,1'-biphenyl]-4-yl)-5-oxotetrahydrofuran-2-yl)acetate (3s)

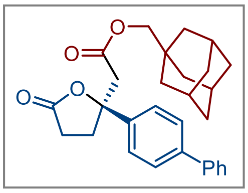

Purification by flash chromatography (PE/EA = 4/1) afforded **3s**. Colorless oil; 20.0 mg, 45% yield; Chiral HPLC conditions: ee = 70%; Chiralpak IA 95:5 Hexane/iPrOH, 1.0 mL/min, 60 min. t_R (minor) = 24.0 min. and t_R (major) = 26.9 min. λ = 253 nm.

[α]D^{25} = +12.5 (c = 0.2, $CHCl_3$).

^{1}**H NMR** (400 MHz, $CDCl_3$) δ (ppm) = 7.66−7.53 (m, 4H), 7.51−7.40 (m, 4H), 7.36 (t, *J* = 7.3 Hz, 1H), 3.68−3.56 (m, 2H), 3.13−3.00 (m, 2H), 2.95−2.85 (m, 1H), 2.77−2.60 (m, 2H), 2.58−2.47 (m, 1H), 1.91 (s, 3H), 1.70−1.64 (m, 3H), 1.61−1.55 (m, 3H), 1.45−1.34 (m, 6H).

^{13}**C NMR** (100 MHz, $CDCl_3$) δ (ppm) = 176.0, 169.1, 141.5, 141.0, 140.2, 128.8, 127.5, 127.3, 127.0, 125.1, 85.9, 74.6, 46.4, 39.0, 36.8, 33.4, 32.9, 28.6, 27.9.

HRMS (ESI) for $C_{29}H_{32}O_4Na$ $[M+Na]^+$ calcd. 467.2193, found: 467.2187.

((1s,3R)-adamantan-1-yl)methyl 2-((S)-2-(4-chlorophenyl)-5-oxotetrahydrofuran-2-yl)acetate (3t)

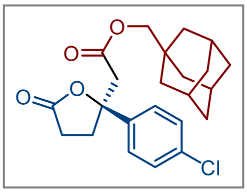

Purification by flash chromatography (PE/EA = 4/1) afforded **3t**. Colorless oil; 18.9 mg, 47% yield; Chiral HPLC conditions: ee = 71%; Chiralpak OX-H 95:5 Hexane/iPrOH, 1.0 mL/min, 35 min. t_R (minor) = 23.0 min. and t_R (major) = 27.7 min. λ = 221 nm.

[α]D^{25} = +11.8 (c = 0.2, $CHCl_3$).

^{1}**H NMR** (400 MHz, $CDCl_3$) δ (ppm) = 7.40−7.29 (m, 4H), 3.65−3.55 (m, 2H), 3.01 (d, *J* = 15.3 Hz, 1H), 2.96 (d, *J* = 15.3 Hz, 1H), 2.91−2.81 (m, 1H), 2.74−2.65 (m, 1H), 2.60−2.45 (m, 2H), 1.94 (s, 3H), 1.72−1.65 (m, 3H), 1.62−1.55 (m, 3H), 1.44−1.34 (m, 6H).

^{13}**C NMR** (100 MHz, $CDCl_3$) δ (ppm) = 175.6, 168.8, 141.0, 134.1, 128.8, 126.2, 85.5, 74.6, 46.3, 39.0, 36.8, 33.4, 32.9, 28.4, 27.9.

HRMS (ESI) for $C_{23}H_{27}ClO_4Na$ $[M+Na]^+$ calcd. 425.1490, found 425.1493.

methyl (S)-2-(2-benzoyl-5-oxotetrahydrofuran-2-yl)acetate (3u)

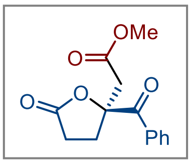

Purification by flash chromatography (PE/EA = 4/1) afforded **3u**. White solid; m.p. = 117−119 ºC; 21.5 mg, 82% yield; Chiral HPLC conditions: ee = 96%; Chiralpak ID 95:5 Hexane/iPrOH, 1.0 mL/min, 100 min. t_R (minor) = 65.9 min. and t_R (major) = 75.6 min. λ = 245 nm.

[α]$_D$^{25} = +40.6 (c = 0.1, $CHCl_3$).

^{1}H NMR (400 MHz, $CDCl_3$) δ (ppm) = 8.01 (d, J = 7.5 Hz, 2H), 7.57 (t, J = 7.5 Hz, 1H), 7.46 (t, J = 7.5 Hz, 2H), 3.67 (s, 3H), 3.40 (d, J = 16.4 Hz, 1H), 3.00 (d, J = 16.4 Hz, 1H), 2.78−2.60 (m, 2H), 2.54−2.40 (m, 2H).

^{13}C NMR (100 MHz, $CDCl_3$) δ (ppm) = 199.5, 175.3, 169.0, 134.6, 133.2, 129.9, 128.5, 88.8, 52.2, 43.0, 31.0, 27.8.

HRMS (ESI) for $C_{14}H_{14}O_5Na$ $[M+Na]^+$ calcd. 285.0733, found 285.0736.

methyl-d3 (S)-2-(2-benzoyl-5-oxotetrahydrofuran-2-yl)acetate (3v)

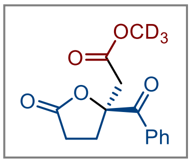

Purification by flash chromatography (PE/EA = 4/1) afforded **3v**. White solid; m.p. = 75−77 ºC; 20.9 mg, 79% yield; Chiral HPLC conditions: ee = 95%; Chiralpak OD-H 95:5 Hexane/iPrOH, 1.0 mL/min, 45 min. t_R (minor) = 27.1 min. and t_R (major) = 36.9 min. λ = 245 nm.

[α]$_D$^{25} = +40.4 (c = 0.1, $CHCl_3$).

^{1}H NMR (400 MHz, $CDCl_3$) δ (ppm) = 8.01 (d, J = 7.7 Hz, 2H), 7.57 (t, J = 7.3 Hz, 1H), 7.46 (t, J = 7.6 Hz, 2H), 3.40 (d, J = 16.4 Hz, 1H), 3.00 (d, J = 16.4 Hz, 1H), 2.78−2.60 (m, 2H), 2.54−2.40 (m, 2H).

^{13}C NMR (100 MHz, $CDCl_3$) δ (ppm) = 199.5, 175.3, 169.0, 134.6, 133.2, 129.9, 128.5, 88.8, 43.0, 31.0, 27.8.

HRMS (ESI) for $C_{14}H_{11}D_3O_5Na$ $[M+Na]^+$ calcd. 288.0922, found 288.0920.

ethyl (S)-2-(2-benzoyl-5-oxotetrahydrofuran-2-yl)acetate (3w)

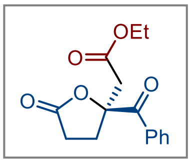

Purification by flash chromatography (PE/EA = 4/1) afforded **3w**. White solid; m.p. = 76−78 ºC; 23.2 mg, 84% yield; Chiral HPLC conditions: ee = 95%; Chiralpak OD-H 95:5 Hexane/iPrOH, 1.0 mL/min, 40 min. t_R (minor) = 21.3 min. and t_R (major) = 27.9 min. λ = 245 nm.

$[\alpha]_D^{25}$ = +23.6 (c = 0.1, $CHCl_3$).

^{1}H NMR (400 MHz, $CDCl_3$) δ (ppm) = 8.03 (dd, J = 8.0, 0.6 Hz, 2H), 7.56 (t, J = 7.4 Hz, 1H), 7.45 (t, J = 7.7 Hz, 2H), 4.19−4.06 (m, 2H), 3.38 (d, J = 16.4 Hz, 1H), 2.99 (d, J = 16.4 Hz, 1H), 2.77−2.57 (m, 2H), 2.53−2.39 (m, 2H), 1.20 (t, J = 7.1 Hz, 3H).

^{13}C NMR (100 MHz, $CDCl_3$) δ (ppm) = 199.3, 175.3, 168.4, 134.6, 133.1, 129.8, 128.4, 88.7, 61.2, 43.2, 30.9, 27.7, 13.9.

HRMS (ESI) for $C_{15}H_{16}O_5Na$ $[M+Na]^+$ calcd. 299.0890, found 299.0892.

cyclopentylmethyl (S)-2-(2-benzoyl-5-oxotetrahydrofuran-2-yl)acetate (3x)

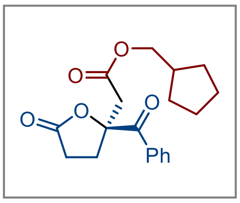

Purification by flash chromatography (PE/EA = 4/1) afforded **3x**. White solid; m.p. = 108−110 ºC; 29.4 mg, 89% yield; Chiral HPLC conditions: ee = 95%; Chiralpak OD-H 95:5 Hexane/iPrOH, 1.0 mL/min, 30 min. t_R (minor) = 18.3 min. and t_R (major) = 23.7 min. λ = 245 nm.

$[\alpha]_D^{25}$ = +28.1 (c = 0.1, $CHCl_3$).

^{1}H NMR (400 MHz, $CDCl_3$) δ (ppm) = 8.03 (d, J = 7.6 Hz, 2H), 7.57 (t, J = 7.4 Hz, 1H), 7.46 (t, J = 7.7 Hz, 2H), 4.00−3.90 (m, 2H), 3.38 (d, J = 16.3 Hz, 1H), 3.01 (d, J = 16.3 Hz, 1H), 2.79−2.60 (m, 2H), 2.53−2.40 (m, 2H), 2.18−2.06 (m, 1H), 1.73−1.63 (m, 2H), 1.60−1.46 (m, 4H), 1.21−1.11 (m, 2H).

^{13}C NMR (100 MHz, $CDCl_3$) δ (ppm) = 199.1, 175.3, 168.6, 134.5, 133.1, 129.9, 128.4, 88.8, 69.3, 43.3, 38.2, 30.9, 29.2, 29.2, 27.8, 25.2.

HRMS (ESI) for $C_{19}H_{22}O_5Na$ $[M+Na]^+$ calcd. 353.1359, found 353.1361.

2-isopropoxyethyl (S)-2-(2-benzoyl-5-oxotetrahydrofuran-2-yl)acetate (3y)

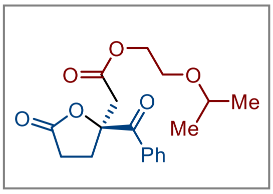

Purification by flash chromatography (PE/EA = 4/1) afforded **3y**. White solid; m.p. = 71−73 ºC; 17.0 mg, 51% yield; Chiral HPLC conditions: ee = 95%; Chiralpak OD-H 95:5 Hexane/iPrOH, 1.0 mL/min, 40 min. t_R (minor) = 23.1 min. and t_R (major) = 32.9 min. λ = 245 nm.

[α]D^{25} = +11.5 (c = 0.1, $CHCl_3$).

^{1}H NMR (400 MHz, $CDCl_3$) δ (ppm) = 8.02 (d, *J* = 7.4 Hz, 2H), 7.57 (t, *J* = 7.4 Hz, 1H), 7.46 (t, *J* = 7.7 Hz, 2H), 4.26−4.15 (m, 2H), 3.63−3.50 (m, 3H), 3.41 (d, *J* = 16.5 Hz, 1H), 3.04 (d, *J* = 16.5 Hz, 1H), 2.80−2.62 (m, 2H), 2.54−2.40 (m, 2H), 1.14 (d, *J* = 6.1 Hz, 6H).

^{13}C NMR (100 MHz, $CDCl_3$) δ (ppm) = 199.2, 175.3, 168.6, 134.6, 133.2, 129.9, 128.5, 88.7, 72.0, 65.5, 64.8, 43.1, 30.8, 27.8, 21.9.

HRMS (ESI) for $C_{18}H_{22}O_6Na$ $[M+Na]^+$ calcd. 357.1309, found 357.1311.

2-(trimethylsilyl)ethyl (S)-2-(2-benzoyl-5-oxotetrahydrofuran-2-yl)acetate (3z)

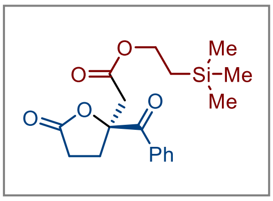

Purification by flash chromatography (PE/EA = 4/1) afforded **3z**. White solid; m.p. = 80−82 ºC; 24.0 mg, 69% yield; Chiral HPLC conditions: ee = 95%; Chiralpak OD-H 95:5 Hexane/iPrOH, 1.0 mL/min, 30 min. t_R (minor) = 13.3 min. and t_R (major) = 17.2 min. λ = 245 nm.

[α]D^{25} = +28.3 (c = 0.1, $CHCl_3$).

^{1}H NMR (400 MHz, $CDCl_3$) δ (ppm) = 8.01 (d, *J* = 7.4 Hz, 2H), 7.56 (t, *J* = 7.4 Hz, 1H), 7.45 (t, *J* = 7.7 Hz, 2H), 4.20−4.08 (m, 2H), 3.35 (d, *J* = 16.4 Hz, 1H), 2.96 (d, *J* = 16.4 Hz, 1H), 2.77−2.58 (m, 2H), 2.52−2.40 (m, 2H), 0.97−0.86 (m, 2H), 0.00 (s, 9H).

^{13}C NMR (100 MHz, $CDCl_3$) δ (ppm) = 199.3, 175.4, 168.6, 134.6, 133.2, 129.9, 128.5, 88.8, 63.7, 43.4, 30.9, 27.8, 17.2, -1.6.

HRMS (ESI) for $C_{17}H_{24}O_5SiNa$ $[M+Na]^+$ calcd. 371.1285, found 371.1289.

phenethyl (S)-2-(2-benzoyl-5-oxotetrahydrofuran-2-yl)acetate (3aa)

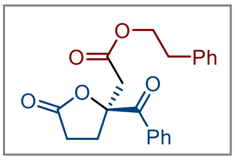

Purification by flash chromatography (PE/EA = 4/1) afforded **3aa**. White solid; m.p. = 95−97 °C; 24.6 mg, 70% yield; Chiral HPLC conditions: ee = 94%; Chiralpak IA 95:5 Hexane/iPrOH, 1.0 mL/min, 40 min. t_R (minor) = 25.1 min. and t_R (major) = 29.0 min. λ = 244 nm.

$[\alpha]_D^{25}$ = +27.2 (c = 0.1, $CHCl_3$).

^{1}H NMR (400 MHz, $CDCl_3$) δ (ppm) = 8.00 (d, *J* = 7.6 Hz, 2H), 7.57 (t, *J* = 7.4 Hz, 1H), 7.45 (t, *J* = 7.7 Hz, 2H), 7.32−7.19 (m, 3H), 7.16 (d, *J* = 7.2 Hz, 2H), 4.36−4.22 (m, 2H), 3.35 (d, *J* = 16.4 Hz, 1H), 2.97 (d, *J* = 16.4 Hz, 1H), 2.88 (t, *J* = 7.1 Hz, 2H), 2.75−2.55 (m, 2H), 2.49−2.34 (m, 2H).

^{13}C NMR (100 MHz, $CDCl_3$) δ (ppm) = 199.2, 175.3, 168.4, 137.3, 134.5, 133.2, 129.9, 128.8, 128.5, 126.6, 88.7, 65.7, 43.2, 34.8, 30.9, 27.7.

HRMS (ESI) for $C_{21}H_{20}O_5Na$ $[M+Na]^+$ calcd. 375.1203, found 375.1205.

hex-5-en-1-yl (S)-2-(2-benzoyl-5-oxotetrahydrofuran-2-yl)acetate (3ab)

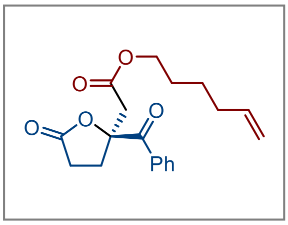

Purification by flash chromatography (PE/EA = 4/1) afforded **3ab**. White solid; m.p. = 79−81 °C; 28.7 mg, 87% yield; Chiral HPLC conditions: ee = 94%; Chiralpak OD-H 95:5 Hexane/iPrOH, 1.0 mL/min, 35 min. t_R (minor) = 19.2 min. and t_R (major) = 25.2 min. λ = 245 nm.

$[\alpha]_D^{25}$ = +33.4 (c = 0.1, $CHCl_3$).

^{1}H NMR (400 MHz, $CDCl_3$) δ (ppm) = 8.02 (d, *J* = 7.6 Hz, 2H), 7.57 (t, *J* = 7.4 Hz, 1H), 7.46 (t, *J* = 7.7 Hz, 2H), 5.82−5.68 (m, 1H), 5.04−4.91 (m, 2H), 4.13−4.01 (m, 2H), 3.39 (d, *J* = 16.4 Hz, 1H), 3.00 (d, *J* = 16.4 Hz, 1H), 2.78−2.58 (m, 2H), 2.54−2.39 (m, 2H), 2.08−1.97 (m, 2H), 1.63−1.53 (m, 2H), 1.43−1.32 (m, 2H).

^{13}C NMR (100 MHz, $CDCl_3$) δ (ppm) = 199.3, 175.3, 168.5, 138.1, 134.6, 133.2, 129.9, 128.5, 114.9, 88.8, 65.3, 43.3, 33.1, 31.0, 27.8, 27.7, 25.0.

HRMS (ESI) for $C_{19}H_{22}O_5Na$ $[M+Na]^+$ calcd. 353.1359, found 353.1362.

hept-6-yn-1-yl (S)-2-(2-benzoyl-5-oxotetrahydrofuran-2-yl)acetate (3ac)

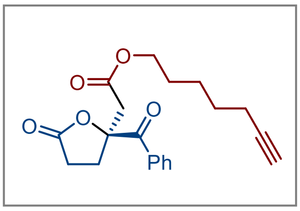

Purification by flash chromatography (PE/EA = 4/1) afforded **3ac**. White solid; m.p. = 75−77 °C; 30.1 mg, 88% yield; Chiral HPLC conditions: ee = 96%; Chiralpak IB 95:5 Hexane/iPrOH, 1.0 mL/min, 35 min. t_R (minor) = 19.6 min. and t_R (major) = 24.2 min. λ = 245 nm.

$[\alpha]_D^{25}$ = +29.1 (c = 0.1, $CHCl_3$).

^{1}H NMR (400 MHz, $CDCl_3$) δ (ppm) = 8.02 (d, J = 7.5 Hz, 2H), 7.57 (t, J = 7.4 Hz, 1H), 7.46 (t, J = 7.7 Hz, 2H), 4.13−4.01 (m, 2H), 3.39 (d, J = 16.3 Hz, 1H), 3.00 (d, J = 16.3 Hz, 1H), 2.78−2.59 (m, 2H), 2.54−2.39 (m, 2H), 2.21−2.12 (m, 2H), 1.95 (t, J = 2.5 Hz, 1H), 1.64−1.45 (m, 4H), 1.44−1.34 (m, 2H).

^{13}C NMR (100 MHz, $CDCl_3$) δ (ppm) = 199.2, 175.3, 168.5, 134.5, 133.2, 129.9, 128.5, 88.8, 84.1, 68.4, 65.2, 43.3, 31.0, 27.8, 27.8, 27.7, 24.8, 18.2.

HRMS (ESI) for $C_{20}H_{22}O_5Na$ $[M+Na]^+$ calcd. 365.1359, found 365.1363.

ethyl (S)-6-(2-(2-benzoyl-5-oxotetrahydrofuran-2-yl)acetoxy)hexanoate (3ad)

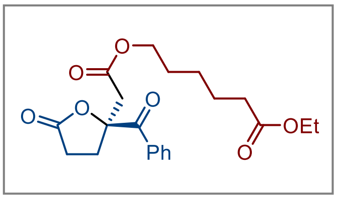

Purification by flash chromatography (PE/EA = 4/1) afforded **3ad**. White solid; m.p. = 55−57 °C; 32.8 mg, 84% yield; Chiral HPLC conditions: ee = 96%; Chiralpak OD-H 95:5 Hexane/iPrOH, 1.0 mL/min, 80 min. t_R (minor) = 45.0 min. and t_R (major) = 66.4 min. λ = 245 nm.

$[\alpha]_D^{25}$ = +17.4 (c = 0.1, $CHCl_3$).

^{1}H NMR (400 MHz, $CDCl_3$) δ (ppm) = 8.02 (d, J = 7.5 Hz, 2H), 7.57 (t, J = 7.4 Hz, 1H), 7.46 (t, J = 7.7 Hz, 2H), 4.18−3.98 (m, 4H), 3.38 (d, J = 16.3 Hz, 1H), 3.00 (d, J = 16.3 Hz, 1H), 2.78−2.59 (m, 2H), 2.54−2.37 (m, 2H), 2.27 (t, J = 7.5 Hz, 2H), 1.66−1.52 (m, 4H), 1.35−1.22 (m, 5H).

^{13}C NMR (100 MHz, $CDCl_3$) δ (ppm) = 199.2, 175.3, 173.4, 168.5, 134.6, 133.2, 129.9, 128.5, 88.8, 65.1, 60.2, 43.3, 34.0, 31.0, 28.0, 27.7, 25.3, 24.4, 14.2.

HRMS (ESI) for $C_{21}H_{26}O_7Na$ $[M+Na]^+$ calcd. 413.1571, found 413.1571.

12-bromododecyl (S)-2-(2-benzoyl-5-oxotetrahydrofuran-2-yl)acetate (3ae)

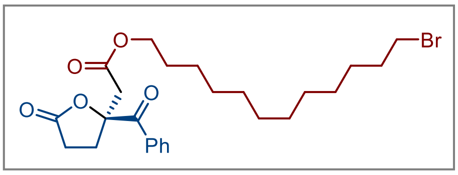

Purification by flash chromatography (PE/EA = 4/1) afforded **3ae**. White solid; m.p. = 68−70 °C; 42.5 mg, 86% yield; Chiral HPLC conditions: ee = 95%; Chiralpak OD-H 95:5 Hexane/iPrOH, 1.0 mL/min, 30 min. t_R (minor) = 19.3 min. and t_R (major) = 23.9 min. λ = 245 nm.

[α]$_D^{25}$ = +23.5 (c = 0.1, $CHCl_3$).

^{1}H NMR (400 MHz, $CDCl_3$) δ (ppm) = 8.02 (d, *J* = 7.4 Hz, 2H), 7.57 (t, *J* = 7.4 Hz, 1H), 7.46 (t, *J* = 7.7 Hz, 2H), 4.11−3.99 (m, 2H), 3.46−3.32 (m, 3H), 2.99 (d, *J* = 16.3 Hz, 1H), 2.78−2.58 (m, 2H), 2.54−2.38 (m, 2H), 1.91−1.78 (m, 2H), 1.60−1.50 (m, 2H), 1.46−1.37 (m, 2H), 1.34−1.20 (m, 14H).

^{13}C NMR (100 MHz, $CDCl_3$) δ (ppm) = 199.2, 175.3, 168.5, 134.6, 133.1, 129.9, 128.4, 88.8, 65.5, 43.3, 34.0, 32.8, 31.0, 29.4, 29.3, 29.1, 28.7, 28.3, 28.1, 27.8, 25.7.

HRMS (ESI) for $C_{25}H_{35}BrO_5Na$ $[M+Na]^+$ calcd. 517.1560, found 517.1567.

isopropyl (S)-2-(2-benzoyl-5-oxotetrahydrofuran-2-yl)acetate (3af)

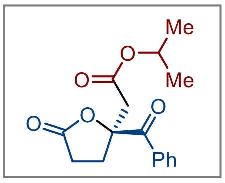

Purification by flash chromatography (PE/EA = 4/1) afforded **3af**. White solid; m.p. = 87−89 °C; 26.4 mg, 91% yield; Chiral HPLC conditions: ee = 95%; Chiralpak AY-H 95:5 Hexane/iPrOH, 1.0 mL/min, 60 min. t_R (minor) = 40.1 min. and t_R (major) = 45.4 min. λ = 245 nm.

[α]$_D^{25}$ = +27.1 (c = 0.1, $CHCl_3$).

^{1}H NMR (400 MHz, $CDCl_3$) δ (ppm) = 8.07−7.99 (m, 2H), 7.57 (t, *J* = 7.4 Hz, 1H), 7.46 (t, *J* = 7.7 Hz, 2H), 5.04−4.94 (m, 1H), 3.33 (d, *J* = 16.2 Hz, 1H), 2.97 (d, *J* = 16.2 Hz, 1H), 2.78−2.58 (m, 2H), 2.53−2.40 (m, 2H), 1.19 (d, *J* = 6.3 Hz, 6H).

^{13}C NMR (100 MHz, $CDCl_3$) δ (ppm) = 199.2, 175.4, 167.9, 134.6, 133.1, 129.9, 128.5, 88.8, 69.1, 43.6, 30.9, 27.8, 21.6.

HRMS (ESI) for $C_{16}H_{18}O_5Na$ $[M+Na]^+$ calcd. 313.1046, found 313.1048.

cyclopropyl (S)-2-(2-benzoyl-5-oxotetrahydrofuran-2-yl)acetate (3ag)

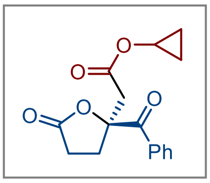

Purification by flash chromatography (PE/EA = 4/1) afforded **3ag**. White solid; m.p. = 107−109 °C; 22.5 mg, 78% yield; Chiral HPLC conditions: ee = 96%; Chiralpak OD-H 95:5 Hexane/iPrOH, 1.0 mL/min, 50 min. t_R (minor) = 28.6 min. and t_R (major) =37.0 min. λ = 245 nm.

[α]$_D$^{25} = +33.3 (c = 0.1, $CHCl_3$).

^{1}H NMR (400 MHz, $CDCl_3$) δ (ppm) = 8.01 (d, J = 7.4 Hz, 2H), 7.57 (t, J = 7.4 Hz, 1H), 7.46 (t, J = 7.7 Hz, 2H), 4.16−4.07 (m, 1H), 3.34 (d, J = 16.4 Hz, 1H), 2.96 (d, J = 16.4 Hz, 1H), 2.76−2.59 (m, 2H), 2.53−2.40 (m, 2H), 0.72−0.60 (m, 4H).

^{13}C NMR (100 MHz, $CDCl_3$) δ (ppm) = 199.2, 175.2, 169.3, 134.5, 133.2, 129.9, 128.5, 88.7, 49.7, 43.1, 30.9, 27.7, 4.9, 4.9.

HRMS (ESI) for $C_{16}H_{16}O_5Na$ $[M+Na]^+$ calcd. 311.0890, found 311.0892.

cyclobutyl (S)-2-(2-benzoyl-5-oxotetrahydrofuran-2-yl)acetate (3ah)

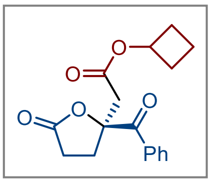

Purification by flash chromatography (PE/EA = 4/1) afforded **3ah**. White solid; m.p. = 96−98 °C; 22.7 mg, 75% yield; Chiral HPLC conditions: ee = 95%; Chiralpak OD-H 95:5 Hexane/iPrOH, 1.0 mL/min, 30 min. t_R (minor) = 20.9 min. and t_R (major) = 23.1 min. λ = 245 nm.

[α]$_D$^{25} = +27.5 (c = 0.1, $CHCl_3$).

^{1}H NMR (400 MHz, $CDCl_3$) δ (ppm) = 8.02 (d, J = 7.6 Hz, 2H), 7.57 (t, J = 7.4 Hz, 1H), 7.46 (t, J = 7.7 Hz, 2H), 5.03−4.88 (m, 1H), 3.34 (d, J = 16.3 Hz, 1H), 2.96 (d, J = 16.3 Hz, 1H), 2.78−2.58 (m, 2H), 2.53−2.38 (m, 2H), 2.36−2.21 (m, 2H), 2.07−1.92 (m, 2H), 1.82−1.70 (m, 1H), 1.65−1.51 (m, 1H).

^{13}C NMR (100 MHz, $CDCl_3$) δ (ppm) = 199.2, 175.3, 167.7, 134.6, 133.2, 129.9, 128.4, 88.8, 69.6, 43.2, 30.9, 30.1, 30.0, 27.7, 13.4.

HRMS (ESI) for $C_{17}H_{18}O_5Na$ $[M+Na]^+$ calcd. 325.1046, found 325.1049.

cyclopentyl (S)-2-(2-benzoyl-5-oxotetrahydrofuran-2-yl)acetate (3ai)

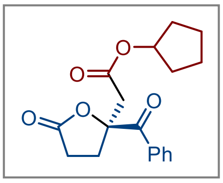

Purification by flash chromatography (PE/EA = 4/1) afforded **3ai**. White solid; m.p. = 79−81 °C; 23.7 mg, 75% yield; Chiral HPLC conditions: ee = 95%; Chiralpak IA 95:5 Hexane/iPrOH, 1.0 mL/min, 30 min. t_R (minor) = 16.2 min. and t_R (major) = 17.4 min. λ = 245 nm.

[α]$_D$^{25} = +13.2 (c = 0.1, $CHCl_3$).

^{1}H NMR (400 MHz, $CDCl_3$) δ (ppm) = 8.09−7.95 (m, 2H), 7.57 (t, *J* = 7.4 Hz, 1H), 7.46 (t, *J* = 7.7 Hz, 2H), 5.21−5.10 (m, 1H), 3.32 (d, *J* = 16.2 Hz, 1H), 2.98 (d, *J* = 16.2 Hz, 1H), 2.80−2.59 (m, 2H), 2.53−2.38 (m, 2H), 1.88−1.71 (m, 2H), 1.67−1.51 (m, 6H).

^{13}C NMR (100 MHz, $CDCl_3$) δ (ppm) = 198.9, 175.3, 168.2, 134.5, 133.2, 129.9, 128.5, 88.8, 78.4, 43.6, 32.5, 32.5, 30.9, 27.8, 23.6.

HRMS (ESI) for $C_{18}H_{20}O_5Na$ $[M+Na]^+$ calcd. 339.1203, found 339.1206.

cyclohexyl (S)-2-(2-benzoyl-5-oxotetrahydrofuran-2-yl)acetate (3aj)

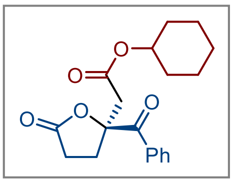

Purification by flash chromatography (PE/EA = 4/1) afforded **3aj**. White solid; m.p. = 105−107 °C; 27.4 mg, 83% yield; Chiral HPLC conditions: ee = 96%; Chiralpak ID 95:5 Hexane/iPrOH, 1.0 mL/min, 80 min. t_R (minor) = 46.7 min. and t_R (major) = 51.7 min. λ = 245 nm.

[α]$_D$^{25} = +12.6 (c = 0.1, $CHCl_3$).

^{1}H NMR (400 MHz, $CDCl_3$) δ (ppm) = 8.03 (d, *J* = 7.6 Hz, 2H), 7.57 (t, *J* = 7.4 Hz, 1H), 7.46 (t, *J* = 7.6 Hz, 2H), 4.87−4.66 (m, 1H), 3.34 (d, *J* = 16.2 Hz, 1H), 3.00 (d, *J* = 16.2 Hz, 1H), 2.81−2.70 (m, 1H), 2.69−2.59 (m, 1H), 2.54−2.38 (m, 2H), 1.85−1.75 (m, 2H), 1.72−1.63 (m, 2H), 1.55−1.47 (m, 1H), 1.40−1.18 (m, 5H).

^{13}C NMR (100 MHz, $CDCl_3$) δ (ppm) = 199.0, 175.4, 167.9, 134.5, 133.1, 129.9, 128.4, 88.8, 74.1, 43.6, 31.4, 30.8, 27.8, 25.1, 23.6.

HRMS (ESI) for $C_{19}H_{22}O_5Na$ $[M+Na]^+$ calcd. 353.1359, found 353.1361.

tert-butyl (S)-4-(2-(2-benzoyl-5-oxotetrahydrofuran-2-yl)acetoxy)piperidine-1-carboxylate (3ak)

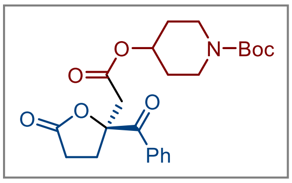

Purification by flash chromatography (PE/EA = 2/1) afforded **3ak**. White solid; m.p. = 71−73 °C; 33.2 mg, 77% yield; Chiral HPLC conditions: ee = 96%; Chiralpak OD-H 95:5 Hexane/iPrOH, 1.0 mL/min, 80 min. t_R (minor) = 47.7 min. and t_R (major) = 61.3 min. λ = 245 nm.

$[\alpha]_D^{25}$ = +9.6 (c = 0.1, $CHCl_3$).

^{1}H NMR (400 MHz, $CDCl_3$) δ (ppm) = 8.02 (d, *J* = 7.6 Hz, 2H), 7.58 (t, *J* = 7.3 Hz, 1H), 7.46 (t, *J* = 7.6 Hz, 2H), 4.97−4.86 (m, 1H), 3.72−3.58 (m, 2H), 3.38 (d, *J* = 16.1 Hz, 1H), 3.20−3.10 (m, 2H), 3.01 (d, *J* = 16.1 Hz, 1H), 2.78−2.60 (m, 2H), 2.53−2.40 (m, 2H), 1.85−1.75 (m, 2H), 1.58−1.49 (m, 2H), 1.45 (s, 9H).

^{13}C NMR (100 MHz, $CDCl_3$) δ (ppm) = 199.0, 175.2, 167.8, 154.6, 134.5, 133.3, 129.9, 128.6, 88.8, 79.7, 71.3, 43.6, 40.8, 31.0, 30.4, 28.4, 27.7.

HRMS (ESI) for $C_{23}H_{29}O_7NNa$ $[M+Na]^+$ calcd. 454.1836, found 454.1839.

cyclododecyl (S)-2-(2-benzoyl-5-oxotetrahydrofuran-2-yl)acetate (3al)

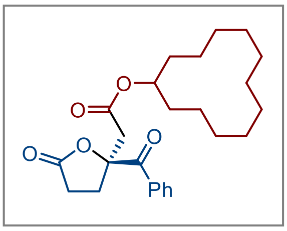

Purification by flash chromatography (PE/EA = 4/1) afforded **3al**. White solid; m.p. = 101−103 °C; 31.9 mg, 77% yield; Chiral HPLC conditions: ee = 95%; Chiralpak ID 95:5 Hexane/iPrOH, 1.0 mL/min, 40 min. t_R (minor) = 28.2 min. and t_R (major) = 34.3 min. λ = 246 nm.

$[\alpha]_D^{25}$ = +13.5 (c = 0.1, $CHCl_3$).

^{1}H NMR (400 MHz, $CDCl_3$) δ (ppm) = 8.03 (d, *J* = 7.6 Hz, 2H), 7.57 (t, *J* = 7.4 Hz, 1H), 7.46 (t, *J* = 7.6 Hz, 2H), 5.05−4.91 (m, 1H), 3.33 (d, *J* = 16.1 Hz, 1H), 2.99 (d, *J* = 16.1 Hz, 1H), 2.82−2.71 (m, 1H), 2.70−2.59 (m, 1H), 2.53−2.39 (m, 2H), 1.69−1.58 (m, 2H), 1.49−1.22 (m, 20H).

^{13}C NMR (100 MHz, $CDCl_3$) δ (ppm) = 198.9, 175.4, 168.0, 134.5, 133.2, 130.0, 128.5, 88.9, 73.9, 43.6, 30.8, 28.8, 28.7, 27.8, 24.0, 23.9, 23.2, 23.2, 23.1, 23.0, 20.7, 20.7.

HRMS (ESI) for $C_{25}H_{34}O_5Na$ $[M+Na]^+$ calcd. 437.2298, found 437.2308.

(1S,3S,5S,7S)-adamantan-2-yl 2-((S)-2-benzoyl-5-oxotetrahydrofuran-2-yl)acetate (3am)

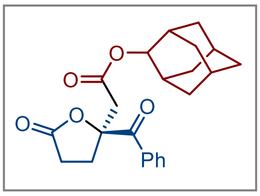

Purification by flash chromatography (PE/EA = 4/1) afforded **3am**. Colorless oil; 31.7 mg, 83% yield; Chiral HPLC conditions: ee = 96%; Chiralpak OD-H 95:5 Hexane/iPrOH, 1.0 mL/min, 40 min. t_R (minor) = 15.2 min. and t_R (major) = 17.6 min. λ = 245 nm.

[α]$_D^{25}$ = +9.0 (c = 0.1, $CHCl_3$).

^{1}H NMR (400 MHz, $CDCl_3$) δ (ppm) = 8.04 (d, *J* = 7.4 Hz, 2H), 7.57 (t, *J* = 7.4 Hz, 1H), 7.46 (t, *J* = 7.6 Hz, 2H), 4.93 (s, 1H), 3.40 (d, *J* = 16.2 Hz, 1H), 3.06 (d, *J* = 16.2 Hz, 1H), 2.82–2.60 (m, 2H), 2.54–2.41 (m, 2H), 1.99–1.77 (m, 8H), 1.76–1.67 (m, 4H), 1.56–1.47 (m, 2H).

^{13}C NMR (100 MHz, $CDCl_3$) δ (ppm) = 198.9, 175.4, 167.9, 134.5, 133.2, 129.9, 128.5, 88.8, 78.5, 43.7, 37.2, 36.2, 36.2, 31.7, 31.6, 31.6, 31.6, 30.8, 27.8, 27.0, 26.8.

HRMS (ESI) for $C_{23}H_{26}O_5Na$ $[M+Na]^+$ calcd. 405.1672, found 405.1677.

(3R,5R,7R)-adamantan-1-yl 2-((S)-2-benzoyl-5-oxotetrahydrofuran-2-yl)acetate (3an)

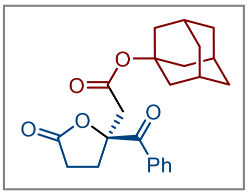

Purification by flash chromatography (PE/EA = 4/1) afforded **3an**. Colorless oil; 22.5 mg, 59% yield; Chiral HPLC conditions: ee = 95%; Chiralpak IK 95:5 Hexane/iPrOH, 1.0 mL/min, 40 min. t_R (minor) = 26.6 min. and t_R (major) = 30.4 min. λ = 245 nm.

[α]$_D^{25}$ = +4.7 (c = 0.1, $CHCl_3$).

^{1}H NMR (400 MHz, $CDCl_3$) δ (ppm) = 8.07–7.98 (m, 2H), 7.57 (t, *J* = 7.4 Hz, 1H), 7.46 (t, *J* = 7.6 Hz, 2H), 3.25 (d, *J* = 15.8 Hz, 1H), 2.95 (d, *J* = 15.8 Hz, 1H), 2.84–2.72 (m, 1H), 2.70–2.58 (m, 1H), 2.54–2.37 (m, 2H), 2.13 (s, 3H), 2.07–2.00 (m, 6H), 1.62 (m, 6H).

^{13}C NMR (100 MHz, $CDCl_3$) δ (ppm) = 198.6, 175.5, 167.2, 134.5, 133.1, 130.0, 128.4, 88.9, 82.5, 44.7, 41.1, 36.0, 30.7, 30.5, 27.9.

HRMS (ESI) for $C_{23}H_{26}O_5Na$ $[M+Na]^+$ calcd. 405.1672, found 405.1677.

(S)-5-(((3S,5S,7S)-adamantan-1-yl)methyl)-5-benzoyldihydrofuran-2(3H)-one (3an')

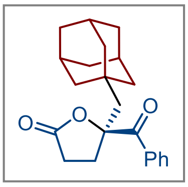

Purification by flash chromatography (PE/EA = 4/1) afforded **3an'**. Colorless oil; 5.8 mg, 17% yield; Chiral HPLC conditions: ee = 89%; Chiralpak OX-H 99:1 Hexane/iPrOH, 0.9 mL/min, 50 min. t_R (minor) = 34.0 min. and t_R (major) = 43.0 min. λ = 246 nm.

$[\alpha]_D^{25}$ = -5.1 (c = 0.2, $CHCl_3$)

^{1}H NMR (400 MHz, $CDCl_3$) δ (ppm) = 8.15−8.00 (m, 2H), 7.61−7.52 (m, 1H), 7.46 (t, J = 7.6 Hz, 2H), 2.77−2.67 (m, 1H), 2.61−2.49 (m, 1H), 2.47−2.36 (m, 1H), 2.34−2.22 (m, 2H), 1.93−1.81 (m, 4H), 1.68−1.53 (m, 9H), 1.52−1.47 (m, 3H),

^{13}C NMR (100 MHz, $CDCl_3$) δ (ppm) = 200.0, 175.7, 134.8, 133.1, 130.2, 128.4, 92.6, 52.7, 43.2, 36.5, 34.3, 33.4, 28.4, 27.7.

HRMS (ESI) for $C_{22}H_{26}O_3Na$ $[M+Na]^+$ calcd. 361.1774, found 361.1787.

(1S,1's,4R,4'S)-4'-propyl-[1,1'-bi(cyclohexan)]-4-yl 2-((S)-2-benzoyl-5-oxotetrahydrofuran-2-yl)acetate (3ao)

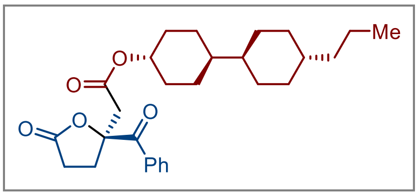

Purification by flash chromatography (PE/EA = 4/1) afforded **3ao**. White solid; m.p. = 127−129 °C; 37.3 mg, 82% yield; Chiral HPLC conditions: ee = 95%; Chiralpak OD-H 95:5 Hexane/iPrOH, 1.0 mL/min, 60 min. t_R (minor) = 17.9 min. and t_R (major) = 19.7 min. λ = 245 nm.

$[\alpha]_D^{25}$ = +27.2 (c = 0.1, $CHCl_3$).

^{1}H NMR (400 MHz, $CDCl_3$) δ (ppm) = 8.06−7.99 (m, 2H), 7.56 (t, J = 7.4 Hz, 1H), 7.45 (t, J = 7.8 Hz, 2H), 4.69−4.55 (m, 1H), 3.33 (d, J = 16.2 Hz, 1H), 2.98 (d, J = 16.2 Hz, 1H), 2.79−2.69 (m, 1H), 2.68−2.58 (m, 1H), 2.52−2.38 (m, 2H), 1.98−1.89 (m, 2H), 1.77−1.70 (m, 4H), 1.69−1.62 (m, 2H), 1.34−1.19 (m, 4H), 1.16−1.09 (m, 3H), 1.06−0.90 (m, 6H), 0.89−0.78 (m, 5H).

^{13}C NMR (100 MHz, $CDCl_3$) δ (ppm) = 199.1, 175.4, 168.0, 134.6, 133.1, 129.9, 128.4,

88.8, 75.1, 43.6, 42.6, 42.0, 39.7, 37.4, 33.4, 31.6, 30.8, 30.1, 27.8, 27.7, 20.0, 14.4.

HRMS (ESI) for $C_{28}H_{38}O_5Na$ $[M+Na]^+$ calcd. 477.2611, found 477.2616.

hexadecyl (S)-2-(2-benzoyl-5-oxotetrahydrofuran-2-yl)acetate (3ap)

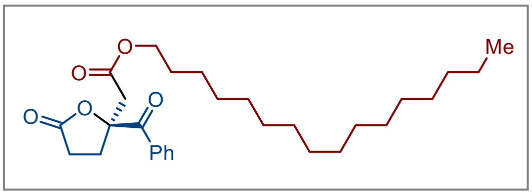

Purification by flash chromatography (PE/EA = 4/1) afforded **3ap**. White solid; m.p. = 76–78 °C; 38.3 mg, 81% yield; Chiral HPLC conditions: ee = 94%; Chiralpak OD-H 95:5 Hexane/iPrOH, 1.0 mL/min, 30 min. t_R (minor) = 11.2 min. and t_R (major) = 13.3 min. λ = 245 nm.

$[\alpha]_D^{25}$ = +24.7 (c = 0.1, $CHCl_3$).

^{1}H NMR (400 MHz, $CDCl_3$) δ (ppm) = 8.02 (d, *J* = 7.6 Hz, 2H), 7.57 (t, *J* = 7.1 Hz, 1H), 7.46 (t, *J* = 7.4 Hz, 2H), 4.14–3.99 (m, 2H), 3.38 (d, *J* = 16.3 Hz, 1H), 3.00 (d, *J* = 16.3 Hz, 1H), 2.79–2.59 (m, 2H), 2.54–2.38 (m, 2H), 1.60–1.48 (m, 2H), 1.35–1.16 (m, 26H), 0.88 (t, *J* = 6.1 Hz, 3H).

^{13}C NMR (100 MHz, $CDCl_3$) δ (ppm) = 199.3, 175.3, 168.5, 134.6, 133.2, 129.9, 128.5, 88.8, 65.6, 43.3, 31.9, 31.0, 29.7, 29.6, 29.5, 29.4, 29.3, 29.1, 28.3, 27.8, 25.7, 22.7, 14.1.

HRMS (ESI) for $C_{29}H_{44}O_5Na$ $[M+Na]^+$ calcd. 495.3081, found 495.3084.

(S)-3,7-dimethyloct-6-en-1-yl 2-((S)-2-benzoyl-5-oxotetrahydrofuran-2-yl)acetate (3aq)

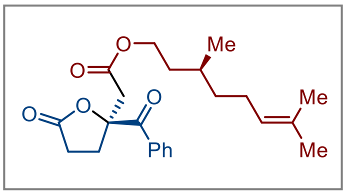

Purification by flash chromatography (PE/EA = 4/1) afforded **3aq**. White solid; m.p. = 72–74 °C; 27.0 mg, 70% yield; Chiral HPLC conditions: de = 95%; Chiralpak OD-H 95:5 Hexane/iPrOH, 1.0 mL/min, 30 min. t_R (minor) = 15.1 min. and t_R (major) = 21.3 min. λ = 245 nm.

$[\alpha]_D^{25}$ = +17.6 (c = 0.1, $CHCl_3$).

^{1}H NMR (400 MHz, $CDCl_3$) δ (ppm) = 8.08–7.97 (m, 2H), 7.57 (t, *J* = 7.4 Hz, 1H),

7.46 (t, J = 7.7 Hz, 2H), 5.06 (t, J = 7.0 Hz, 1H), 4.10 (t, J = 7.0 Hz, 2H), 3.37 (d, J = 16.3 Hz, 1H), 2.99 (d, J = 16.3 Hz, 1H), 2.79−2.60 (m, 2H), 2.53−2.40 (m, 2H), 2.01−1.85 (m, 2H), 1.68 (s, 3H), 1.64−1.55 (m, 4H), 1.50−1.30 (m, 3H), 1.18−1.08 (m, 1H), 0.87 (d, J = 6.5 Hz, 3H).

^{13}C NMR (100 MHz, $CDCl_3$) δ (ppm) = 199.2, 175.3, 168.5, 134.6, 133.2, 131.4, 129.9, 128.5, 124.5, 88.8, 64.0, 43.3, 36.9, 35.1, 31.0, 29.3, 27.8, 25.7, 25.3, 19.2, 17.6.

HRMS (ESI) for $C_{23}H_{30}O_5Na$ $[M+Na]^+$ calcd. 409.1985, found 409.1987.

(2S,4R)-1,7,7-trimethylbicyclo[2.2.1]heptan-2-yl 2-((S)-2-benzoyl-5-oxotetrahydrofuran-2-yl)acetate (3ar)

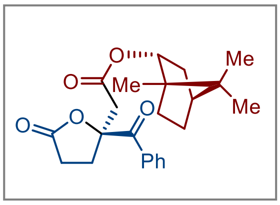

Purification by flash chromatography (PE/EA = 4/1) afforded **3ar**. Colorless oil; 36.5 mg, 95% yield; Chiral HPLC conditions: de = 95%; Chiralpak AS-H 95:5 Hexane/iPrOH, 1.0 mL/min, 30 min. t_R (minor) = 13.1 min. and t_R (major) = 14.8 min. λ = 247 nm.

$[\alpha]_D^{25}$ = -8.6 (c = 0.1, $CHCl_3$).

^{1}H NMR (400 MHz, $CDCl_3$) δ (ppm) = 8.10−8.00 (m, 2H), 7.57 (t, J = 7.4 Hz, 1H), 7.46 (t, J = 7.7 Hz, 2H), 4.94−4.82 (m, 1H), 3.36 (d, J = 16.0 Hz, 1H), 3.07 (d, J = 16.0 Hz, 1H), 2.85−2.75 (m, 1H), 2.71−2.60 (m, 1H), 2.55−2.42 (m, 2H), 2.38−2.27 (m, 1H), 1.81−1.64 (m, 3H), 1.26−1.12 (m, 2H), 0.94 (dd, J = 13.8, 3.4 Hz, 1H), 0.86 (s, 3H), 0.85 (s, 3H), 0.77 (s, 3H).

^{13}C NMR (100 MHz, $CDCl_3$) δ (ppm) = 198.6, 175.3, 168.8, 134.4, 133.3, 130.0, 128.5, 88.8, 81.4, 48.7, 47.8, 44.7, 43.5, 36.5, 30.7, 27.9, 27.8, 27.0, 19.6, 18.7, 13.4.

HRMS (ESI) for $C_{23}H_{28}O_5Na$ $[M+Na]^+$ calcd. 407.1829, found 407.1833.

(1R,2R,4S)-1,3,3-trimethylbicyclo[2.2.1]heptan-2-yl 2-((S)-2-benzoyl-5-oxotetrahydrofuran-2-yl)acetate (3as)

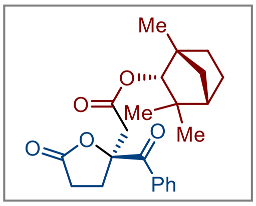

Purification by flash chromatography (PE/EA = 4/1) afforded **3as**. Colorless oil; 32.3 mg, 84% yield; Chiral HPLC conditions: de = 97%; Chiralpak IA 95:5 Hexane/iPrOH, 1.0 mL/min, 30 min. t_R (minor) = 9.8 min. and t_R (major) = 11.1 min. λ = 245 nm.

$[\alpha]_D^{25}$ = +24.5 (c = 0.1, $CHCl_3$).

^{1}H NMR (400 MHz, $CDCl_3$) δ (ppm) = 8.03 (d, J = 7.6 Hz, 2H), 7.57 (t, J = 7.4 Hz, 1H), 7.46 (t, J = 7.7 Hz, 2H), 4.36 (d, J = 1.6 Hz, 1H), 3.37 (d, J = 16.3 Hz, 1H), 3.10 (d, J = 16.3 Hz, 1H), 2.85−2.74 (m, 1H), 2.72−2.61 (m, 1H), 2.54−2.41 (m, 2H), 1.71−1.51 (m, 4H), 1.48−1.38 (m, 1H), 1.17 (d, J = 10.2 Hz, 1H), 1.08−0.97 (m, 7H), 0.72 (s, 3H).

^{13}C NMR (100 MHz, $CDCl_3$) δ (ppm) = 198.6, 175.3, 168.9, 134.4, 133.1, 129.9, 128.5, 88.6, 87.5, 48.1, 48.1, 43.1, 41.3, 39.3, 30.6, 29.5, 27.8, 26.4, 25.7, 20.1, 19.2.

HRMS (ESI) for $C_{23}H_{28}O_5Na$ $[M+Na]^+$ calcd. 407.1829, found 407.1832.

(1R,2S,5R)-2-isopropyl-5-methylcyclohexyl 2-((S)-2-benzoyl-5-oxotetrahydrofuran-2-yl)acetate (3at)

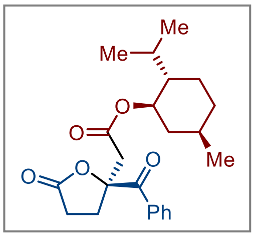

Purification by flash chromatography (PE/EA = 4/1) afforded **3at**. White solid; m.p. = 139−141 °C; 34.0 mg, 88% yield; Chiral HPLC conditions: de = 96%; Chiralpak IA 95:5 Hexane/iPrOH, 1.0 mL/min, 30 min. t_R (minor) = 10.0 min. and t_R (major) = 11.9 min. λ = 245 nm.

$[\alpha]_D^{25}$ = -25.9 (c = 0.1, $CHCl_3$).

^{1}H NMR (400 MHz, $CDCl_3$) δ (ppm) = 8.02 (d, J = 7.4 Hz, 2H), 7.57 (t, J = 7.4 Hz, 1H), 7.46 (t, J = 7.6 Hz, 2H), 4.67 (td, J = 10.9, 4.3 Hz, 1H), 3.33 (d, J = 16.2 Hz, 1H), 3.02 (d, J = 16.2 Hz, 1H), 2.83−2.72 (m, 1H), 2.70−2.58 (m, 1H), 2.54−2.38 (m, 2H), 2.00−1.90 (m, 1H), 1.82−1.71 (m, 1H), 1.69−1.60 (m, 2H), 1.49−1.38 (m, 1H), 1.34−1.25 (m, 1H), 1.06−0.81 (m, 9H), 0.69 (d, J = 7.0 Hz, 3H).

^{13}C NMR (100 MHz, $CDCl_3$) δ (ppm) = 198.9, 175.3, 168.0, 134.6, 133.1, 129.8, 128.5, 88.7, 75.6, 46.7, 43.6, 40.6, 34.0, 31.3, 30.7, 27.8, 26.1, 23.2, 21.9, 20.7, 16.1.

HRMS (ESI) for $C_{23}H_{30}O_5Na$ $[M+Na]^+$ calcd. 409.1985, found 409.1989.

(1S,2R,5S)-2-isopropyl-5-methylcyclohexyl 2-((S)-2-benzoyl-5-oxotetrahydrofuran-2-yl)acetate (3au)

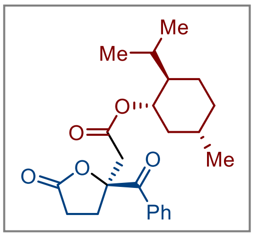

Purification by flash chromatography (PE/EA = 4/1) afforded **3au**. White solid; m.p. = 114−116 °C; 29.0 mg, 75% yield; Chiral HPLC conditions: de = 96%; Chiralpak IA 95:5 Hexane/iPrOH, 1.0 mL/min, 15 min. t_R (minor) = 7.1 min. and t_R (major) = 8.2 min. λ = 245 nm.

$[\alpha]_D^{25}$ = -26.4 (c = 0.1, $CHCl_3$).

^{1}H NMR (400 MHz, $CDCl_3$) δ (ppm) = 8.06−7.98 (m, 2H), 7.57 (t, J = 7.4 Hz, 1H), 7.46 (t, J = 7.6 Hz, 2H), 4.67 (td, J = 10.9, 4.3 Hz, 1H), 3.33 (d, J = 16.2 Hz, 1H), 3.02 (d, J = 16.2 Hz, 1H), 2.82−2.72 (m, 1H), 2.69−2.59 (m, 1H), 2.53−2.38 (m, 2H), 1.99−1.91 (m, 1H), 1.81−1.73 (m, 1H), 1.68−1.60 (m, 2H), 1.49−1.38 (m, 1H), 1.34−1.26 (m, 1H), 1.06−0.81 (m, 9H), 0.69 (d, J = 7.0 Hz, 3H)

^{13}C NMR (100 MHz, $CDCl_3$) δ (ppm) = 198.9, 175.3, 168.0, 134.6, 133.1, 129.8, 128.5, 88.7, 75.6, 46.7, 43.6, 40.6, 34.0, 31.3, 30.8, 27.8, 26.1, 23.2, 21.9, 20.7, 16.1.

HRMS (ESI) for $C_{23}H_{30}O_5Na$ $[M+Na]^+$ calcd. 409.1985, found 409.1981.

((3aR,5R,5aS,8aS,8bR)-2,2,7,7-tetramethyltetrahydro-5H-bis([1,3]dioxolo)[4,5-b:4',5'-d]pyran-5-yl)methyl 2-((S)-2-benzoyl-5-oxotetrahydrofuran-2-yl)acetate (3av)

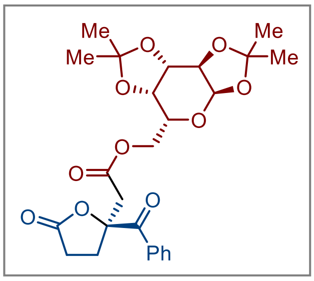

Purification by flash chromatography (PE/EA = 2/1) afforded **3av**. Colorless oil; 36.8 mg, 75% yield; Chiral HPLC conditions: de = 98%; Chiralpak IK 95:5 Hexane/iPrOH, 1.0 mL/min, 100 min. t_R (minor) = 64.6 min. and t_R (major) = 89.3 min. λ = 245 nm.

$[\alpha]_D^{25}$ = -25.9 (c = 0.1, $CHCl_3$).

^{1}H NMR (400 MHz, $CDCl_3$) δ (ppm) = 8.01 (d, J = 7.4 Hz, 2H), 7.57 (t, J = 7.4 Hz, 1H), 7.46 (t, J = 7.6 Hz, 2H), 5.52 (d, J = 4.9 Hz, 1H), 4.59 (dd, J = 7.8, 1.9 Hz, 1H),

4.34–4.19 (m, 3H), 4.14 (d, J = 7.8 Hz, 1H), 3.98 (t, J = 5.4 Hz, 1H), 3.39 (d, J = 16.4 Hz, 1H), 3.07 (d, J = 16.4 Hz, 1H), 2.81–2.63 (m, 2H), 2.53–2.40 (m, 2H), 1.51 (s, 3H), 1.43 (s, 3H), 1.33 (s, 3H), 1.31 (s, 3H).

^{13}C NMR (100 MHz, $CDCl_3$) δ (ppm) = 198.9, 175.3, 168.4, 134.5, 133.2, 129.9, 128.5, 109.6, 108.8, 96.2, 88.6, 70.8, 70.6, 70.3, 65.7, 64.2, 43.0, 30.6, 27.8, 26.0, 25.9, 24.9, 24.4.

HRMS (ESI) for $C_{25}H_{30}O_{10}Na$ $[M+Na]^+$ calcd. 513.1731, found 513.1736.

((3aS,5aR,8aR,8bS)-2,2,7,7-tetramethyltetrahydro-3aH-bis([1,3]dioxolo)[4,5-b:4',5'-d]pyran-3a-yl)methyl 2-((S)-2-benzoyl-5-oxotetrahydrofuran-2-yl)acetate (3aw)

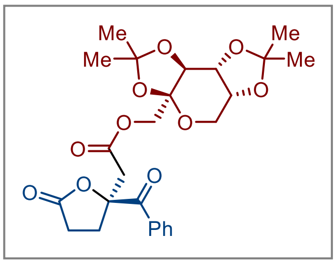

Purification by flash chromatography (PE/EA = 2/1) afforded **3aw**. Colorless oil; 36.3 mg, 74% yield; Chiral HPLC conditions: de = 95%; Chiralpak IA 95:5 Hexane/iPrOH, 1.0 mL/min, 80 min. t_R (minor) = 42.5 min. and t_R (major) = 48.5 min. λ = 245 nm.

$[\alpha]_D^{25}$ = -12.7 (c = 0.1, $CHCl_3$).

^{1}H NMR (400 MHz, $CDCl_3$) δ (ppm) = 8.02 (d, J = 7.4 Hz, 2H), 7.57 (t, J = 7.4 Hz, 1H), 7.46 (t, J = 7.6 Hz, 2H), 4.57 (dd, J = 7.9, 2.5 Hz, 1H), 4.46 (d, J = 11.6 Hz, 1H), 4.25–4.18 (m, 2H), 4.00 (d, J = 11.6 Hz, 1H), 3.88 (dd, J = 13.0, 1.6 Hz, 1H), 3.74 (d, J = 13.0 Hz, 1H), 3.43 (d, J = 16.8 Hz, 1H), 3.04 (d, J = 16.8 Hz, 1H), 2.78–2.62 (m, 2H), 2.53–2.42 (m, 2H), 1.53 (s, 3H), 1.45 (s, 3H), 1.34 (s, 3H), 1.33 (s, 3H).

^{13}C NMR (100 MHz, $CDCl_3$) δ (ppm) = 199.4, 175.3, 168.0, 134.5, 133.2, 129.9, 128.5, 109.1, 108.8, 101.2, 88.6, 70.6, 70.6, 69.9, 66.0, 61.3, 42.7, 30.7, 27.8, 26.4, 25.9, 25.1, 24.0.

HRMS (ESI) for $C_{25}H_{30}O_{10}Na$ $[M+Na]^+$ calcd. 513.1731, found 513.1737.

((3aR,4R,6R,6aR)-6-methoxy-2,2-dimethyltetrahydrofuro[3,4-d][1,3]dioxol-4-yl)methyl 2-((S)-2-benzoyl-5-oxotetrahydrofuran-2-yl)acetate (3ax)

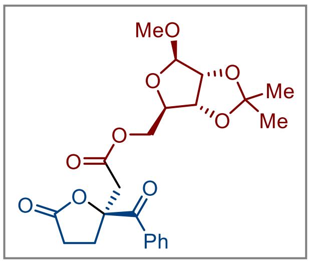

Purification by flash chromatography (PE/EA = 2/1) afforded **3ax**. Colorless oil; 35.6 mg, 82% yield; Chiral HPLC conditions: de = 95%; Chiralpak IA 95:5 Hexane/iPrOH, 1.0 mL/min, 60 min. t_R (minor) = 39.5 min. and t_R (major) = 42.9 min. λ = 245 nm.

[α]$_D$^{25} = -9.5 (c = 0.1, $CHCl_3$).

^{1}H NMR (400 MHz, $CDCl_3$) δ (ppm) = 8.02 (d, *J* = 7.6 Hz, 2H), 7.58 (t, *J* = 7.4 Hz, 1H), 7.46 (t, *J* = 7.6 Hz, 2H), 4.95 (s, 1H), 4.64−4.51 (m, 2H), 4.32 (t, *J* = 6.7 Hz, 1H), 4.20−4.05 (m, 2H), 3.43 (d, *J* = 16.5 Hz, 1H), 3.28 (s, 3H), 3.04 (d, *J* = 16.5 Hz, 1H), 2.80−2.61 (m, 2H), 2.55−2.40 (m, 2H), 1.47 (s, 3H), 1.31 (s, 3H).

^{13}C NMR (100 MHz, $CDCl_3$) δ (ppm) = 199.2, 175.2, 168.2, 134.5, 133.3, 129.9, 128.5, 112.5, 109.4, 88.6, 85.1, 83.8, 81.6, 65.3, 55.0, 43.0, 31.0, 27.8, 26.3, 24.9.

HRMS (ESI) for $C_{22}H_{26}O_9Na$ $[M+Na]^+$ calcd. 457.1469, found 457.1477.

(3S,8S,9S,10R,13R,14S,17R)-10,13-dimethyl-17-((R)-6-methylheptan-2-yl)-2,3,4,7,8,9,10,11,12,13,14,15,16,17-tetradecahydro-1H-cyclopenta[a]phenanthren-3-yl 2-((S)-2-benzoyl-5-oxotetrahydrofuran-2-yl)acetate (3ay)

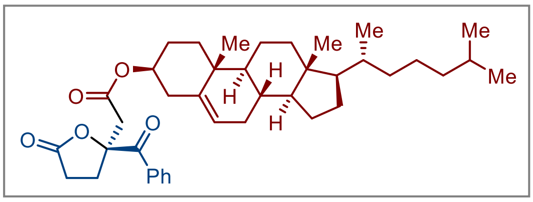

Purification by flash chromatography (PE/EA = 2/1) afforded **3ay**. White solid; m.p. = 126−128 °C; 44.4 mg, 72% yield; Chiral HPLC conditions: de = 95%; Chiralpak IA 95:5 Hexane/iPrOH, 1.0 mL/min, 30 min. t_R (minor) = 11.3 min. and t_R (major) = 12.6 min. λ = 245 nm.

[α]$_D$^{25} = -8.9 (c = 0.1, $CHCl_3$).

^{1}H NMR (400 MHz, $CDCl_3$) δ (ppm) = 8.03 (d, *J* = 7.5 Hz, 2H), 7.56 (t, *J* = 7.4 Hz, 1H), 7.46 (t, *J* = 7.7 Hz, 2H), 5.40−5.29 (m, 1H), 4.67−4.53 (m, 1H), 3.35 (d, *J* = 16.2 Hz, 1H), 2.97 (d, *J* = 16.2 Hz, 1H), 2.78−2.59 (m, 2H), 2.52−2.39 (m, 2H), 2.31−2.17 (m, 2H), 2.04−1.91 (m, 2H), 1.88−1.75 (m, 3H), 1.61−1.26 (m, 11H), 1.19−0.95 (m, 12H), 0.93−0.83 (m, 10H), 0.67 (s, 3H).

^{13}C NMR (100 MHz, $CDCl_3$) δ (ppm) = 199.1, 175.3, 167.8, 139.2, 134.6, 133.2, 129.9, 128.5, 122.9, 88.8, 75.2, 56.6, 56.1, 49.9, 43.6, 42.2, 39.6, 39.5, 37.8, 36.8, 36.5, 36.1, 35.7, 31.8, 31.8, 30.9, 28.2, 28.0, 27.8, 27.5, 24.2, 23.8, 22.8, 22.5, 20.9, 19.2, 18.7, 11.

HRMS (ESI) for $C_{40}H_{56}O_5Na$ $[M+Na]^+$ calcd. 639.4020, found 639.4023.

(3S,5S,8R,9S,10S,13R,14S,17R)-10,13-dimethyl-17-((R)-6-methylheptan-2-yl)hexadecahydro-1H-cyclopenta[a]phenanthren-3-yl 2-((S)-2-benzoyl-5-oxotetrahydrofuran-2-yl)acetate (3az)

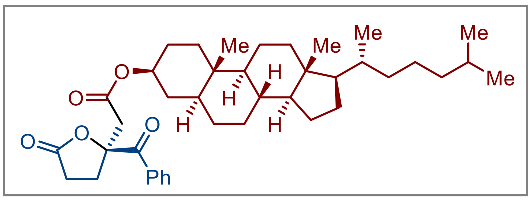

Purification by flash chromatography (PE/EA = 2/1) afforded **3az**. White solid; m.p. = 106−108 °C; 47.6 mg, 77% yield; Chiral HPLC conditions: de = 95%; Chiralpak IA 95:5 Hexane/iPrOH, 1.0 mL/min, 20 min. t_R (minor) = 11.7 min. and t_R (major) = 13.6 min. λ = 245 nm.

$[\alpha]_D^{25}$ = +23.8 (c = 0.1, $CHCl_3$).

^{1}H NMR (400 MHz, $CDCl_3$) δ (ppm) = 8.03 (d, J = 7.6 Hz, 2H), 7.56 (t, J = 7.4 Hz, 1H), 7.45 (t, J = 7.7 Hz, 2H), 4.74−4.61 (m, 1H), 3.34 (d, J = 16.2 Hz, 1H), 2.96 (d, J = 16.2 Hz, 1H), 2.78−2.58 (m, 2H), 2.52−2.38 (m, 2H), 2.00−1.90 (m, 1H), 1.83−1.60 (m, 4H), 1.58−1.39 (m, 5H), 1.39−1.18 (m, 10H), 1.16−1.04 (m, 6H), 1.02−0.94 (m, 3H), 0.91−0.82 (m, 10H), 0.77 (s, 3H), 0.65−0.56 (m, 4H).

^{13}C NMR (100 MHz, $CDCl_3$) δ (ppm) = 199.2, 175.4, 167.9, 134.6, 133.1, 129.9, 128.4, 88.9, 75.1, 56.3, 56.2, 54.1, 44.5, 43.7, 42.5, 39.9, 39.5, 36.6, 36.1, 35.7, 35.4, 35.3, 33.7, 31.9, 30.9, 28.5, 28.2, 28.0, 27.8, 27.2, 24.1, 23.8, 22.8, 22.5, 21.1, 18.6, 12.1, 12.0.

HRMS (ESI) for $C_{40}H_{58}O_5Na$ $[M+Na]^+$ calcd. 641.4176, found 641.4186.

(4S,5'R,6aR,6bS,8aS,8bR,9S,10R,11aS,12aS,12bS)-5',6a,8a,9-tetramethyl-1,3,3',4,4',5,5',6,6a,6b,6',7,8,8a,8b,9,11a,12,12a,12b-icosahydrospiro[naphtho[2',1':4,5]indeno[2,1-b]furan-10,2'-pyran]-4-yl 2-((S)-2-

benzoyl-5-oxotetrahydrofuran-2-yl)acetate (3ba)

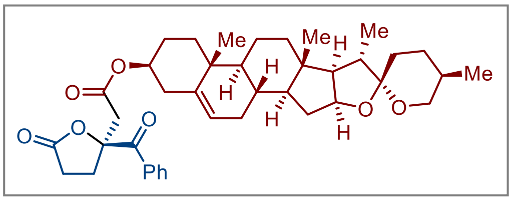

Purification by flash chromatography (PE/EA = 2/1) afforded **3ba**. White solid; m.p. = 139−141 °C; 48.3 mg, 75% yield; Chiral HPLC conditions: de = 94%; Chiralpak IA 97:3 Hexane/iPrOH, 1.0 mL/min, 100 min. t_R (minor) = 28.7 min. and t_R (major) = 31.2 min. λ = 245 nm.

[α]$_D^{25}$ = -56.8 (c = 0.1, $CHCl_3$).

^{1}H NMR (400 MHz, $CDCl_3$) δ (ppm) = 8.03 (d, J = 7.5 Hz, 2H), 7.57 (t, J = 7.4 Hz, 1H), 7.46 (t, J = 7.7 Hz, 2H), 5.41−5.29 (m, 1H), 4.67−4.53 (m, 1H), 4.40 (q, J = 7.4 Hz, 1H), 3.50−3.31 (m, 3H), 2.97 (d, J = 16.2 Hz, 1H), 2.78−2.59 (m, 2H), 2.53−2.40 (m, 2H), 2.30−2.17 (m, 2H), 2.02−1.93 (m, 2H), 1.89−1.70 (m, 5H), 1.67−1.39 (m, 10H), 1.20−0.88 (m, 11H), 0.82−0.73 (m, 6H).

^{13}C NMR (100 MHz, $CDCl_3$) δ (ppm) = 199.1, 175.4, 167.8, 139.2, 134.6, 133.2, 129.9, 128.5, 122.6, 109.2, 88.9, 80.7, 75.2, 66.8, 62.0, 56.3, 49.8, 43.6, 41.5, 40.2, 39.6, 37.7, 36.8, 36.6, 31.9, 31.8, 31.3, 30.9, 30.2, 28.7, 27.8, 27.5, 20.7, 19.2, 17.1, 16.2, 14.5.

HRMS (ESI) for $C_{40}H_{52}O_7Na$ $[M+Na]^+$ calcd. 667.3605, found 667.3615.

(3S,8S,9S,10R,13S,14S,17S)-17-acetyl-10,13-dimethyl-2,3,4,7,8,9,10,11,12,13,14,15,16,17-tetradecahydro-1H-cyclopenta[a]phenanthren-3-yl 2-((S)-2-benzoyl-5-oxotetrahydrofuran-2-yl)acetate (3bb)

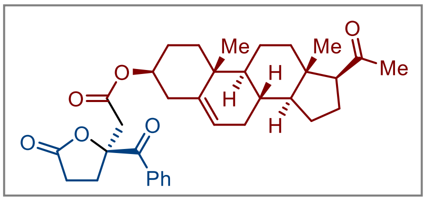

Purification by flash chromatography (PE/EA = 2/1) afforded **3bb**. Colorless oil; 29.5 mg, 54% yield; Chiral HPLC conditions: de = 95%; Chiralpak OD-H 95:5 Hexane/iPrOH, 1.0 mL/min, 80 min. t_R (minor) = 45.0 min. and t_R (major) = 66.4 min. λ = 245 nm.

[α]$_D^{25}$ = +25.8 (c = 0.1, $CHCl_3$).

^{1}H NMR (400 MHz, $CDCl_3$) δ (ppm) = 8.12−7.96 (m, 2H), 7.57 (t, J = 7.4 Hz, 1H), 7.46 (t, J = 7.7 Hz, 2H), 5.44−5.26 (m, 1H), 4.70−4.52 (m, 1H), 3.36 (d, J = 16.2 Hz,

1H), 2.98 (d, J = 16.2 Hz, 1H), 2.78−2.59 (m, 2H), 2.56−2.41 (m, 3H), 2.30−2.10 (m, 6H), 2.06−1.94 (m, 2H), 1.87−1.78 (m, 2H), 1.71−1.62 (m, 2H), 1.60−1.50 (m, 2H), 1.49−1.40 (m, 3H), 1.29−1.04 (m, 4H), 1.03−0.92 (m, 4H), 0.62 (s, 3H).

^{13}C NMR (100 MHz, $CDCl_3$) δ (ppm) = 209.5, 199.2, 175.3, 167.8, 139.2, 134.6, 133.2, 129.9, 128.5, 122.6, 88.9, 75.1, 63.6, 56.7, 49.7, 43.9, 43.6, 38.7, 37.7, 36.8, 36.5, 31.7, 31.7, 31.5, 30.9, 27.8, 27.4, 24.4, 22.7, 20.9, 19.2, 13.2.

HRMS (ESI) for $C_{34}H_{42}O_6Na$ $[M+Na]^+$ calcd. 569.2874, found 569.2879.

(3S,5S,8R,9S,10S,13S,14S)-10,13-dimethyl-17-oxohexadecahydro-1H-cyclopenta [a]phenanthren-3-yl 2-((S)-2-benzoyl-5-oxotetrahydrofuran-2-yl)acetate (3av)

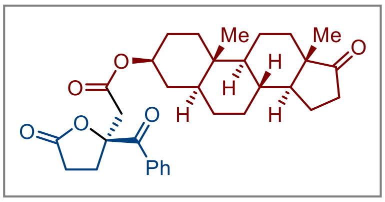

Purification by flash chromatography (PE/EA = 2/1) afforded **3bc**. Colorless oil; 46.8 mg, 90% yield; Chiral HPLC conditions: de = 94%; Chiralpak IB 95:5 Hexane/iPrOH, 1.0 mL/min, 100 min. t_R (minor) = 60.1 min. and t_R (major) = 68.7 min. λ = 245 nm.

$[\alpha]_D^{25}$ = +56.1 (c = 0.1, $CHCl_3$).

^{1}H NMR (400 MHz, $CDCl_3$) δ (ppm) = 8.03 (d, J = 7.5 Hz, 2H), 7.57 (t, J = 7.4 Hz, 1H), 7.46 (t, J = 7.6 Hz, 2H), 4.79−4.58 (m, 1H), 3.36 (d, J = 16.2 Hz, 1H), 2.97 (d, J = 16.2 Hz, 1H), 2.79−2.57 (m, 2H), 2.54−2.36 (m, 3H), 2.11−2.00 (m, 1H), 1.96−1.87 (m, 1H), 1.82−1.67 (m, 4H), 1.65−1.40 (m, 5H), 1.36−1.21 (m, 6H), 1.19−1.11 (m, 1H), 1.04−0.92 (m, 2H), 0.85 (s, 3H), 0.80 (s, 3H), 0.74−0.63 (m, 1H).

^{13}C NMR (100 MHz, $CDCl_3$) δ (ppm) = 221.2, 199.2, 175.3, 167.9, 134.6, 133.1, 129.9, 128.4, 88.8, 74.8, 54.1, 51.2, 47.7, 44.5, 43.7, 36.5, 35.7, 35.5, 34.9, 33.5, 31.4, 30.9, 30.6, 28.1, 27.8, 27.1, 21.7, 20.3, 13.7, 12.1.

HRMS (ESI) for $C_{32}H_{40}O_6Na$ $[M+Na]^+$ calcd. 543.2717, found 543.2723.

(1S,2R,5S)-2-isopropyl-5-methylcyclohexyl 2-((S)-2-benzoyl-5-oxotetrahydrofuran-2-yl)acetate and (1R,2S,5R)-2-isopropyl-5-methylcyclohexyl 2-((S)-2-benzoyl-5-oxotetrahydrofuran-2-yl)acetate (3at and 3au)

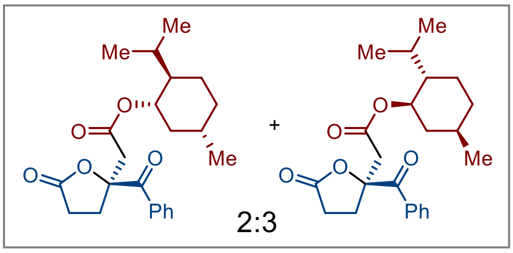

Purification by flash chromatography (PE/EA = 4/1) afforded **3at** and **3au**. White solid; m.p. = 120−122 °C; 32.1 mg, 83% yield; Chiral HPLC conditions: de = 96%; Chiral HPLC conditions: de = 96%; Chiralpak IA 95:5 Hexane/iPrOH, 1.0 mL/min, 30 min. $t_{R'}$ (minor) = 9.2 min. and $t_{R'}$ (major) = 11.1 min.; t_R (minor) = 10.0 min. and t_R (major) = 11.9 min. λ = 245 nm.

$[\alpha]_D^{25}$ = +6.0 (c = 0.1, $CHCl_3$).

^{1}H NMR (400 MHz, $CDCl_3$) δ (ppm) = 8.07−7.95 (m, 2H), 7.56 (t, J = 7.4 Hz, 1H), 7.46 (t, J = 7.6 Hz, 2H), 4.74−4.60 (m, 1H), 3.42−3.27 (m, 1H), 3.08−2.94 (m, 1H), 2.82−2.70 (m, 1H), 2.69−2.58 (m, 1H), 2.54−2.37 (m, 2H), 2.00−1.89 (m, 1H), 1.81−1.60 (m, 3H), 1.50−1.36 (m, 1H), 1.34−1.21 (m, 1H), 1.06−0.79 (m, 9H), 0.73−0.65 (m, 3H).

^{13}C NMR (100 MHz, $CDCl_3$) δ (ppm) = 199.1, 198.9, 175.3, 175.3, 168.0, 134.6, 134.6, 133.1, 133.1, 130.0, 129.8, 128.5, 128.4, 88.8, 88.7, 75.6, 75.6, 46.7, 46.7, 43.6, 43.6, 40.6, 40.6, 34.0, 31.3, 31.0, 30.7, 27.8, 26.1, 26.0, 23.2, 23.2, 21.9, 20.6, 20.6, 16.1.

HRMS (ESI) for $C_{23}H_{30}O_5Na$ $[M+Na]^+$ calcd. 409.1985, found 409.1989.

(4S)-6-(((3R)-adamantan-1-yl)methoxy)-4-benzoyl-4-hydroxy-6-oxohexanoic acid (3a-I)

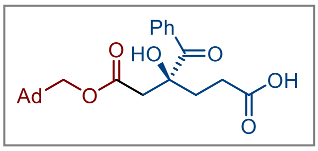

Purification by flash chromatography (PE/EA = 1/1) afforded **3a-I**. Colorless oil; 33.6 mg, 81% yield; Chiral HPLC conditions: ee = 92%; Chiralpak AZ-H 80:20 Hexane/iPrOH, 1.0 mL/min, 15 min. t_R (minor) = 6.8 min. and t_R (major) = 9.7 min. λ = 243 nm.

$[\alpha]_D^{25}$ = +28.0 (c = 0.1, $CHCl_3$).

^{1}H NMR (400 MHz, $CDCl_3$) δ (ppm) = 8.13 (d, J = 7.4 Hz, 2H), 7.55 (t, J = 7.4 Hz, 1H), 7.44 (t, J = 7.7 Hz, 2H), 4.81 (br, 1H), 3.68−3.60 (m, 2H), 3.28 (d, J = 16.5 Hz, 1H), 2.77 (d, J = 16.5 Hz, 1H), 2.55−2.32 (m, 3H), 2.22−2.12 (m, 1H), 1.95 (s, 3H),

1.74−1.66 (m, 3H), 1.64−1.57 (m, 3H), 1.48−1.41 (m, 6H).

^{13}C NMR (100 MHz, $CDCl_3$) δ (ppm) = 202.6, 178.4, 172.2, 134.4, 133.1, 129.8, 128.4, 79.8, 74.7, 43.1, 39.0, 36.8, 33.8, 33.0, 28.2, 27.9.

HRMS (ESI) for $C_{24}H_{30}O_6Na$ $[M+Na]^+$ calcd. 437.1935, found 437.1944.

((3R)-adamantan-1-yl)methyl (3S)-3-benzoyl-3-hydroxy-6-oxo-6-(piperidin-1-yl)hexanoate (3a-II)

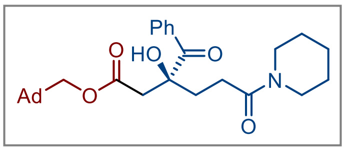

Purification by flash chromatography (PE/EA = 2/1) afforded **3a-II**. Colorless oil; 28.4 mg, 59% yield; Chiral HPLC conditions: ee = 94%; Chiralpak IA 95:5 Hexane/iPrOH, 1.0 mL/min, 45 min. t_R (minor) = 27.8 min. and t_R (major) = 38.3 min. λ = 243nm.

$[\alpha]_D^{25}$ = +10.6 (c = 0.1, $CHCl_3$).

^{1}H NMR (400 MHz, $CDCl_3$) δ (ppm) = 8.15 (d, J = 7.6 Hz, 2H), 7.54 (t, J = 7.3 Hz, 1H), 7.43 (t, J = 7.6 Hz, 2H), 3.63 (s, 2H), 3.58−3.44 (m, 2H), 3.38−3.23 (m, 3H), 2.82 (d, J = 16.2 Hz, 1H), 2.52−2.22 (m, 4H), 1.94 (s, 3H), 1.73−1.56 (m, 9H), 1.53−1.46 (m, 4H), 1.45−1.39 (m, 6H).

^{13}C NMR (100 MHz, $CDCl_3$) δ (ppm) = 203.3, 171.8, 170.3, 134.9, 132.8, 129.9, 128.3, 80.1, 74.5, 46.5, 44.0, 42.9, 39.0, 36.8, 34.6, 33.0, 27.9, 27.5, 26.3, 25.5, 24.4.

HRMS (ESI) for $C_{29}H_{39}O_5NNa$ $[M+Na]^+$ calcd. 504.2720, found 504.2724.

(2R)-2-(2-(((3S)-adamantan-1-yl)methoxy)-2-oxoethyl)-5-oxotetrahydrofuran-2-yl benzoate (3a-III)

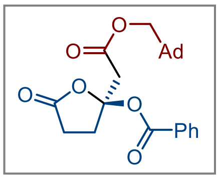

Purification by flash chromatography (PE/EA = 4/1) afforded **3a-III**. Colorless oil; 31.3 mg, 76% yield; Chiral HPLC conditions: ee = 96%; Chiralpak AZ-H 95:5 Hexane/iPrOH, 1.0 mL/min, 40 min. t_R (minor) = 27.5 min. and t_R (major) = 30.8 min. λ = 230nm.

$[\alpha]_D^{25}$ = -27.1 (c = 0.1, $CHCl_3$).

^{1}H NMR (400 MHz, $CDCl_3$) δ (ppm) = 7.99 (d, J = 7.3 Hz, 2H), 7.59 (t, J = 7.4 Hz,

1H), 7.44 (t, J = 7.7 Hz, 2H), 3.80−2.70 (m, 2H), 3.40 (d, J = 16.0 Hz, 1H), 3.19 (d, J = 16.0 Hz, 1H), 3.11−2.99 (m, 1H), 2.90−2.70 (m, 3H), 1.96 (s, 3H), 1.75−1.67 (m, 3H), 1.65−1.57 (m, 3H), 1.56−1.48 (m, 6H).

^{13}C NMR (100 MHz, $CDCl_3$) δ (ppm) = 175.2, 168.1, 164.4, 133.7, 129.8, 129.4, 128.5, 107.2, 74.7, 43.4, 39.2, 36.8, 33.1, 31.6, 28.8, 27.9.

HRMS (ESI) for $C_{24}H_{28}O_6Na$ $[M+Na]^+$ calcd. 435.1778, found 435.1790.

((3R)-adamantan-1-yl)methyl 2-((S)-2-((R)-hydroxy(phenyl)methyl)-5-oxotetrahydrofuran-2-yl)acetate (3a-IV)

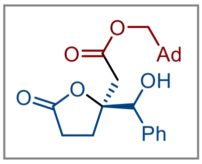

Purification by flash chromatography (PE/EA = 3/1) afforded **3a-IV**. Colorless oil; 35.0 mg, 88% yield, dr 2:1; Chiral HPLC conditions: ee = 95%/ 97%; Chiralpak AZ-H 90:10 Hexane/iPrOH, 0.5 mL/min, 70 min. t_1 = 37.3 min. t_2 = 39.8 min. t_3 = 43.3 min. t_4 = 57.6 min. λ = 206 nm.

$[\alpha]_D^{25}$ = +15.3 (c = 0.1, $CHCl_3$).

^{1}H NMR (400 MHz, $CDCl_3$) δ (ppm) = 7.45−7.32 (m, 5H), 5.00−4.80 (m, 1H), 3.73−3.65 (m, 2H), 2.90−2.40 (m, 5H), 2.30−2.11 (m, 2H), 1.98 (s, 3H), 1.75−1.68 (m, 3H), 1.67−1.60 (m, 3H), 1.53−1.48 (m, 6H).

^{13}C NMR (100 MHz, $CDCl_3$) δ (ppm) = 176.9, 176.8, 170.1, 170.0, 138.2, 138.0, 128.6, 128.5, 128.5, 127.7, 127.6, 87.2, 86.4, 77.2, 74.6, 41.6, 40.8, 39.1, 39.0, 37.1, 36.8, 33.0, 29.1, 28.9, 28.1, 27.9, 27.7, 26.4.

HRMS (ESI) for $C_{24}H_{30}O_5Na$ $[M+Na]^+$ calcd. 421.1985, found 421.1989.

((3R)-adamantan-1-yl)methyl (3S)-3-benzoyl-6-(benzylamino)-3-hydroxy-6-oxohexanoate (3a-V)

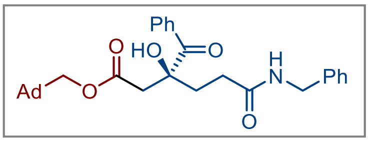

Purification by flash chromatography (PE/EA = 2/1) afforded **3a-V**. Colorless oil; 23.2 mg, 46% yield; Chiral HPLC conditions: ee = 95%; Chiralpak IA 90:10 Hexane/iPrOH, 1.0 mL/min, 30 min. t_R (minor) = 17.2 min. and t_R (major) = 21.6 min. λ = 245nm.

[α]$_D^{25}$ = +22.7 (c = 0.075, $CHCl_3$).

^{1}H NMR (400 MHz, $CDCl_3$) δ (ppm) = 8.18−8.04 (m, 2H), 7.54 (t, *J* = 7.4 Hz, 1H), 7.43 (t, *J* = 7.6 Hz, 2H), 7.35−7.21 (m, 5H), 5.89 (br, 1H), 4.45−4.32 (m, 2H), 3.62 (s, 2H), 3.28 (d, *J* = 16.3 Hz, 1H), 2.80 (d, *J* = 16.3 Hz, 1H), 2.44−2.34 (m, 2H), 2.32−2.21 (m, 2H), 1.94 (s, 3H), 1.73−1.66 (m, 3H), 1.63−1.56 (m, 3H), 1.46−1.39 (m, 6H).

^{13}C NMR (100 MHz, $CDCl_3$) δ (ppm) = 203.0, 171.9, 137.9, 134.7, 133.0, 129.8, 128.7, 128.4, 127.8, 127.6, 80.1, 74.6, 43.7, 39.0, 36.8, 34.6, 33.0, 30.5, 27.9.

HRMS (ESI) for $C_{31}H_{37}O_5NNa$ $[M+Na]^+$ calcd. 526.2564, found 526.2562.

((3r,5r,7r)-adamantan-1-yl)methyl (2,2,6,6-tetramethylpiperidin-1-yl) carbonate (4)

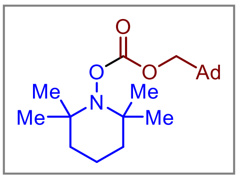

Purification by flash chromatography (PE/EA = 10/1) afforded **4**. Colorless oil; 53.4 mg, 51% yield;

^{1}H NMR (400 MHz, $CDCl_3$) δ (ppm) = 3.77 (s, 2H), 1.99 (s, 3H), 1.77−1.62 (m, 9H), 1.61−1.49 (m, 8H), 1.42−1.36 (m, 1H), 1.18 (s, 6H), 1.12 (s, 6H).

^{13}C NMR (100 MHz, $CDCl_3$) δ (ppm) = 157.3, 77.5, 60.3, 39.2, 39.0, 36.9, 33.6, 31.5, 27.9, 20.4, 16.9.

HRMS (ESI) for $C_{21}H_{36}O_3$ $[M+H]^+$ calcd. 350.2690, found 350.2693.

9. HPLC data and chromatograms

3a

<Chromatogram>

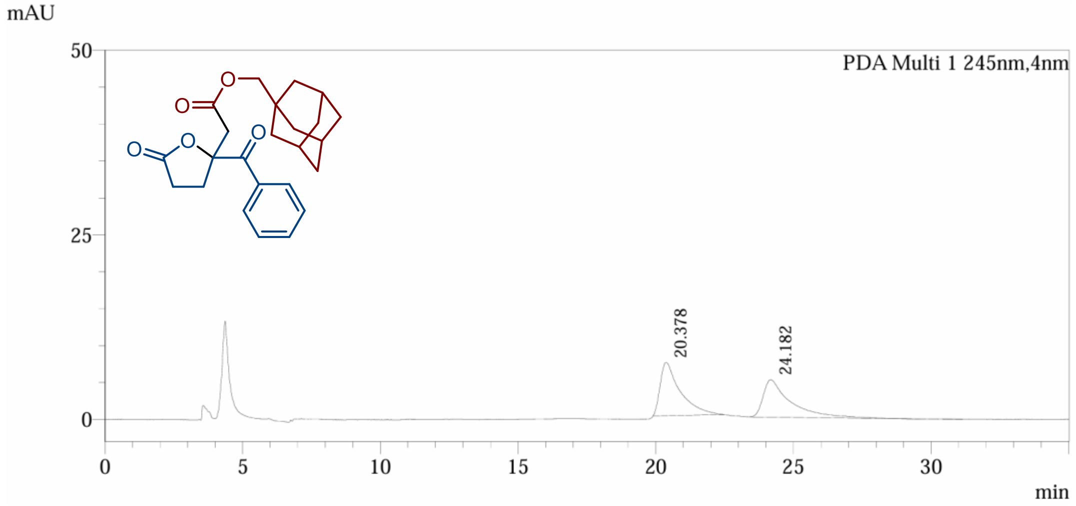

<Peak Table>

PDA Ch1 245nm

Peak#	Ret. Time	Area	Height	Aera%
1	20.378	364868	7216	50.931
2	24.182	351530	5069	49.069

<Chromatogram>

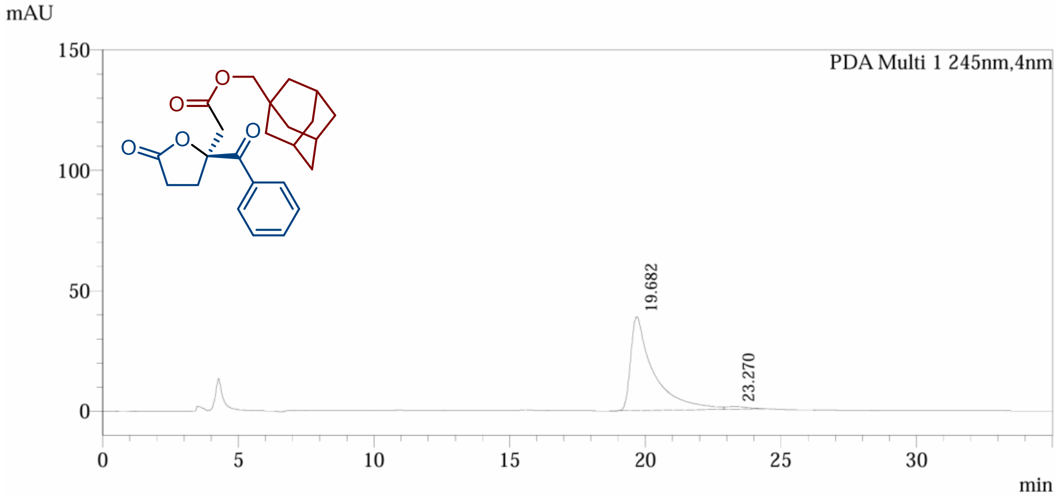

<Peak Table>

PDA Ch1 245nm

Peak#	Ret. Time	Area	Height	Aera%
1	19.682	2302333	39011	97.169
2	23.270	67088	1264	2.831

3b

<Chromatogram>

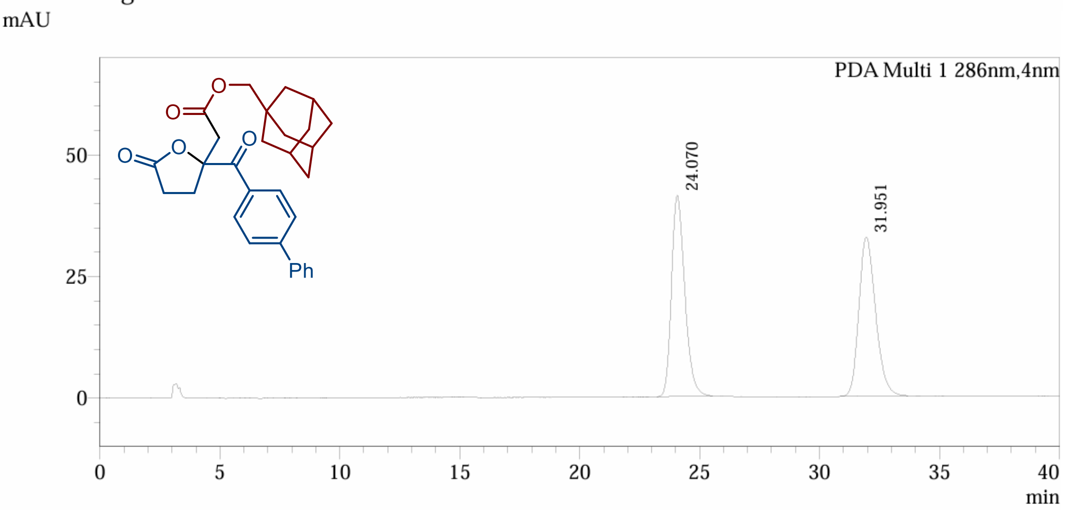

<Peak Table>

PDA Ch1 286nm

Peak#	Ret. Time	Area	Height	Aera%
1	24.070	1588863	41299	50.071
2	31.951	1584370	32680	49.929

<Chromatogram>

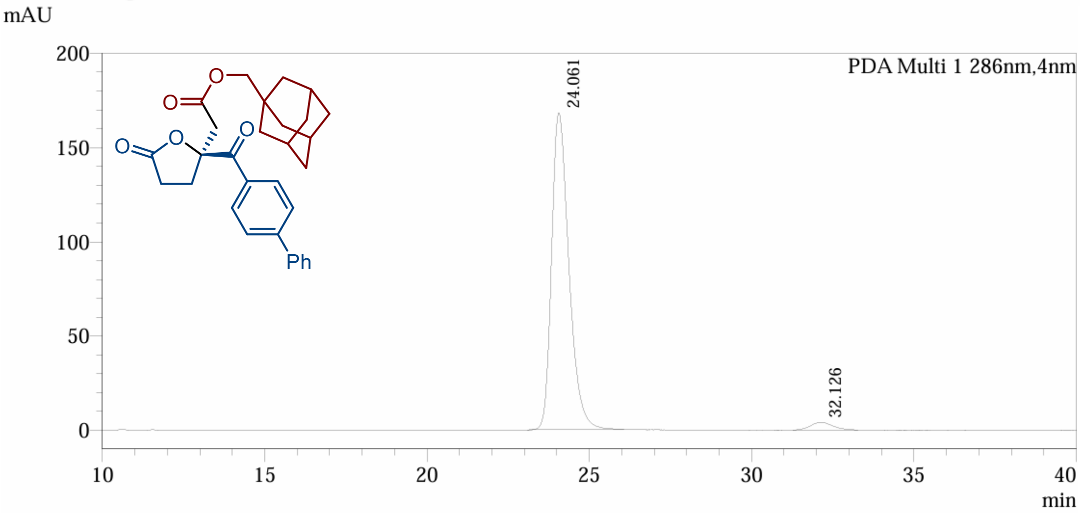

<Peak Table>

PDA Ch1 286nm

Peak#	Ret. Time	Area	Height	Aera%
1	24.061	6234654	168203	97.137
2	32.126	183787	4024	2.863

3c

<Chromatogram>

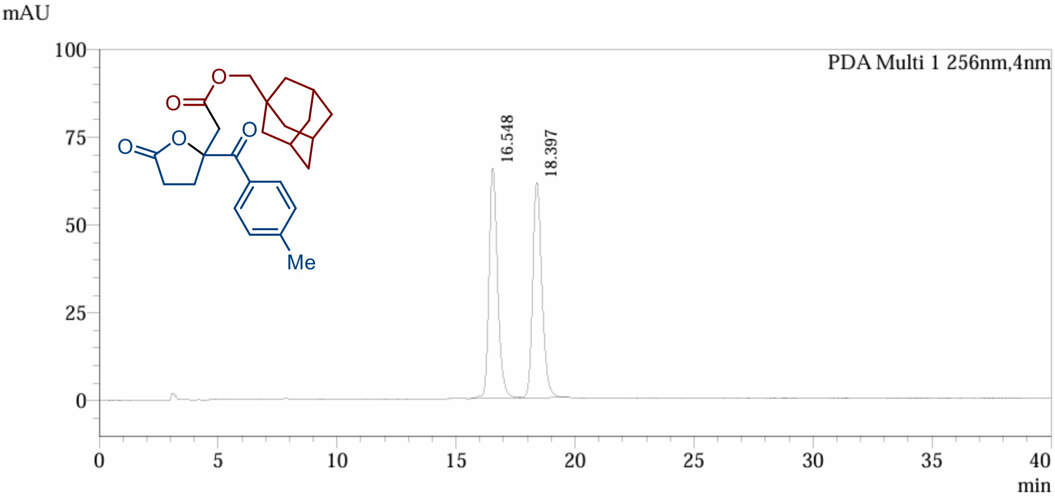

<Peak Table>

PDA Ch1 256nm

Peak#	Ret. Time	Area	Height	Aera%
1	16.548	1649315	65471	49.918
2	18.397	1654718	61263	50.082

<Chromatogram>

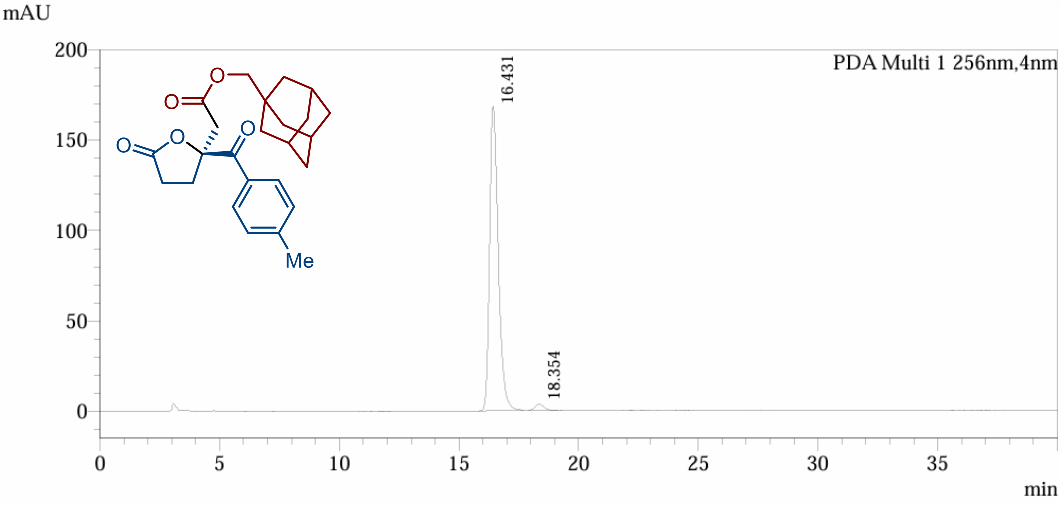

<Peak Table>

PDA Ch1 256nm

Peak#	Ret. Time	Area	Height	Aera%
1	16.431	4243339	168283	97.975
2	18.354	87716	3381	2.025

3d

<Chromatogram>

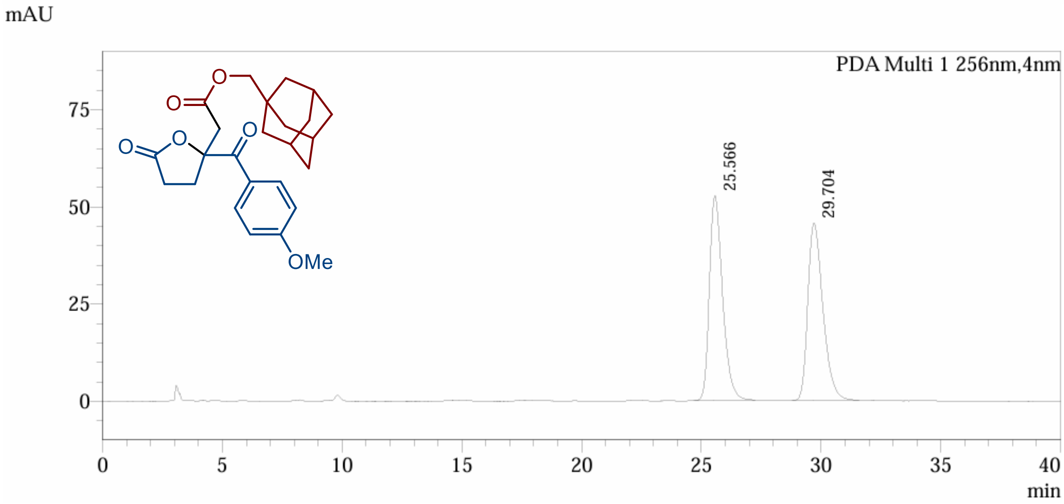

<Peak Table>

PDA Ch1 256nm

Peak#	Ret. Time	Area	Height	Aera%
1	25.566	2008233	52681	50.029
2	29.704	2005865	45716	49.971

<Chromatogram>

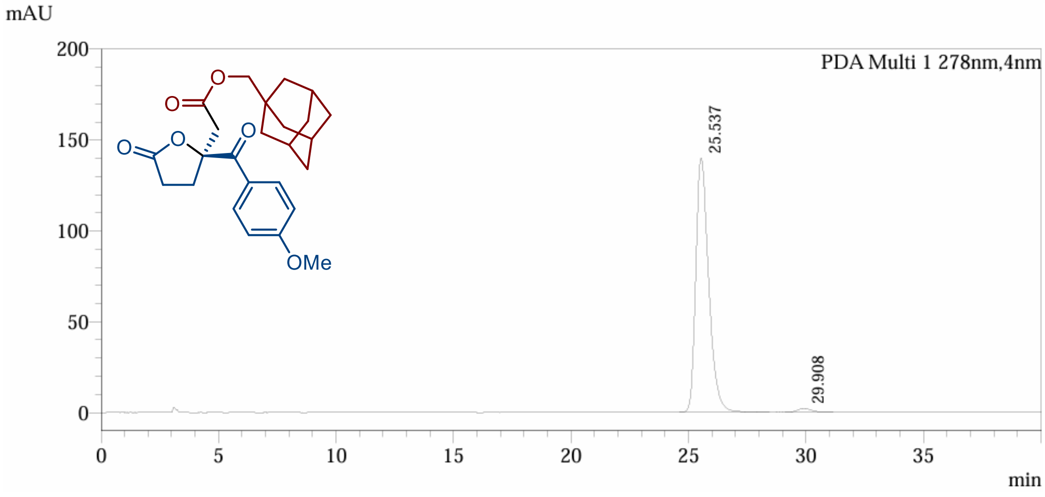

<Peak Table>

PDA Ch1 278nm

Peak#	Ret. Time	Area	Height	Aera%
1	25.537	5364464	140033	98.249
2	29.908	95621	2240	1.751

3e

<Chromatogram>

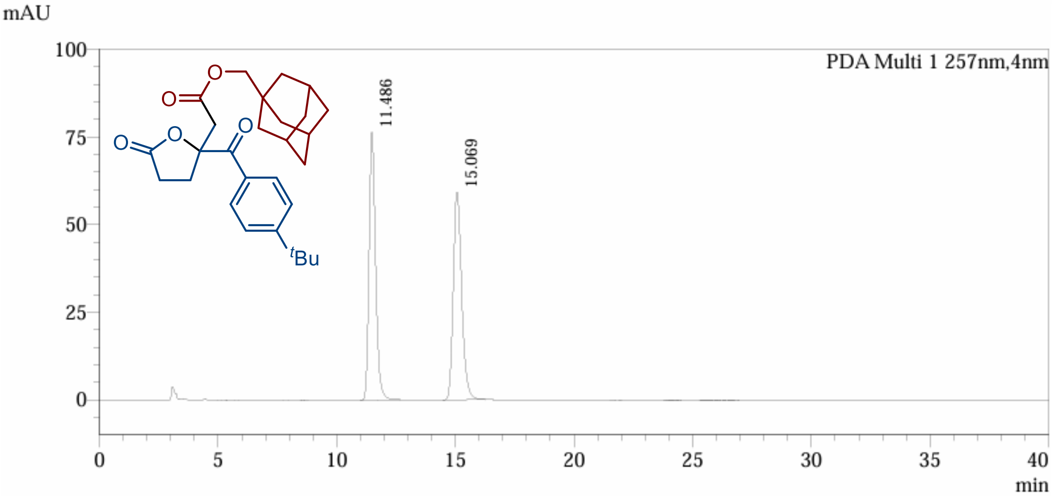

<Peak Table>

PDA Ch1 257nm

Peak#	Ret. Time	Area	Height	Aera%
1	11.486	1463507	76503	49.949
2	15.069	1466518	59317	50.051

<Chromatogram>

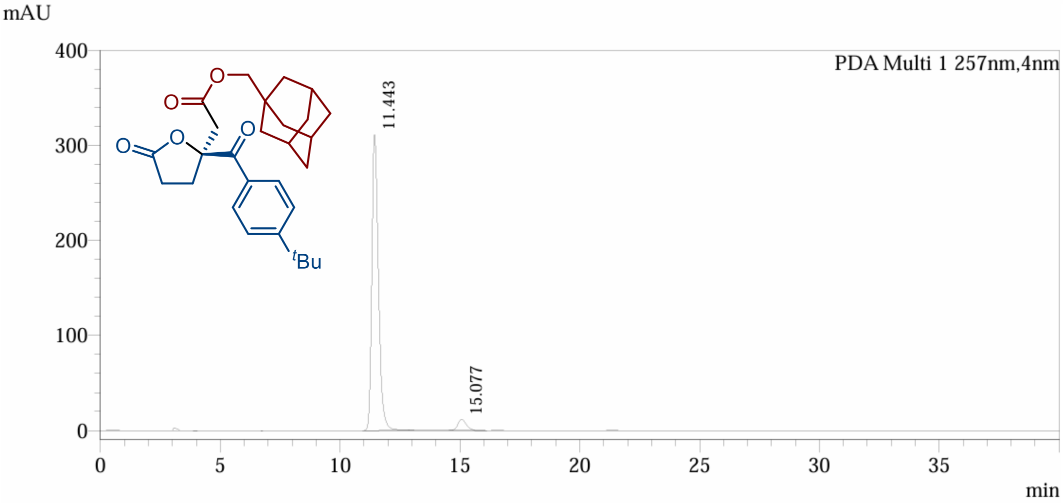

<Peak Table>

PDA Ch1 257nm

Peak#	Ret. Time	Area	Height	Aera%
1	11.443	5962810	311467	95.419
2	15.077	286268	11599	4.581

3f

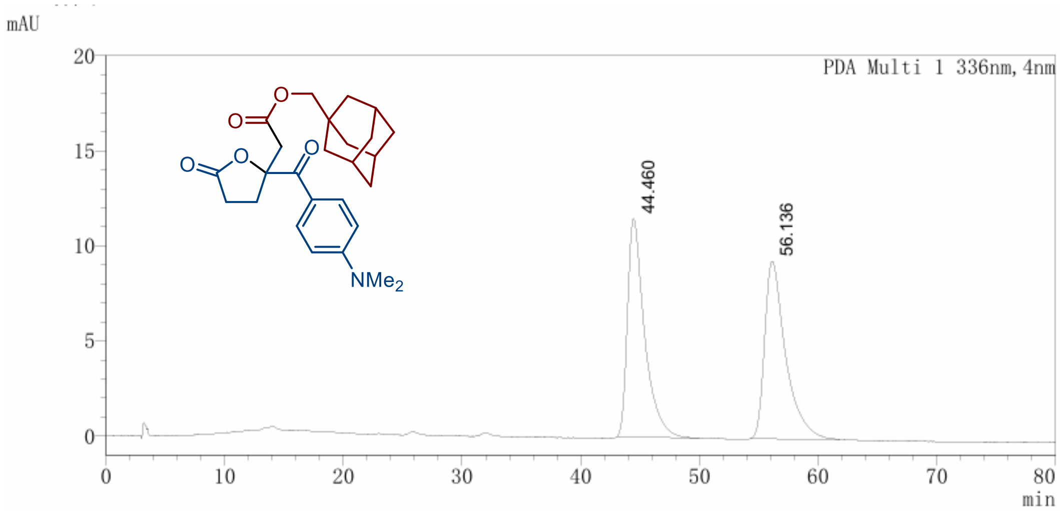

PDA Ch1 336nm

Peak	Ret. Time	Area	Area%
1	44.460	1149387	49.991
2	56.136	1149812	50.009

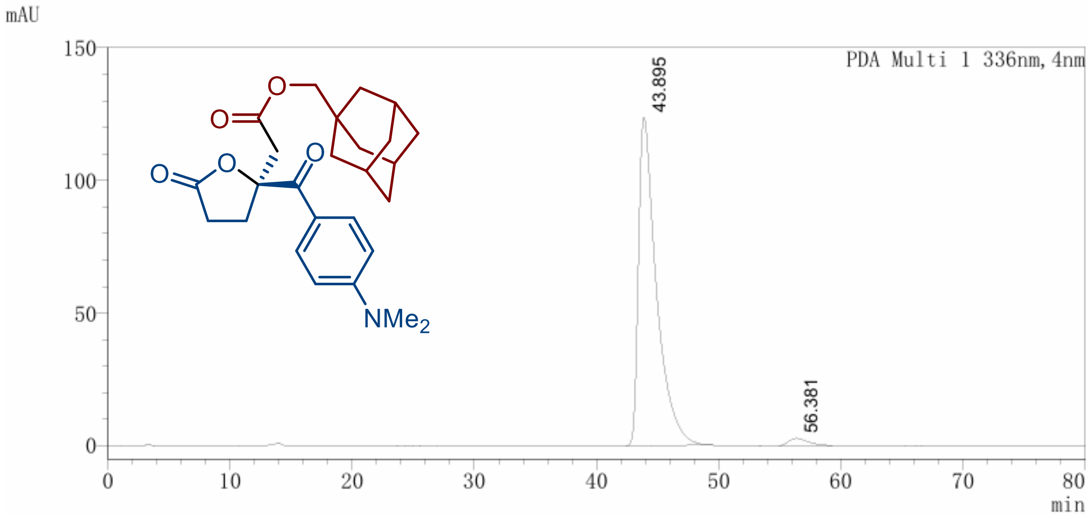

PDA Ch1 336nm

Peak	Ret. Time	Area	Area%
1	43.895	12665994	97.662
2	56.381	303221	2.338

3g

<Chromatogram>

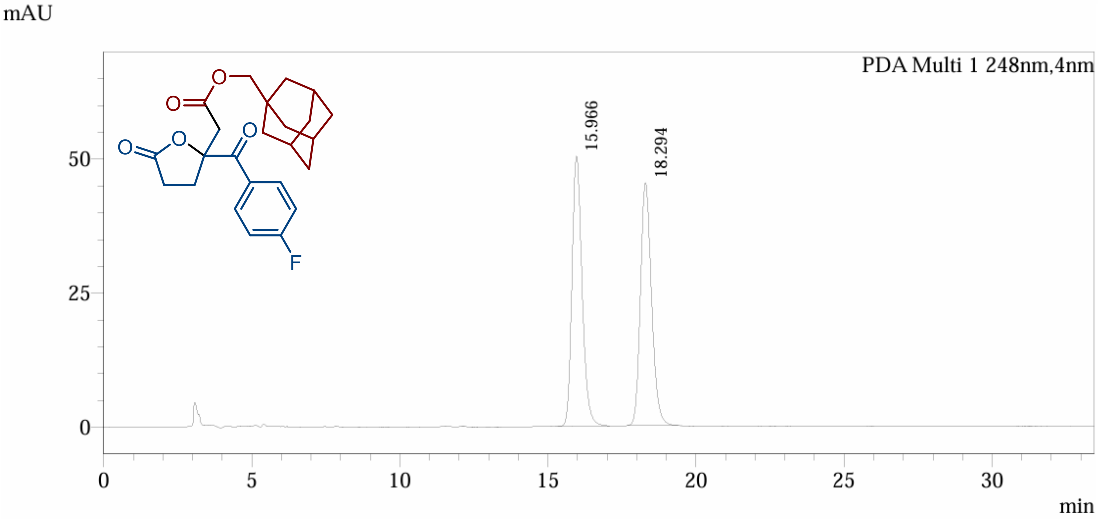

<Peak Table>

PDA Ch1 248nm

Peak#	Ret. Time	Area	Height	Aera%
1	15.966	1187051	50353	50.062
2	18.294	1184127	45263	49.938

<Chromatogram>

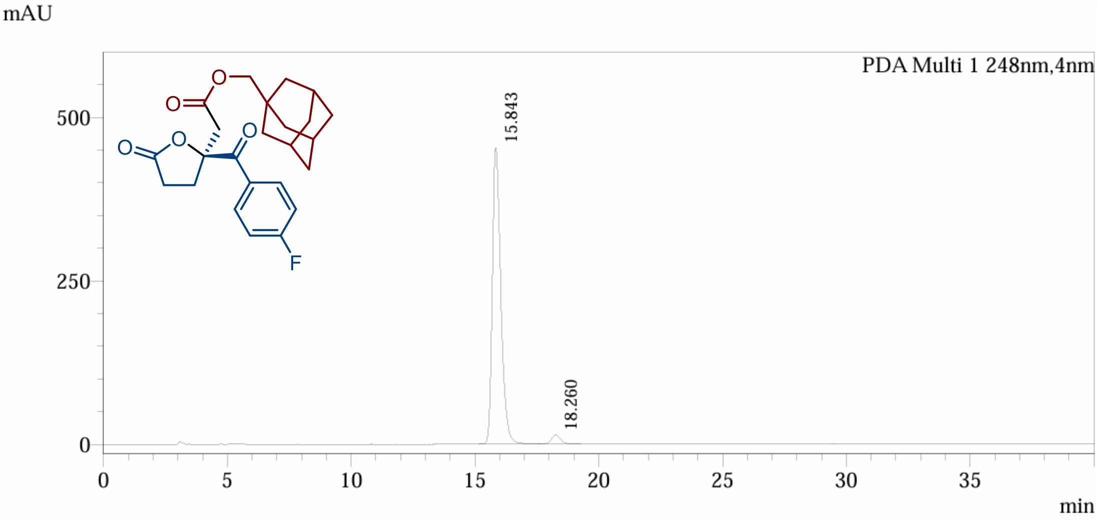

<Peak Table>

PDA Ch1 248nm

Peak#	Ret. Time	Area	Height	Aera%
1	15.843	10819072	453232	96.727
2	18.260	366101	13886	3.273

3h

<Chromatogram>

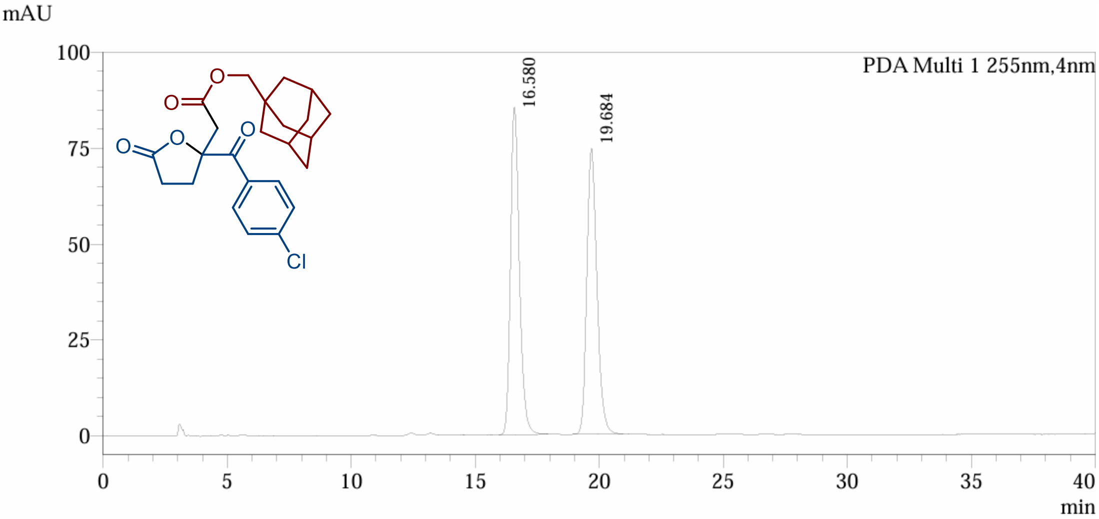

<Peak Table>

PDA Ch1 255nm

Peak#	Ret. Time	Area	Height	Aera%
1	16.580	2123435	85343	50.092
2	19.684	2115661	74503	49.908

<Chromatogram>

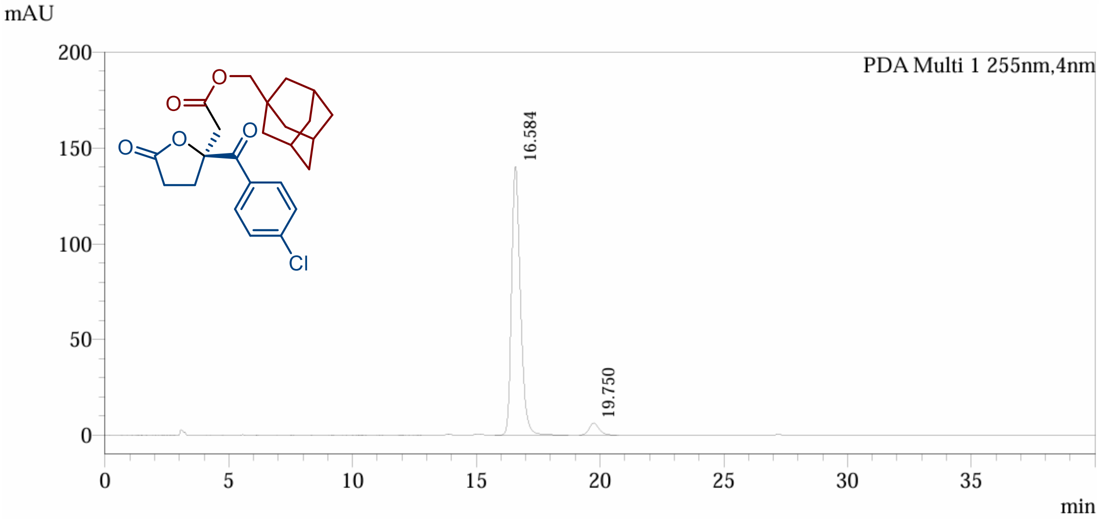

<Peak Table>

PDA Ch1 255nm

Peak#	Ret. Time	Area	Height	Aera%
1	16.584	3474105	140163	95.296
2	19.750	171494	6137	4.704

3i

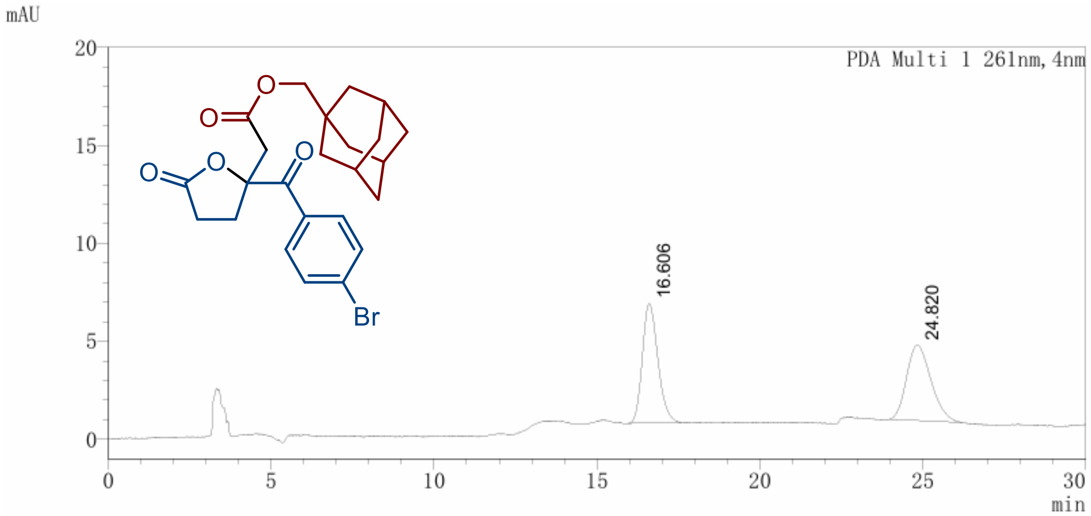

PDA Ch1 261nm

Peak	Ret. Time	Area	Area%
1	16. 606	201350	50. 262
2	24. 820	199249	49. 738

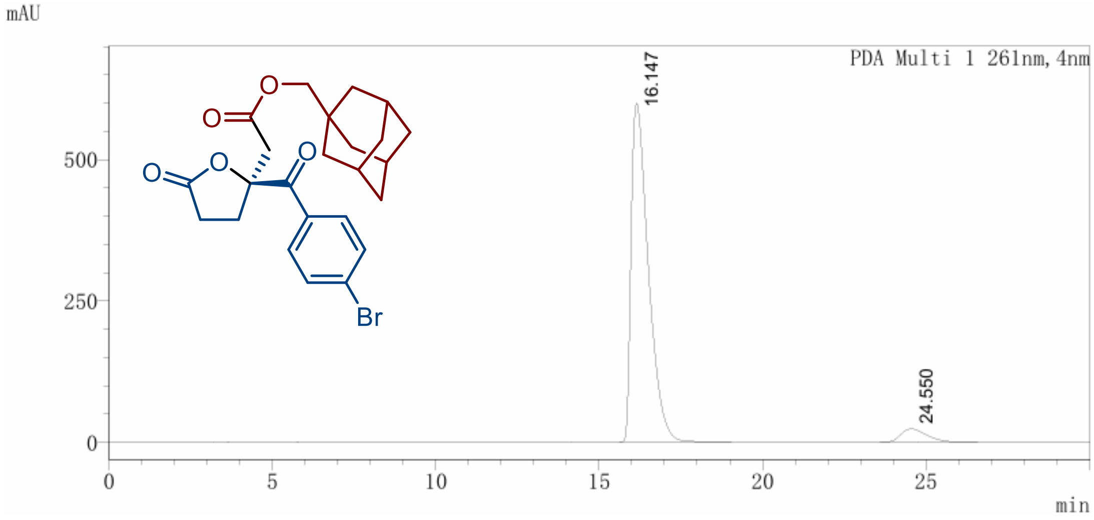

PDA Ch1 261nm

Peak	Ret. Time	Area	Area%
1	16. 147	21921881	94. 483
2	24. 550	1279979	5. 517

3j

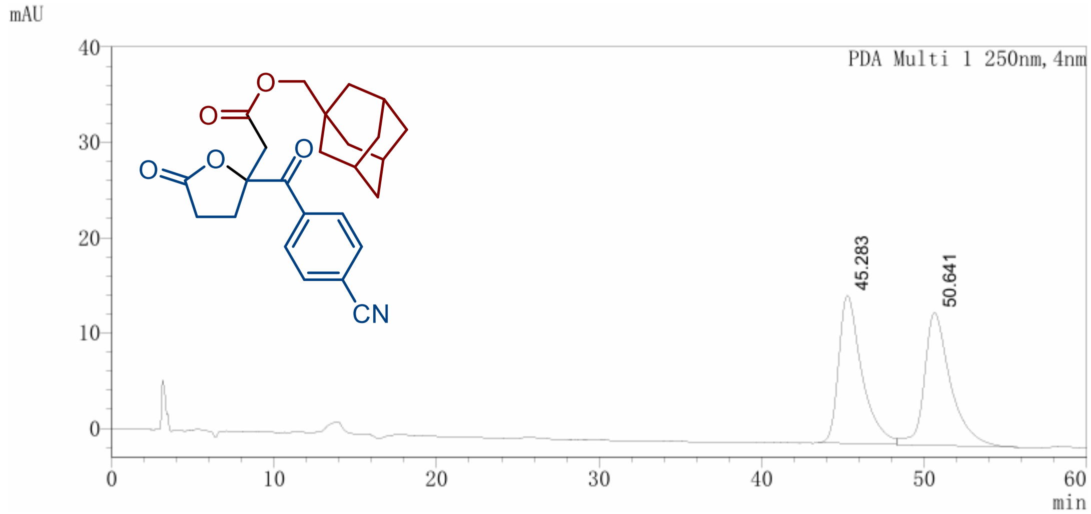

PDA Ch1 250nm

Peak	Ret. Time	Area	Area%
1	45.283	1541899	49.609
2	50.641	1566179	50.391

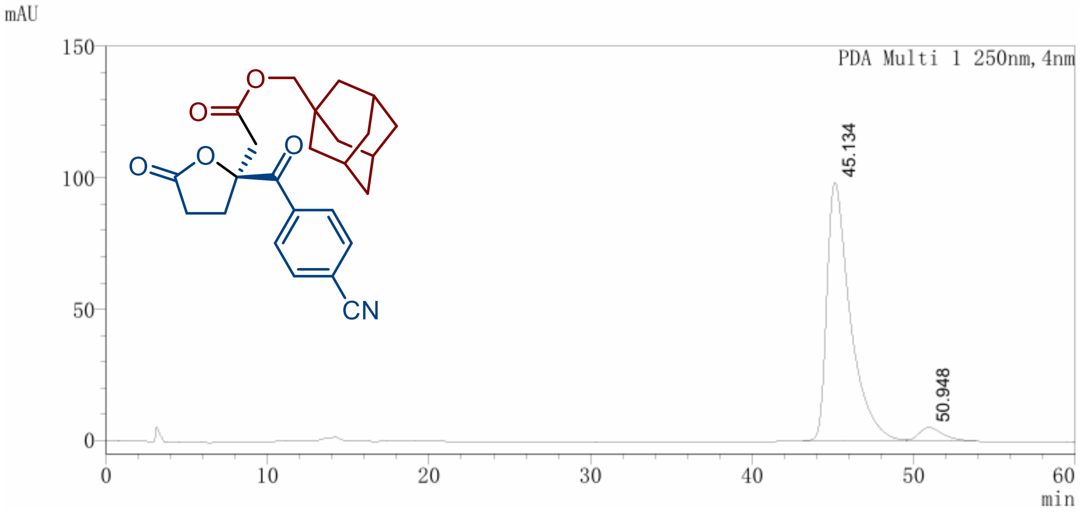

PDA Ch1 250nm

Peak	Ret. Time	Area	Area%
1	45.134	9922262	94.873
2	50.948	536170	5.127

3k

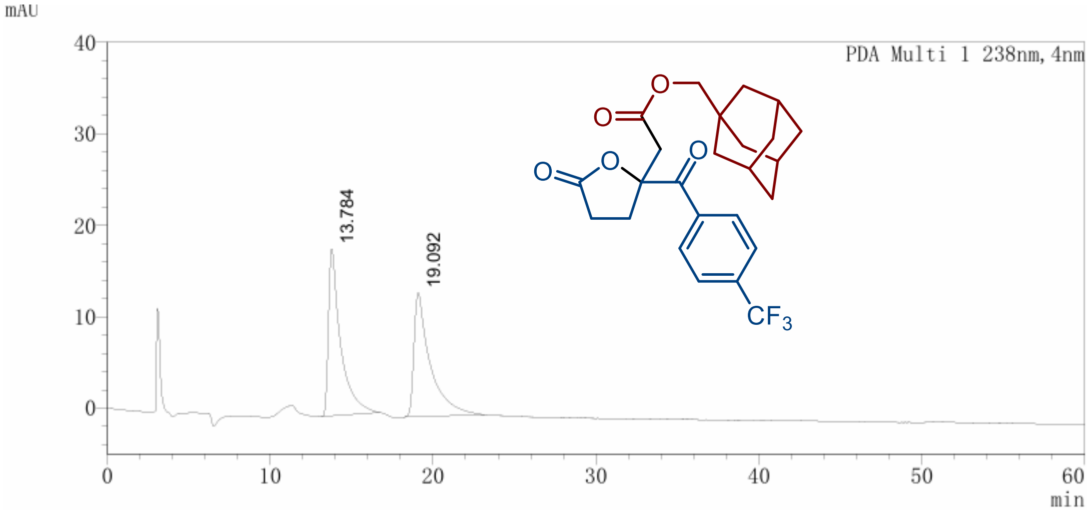

PDA Ch1 238nm

Peak	Ret. Time	Area	Area%
1	13.784	949317	50.259
2	19.092	939535	49.741

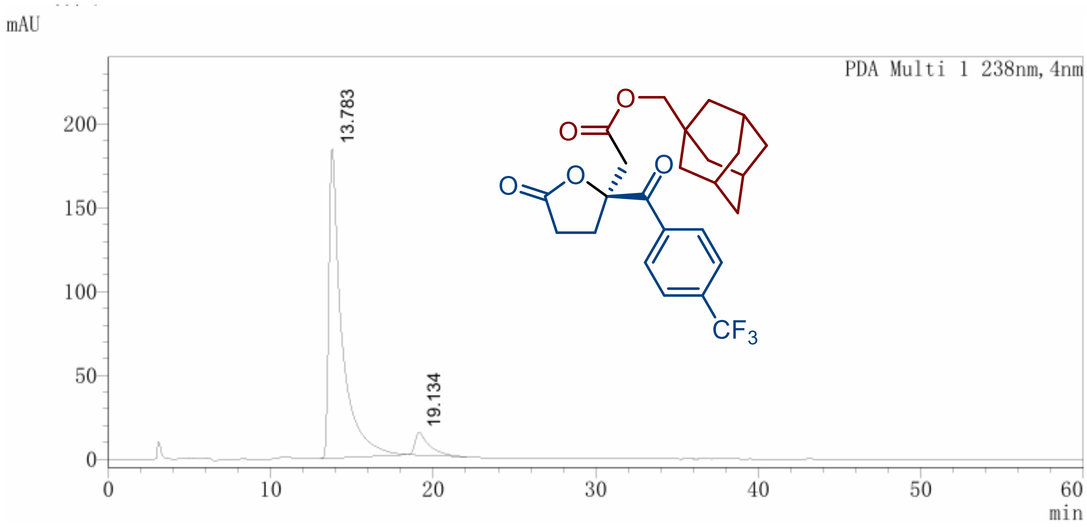

PDA Ch1 238nm

Peak	Ret. Time	Area	Area%
1	13.783	10224815	92.491
2	19.134	830118	7.509

3l

<Chromatogram>

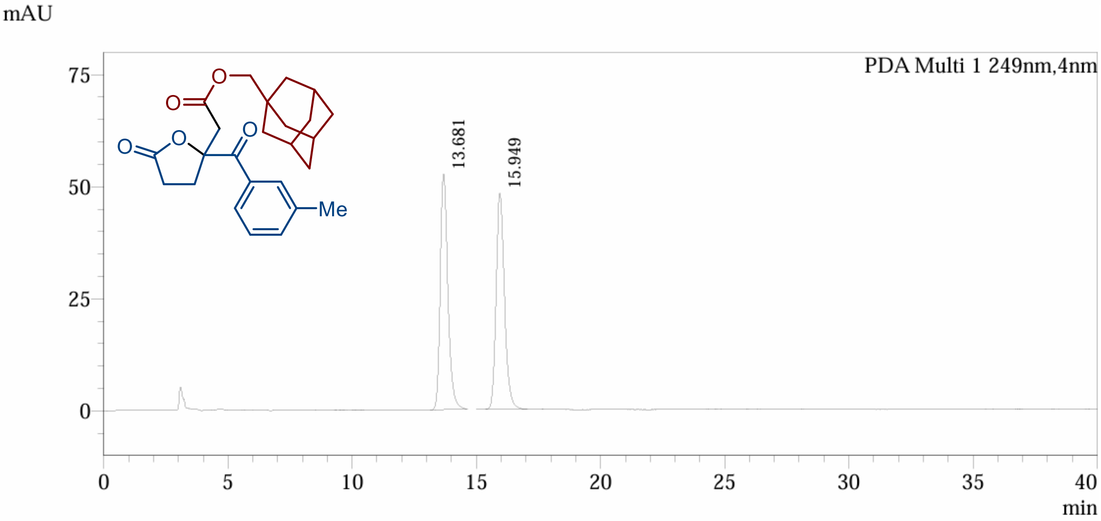

<Peak Table>

PDA Ch1 249nm

Peak#	Ret. Time	Area	Height	Aera%
1	13.681	1127971	52526	49.977
2	15.949	1128995	48205	50.023

<Chromatogram>

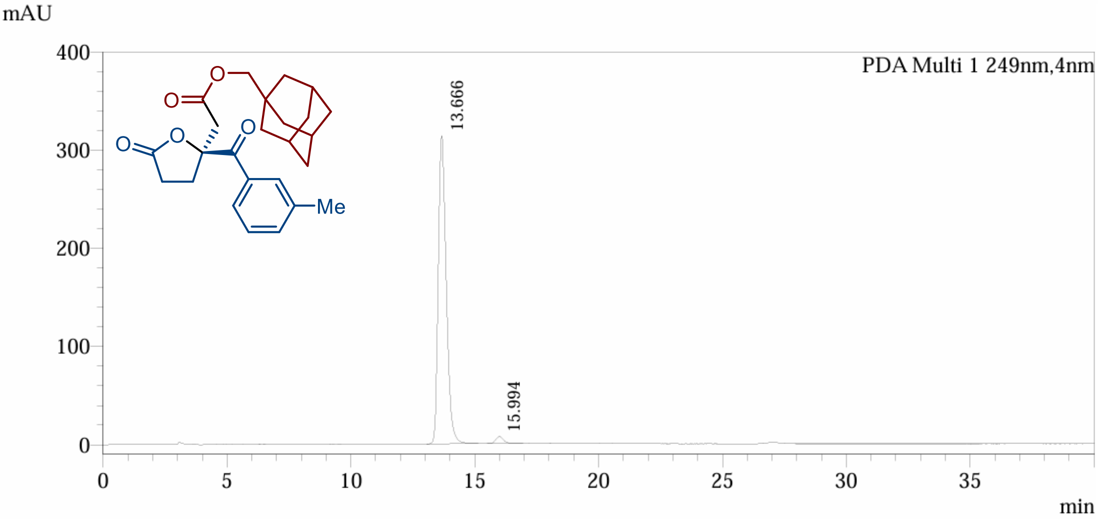

<Peak Table>

PDA Ch1 249nm

Peak#	Ret. Time	Area	Height	Aera%
1	13.666	7019481	313895	97.654
2	15.994	168631	7171	2.346

3m

<Chromatogram>

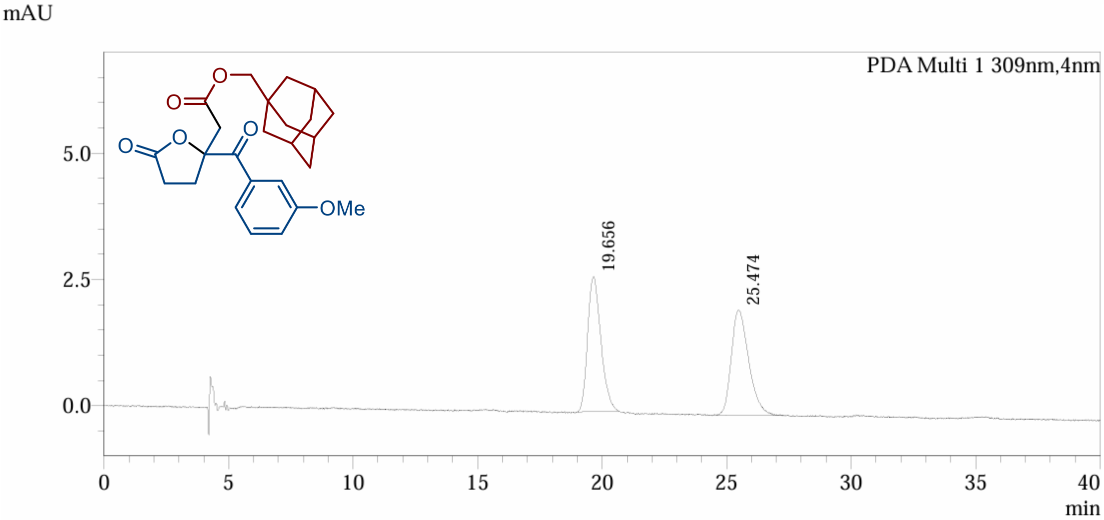

<Peak Table>

PDA Ch1 309nm

Peak#	Ret. Time	Area	Height	Aera%
1	19.656	98741	2669	49.496
2	25.474	100752	2083	50.504

<Chromatogram>

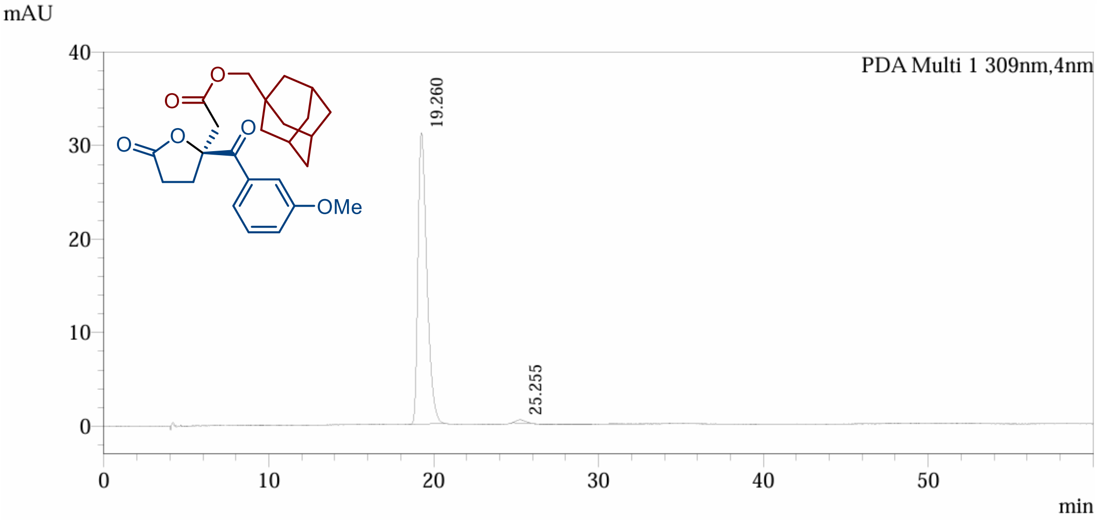

<Peak Table>

PDA Ch1 309nm

Peak#	Ret. Time	Area	Height	Aera%
1	19.260	1169663	31131	98.969
2	25.255	12187	334	1.031

3n

<Chromatogram>

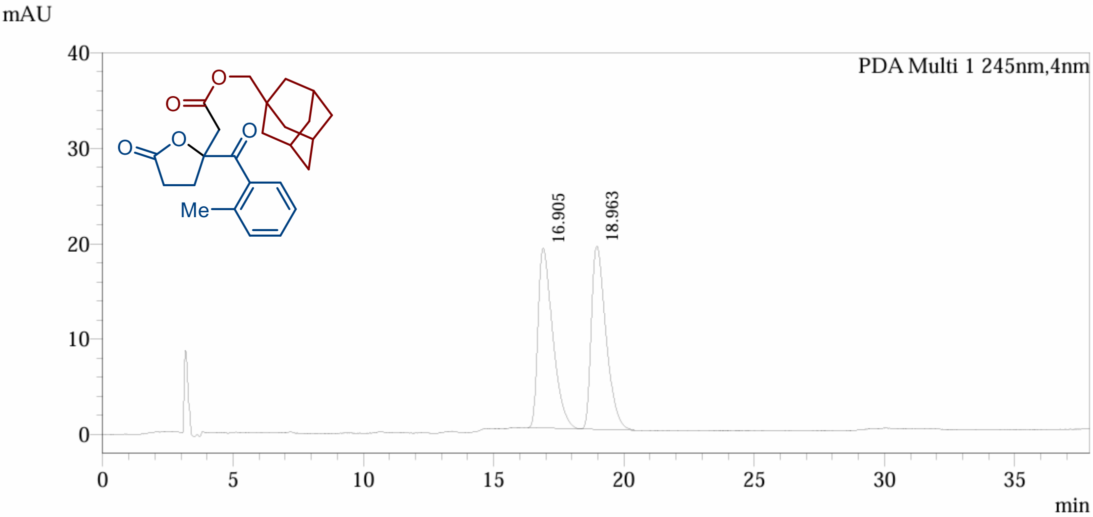

<Peak Table>

PDA Ch1 245nm

Peak#	Ret. Time	Area	Height	Aera%
1	16.905	742017	18838	49.686
2	18.963	751408	19194	50.314

<Chromatogram>

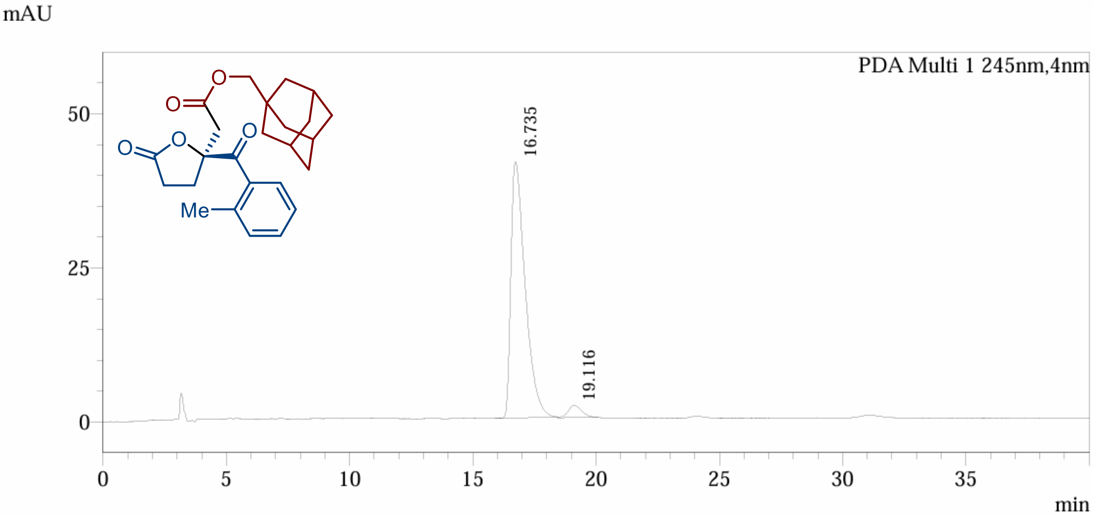

<Peak Table>

PDA Ch1 245nm

Peak#	Ret. Time	Area	Height	Aera%
1	16.735	1667646	41576	95.608
2	19.116	76603	2011	4.392

3o

<Chromatogram>

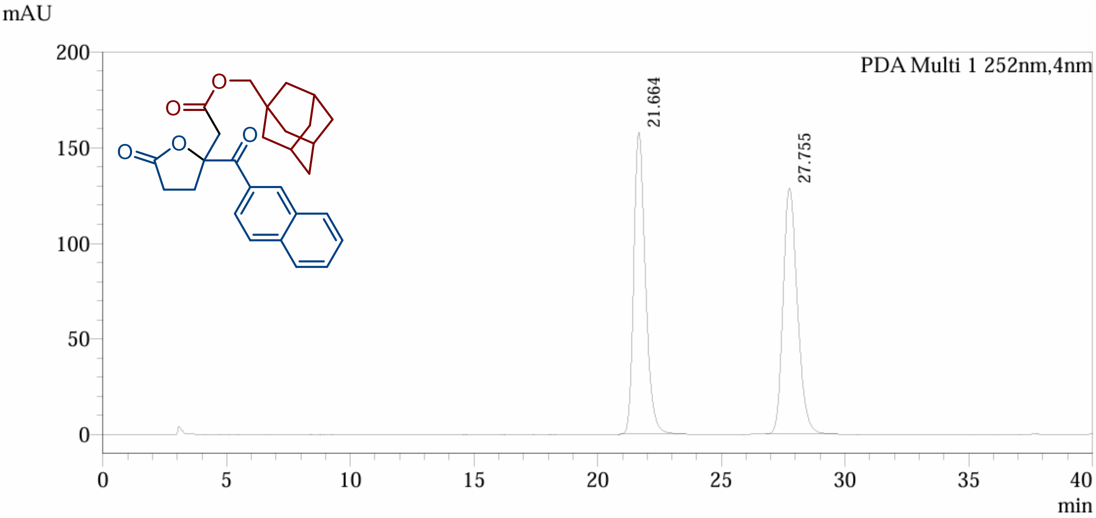

<Peak Table>

PDA Ch1 252nm

Peak#	Ret. Time	Area	Height	Aera%
1	21.664	5179661	157804	50.027
2	27.755	5174146	128592	49.973

<Chromatogram>

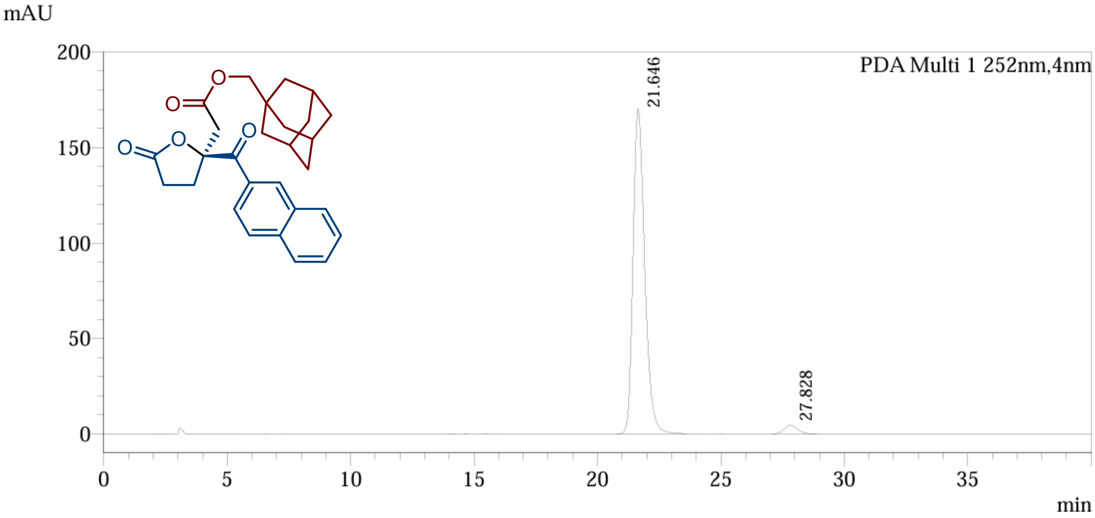

<Peak Table>

PDA Ch1 252nm

Peak#	Ret. Time	Area	Height	Aera%
1	21.646	5573735	170127	97.008
2	27.828	171920	4398	2.992

3p

<Chromatogram>

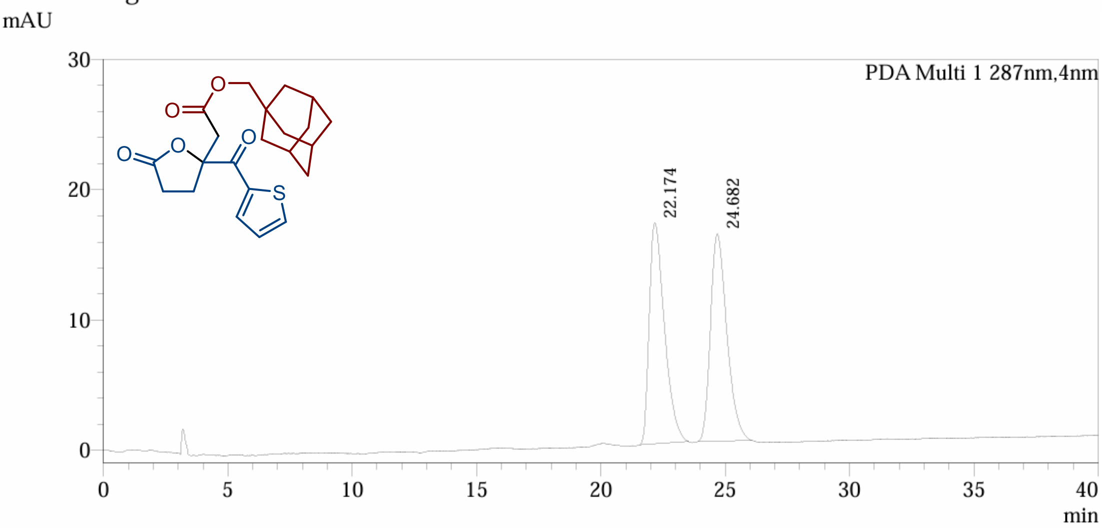

<Peak Table>

PDA Ch1 287nm

Peak#	Ret. Time	Area	Height	Aera%
1	22.174	721577	16947	49.755
2	24.682	728672	15907	50.245

<Chromatogram>

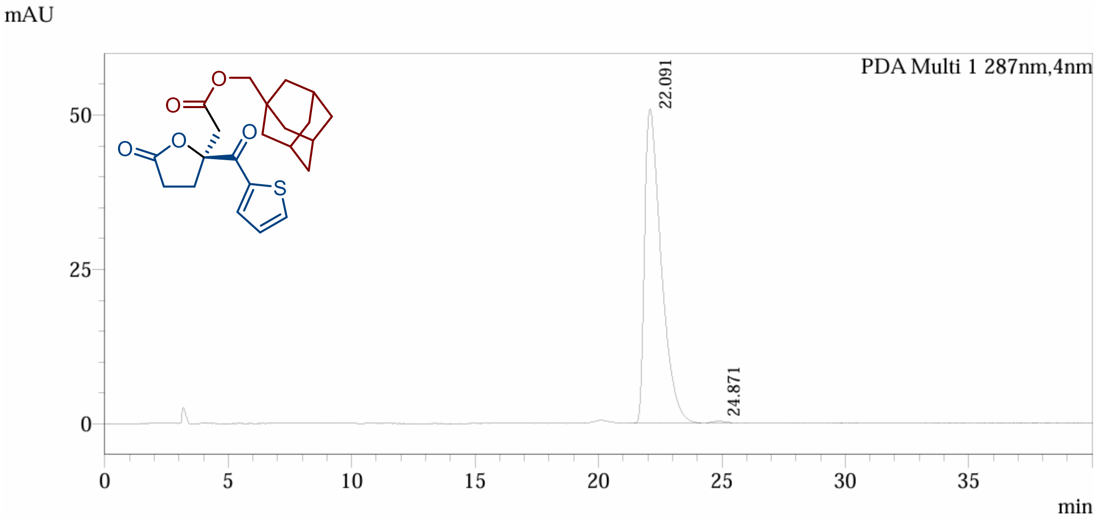

<Peak Table>

PDA Ch1 287nm

Peak#	Ret. Time	Area	Height	Aera%
1	22.091	2374997	50881	99.621
2	24.871	9026	261	0.379

3q

<Chromatogram>

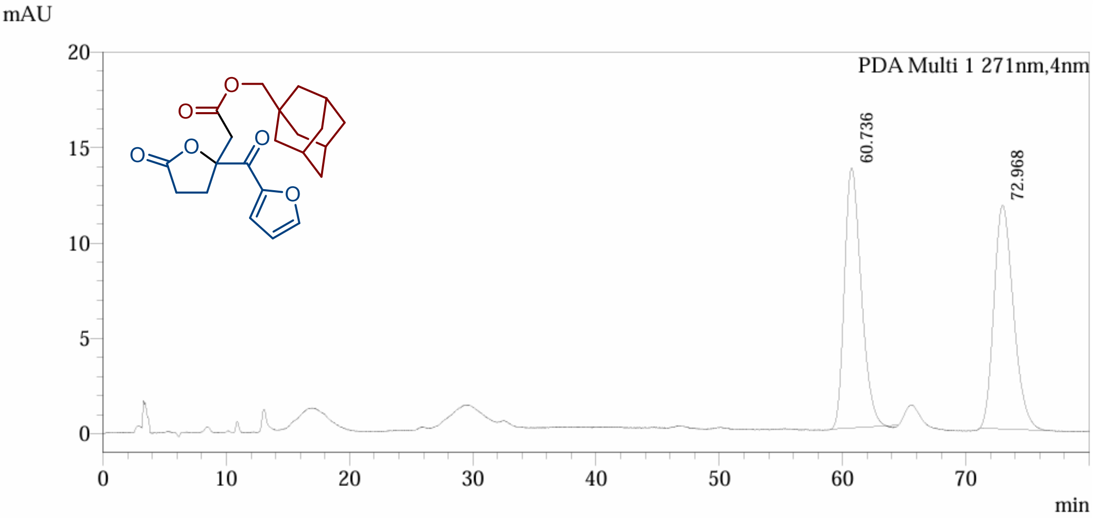

<Peak Table>

PDA Ch1 271nm

Peak#	Ret. Time	Area	Height	Aera%
1	60.736	1286186	13638	49.836
2	72.968	1294664	11728	50.164

<Chromatogram>

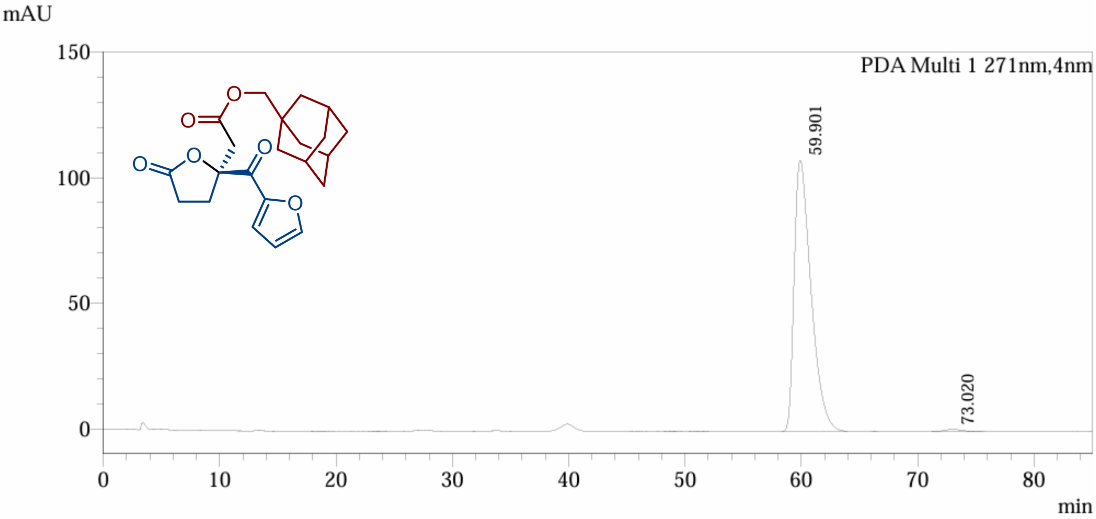

<Peak Table>

PDA Ch1 271nm

Peak#	Ret. Time	Area	Height	Aera%
1	59.901	10873943	108001	99.064
2	73.020	102728	955	0.936

3r

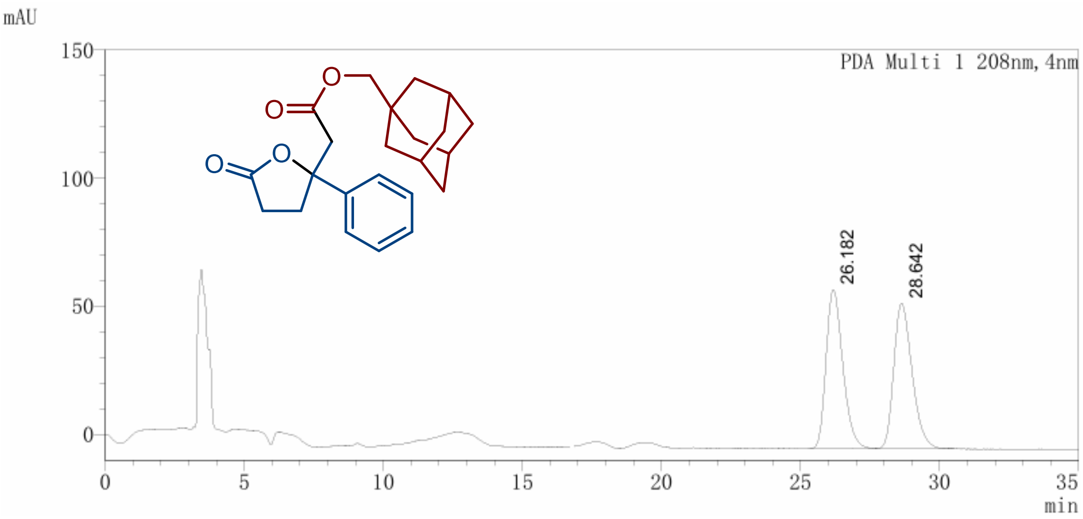

PDA Ch1 208nm

Peak	Ret. Time	Area	Area%
1	26. 182	2608220	50. 011
2	28. 642	2607062	49. 989

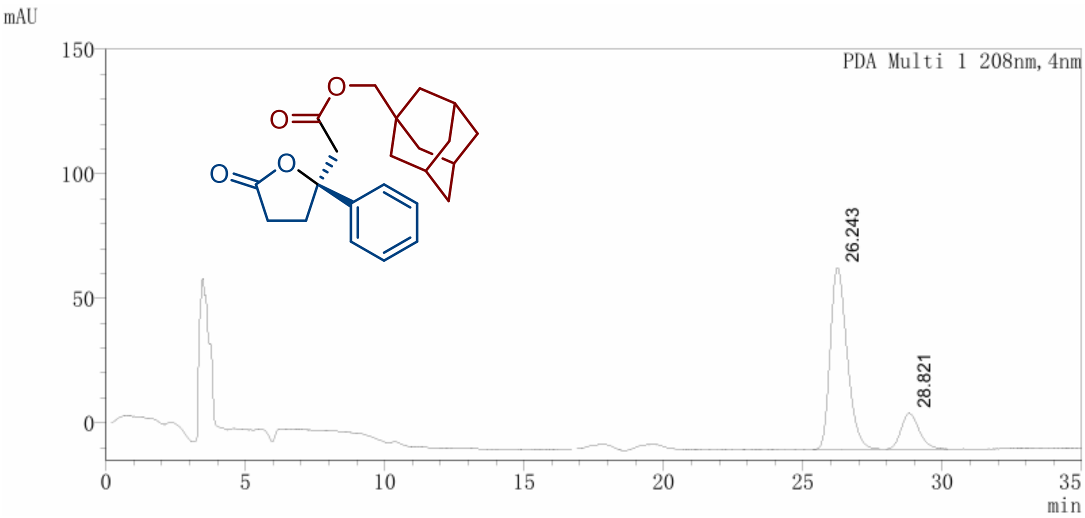

PDA Ch1 208nm

Peak	Ret. Time	Area	Area%
1	26. 243	3075652	82. 677
2	28. 821	644445	17. 323

3s

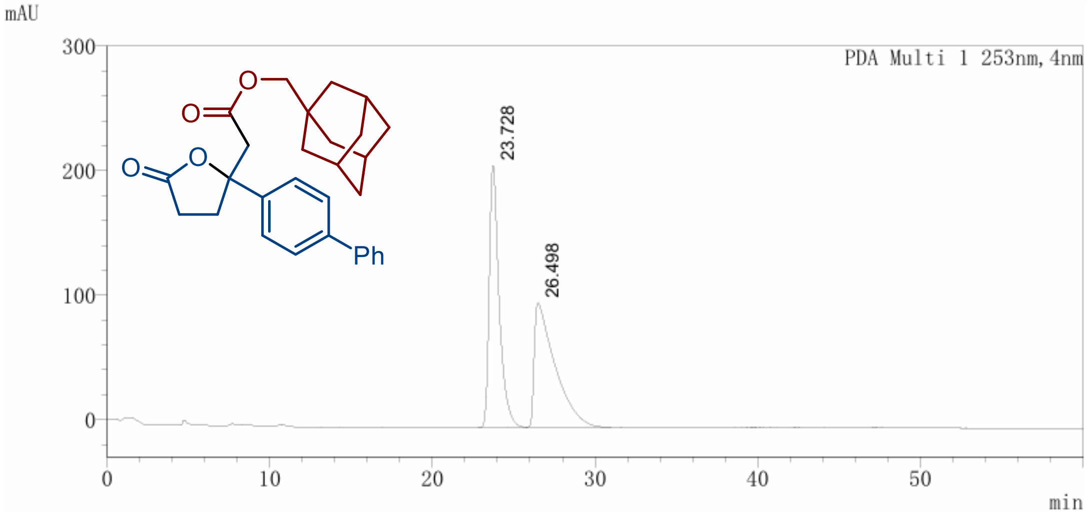

PDA Ch1 253nm

Peak	Ret. Time	Area	Area%
1	23.728	8972034	50.153
2	26.498	8917448	49.847

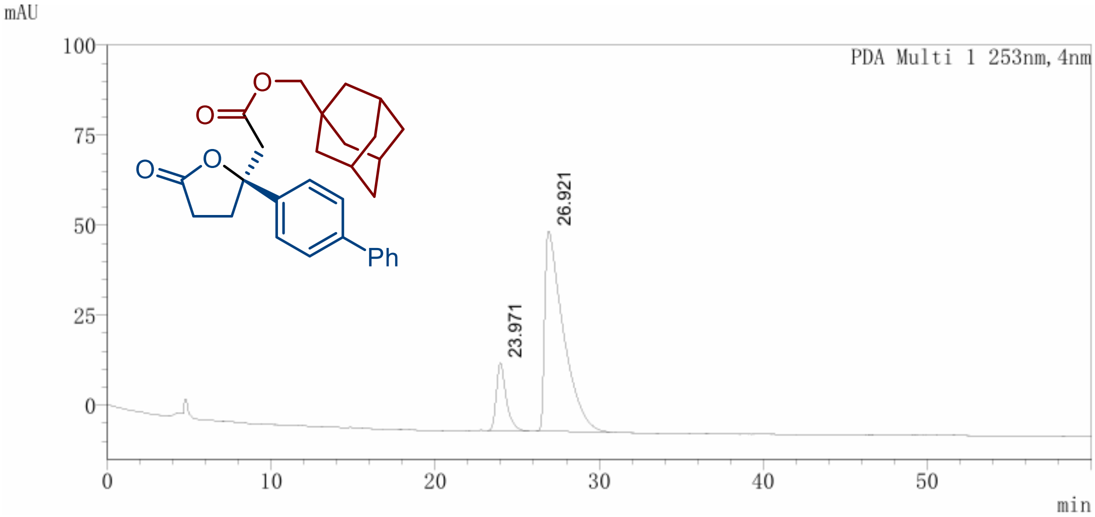

PDA Ch1 253nm

Peak	Ret. Time	Area	Area%
1	23.971	790620	15.233
2	26.921	4399486	84.767

3t

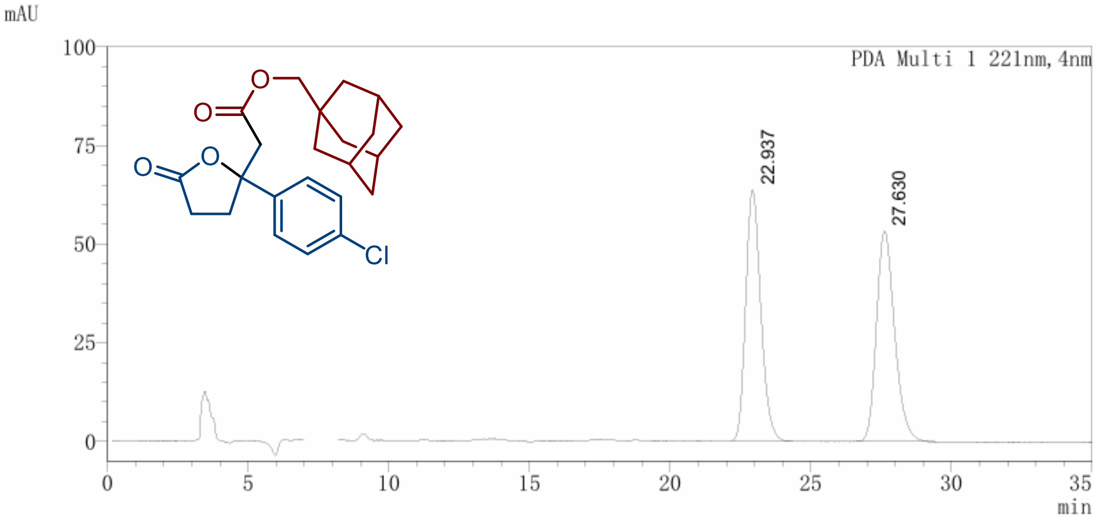

PDA Ch1 221nm

Peak	Ret. Time	Area	Area%
1	22. 937	2371302	49. 737
2	27. 630	2396374	50. 263

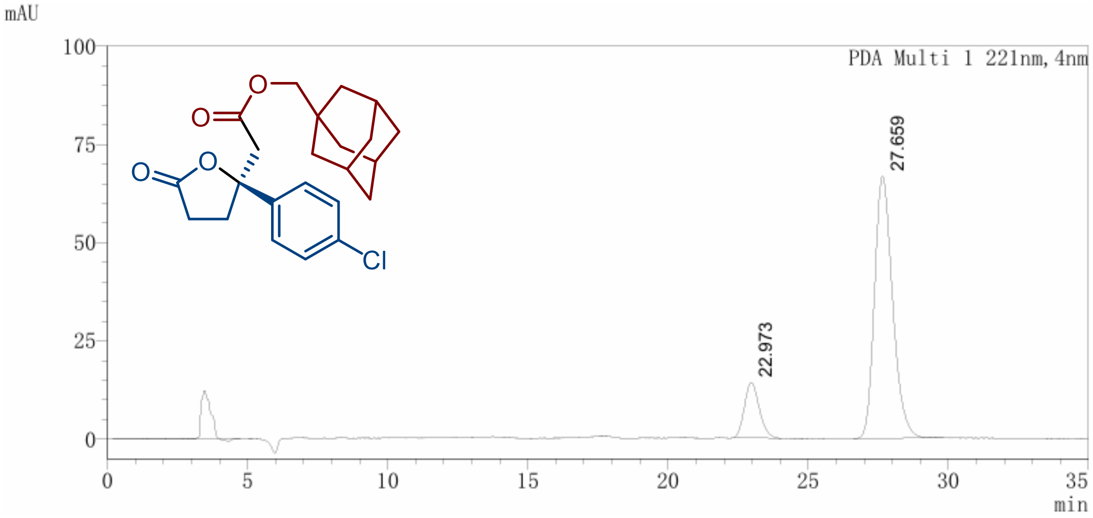

PDA Ch1 221nm

Peak	Ret. Time	Area	Area%
1	22. 973	514874	14. 653
2	27. 659	2998878	85. 347

3u

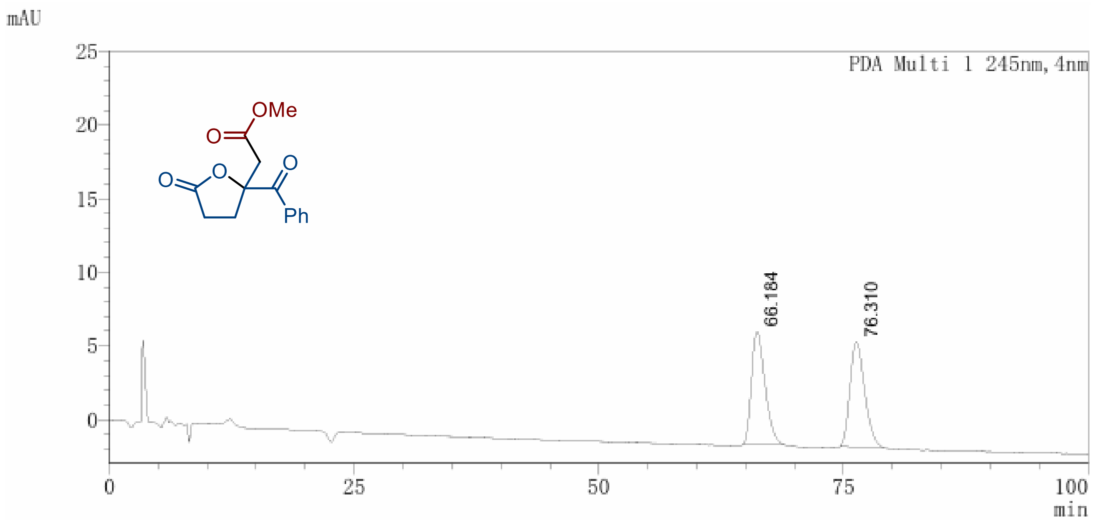

PDA Ch1 245nm

Peak	Ret. Time	Area	Area%
1	66. 184	740141	49. 743
2	76. 310	747782	50. 257

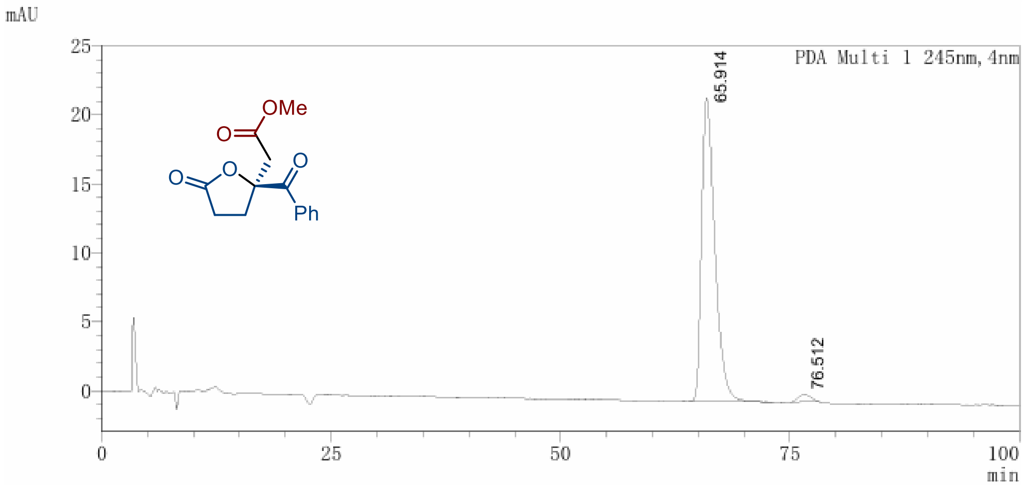

PDA Ch1 245nm

Peak	Ret. Time	Area	Area%
1	65. 914	2210625	97. 842
2	76. 512	48765	2. 158

3v

<Chromatogram>

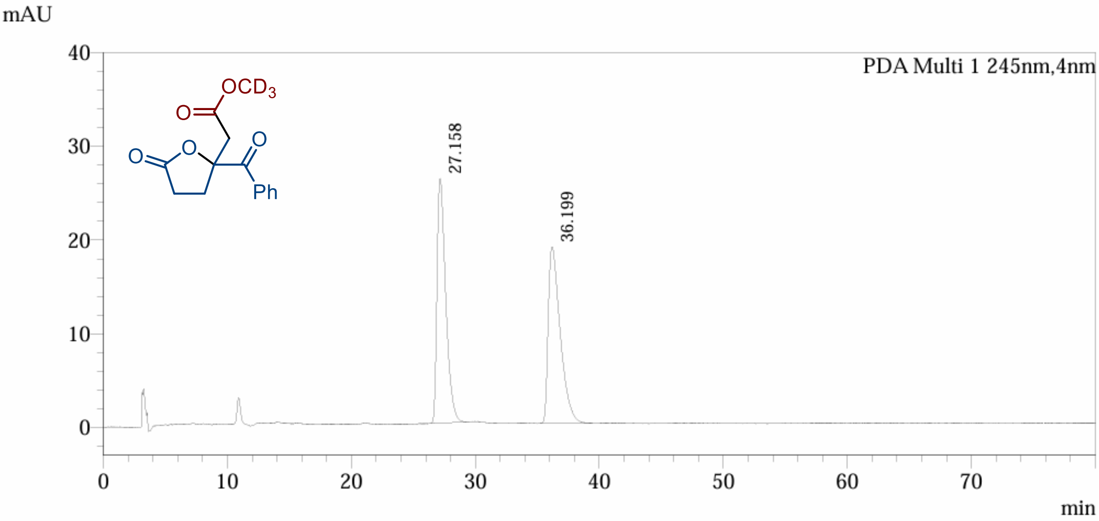

<Peak Table>

PDA Ch1 245nm

Peak#	Ret. Time	Area	Height	Aera%
1	27.158	1270242	26103	50.040
2	36.199	1268207	18851	49.960

<Chromatogram>

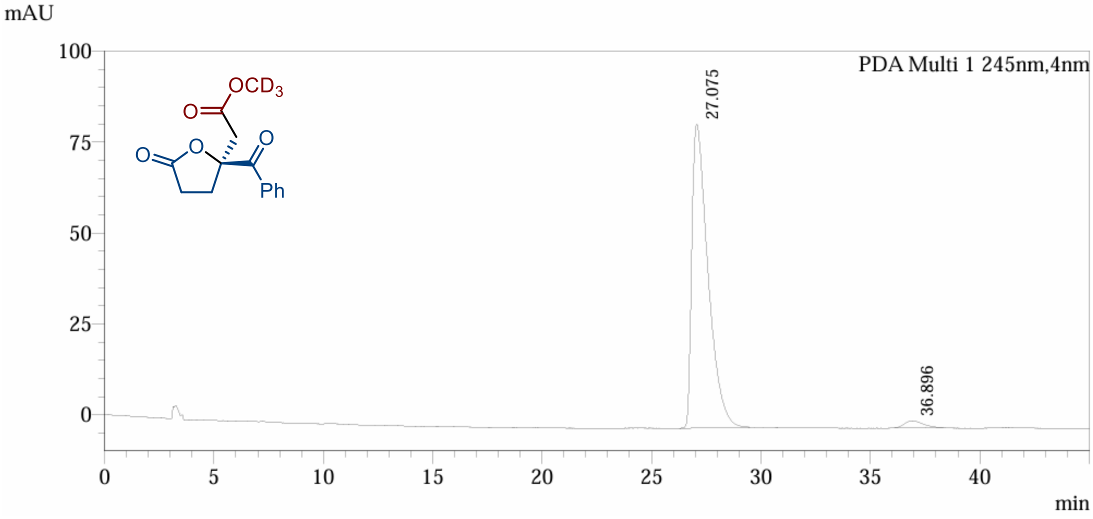

<Peak Table>

PDA Ch1 245nm

Peak#	Ret. Time	Area	Height	Aera%
1	27.075	4302459	83539	97.374
2	36.896	116023	1968	2.626

3w

<Chromatogram>

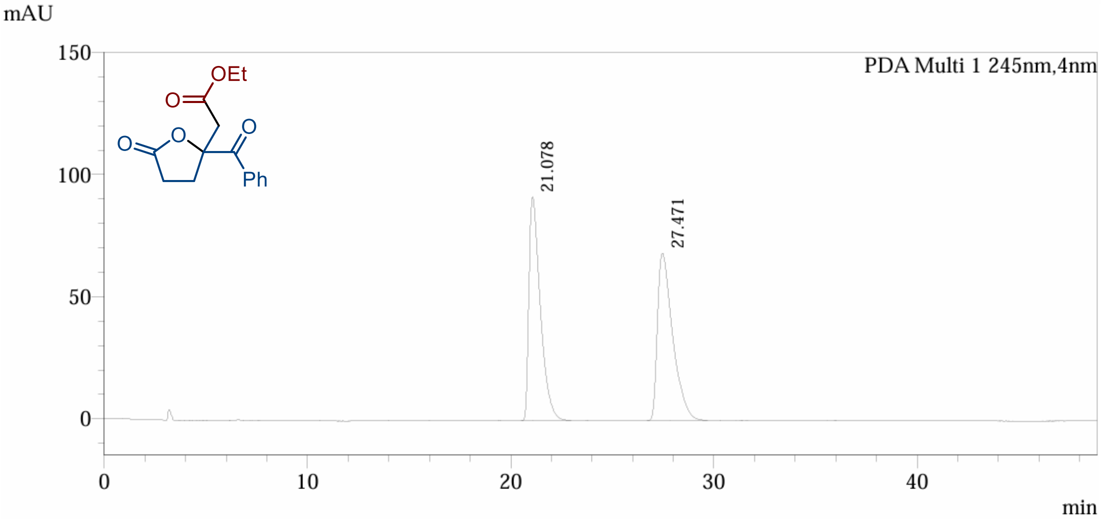

<Peak Table>

PDA Ch1 245nm

Peak#	Ret. Time	Area	Height	Aera%
1	21.078	3550398	91664	49.985
2	27.471	3552476	68467	50.015

<Chromatogram>

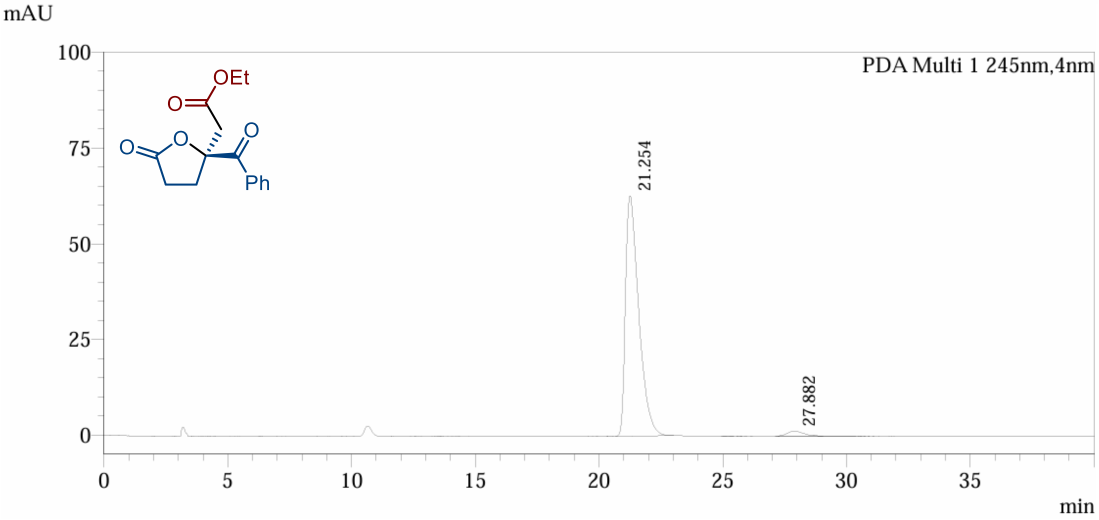

<Peak Table>

PDA Ch1 245nm

Peak#	Ret. Time	Area	Height	Aera%
1	21.254	2334079	62744	97.677
2	27.882	55511	1252	2.323

3x

<Chromatogram>

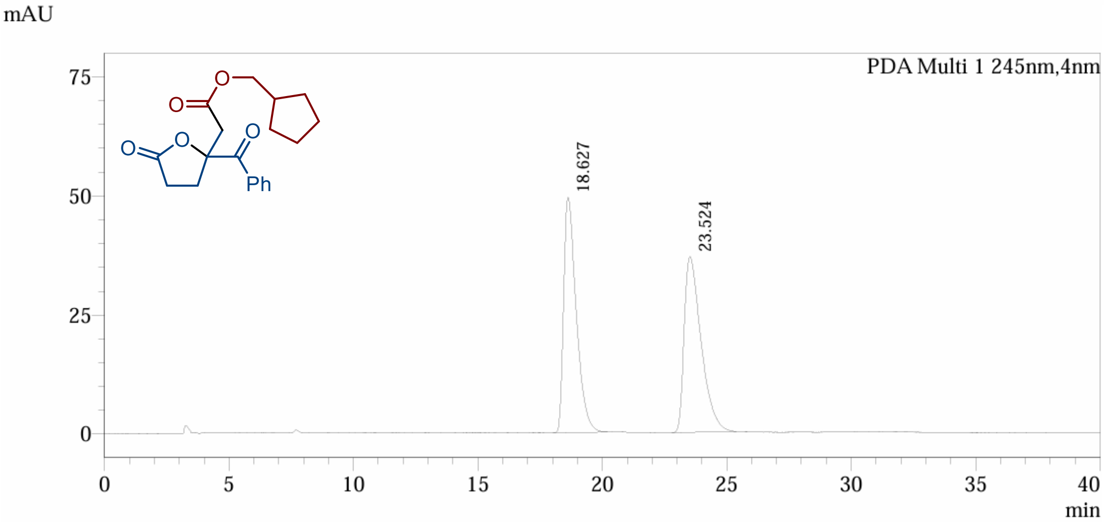

<Peak Table>

PDA Ch1 245nm

Peak#	Ret. Time	Area	Height	Aera%
1	18.627	1738312	49386	49.957
2	23.524	1741333	36908	50.043

<Chromatogram>

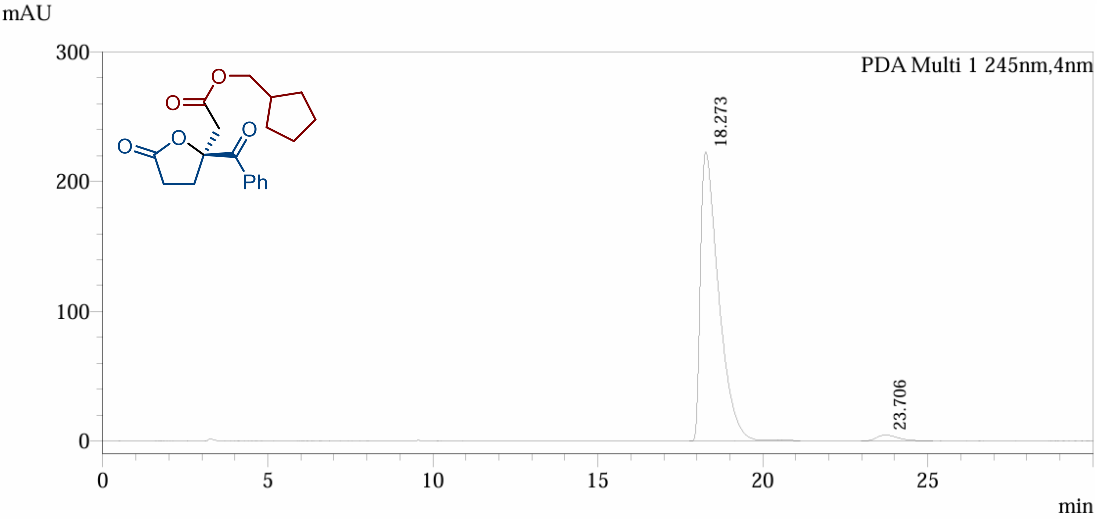

<Peak Table>

PDA Ch1 245nm

Peak#	Ret. Time	Area	Height	Aera%
1	18.273	8563423	222905	97.618
2	23.706	208952	4587	2.382

3y

<Chromatogram>

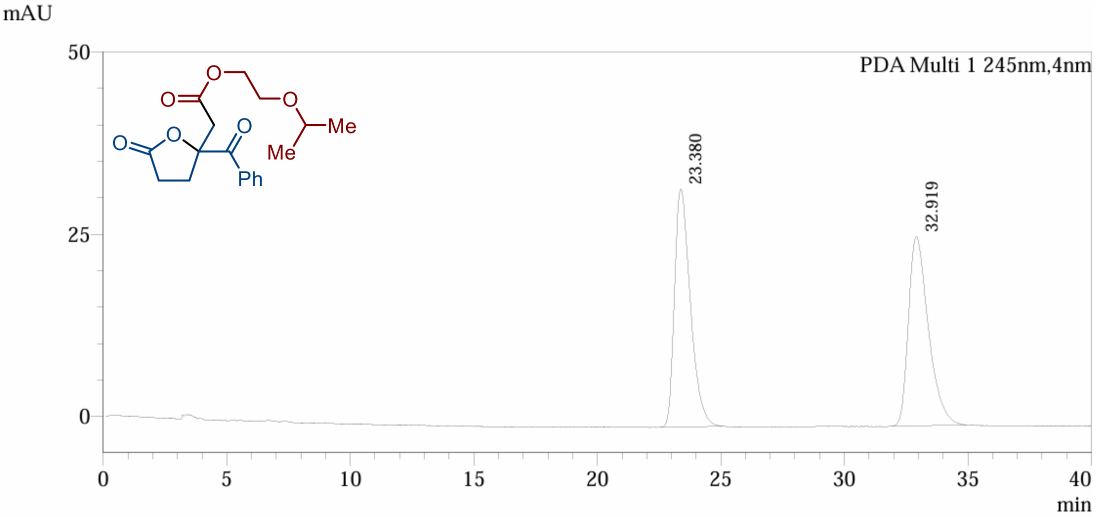

<Peak Table>

PDA Ch1 245nm

Peak#	Ret. Time	Area	Height	Aera%
1	23.380	1462563	32643	50.082
2	32.919	1457783	25931	49.918

<Chromatogram>

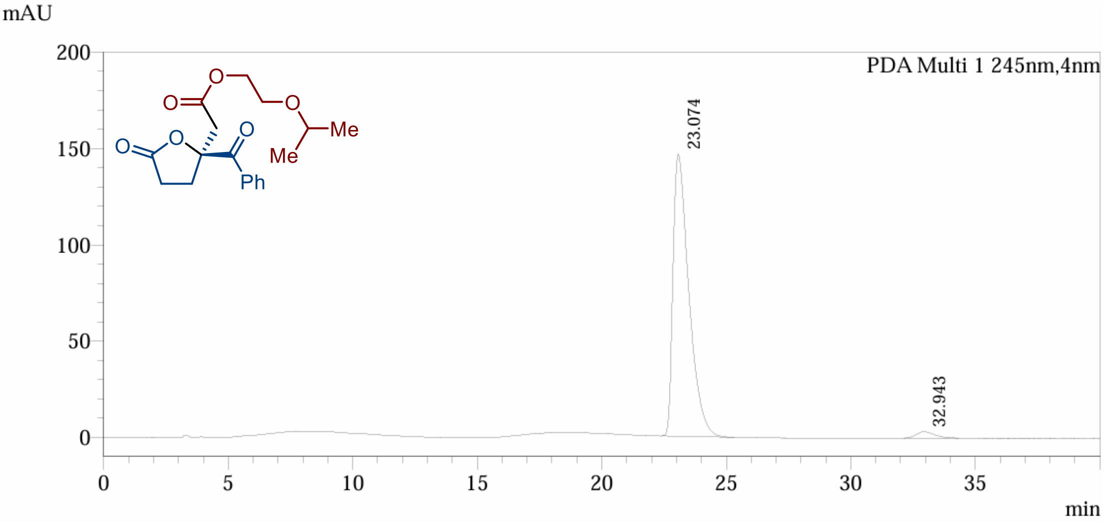

<Peak Table>

PDA Ch1 245nm

Peak#	Ret. Time	Area	Height	Aera%
1	23.074	6489576	146485	97.507
2	32.943	165911	3233	2.493

3z

<Chromatogram>

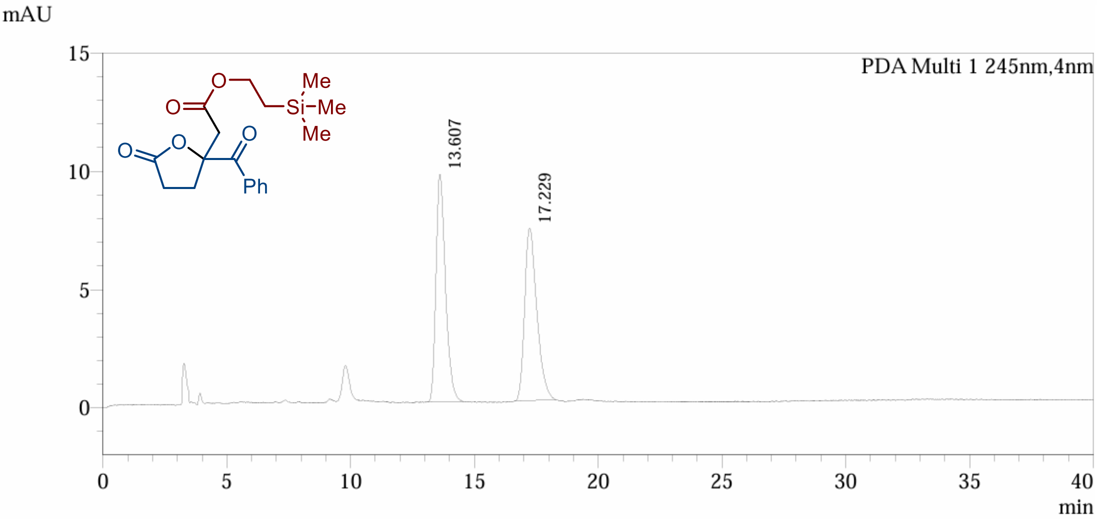

<Peak Table>

PDA Ch1 245nm

Peak#	Ret. Time	Area	Height	Aera%
1	13.607	256643	9612	50.678
2	17.229	249775	7286	49.322

<Chromatogram>

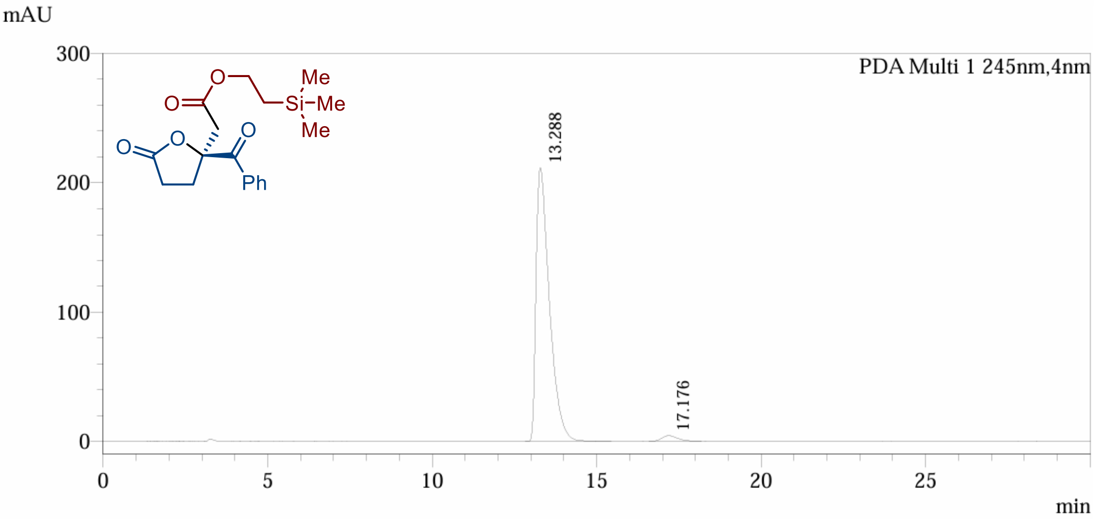

<Peak Table>

PDA Ch1 245nm

Peak#	Ret. Time	Area	Height	Aera%
1	13.288	5964220	211725	97.542
2	17.176	150324	4376	2.458

3aa

<Chromatogram>

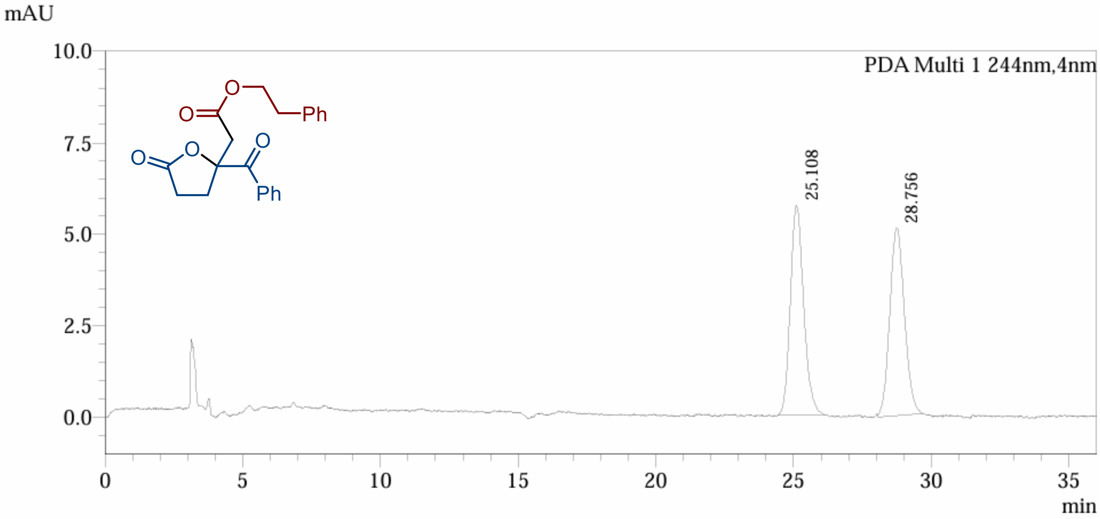

<Peak Table>

PDA Ch1 244nm

Peak#	Ret. Time	Area	Height	Aera%
1	25.108	193818	5734	50.553
2	28.756	189577	5120	49.447

<Chromatogram>

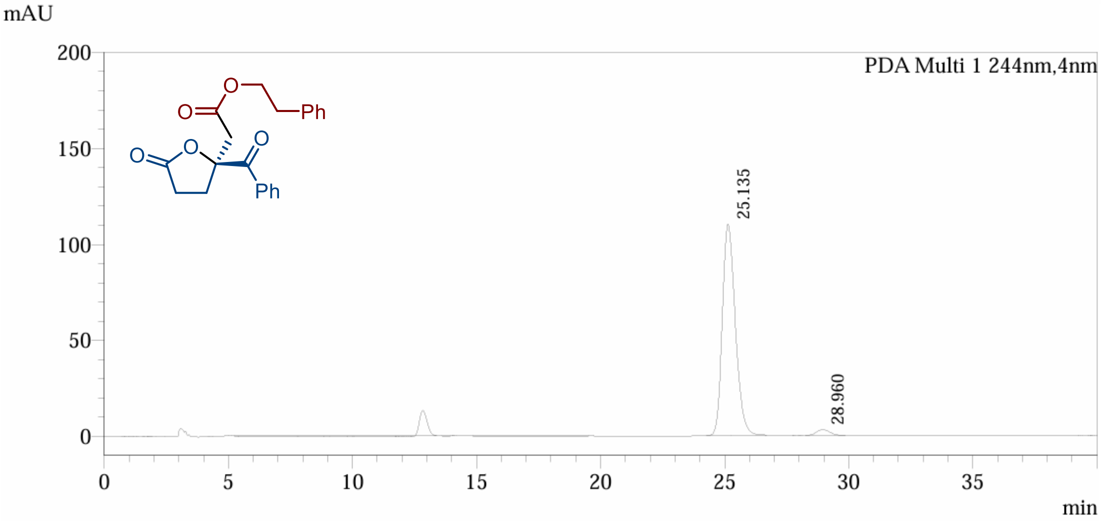

<Peak Table>

PDA Ch1 244nm

Peak#	Ret. Time	Area	Height	Aera%
1	25.135	3992270	109985	97.118
2	28.960	118484	3083	2.882

3ab

<Chromatogram>

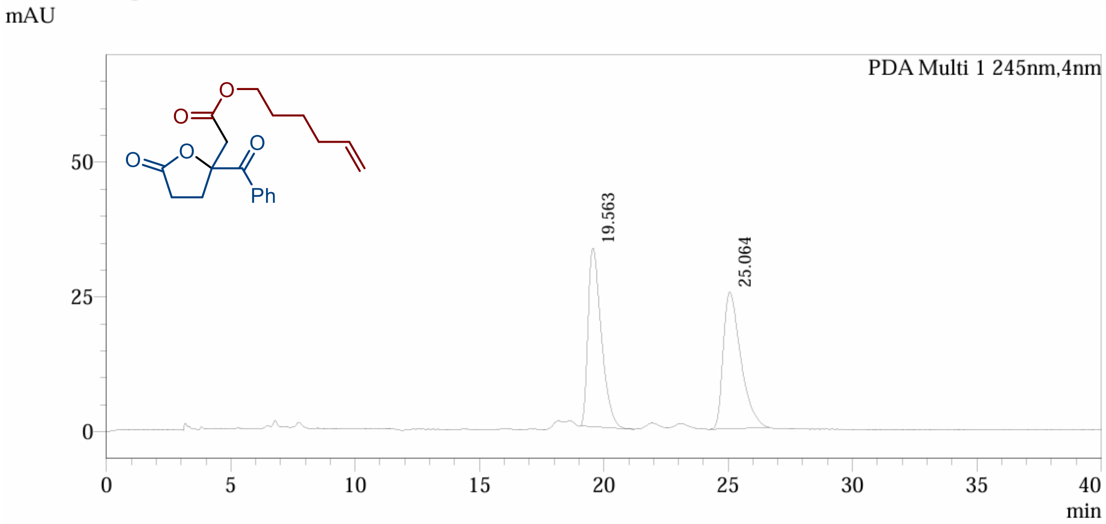

<Peak Table>

PDA Ch1 245nm

Peak#	Ret. Time	Area	Height	Aera%
1	19.563	1229897	33128	49.598
2	25.064	1249835	25333	50.402

<Chromatogram>

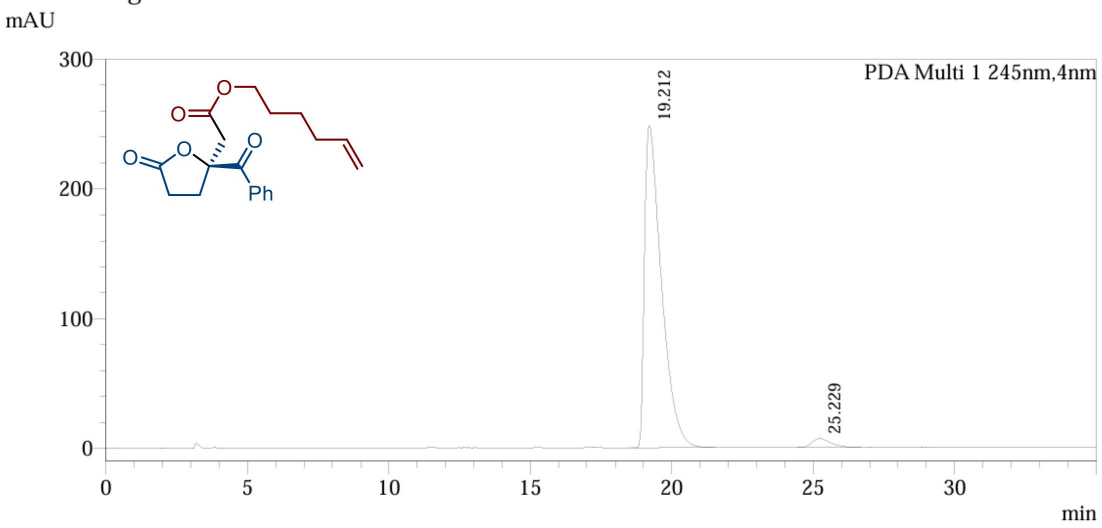

<Peak Table>

PDA Ch1 245nm

Peak#	Ret. Time	Area	Height	Aera%
1	19.212	10310307	248231	96.933
2	25.229	326265	6870	3.067

3ac

<Chromatogram>

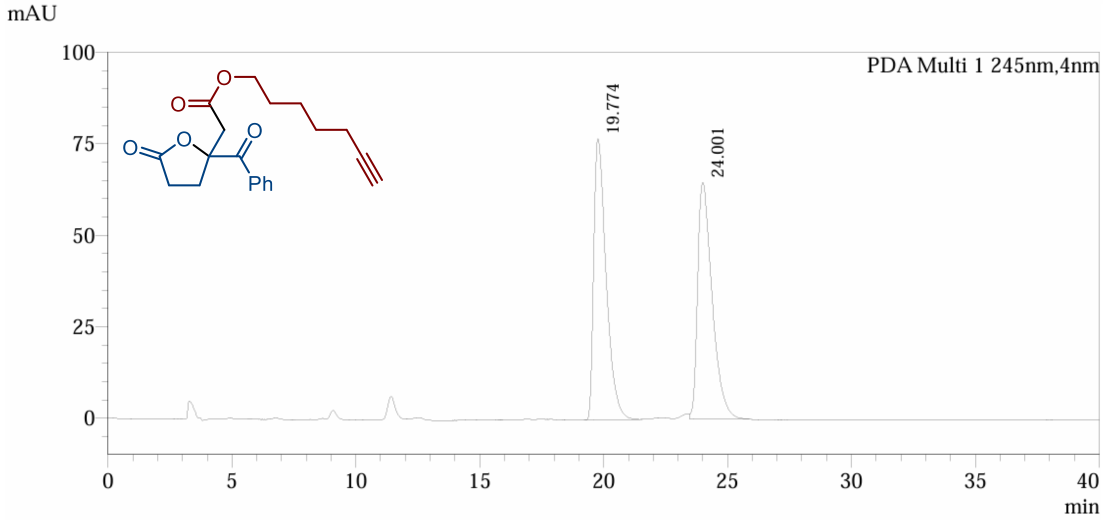

<Peak Table>

PDA Ch1 245nm

Peak#	Ret. Time	Area	Height	Aera%
1	19.774	2673363	76847	50.419
2	24.001	2628885	64583	49.581

<Chromatogram>

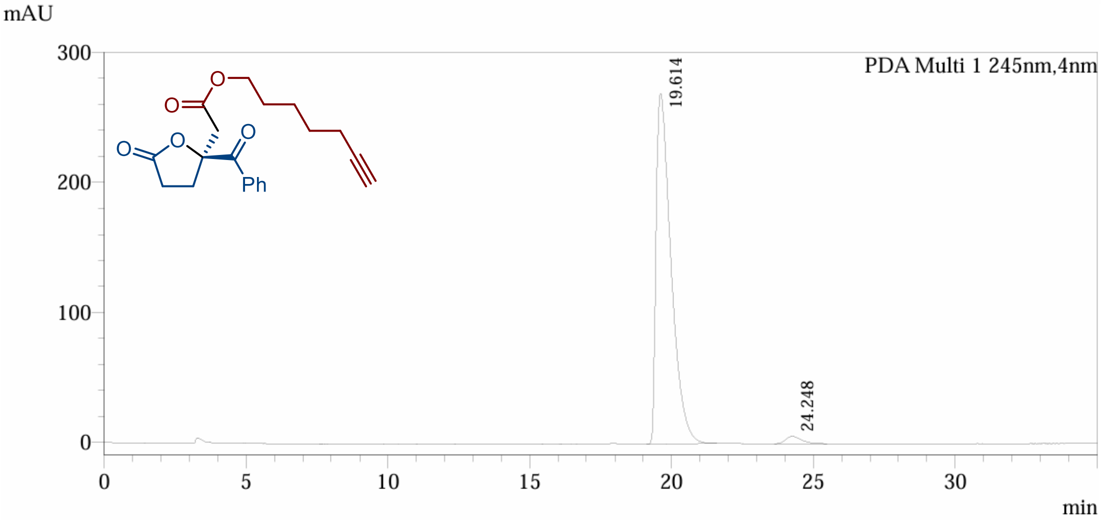

<Peak Table>

PDA Ch1 245nm

Peak#	Ret. Time	Area	Height	Aera%
1	19.614	9896369	269533	97.819
2	24.248	220632	5699	2.181

3ad

<Chromatogram>

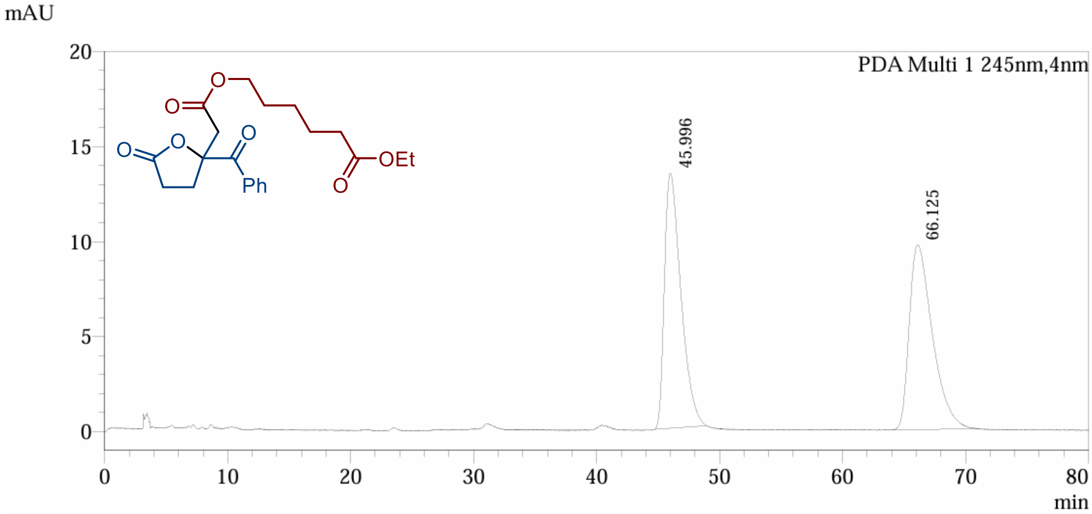

<Peak Table>

PDA Ch1 245nm

Peak#	Ret. Time	Area	Height	Aera%
1	45.996	1252158	13423	49.541
2	66.125	1275382	9729	50.459

<Chromatogram>

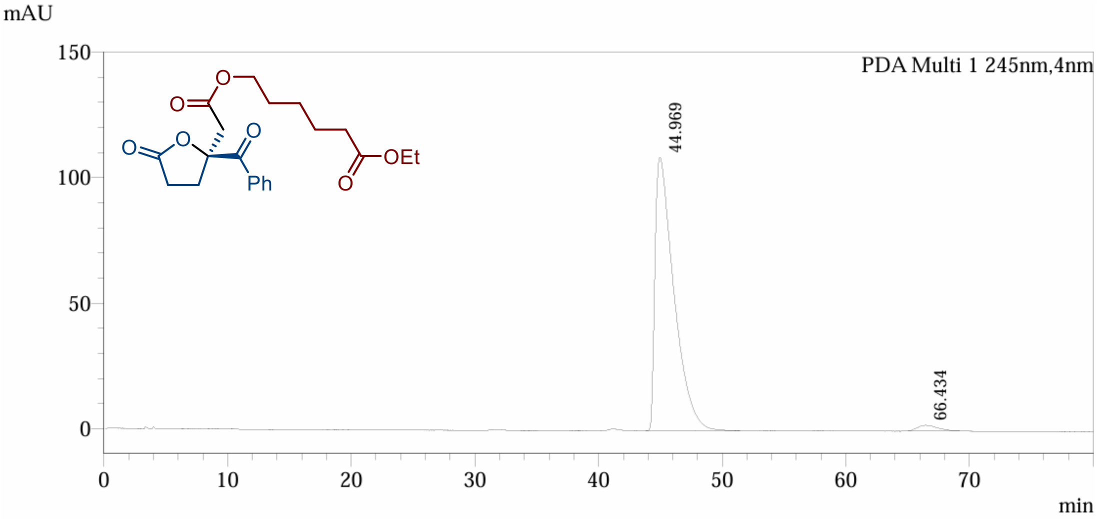

<Peak Table>

PDA Ch1 245nm

Peak#	Ret. Time	Area	Height	Aera%
1	44.969	11594753	108784	97.871
2	66.434	252245	2267	2.129

3ae

<Chromatogram>

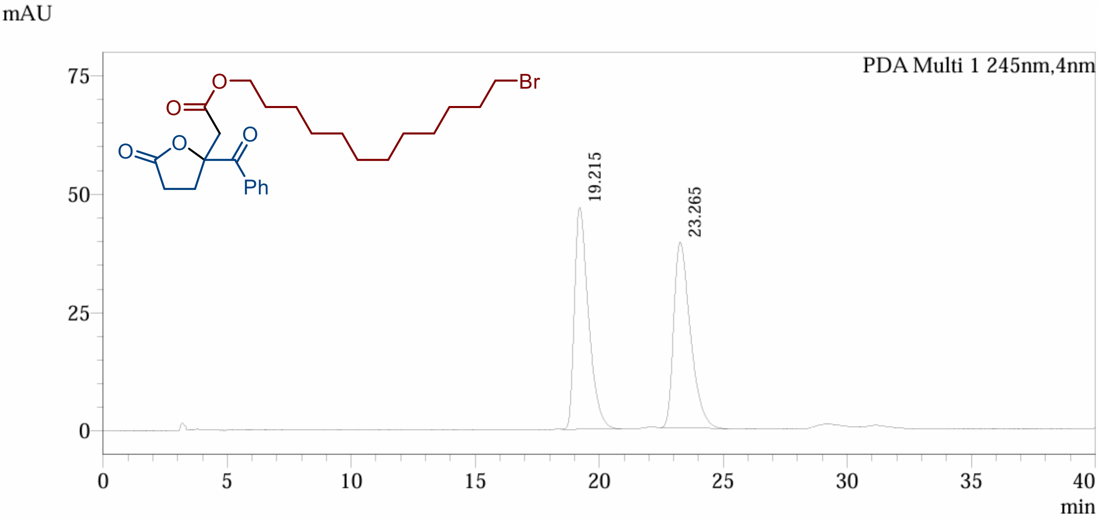

<Peak Table>

PDA Ch1 245nm

Peak#	Ret. Time	Area	Height	Aera%
1	19.215	1850454	46785	50.161
2	23.265	1838566	39236	49.839

<Chromatogram>

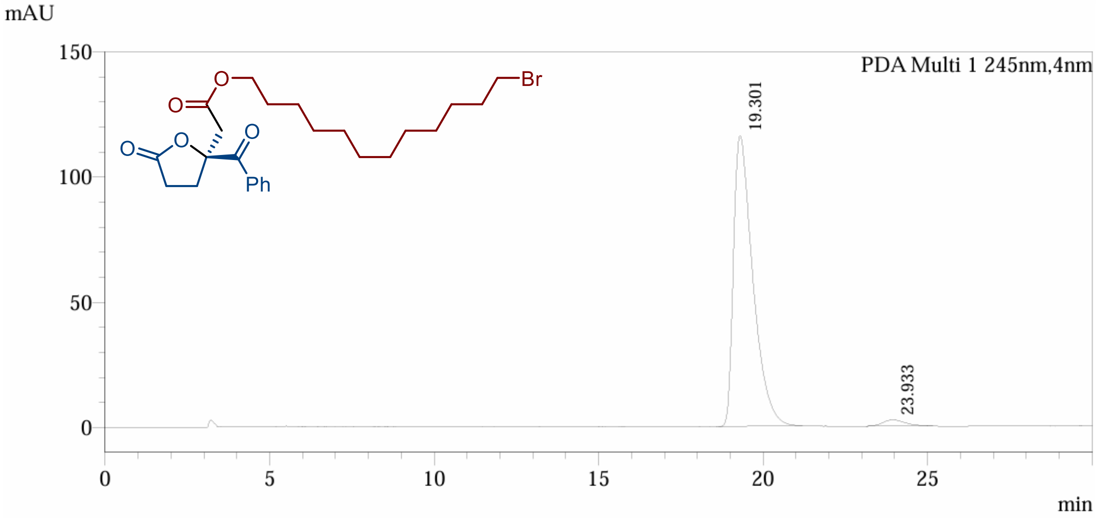

<Peak Table>

PDA Ch1 245nm

Peak#	Ret. Time	Area	Height	Aera%
1	19.301	4743155	115923	97.586
2	23.933	117357	2482	2.414

3af

<Chromatogram>

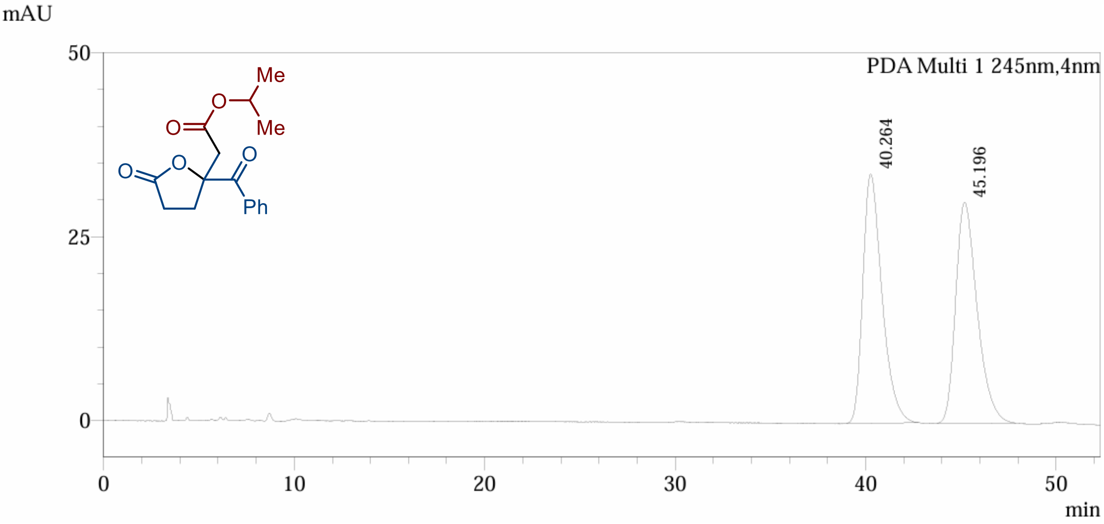

<Peak Table>

PDA Ch1 245nm

Peak#	Ret. Time	Area	Height	Aera%
1	40.264	2375450	33850	50.158
2	45.196	2360452	30008	49.842

<Chromatogram>

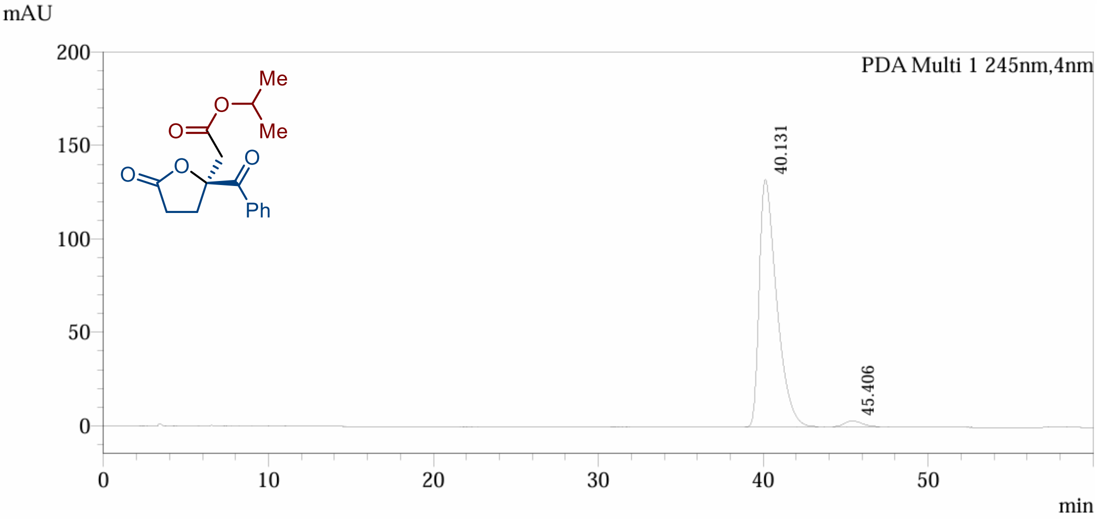

<Peak Table>

PDA Ch1 245nm

Peak#	Ret. Time	Area	Height	Aera%
1	40.131	9721972	132248	97.701
2	45.406	228814	3105	2.299

3ag

<Chromatogram>

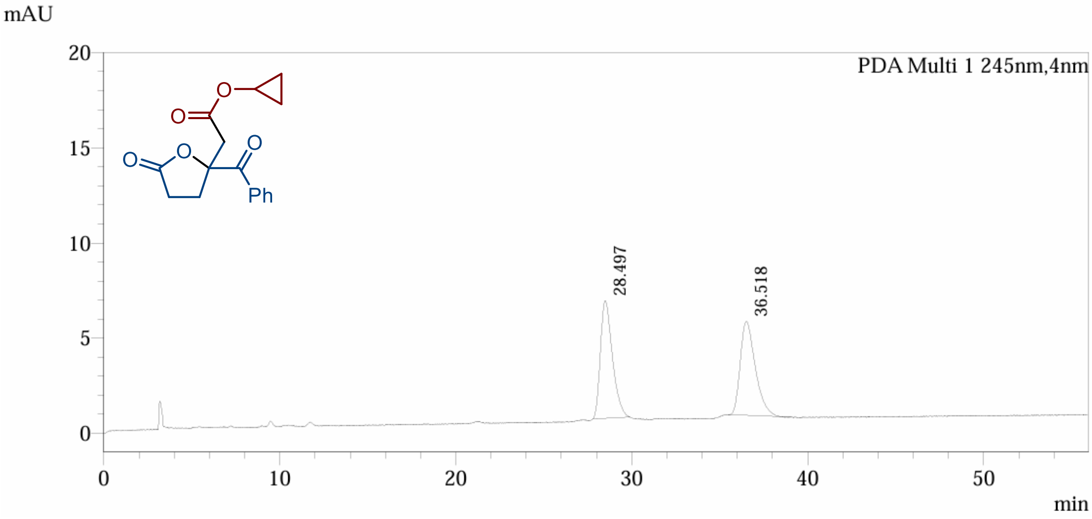

<Peak Table>

PDA Ch1 245nm

Peak#	Ret. Time	Area	Height	Aera%
1	28.497	293978	6176	50.006
2	36.518	293904	4934	49.994

<Chromatogram>

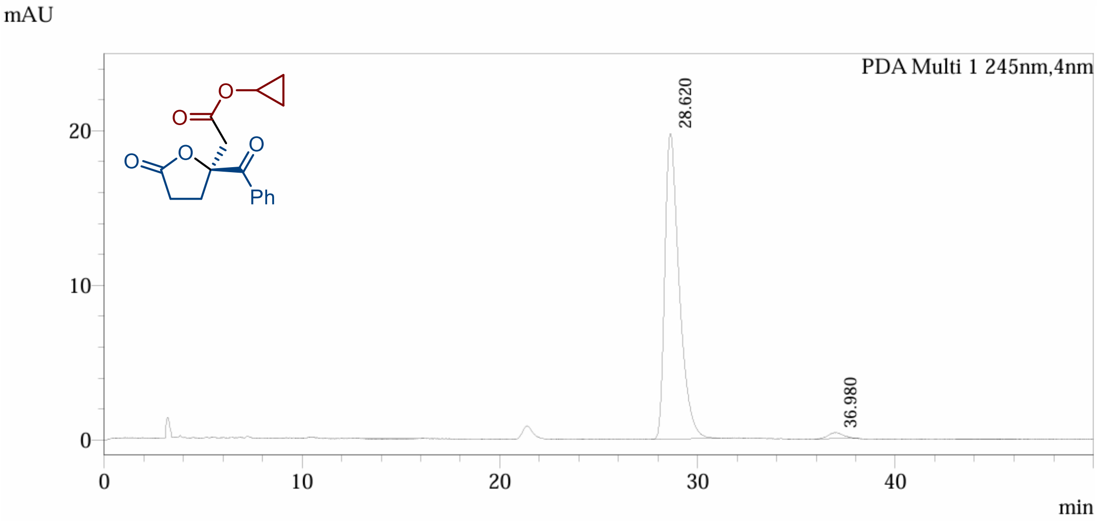

<Peak Table>

PDA Ch1 245nm

Peak#	Ret. Time	Area	Height	Aera%
1	28.620	996617	19775	97.887
2	36.980	21516	396	2.113

3ah

<Chromatogram>

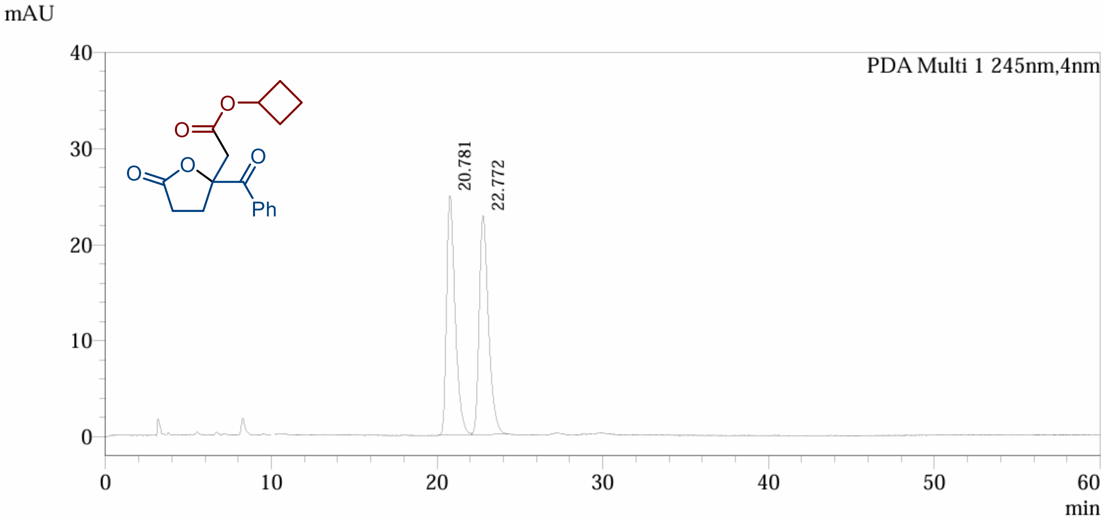

<Peak Table>

PDA Ch1 245nm

Peak#	Ret. Time	Area	Height	Aera%
1	20.781	897235	24916	50.176
2	22.772	890951	22796	49.824

<Chromatogram>

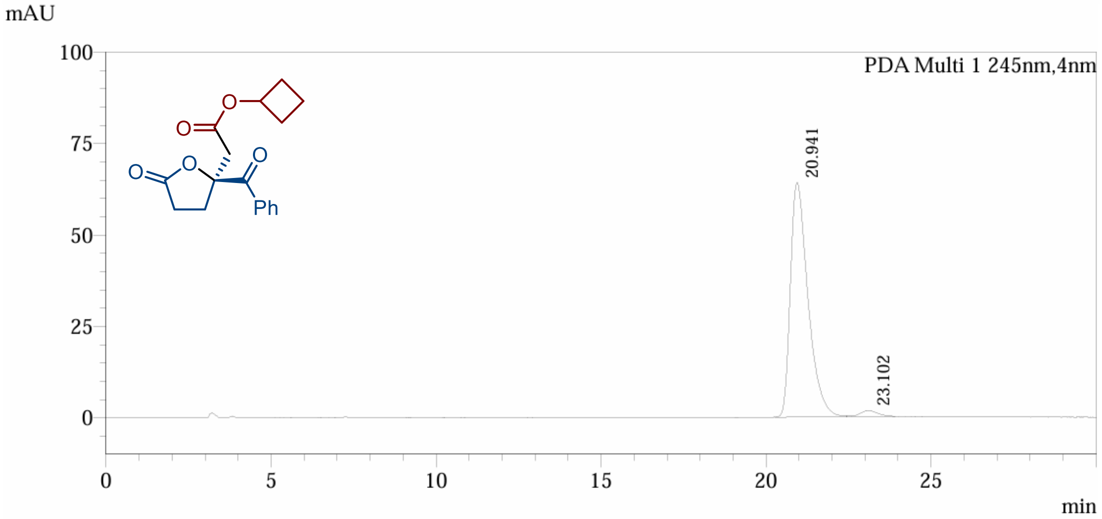

<Peak Table>

PDA Ch1 245nm

Peak#	Ret. Time	Area	Height	Aera%
1	20.941	2371337	64113	97.441
2	23.102	62266	1647	2.559

3ai

<Chromatogram>

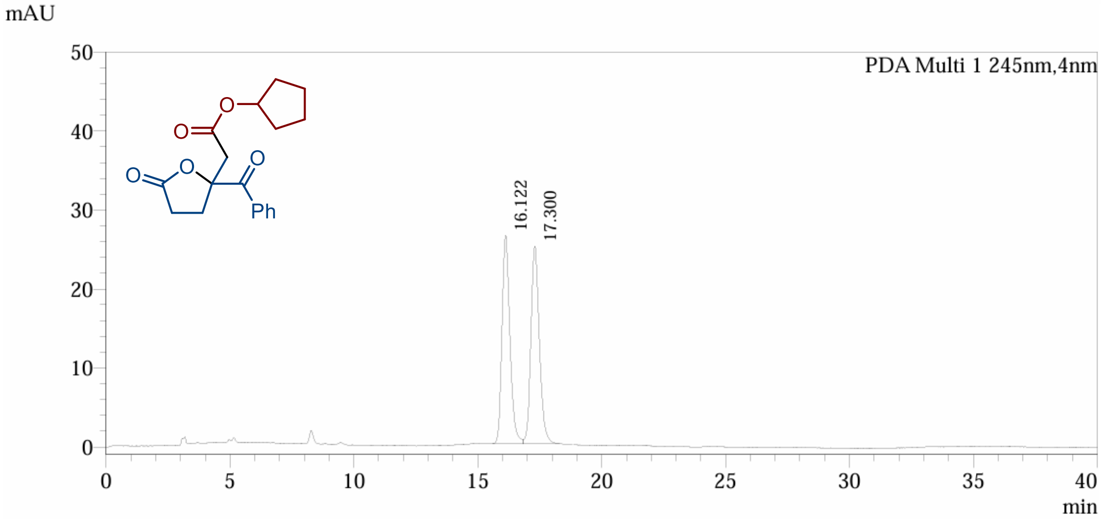

<Peak Table>

PDA Ch1 245nm

Peak#	Ret. Time	Area	Height	Aera%
1	16.122	590708	26397	49.702
2	17.300	597791	25032	50.298

<Chromatogram>

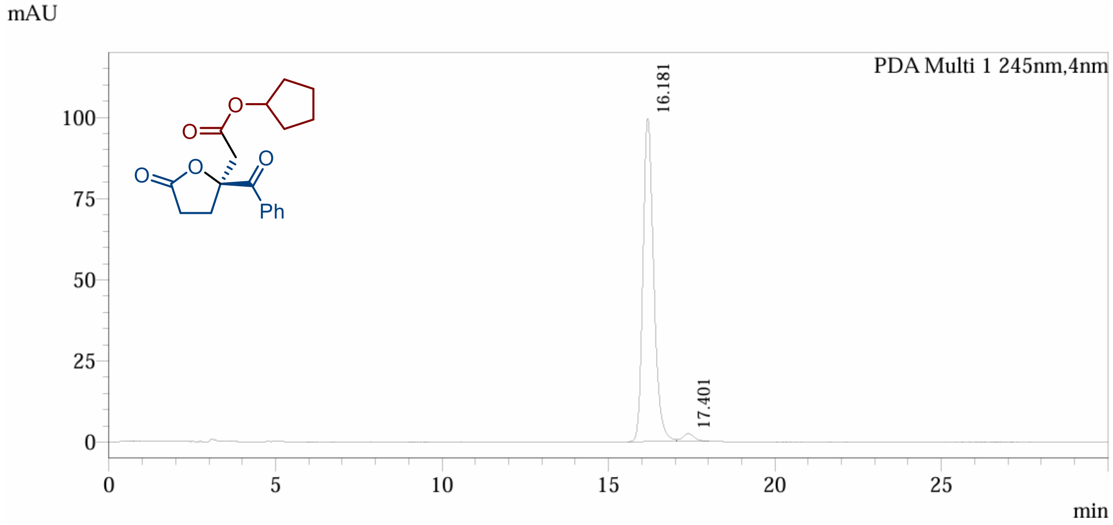

<Peak Table>

PDA Ch1 245nm

Peak#	Ret. Time	Area	Height	Aera%
1	16.181	2233527	99515	97.425
2	17.401	59033	2378	2.575

3aj

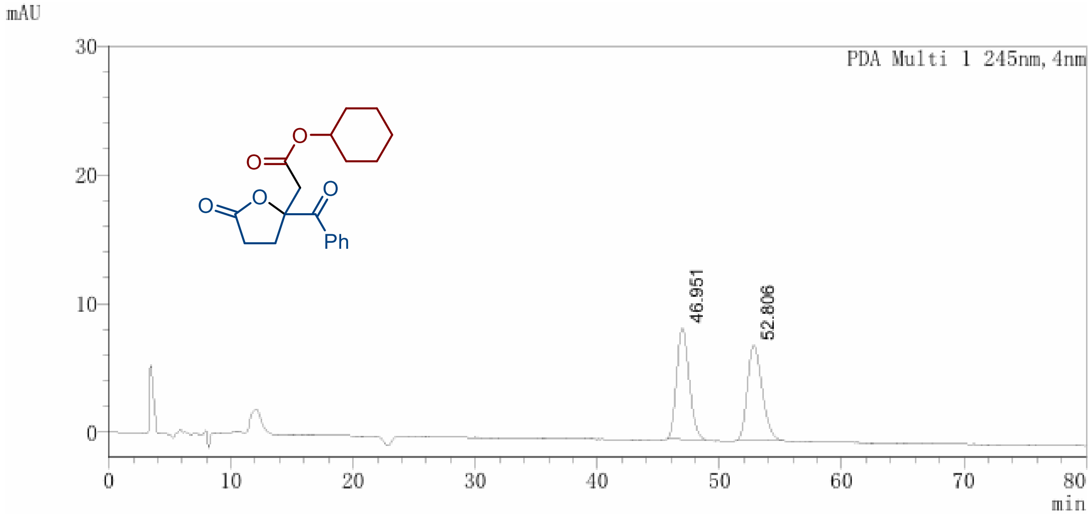

PDA Ch1 245nm

Peak	Ret. Time	Area	Area%
1	46.951	623280	50.117
2	52.806	620357	49.883

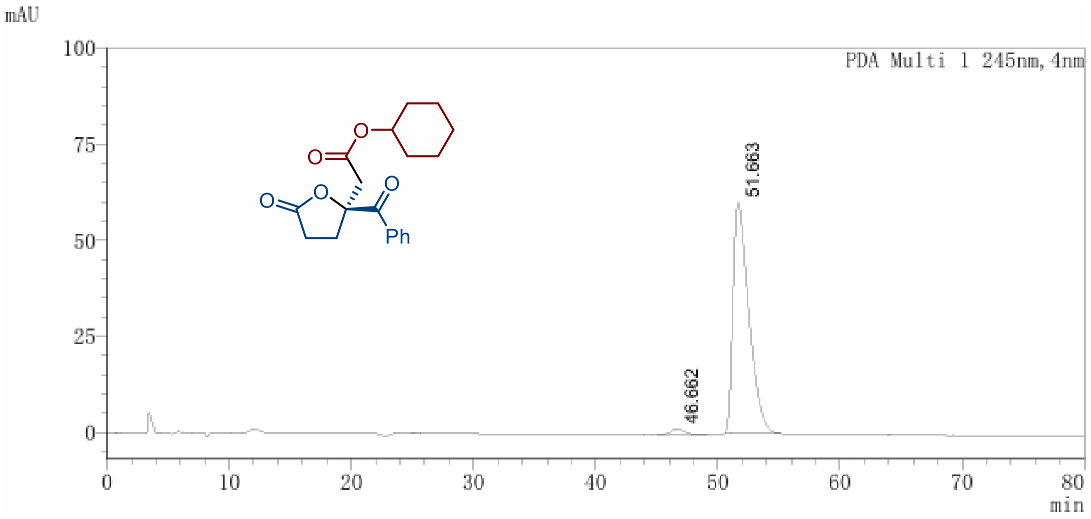

PDA Ch1 245nm

Peak	Ret. Time	Area	Area%
1	46.662	107679	1.936
2	51.663	5453073	98.064

3ak

<Chromatogram>

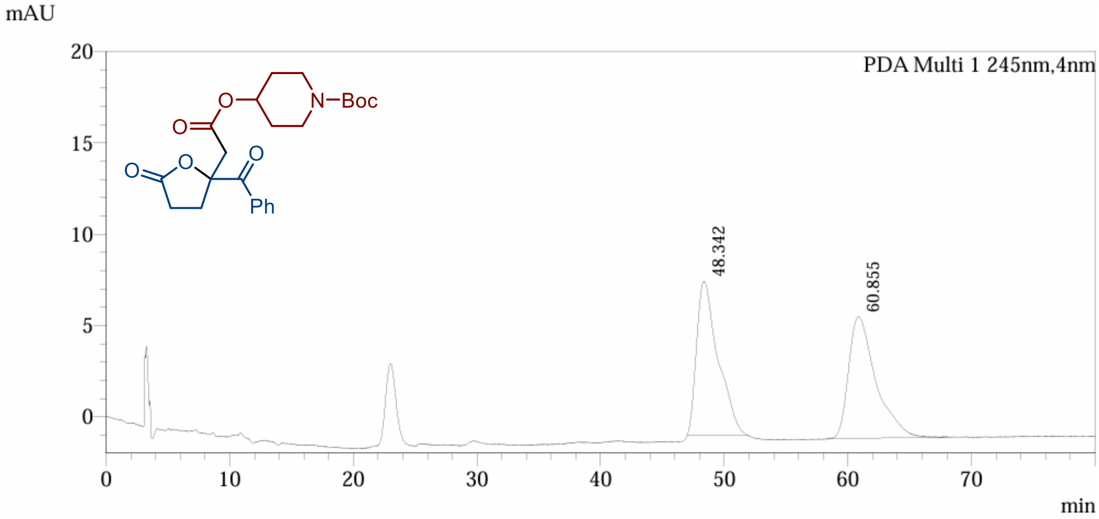

<Peak Table>

PDA Ch1 245nm

Peak#	Ret. Time	Area	Height	Aera%
1	48.342	1024854	8450	50.029
2	60.855	1023684	6672	49.971

<Chromatogram>

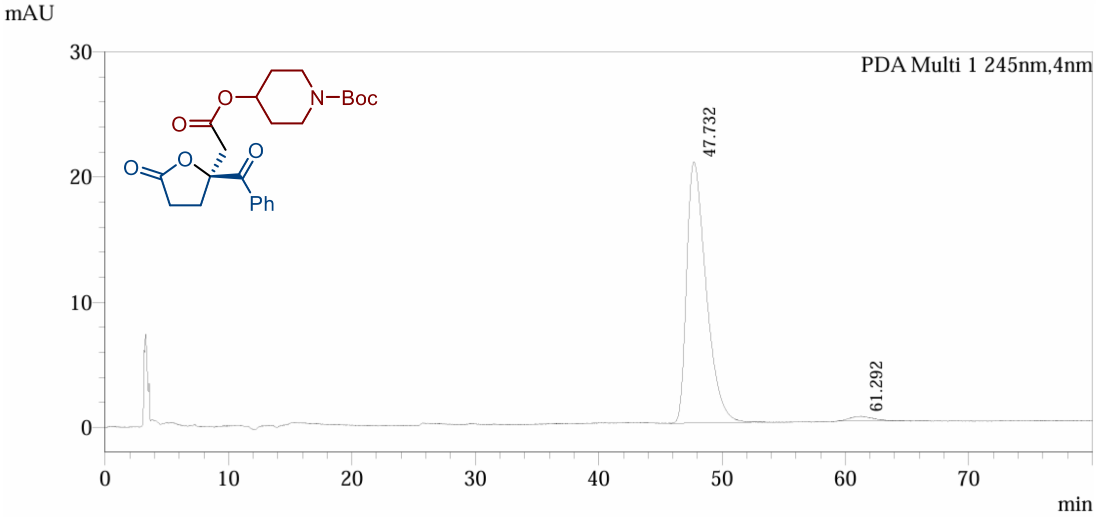

<Peak Table>

PDA Ch1 245nm

Peak#	Ret. Time	Area	Height	Aera%
1	47.732	2283823	20844	97.817
2	61.292	50979	394	2.183

3al

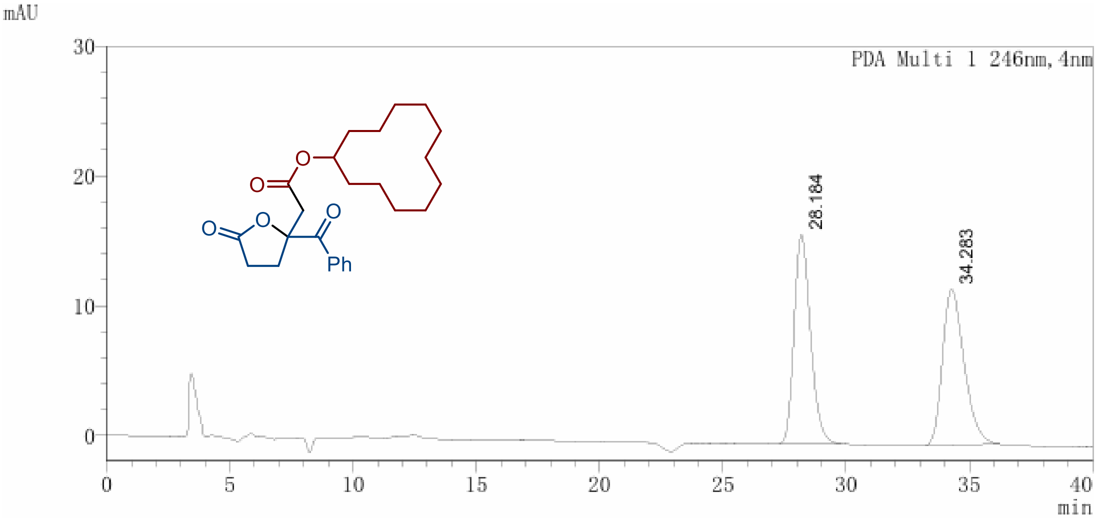

PDA Ch1 246nm

Peak	Ret. Time	Area	Area%
1	28.184	744956	50.466
2	34.283	731207	49.534

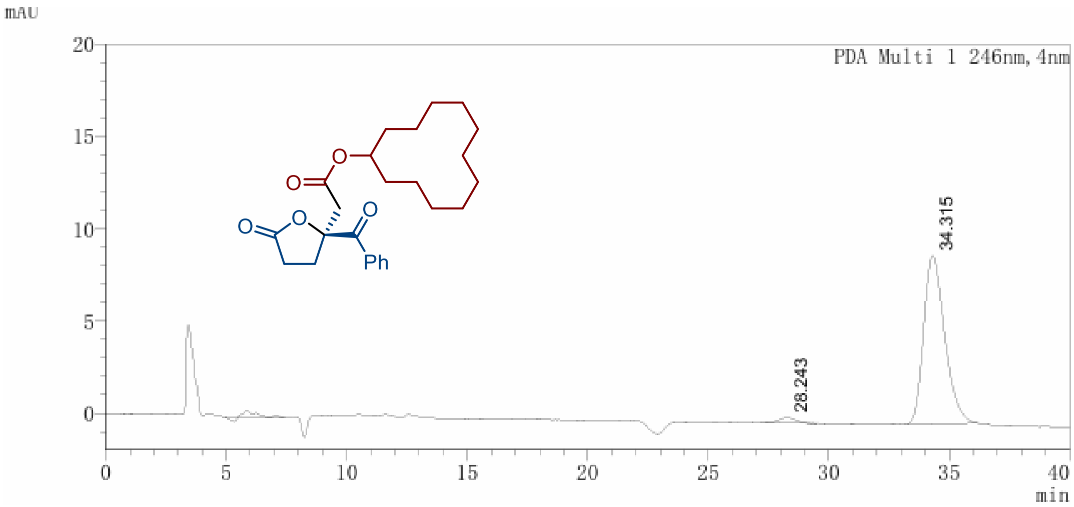

PDA Ch1 246nm

Peak	Ret. Time	Area	Area%
1	28.243	13279	2.341
2	34.315	553919	97.659

3am

<Chromatogram>

mAU

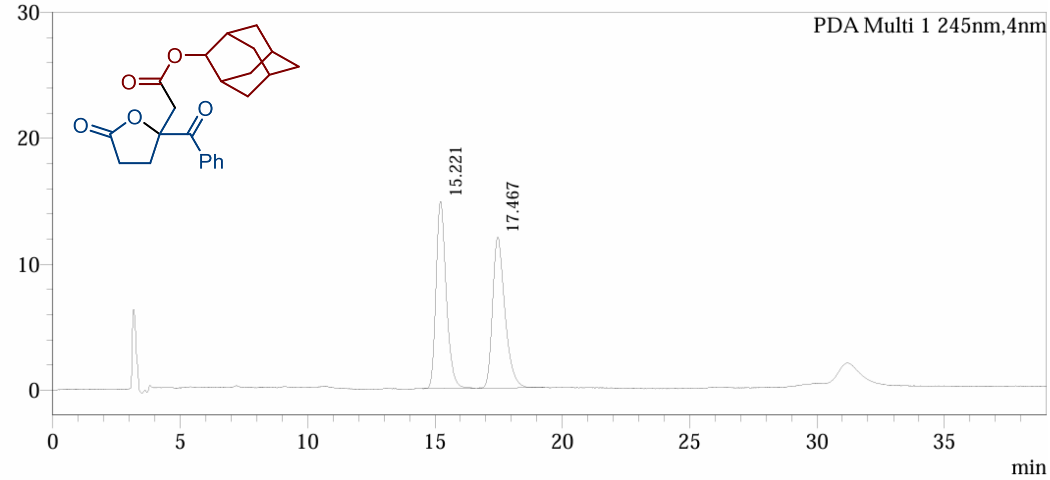

<Peak Table>

PDA Ch1 245nm

Peak#	Ret. Time	Area	Height	Aera%
1	15.221	409646	14864	50.172
2	17.467	406841	11973	49.828

<Chromatogram>

mAU

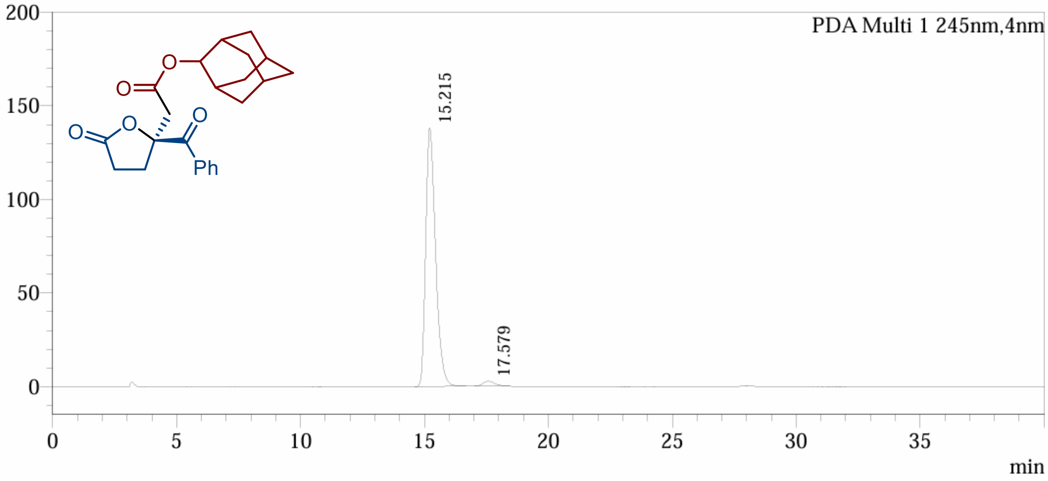

<Peak Table>

PDA Ch1 245nm

Peak#	Ret. Time	Area	Height	Aera%
1	15.215	3807223	138006	97.790
2	17.579	86058	2629	2.210

3an

<Chromatogram>

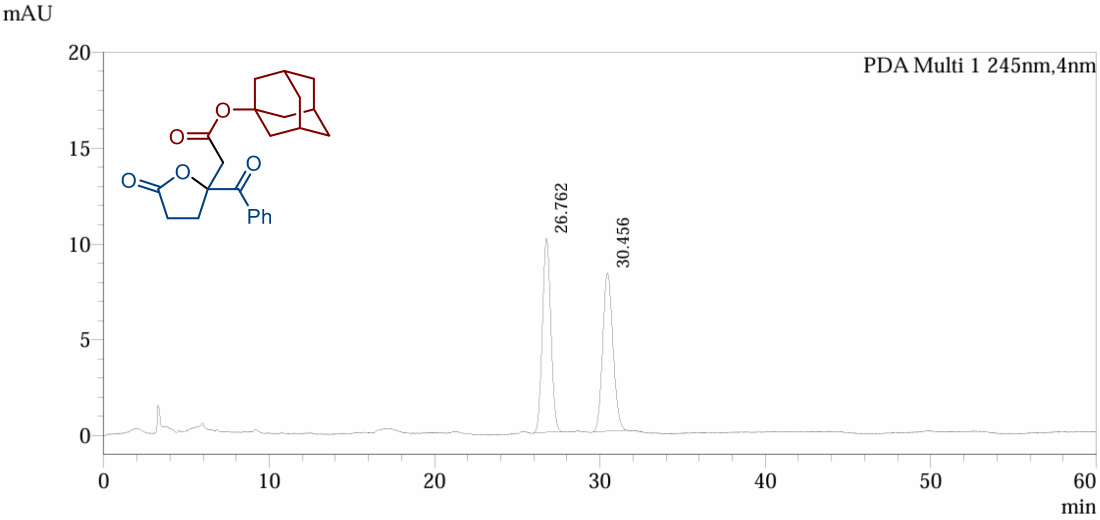

<Peak Table>

PDA Ch1 245nm

Peak#	Ret. Time	Area	Height	Aera%
1	26.762	359386	10114	50.194
2	30.456	356614	8255	49.806

<Chromatogram>

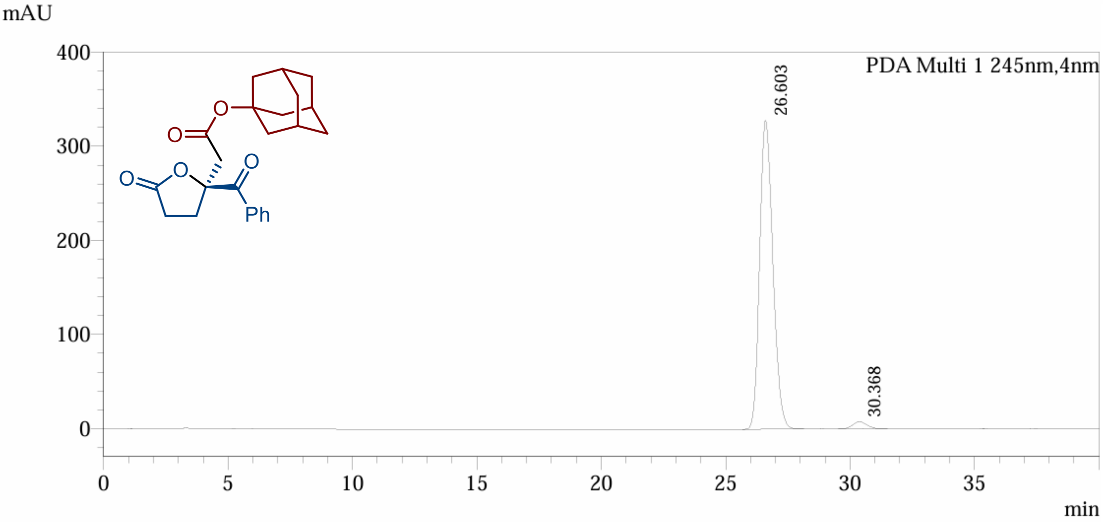

<Peak Table>

PDA Ch1 245nm

Peak#	Ret. Time	Area	Height	Aera%
1	26.603	12143054	328064	97.476
2	30.368	314386	7599	2.524

3an’

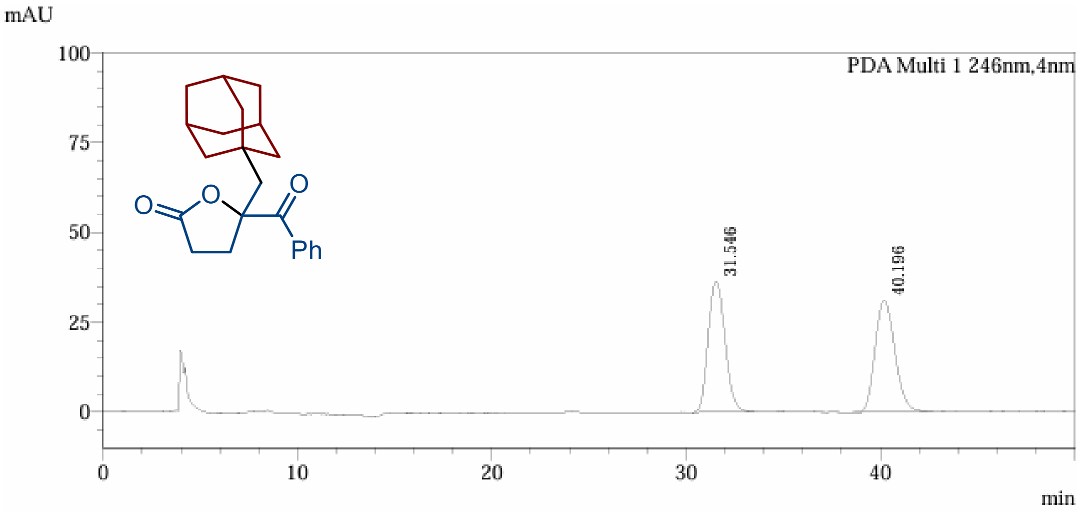

<Peak Table>

PDA Ch1 246nm

Peak#	Ret. Time	Area	Height	Aera%
1	31.546	2202553	36353	50.095
2	40.196	2194205	31084	49.905

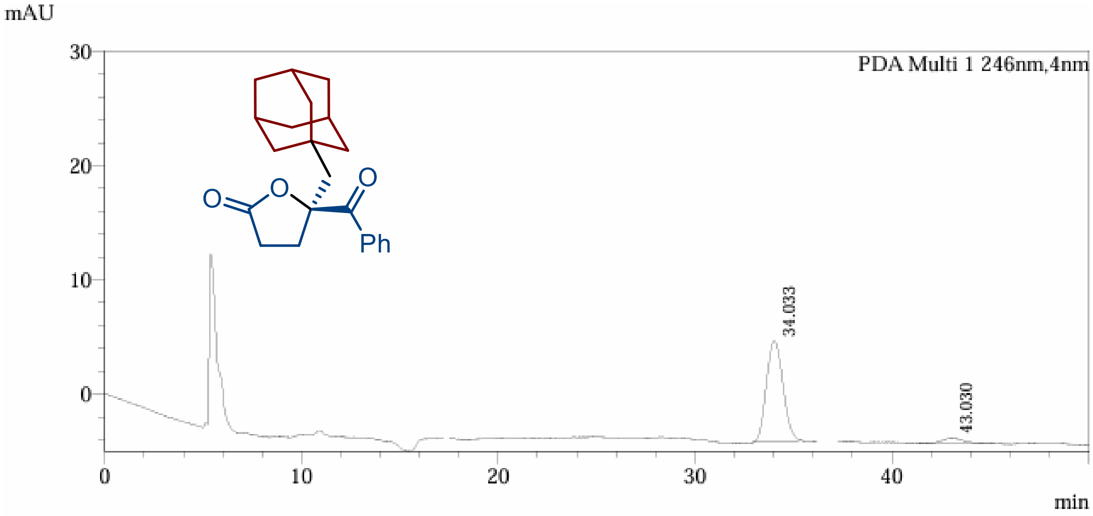

<Peak Table>

PDA Ch1 246nm

Peak#	Ret. Time	Area	Height	Aera%
1	34.033	519321	8794	94.472
2	43.030	30385	437	5.528

3ao

<Chromatogram>

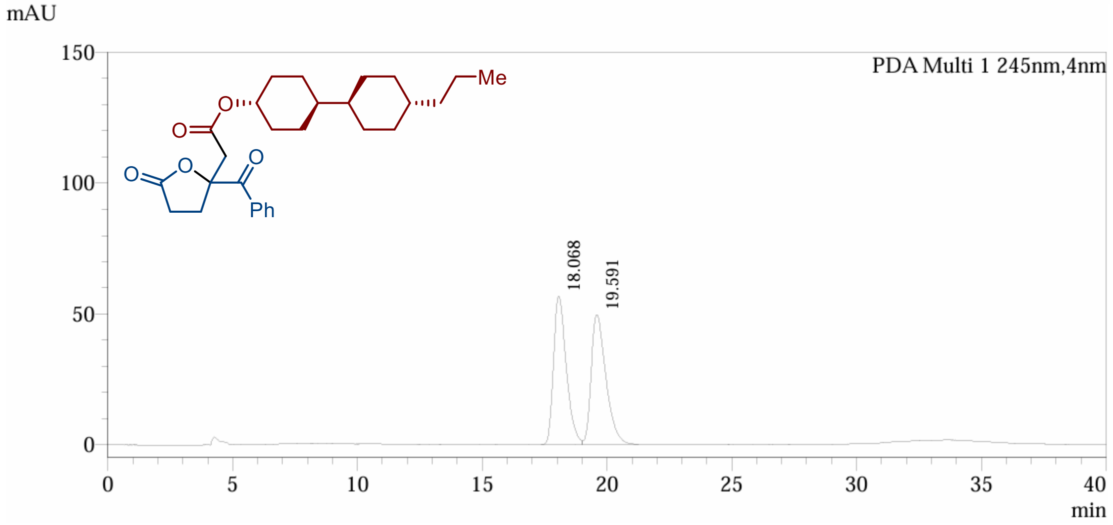

<Peak Table>

PDA Ch1 245nm

Peak#	Ret. Time	Area	Height	Aera%
1	18.068	2014527	56714	49.802
2	19.591	2030536	49622	50.198

<Chromatogram>

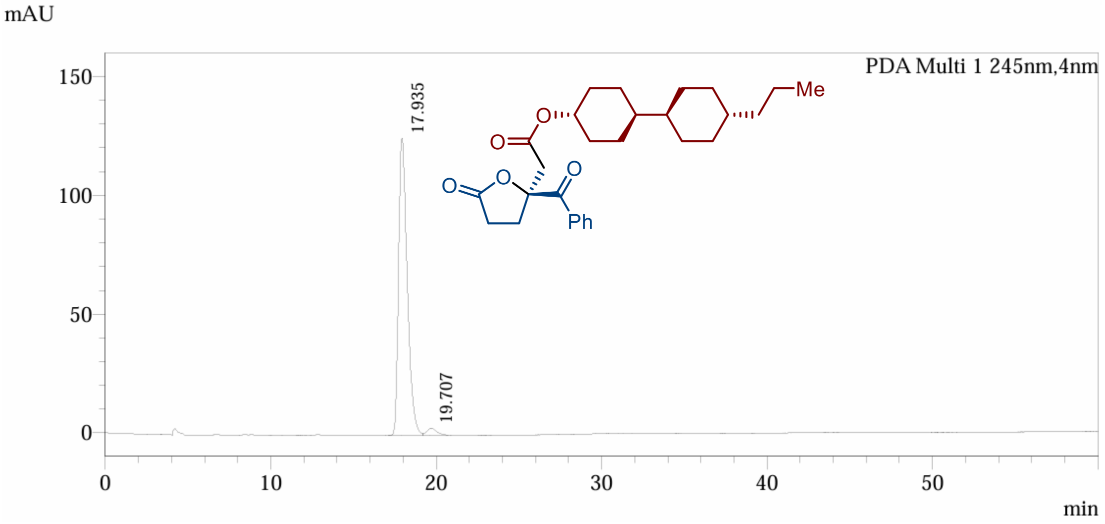

<Peak Table>

PDA Ch1 245nm

Peak#	Ret. Time	Area	Height	Aera%
1	17.935	4553206	125193	97.409
2	19.707	121093	2966	2.591

3ap

<Chromatogram>

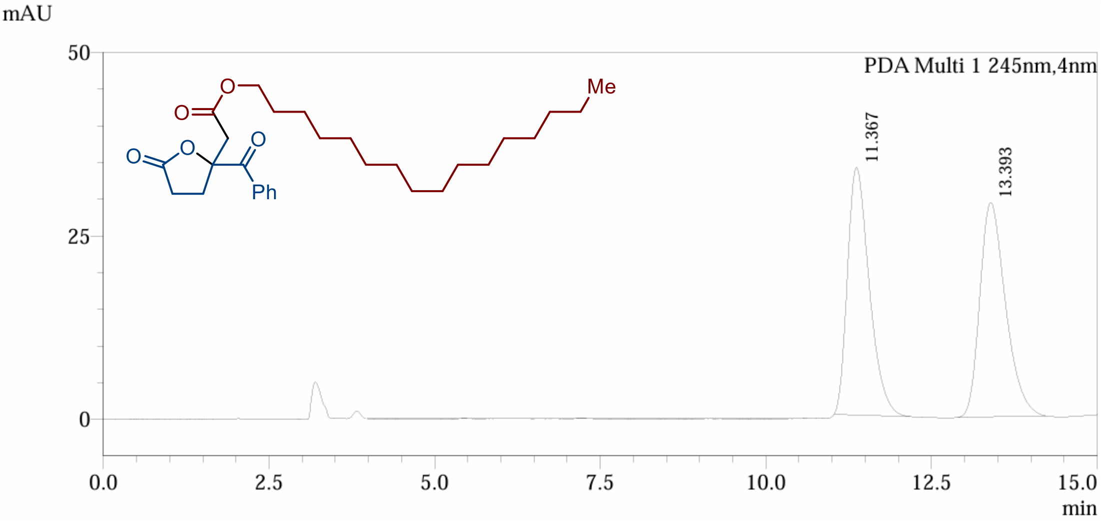

<Peak Table>

PDA Ch1 245nm

Peak#	Ret. Time	Area	Height	Aera%
1	11.367	774537	33720	49.533
2	13.393	789135	29186	50.467

<Chromatogram>

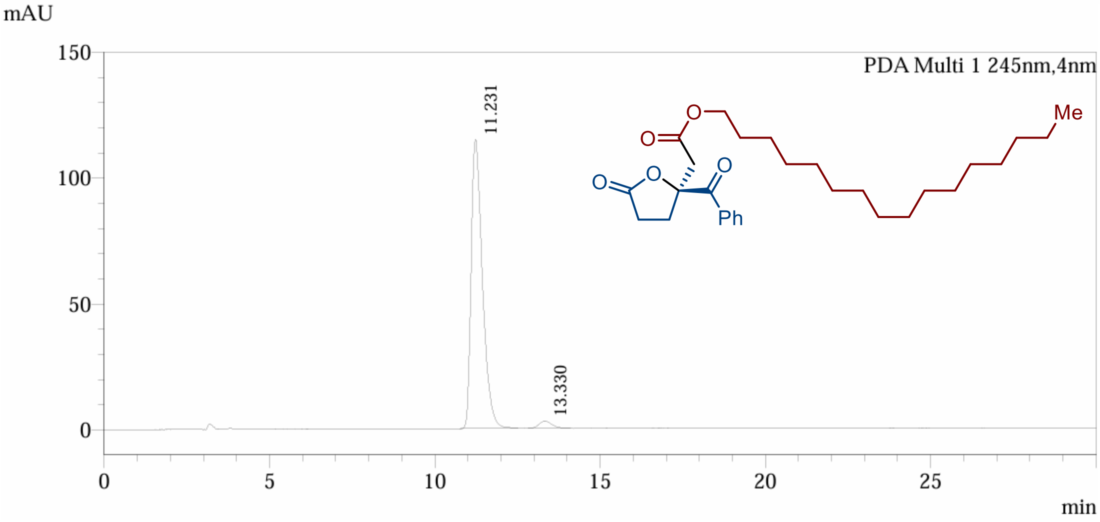

<Peak Table>

PDA Ch1 245nm

Peak#	Ret. Time	Area	Height	Aera%
1	11.231	2719954	114771	97.195
2	13.330	78505	2911	2.805

3aq

<Chromatogram>

mAU

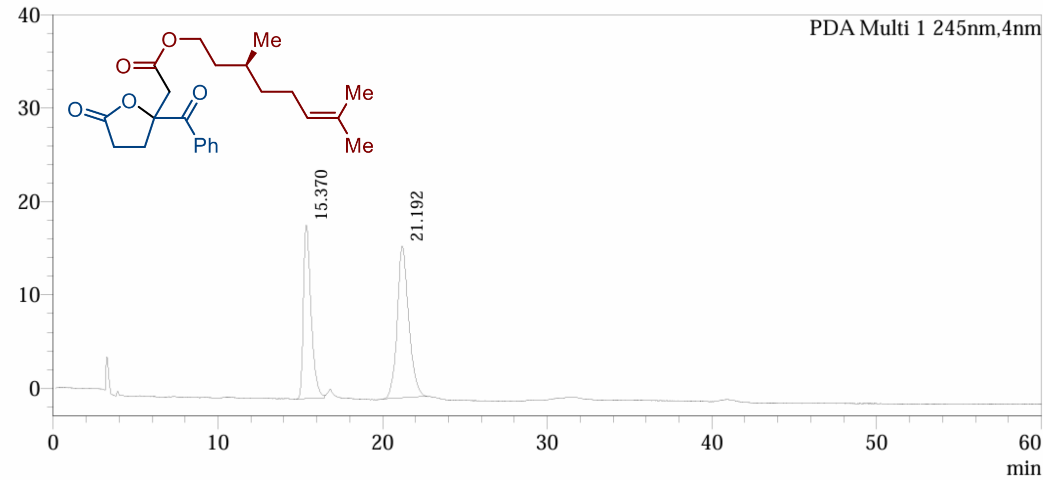

<Peak Table>

PDA Ch1 245nm

Peak#	Ret. Time	Area	Height	Aera%
1	15.370	597843	18581	43.026
2	21.192	791662	16240	56.974

<Chromatogram>

mAU

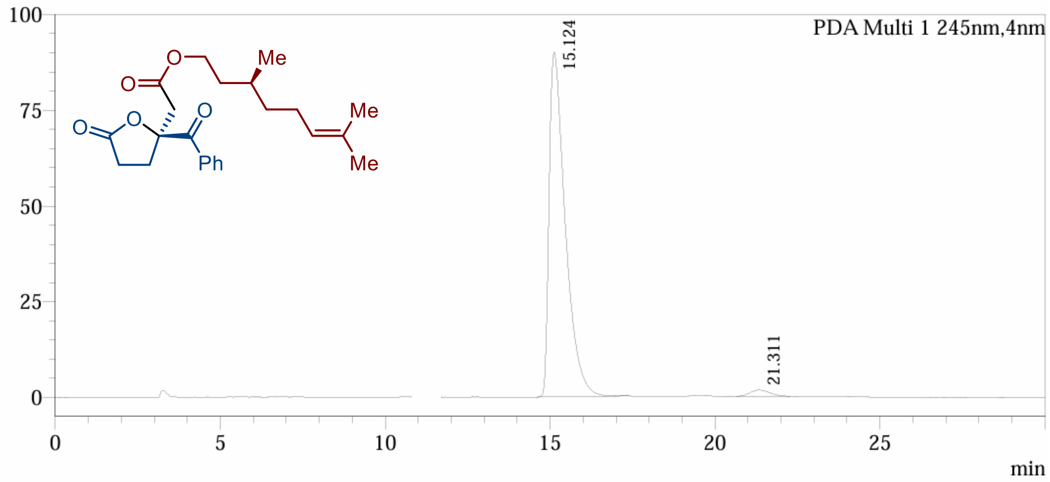

<Peak Table>

PDA Ch1 245nm

Peak#	Ret. Time	Area	Height	Aera%
1	15.124	3059504	90059	97.743
2	21.311	70632	1694	2.257

3ar

<Chromatogram>

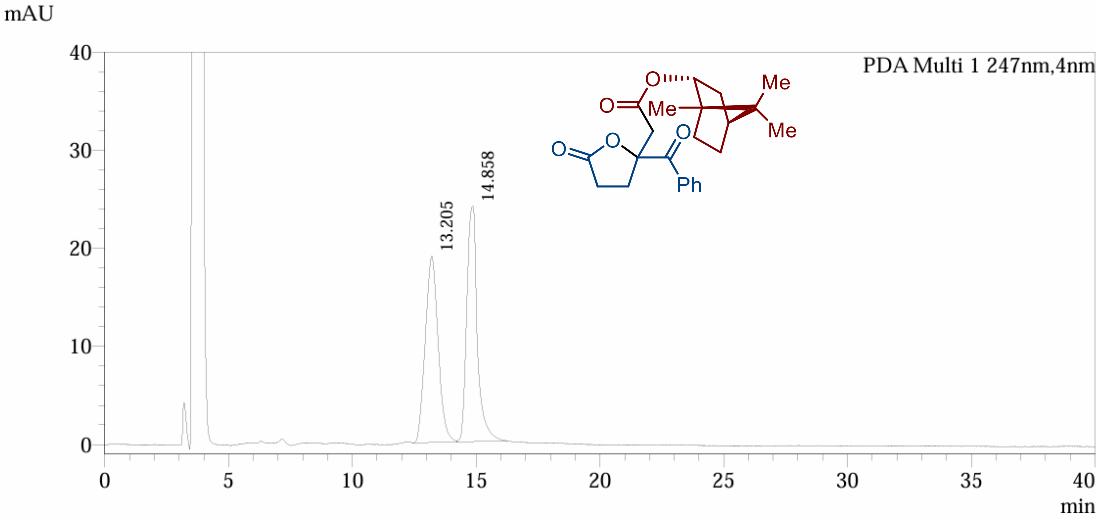

<Peak Table>

PDA Ch1 247nm

Peak#	Ret. Time	Area	Height	Aera%
1	13.205	688003	19002	49.200
2	14.858	710380	24014	50.800

<Chromatogram>

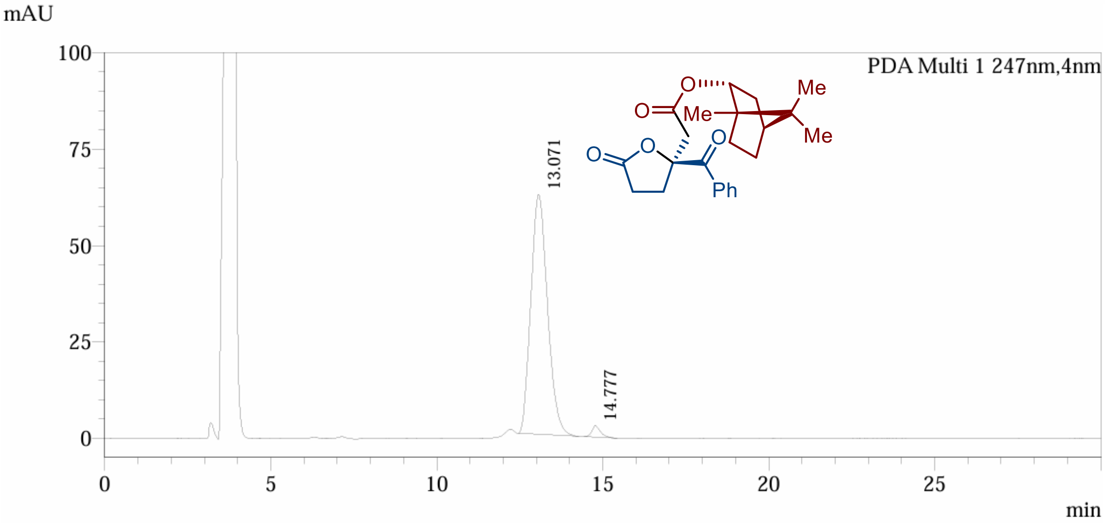

<Peak Table>

PDA Ch1 247nm

Peak#	Ret. Time	Area	Height	Aera%
1	13.071	2173408	62210	97.582
2	14.777	53845	3014	2.418

3as

<Chromatogram>

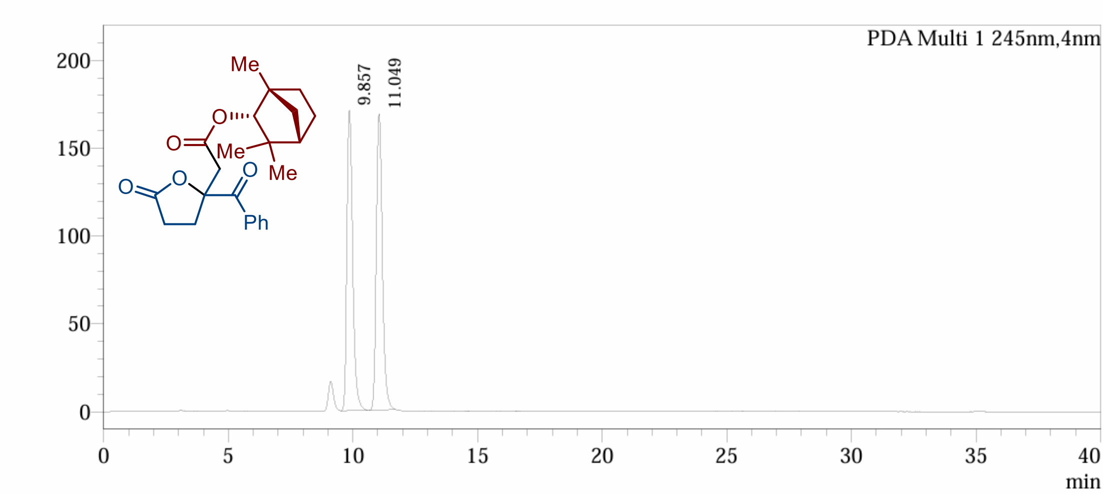

<Peak Table>

PDA Ch1 245nm

Peak#	Ret. Time	Area	Height	Aera%
1	9.857	2754185	170932	49.209
2	11.049	2842701	168563	50.791

<Chromatogram>

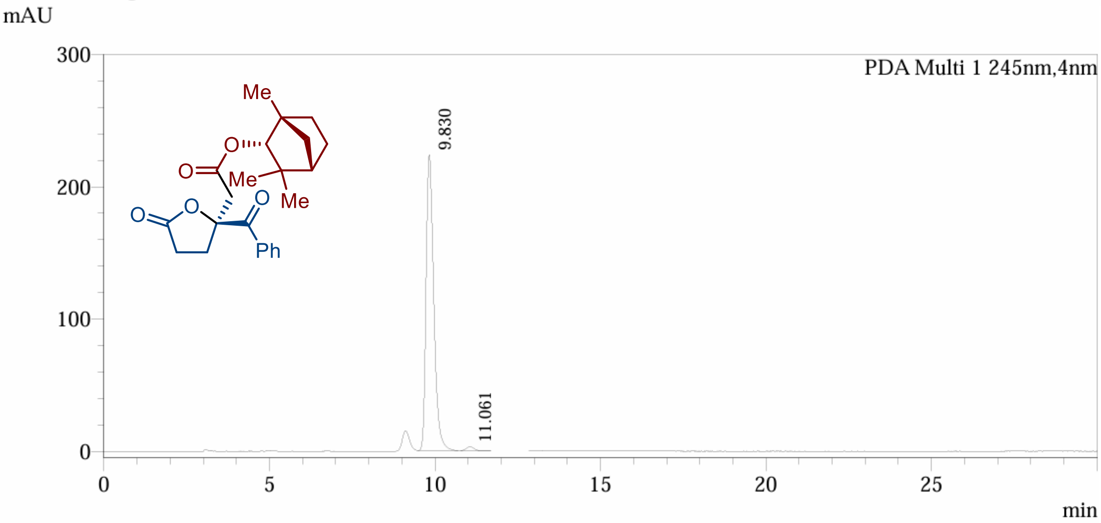

<Peak Table>

PDA Ch1 245nm

Peak#	Ret. Time	Area	Height	Aera%
1	9.830	3599855	223446	98.721
2	11.061	46624	2874	1.279

3at

<Chromatogram>

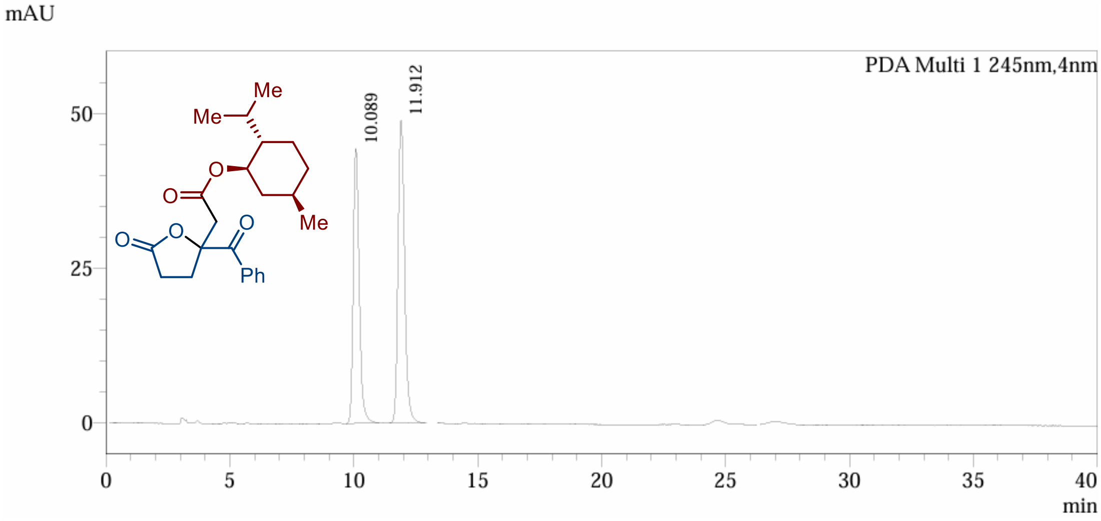

<Peak Table>

PDA Ch1 245nm

Peak#	Ret. Time	Area	Height	Aera%
1	10.089	739468	44379	45.255
2	11.912	894527	48875	54.745

<Chromatogram>

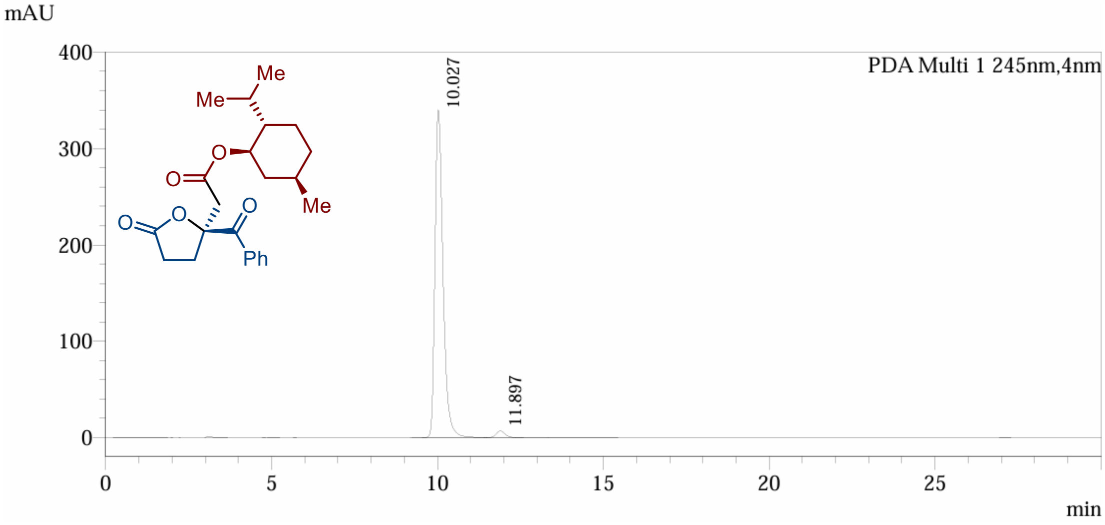

<Peak Table>

PDA Ch1 245nm

Peak#	Ret. Time	Area	Height	Aera%
1	10.027	5722475	340080	97.853
2	11.897	125570	6743	2.147

3au

<Chromatogram>

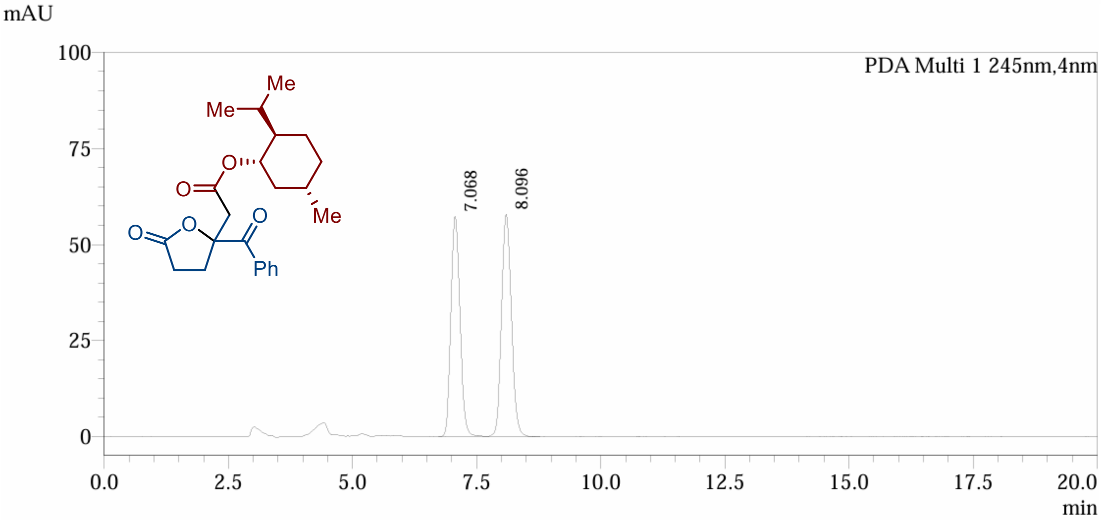

<Peak Table>

PDA Ch1 245nm

Peak#	Ret. Time	Area	Height	Aera%
1	7.068	728252	57190	47.737
2	8.096	797307	57795	52.263

<Chromatogram>

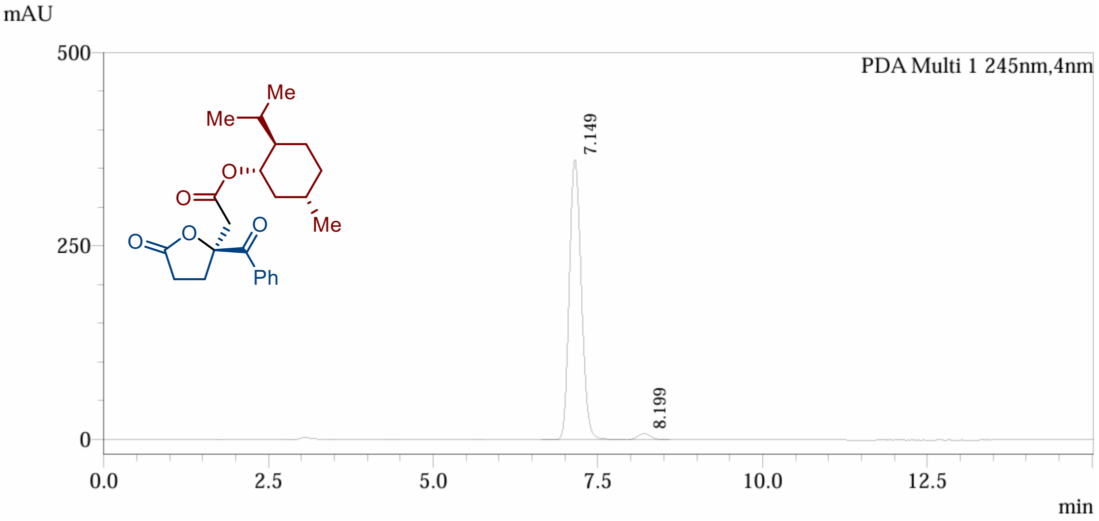

<Peak Table>

PDA Ch1 245nm

Peak#	Ret. Time	Area	Height	Aera%
1	7.149	4589890	362138	97.819
2	8.199	102325	7699	2.181

3av

<Chromatogram>

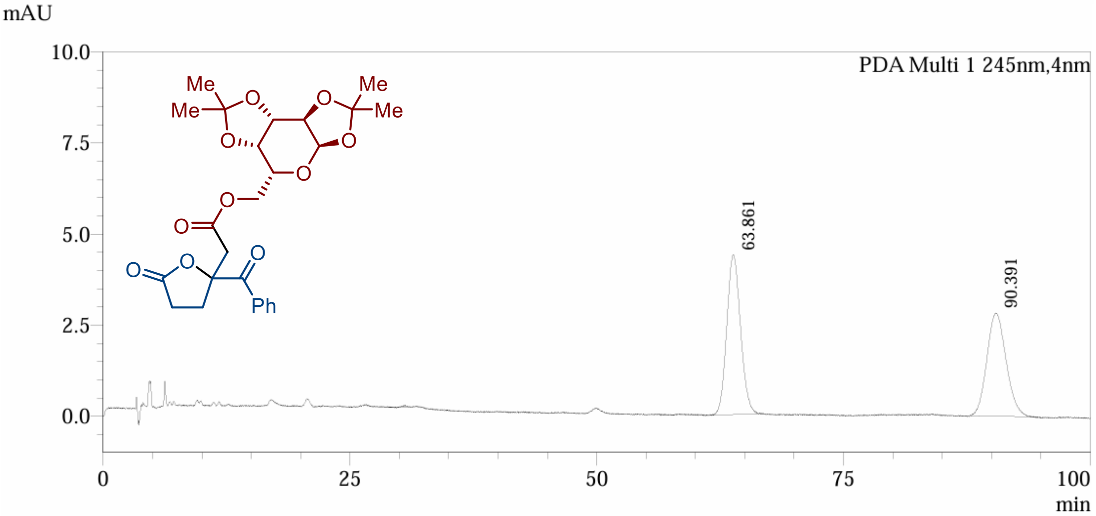

<Peak Table>

PDA Ch1 245nm

Peak#	Ret. Time	Area	Height	Aera%
1	63.861	424869	4388	52.506
2	90.391	384312	2833	47.494

<Chromatogram>

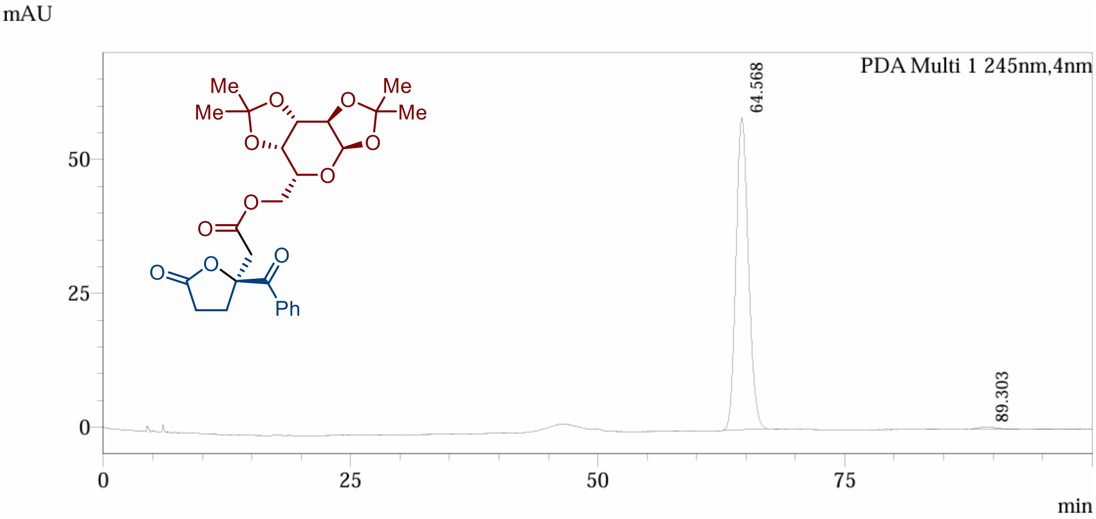

<Peak Table>

PDA Ch1 245nm

Peak#	Ret. Time	Area	Height	Aera%
1	64.568	5103538	58274	99.169
2	89.303	42790	406	0.831

3aw

<Chromatogram>

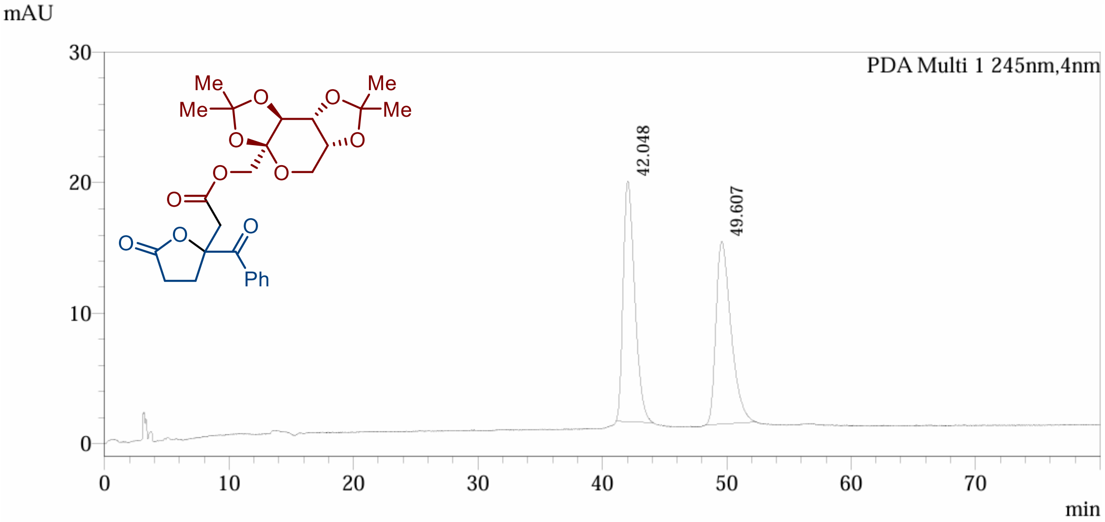

<Peak Table>

PDA Ch1 245nm

Peak#	Ret. Time	Area	Height	Aera%
1	42.048	1185142	18413	50.128
2	49.607	1179099	13987	49.872

<Chromatogram>

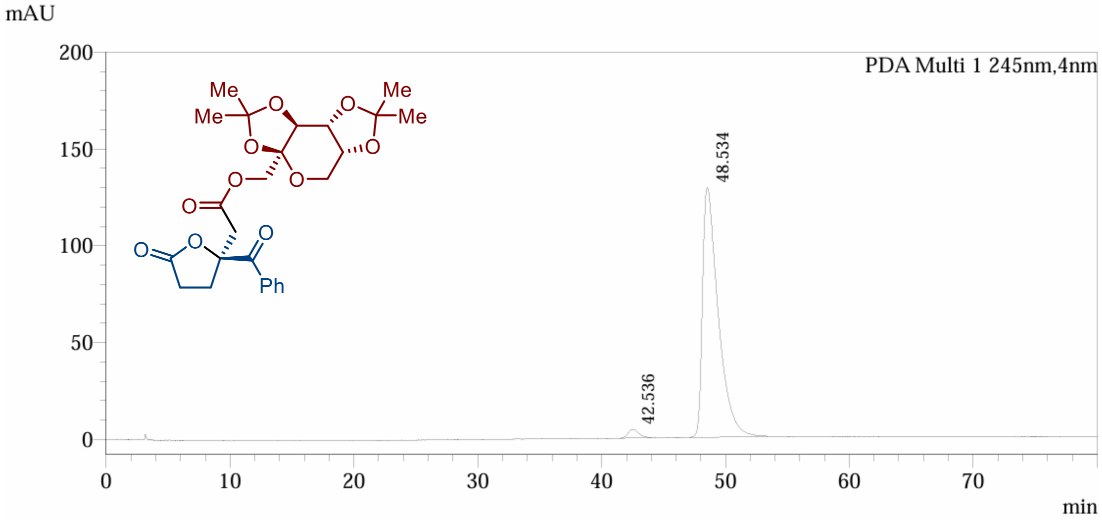

<Peak Table>

PDA Ch1 245nm

Peak#	Ret. Time	Area	Height	Aera%
1	42.536	274959	4489	2.365
2	48.534	11352276	129083	97.635

3ax

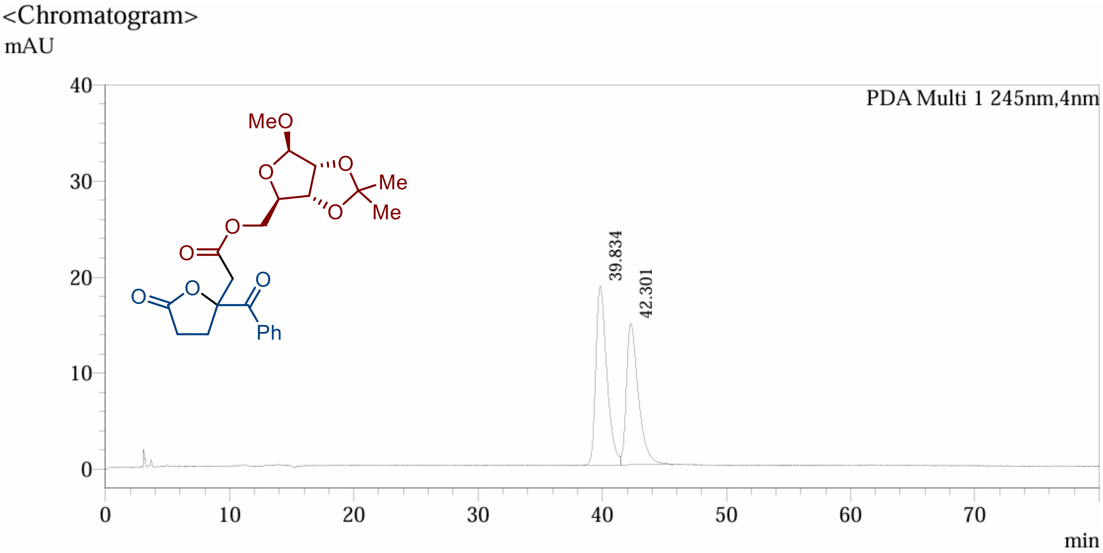

<Peak Table>

PDA Ch1 245nm

Peak#	Ret. Time	Area	Height	Aera%
1	39.834	1144742	18616	53.618
2	42.301	990266	14718	46.382

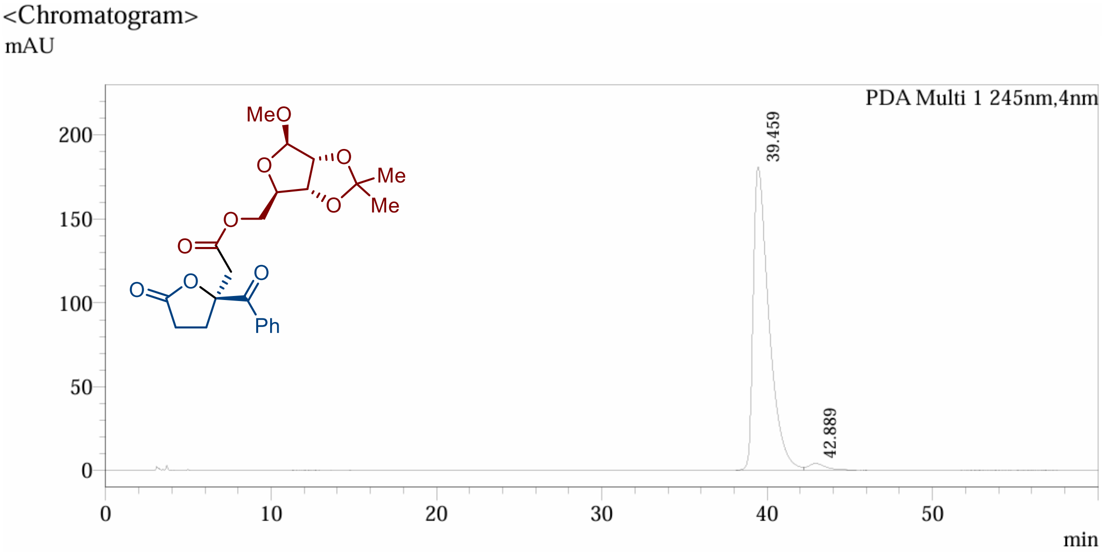

<Peak Table>

PDA Ch1 245nm

Peak#	Ret. Time	Area	Height	Aera%
1	39.459	11936537	180648	97.634
2	42.889	289290	3982	2.366

3ay

<Chromatogram>

mAU

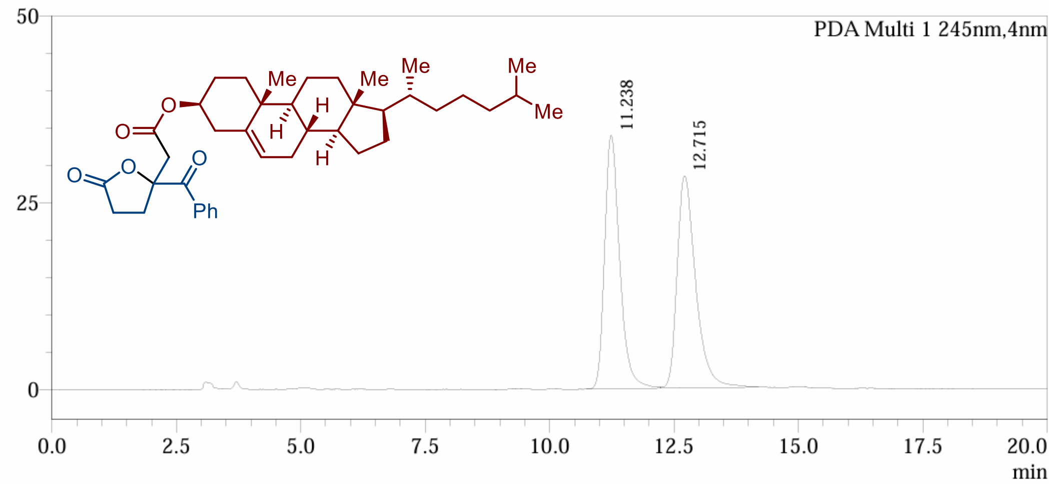

<Peak Table>

PDA Ch1 245nm

Peak#	Ret. Time	Area	Height	Aera%
1	11.238	716412	33891	49.357
2	12.715	735086	28390	50.643

<Chromatogram>

mAU

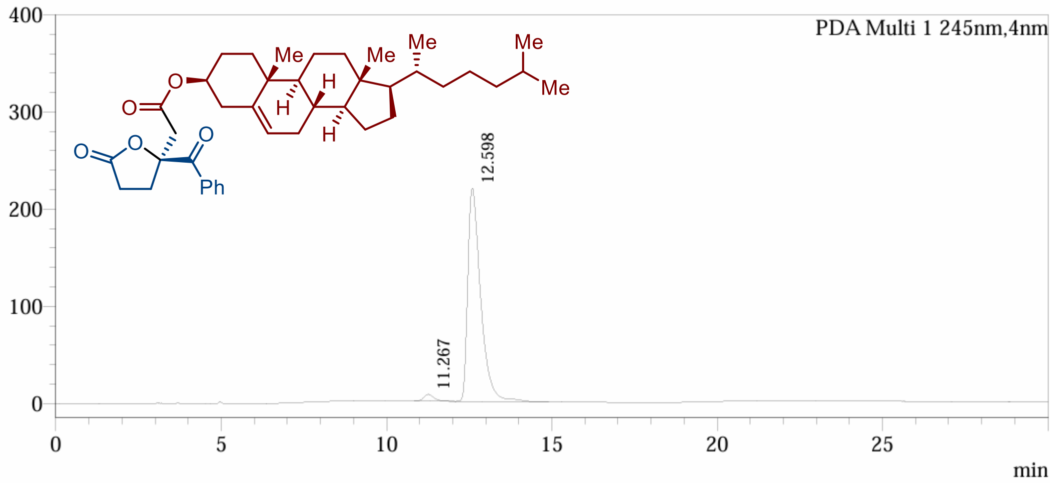

<Peak Table>

PDA Ch1 245nm

Peak#	Ret. Time	Area	Height	Aera%
1	11.267	147291	6895	2.413
2	12.598	5957816	219391	97.587

3az

<Chromatogram>

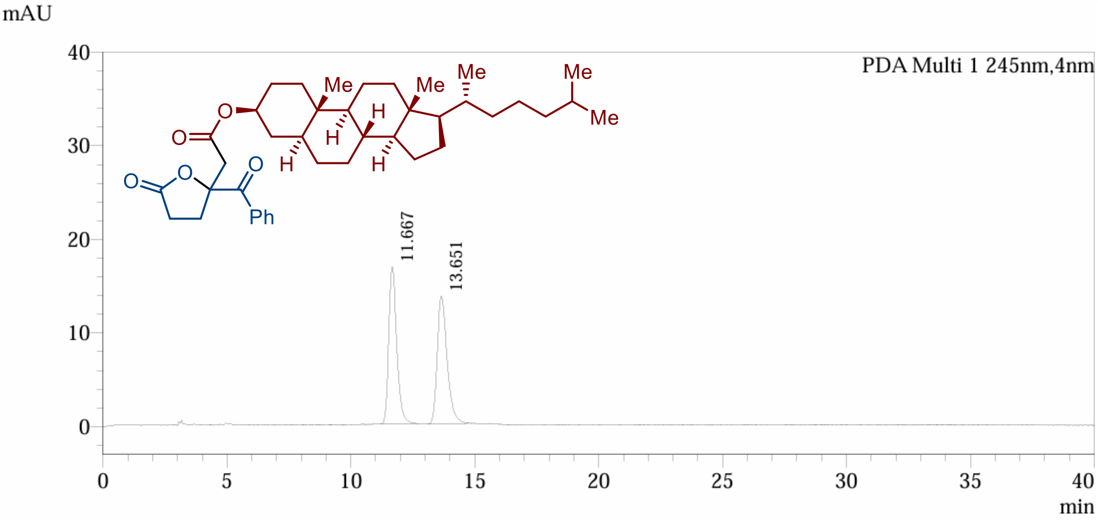

<Peak Table>

PDA Ch1 245nm

Peak#	Ret. Time	Area	Height	Aera%
1	11.667	377448	16782	49.895
2	13.651	379034	13632	50.105

<Chromatogram>

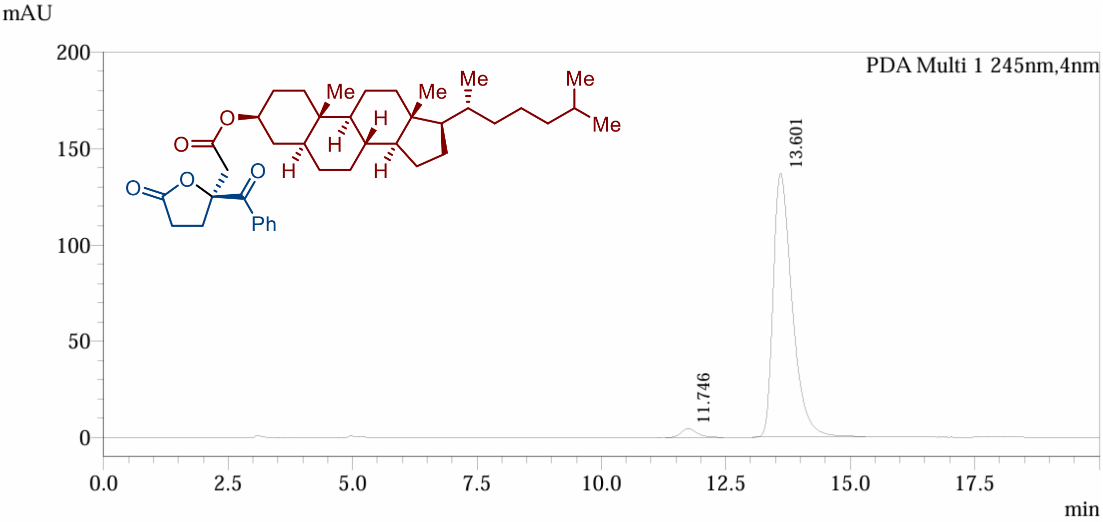

<Peak Table>

PDA Ch1 245nm

Peak#	Ret. Time	Area	Height	Aera%
1	11.746	97931	4488	2.623
2	13.601	3636002	136930	97.377

3ba

<Chromatogram>

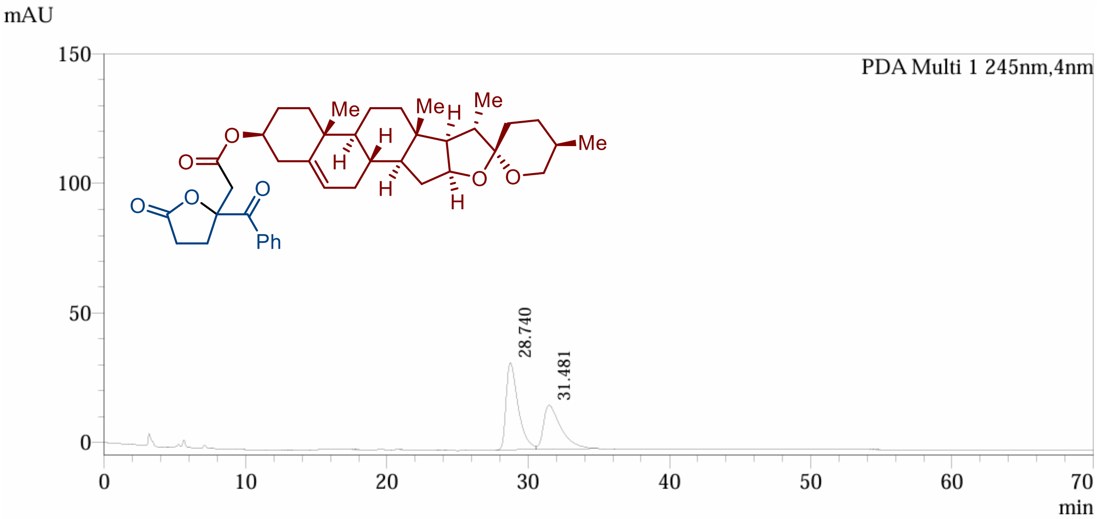

<Peak Table>

PDA Ch1 245nm

Peak#	Ret. Time	Area	Height	Aera%
1	28.740	2004862	33528	57.728
2	31.481	1468107	16993	42.272

<Chromatogram>

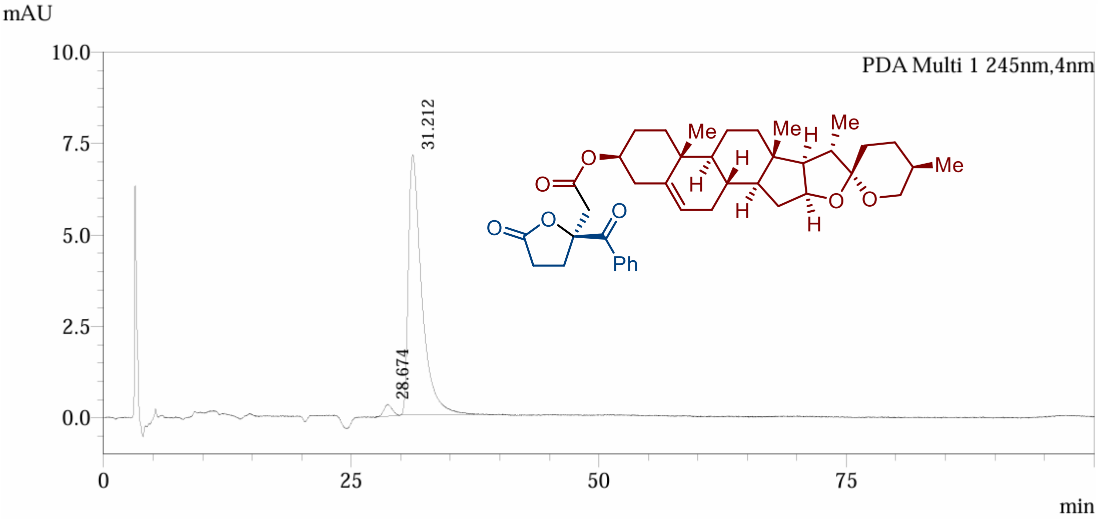

<Peak Table>

PDA Ch1 245nm

Peak#	Ret. Time	Area	Height	Aera%
1	28.674	19098	325	2.789
2	31.212	665617	7106	97.211

3bb

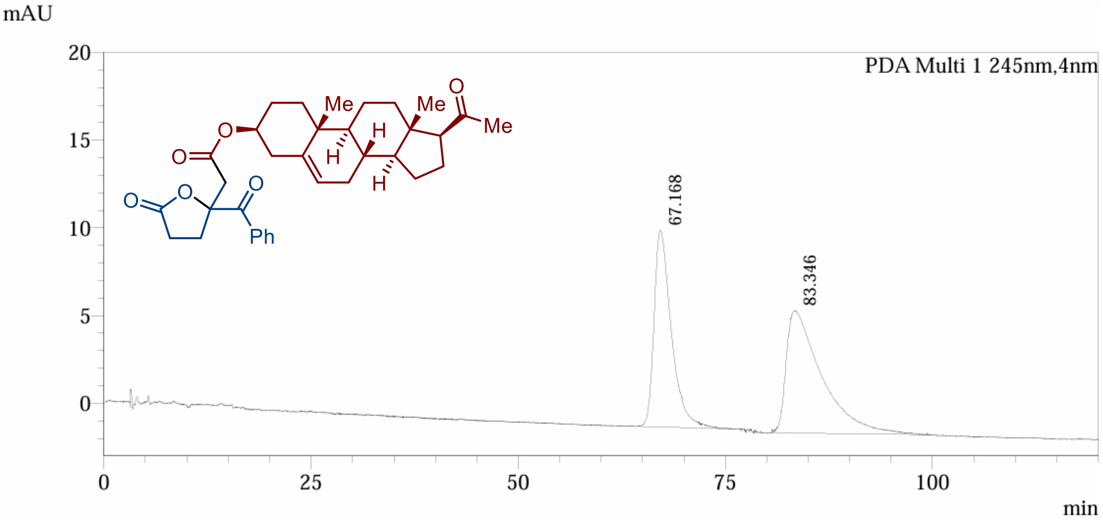

<Peak Table>

PDA Ch1 245nm

Peak#	Ret. Time	Area	Height	Aera%
1	67.168	1628505	11216	43.867
2	83.346	2083850	6984	56.133

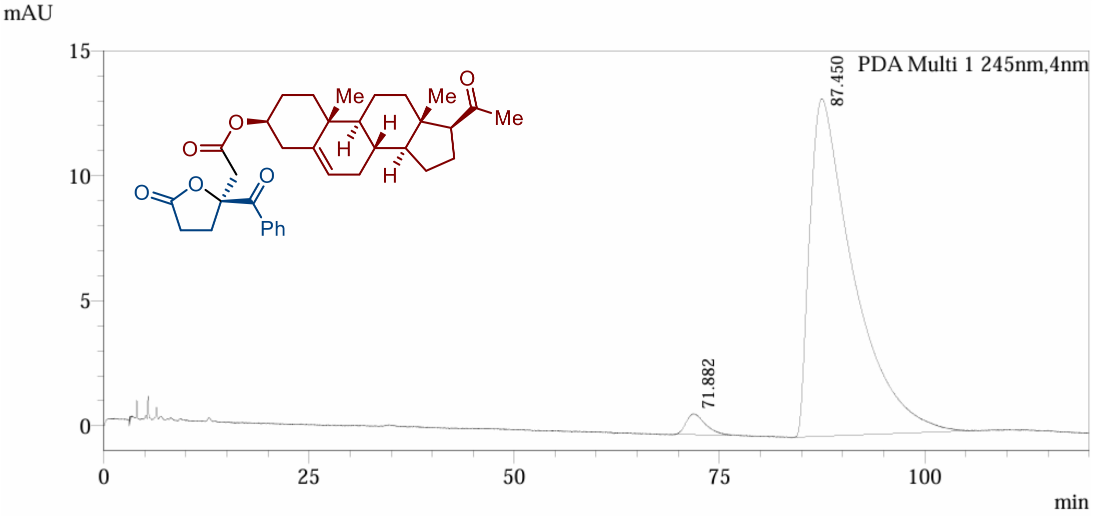

<Peak Table>

PDA Ch1 245nm

Peak#	Ret. Time	Area	Height	Aera%
1	71.882	133826	838	2.599
2	87.450	5014522	13495	97.401

3bc

<Chromatogram>

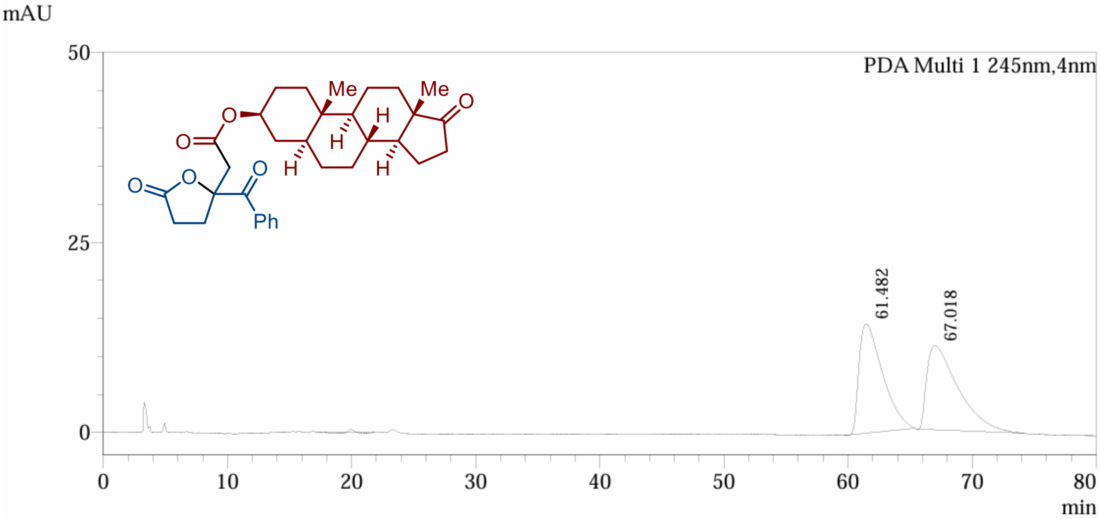

<Peak Table>

PDA Ch1 245nm

Peak#	Ret. Time	Area	Height	Aera%
1	61.482	1899161	14353	49.308
2	67.018	1952481	11056	50.692

<Chromatogram>

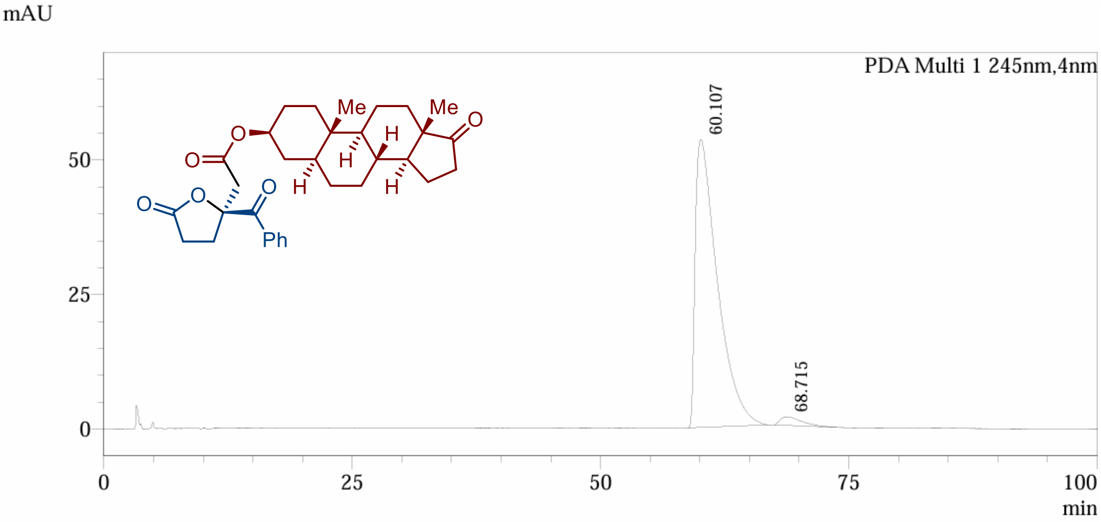

<Peak Table>

PDA Ch1 245nm

Peak#	Ret. Time	Area	Height	Aera%
1	60.107	8327266	53494	97.239
2	68.715	236424	1561	2.761

3at + 3au

<Chromatogram>

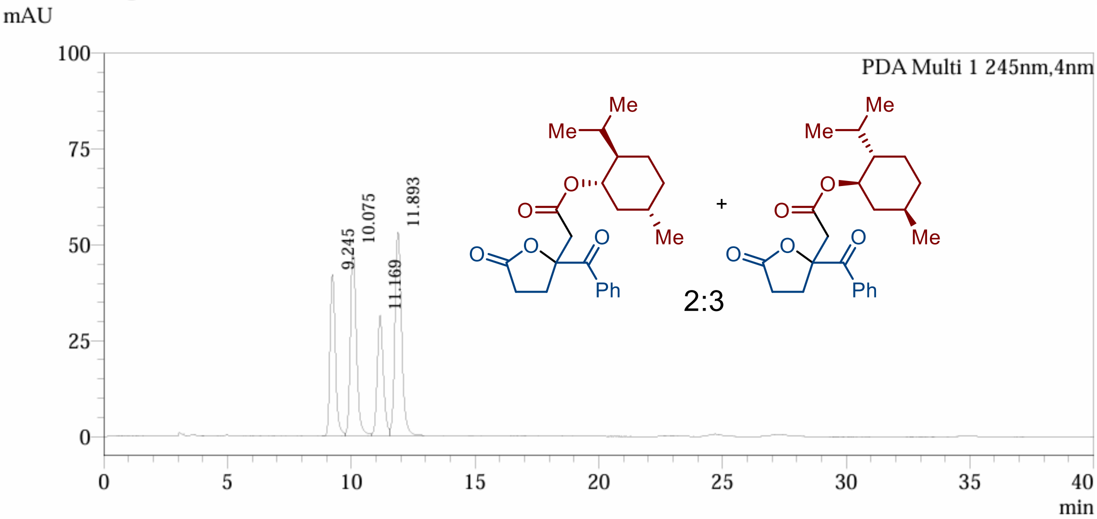

<Peak Table>

PDA Ch1 245nm

Peak#	Ret. Time	Area	Height	Aera%
1	9.245	642916	42243	21.360
2	10.075	834445	48939	27.723
3	11.169	541508	31507	17.991
4	11.893	991096	53105	32.927

<Chromatogram>

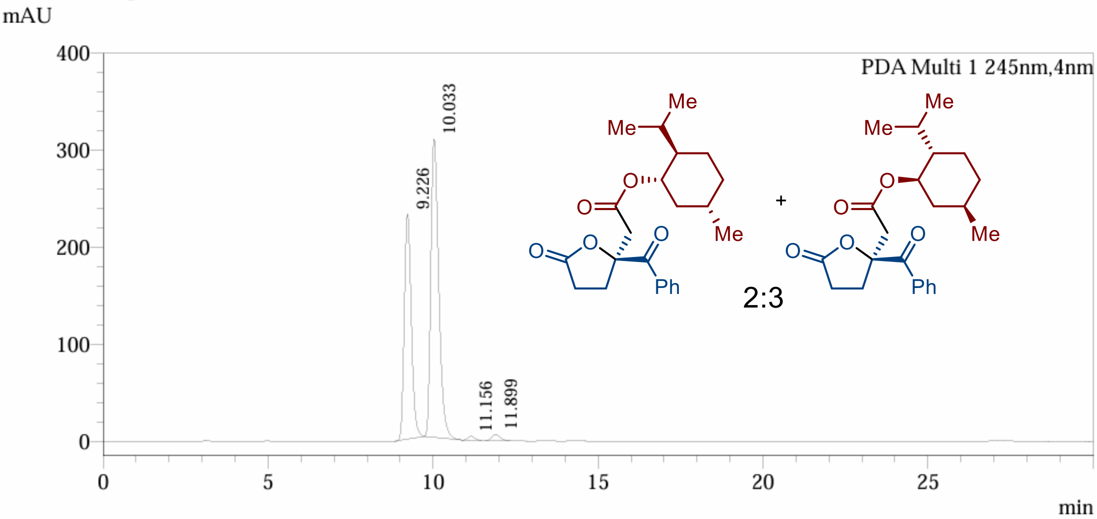

<Peak Table>

PDA Ch1 245nm

Peak#	Ret. Time	Area	Height	Aera%
1	9.226	3394634	232483	39.184
2	10.033	5090877	307925	58.764
3	11.156	61504	4053	0.710
4	11.899	116260	6598	1.342

3a-I

<Chromatogram>

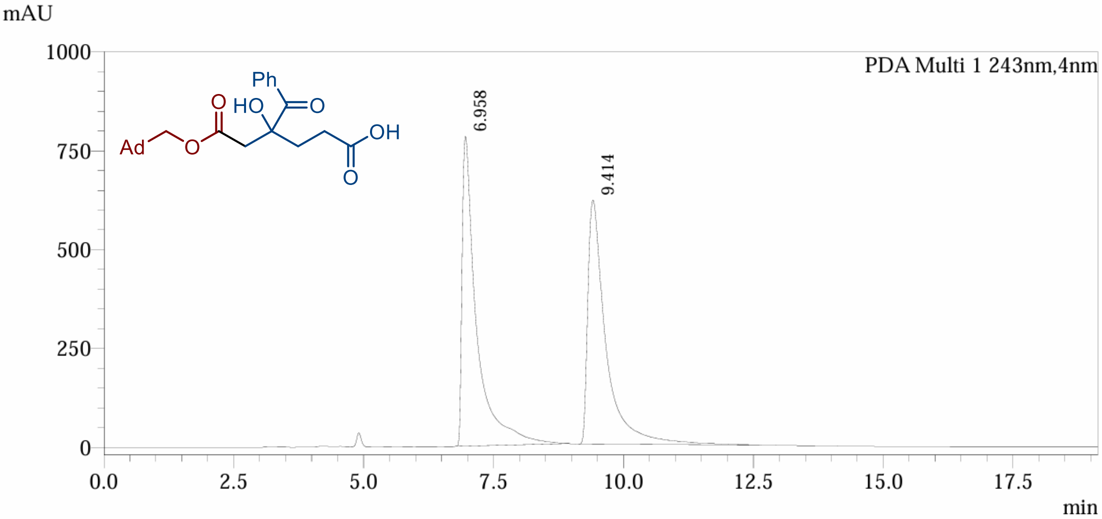

<Peak Table>

PDA Ch1 243nm

Peak#	Ret. Time	Area	Height	Aera%
1	6.958	15307836	783149	50.416
2	9.414	15055075	617333	49.584

<Chromatogram>

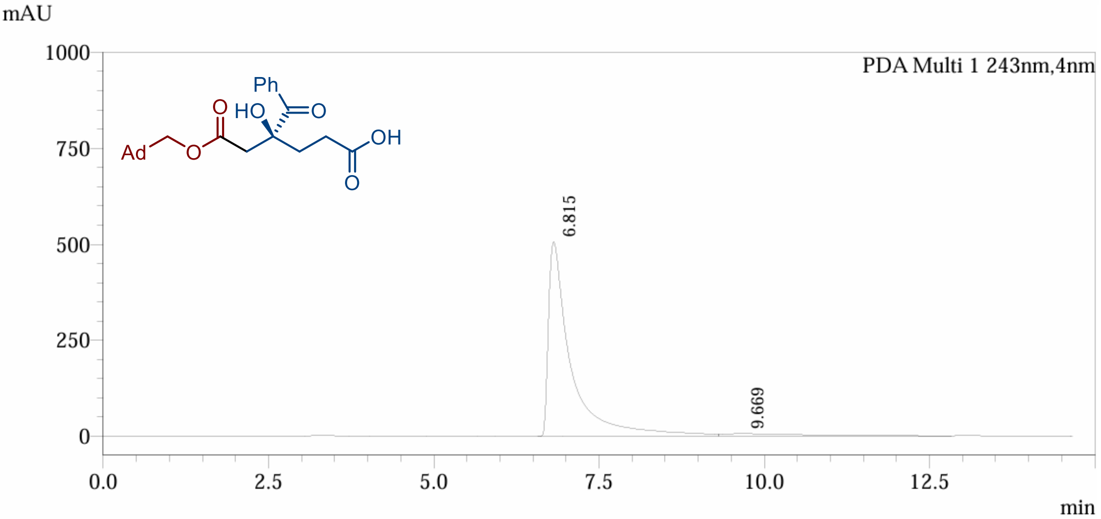

<Peak Table>

PDA Ch1 243nm

Peak#	Ret. Time	Area	Height	Aera%
1	6.815	11960933	506158	96.228
2	9.669	468851	5904	3.772

3a-II

<Chromatogram>

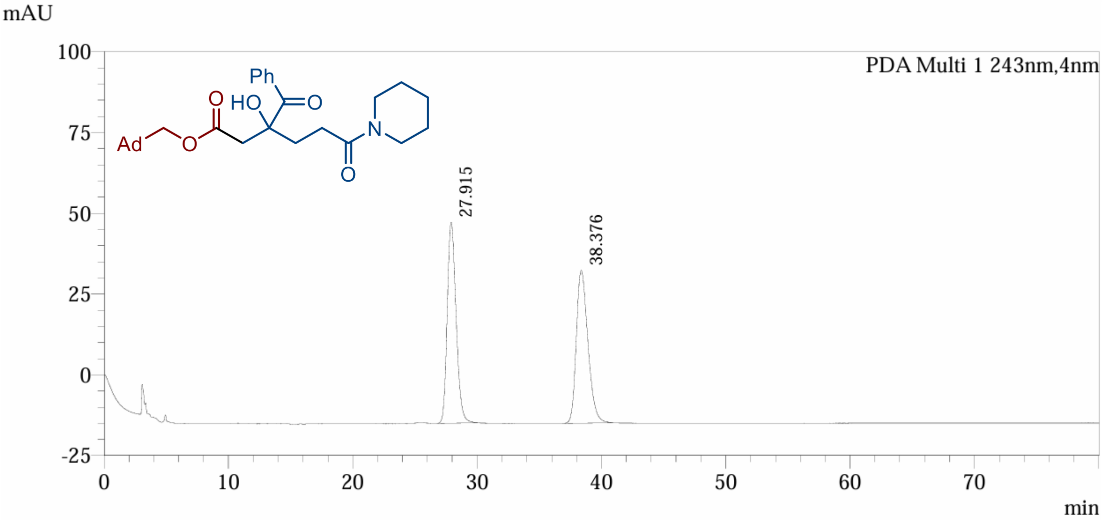

<Peak Table>

PDA Ch1 243nm

Peak#	Ret. Time	Area	Height	Aera%
1	27.915	3075106	62213	50.026
2	38.376	3071914	47341	49.974

<Chromatogram>

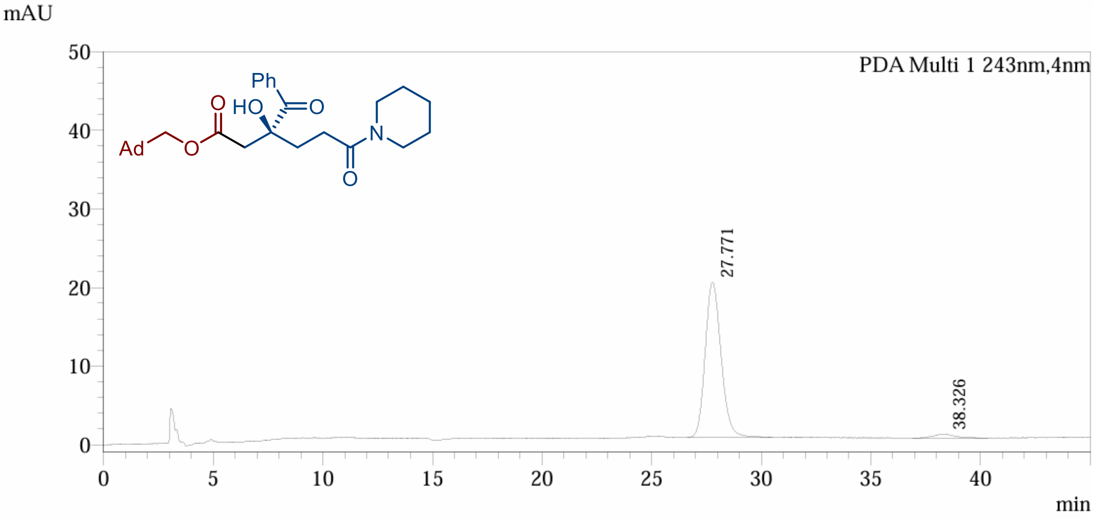

<Peak Table>

PDA Ch1 243nm

Peak#	Ret. Time	Area	Height	Aera%
1	27.771	997139	19754	97.308
2	38.326	27581	435	2.692

3a-III

<Chromatogram>

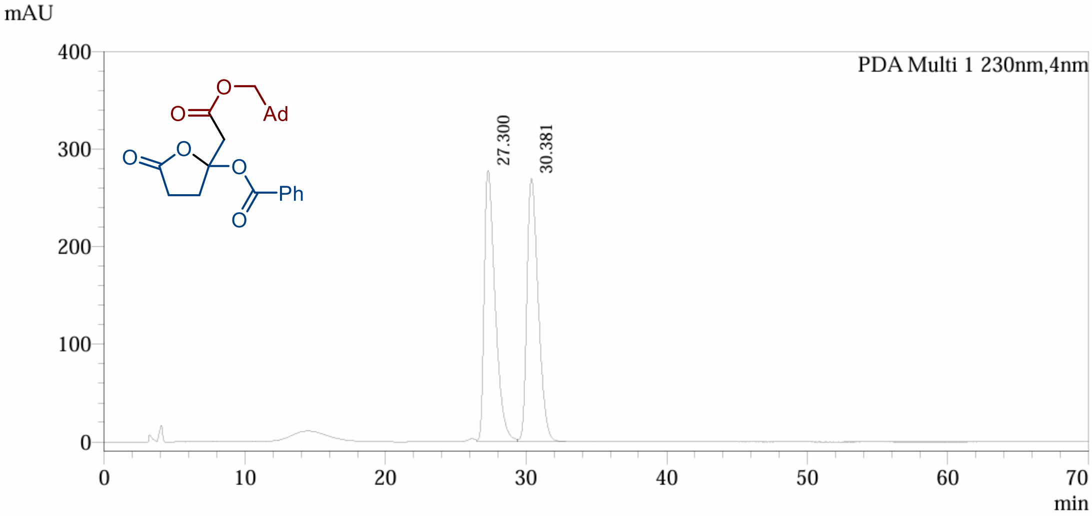

<Peak Table>

PDA Ch1 230nm

Peak#	Ret. Time	Area	Height	Aera%
1	27.300	15036236	278042	49.959
2	30.381	15060712	269670	50.041

<Chromatogram>

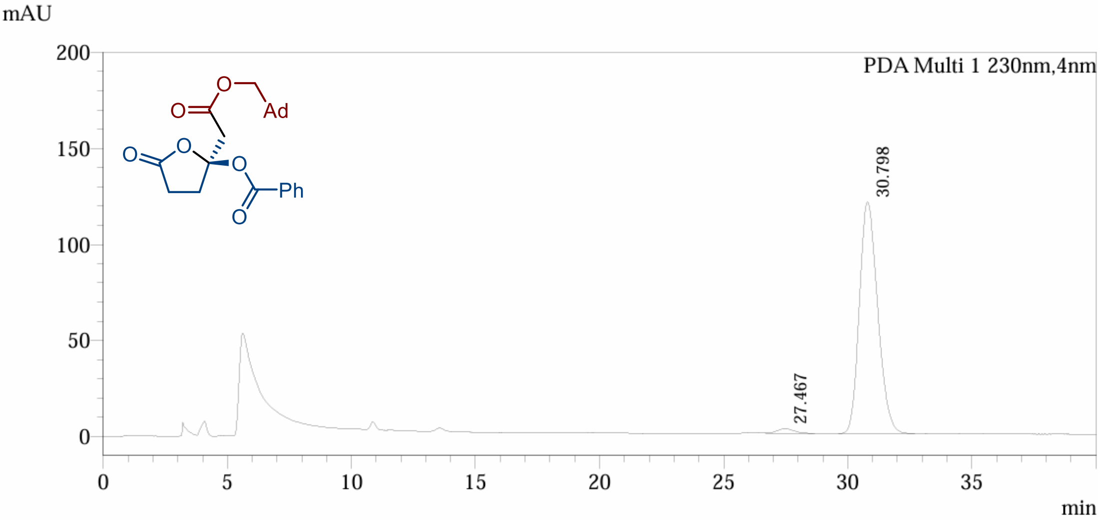

<Peak Table>

PDA Ch1 230nm

Peak#	Ret. Time	Area	Height	Aera%
1	27.467	117584	2400	1.862
2	30.798	6198742	120487	98.138

3a-IV

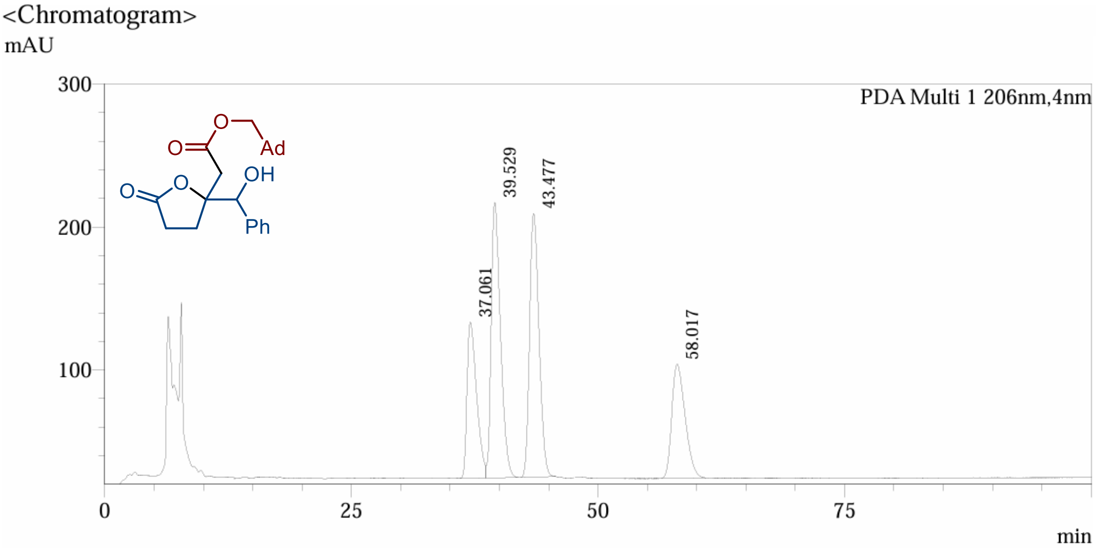

<Peak Table>

PDA Ch1 206nm

Peak#	Ret. Time	Area	Height	Aera%
1	37.061	7353642	108987	18.521
2	39.529	12611072	192425	31.762
3	43.477	12292930	184114	30.961
4	58.017	7446701	79424	18.755

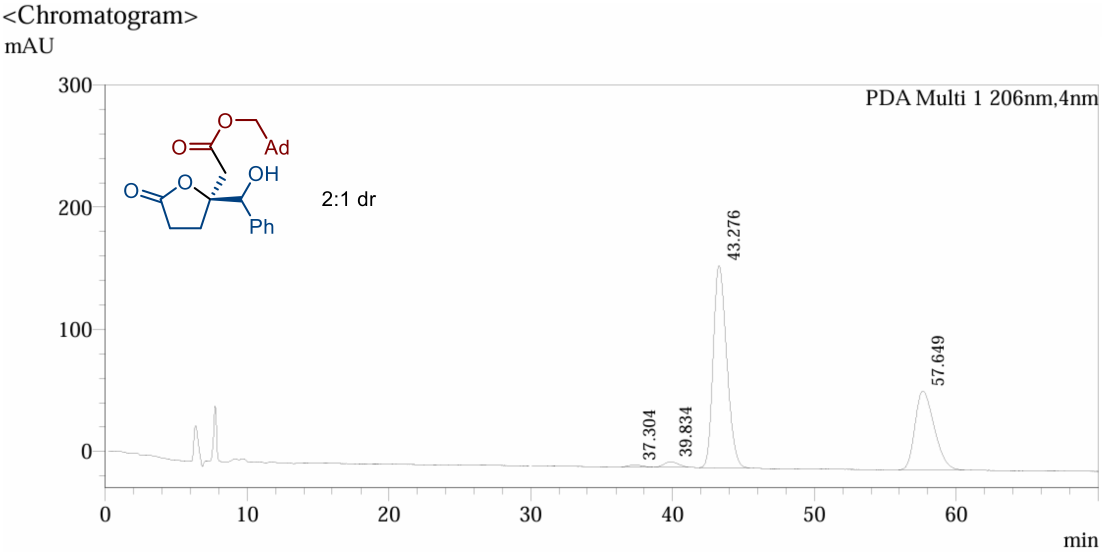

<Peak Table>

PDA Ch1 206nm

Peak#	Ret. Time	Area	Height	Aera%
1	37.304	91392	1478	0.526
2	39.834	282825	4268	1.626
3	43.276	10952221	165313	62.979
4	57.649	6063747	64422	34.869

3a-V

<Chromatogram>

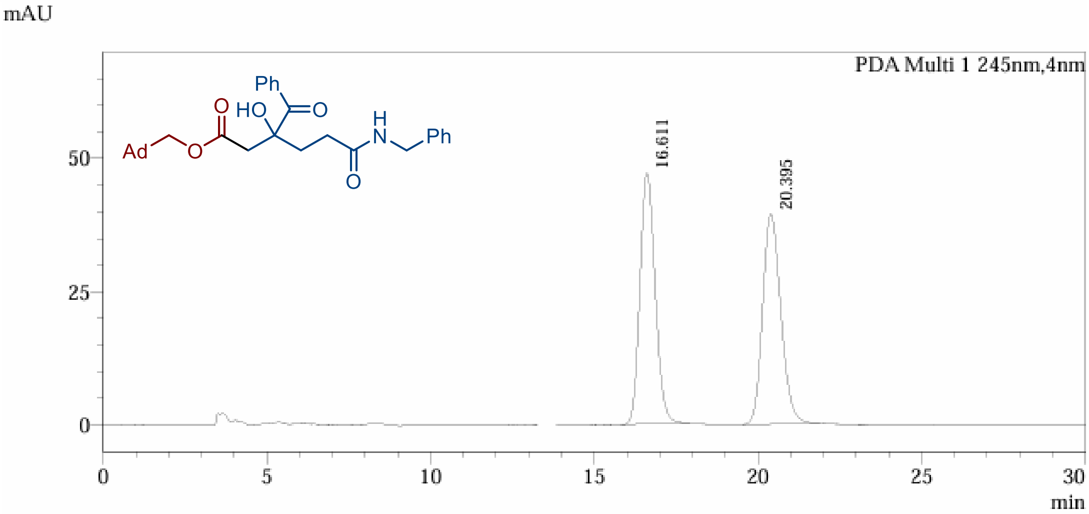

<Peak Table>

PDA Ch1 245nm

Peak#	Ret. Time	Area	Height	Aera%
1	16.611	1529353	47109	50.071
2	20.395	1525046	39406	49.929

<Chromatogram>

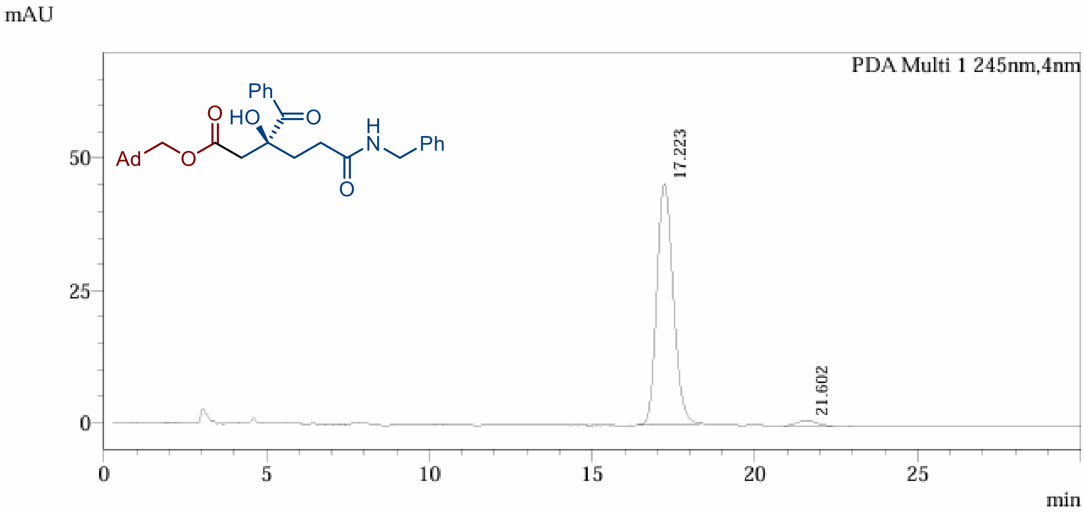

<Peak Table>

PDA Ch1 245nm

Peak#	Ret. Time	Area	Height	Aera%
1	17.223	1599973	45546	97.519
2	21.602	40700	1008	2.481

10. NMR spectra of compounds

((1s,3R)-adamantan-1-yl)methyl 2-((S)-2-benzoyl-5-oxotetrahydrofuran-2-yl)acetate (3a)

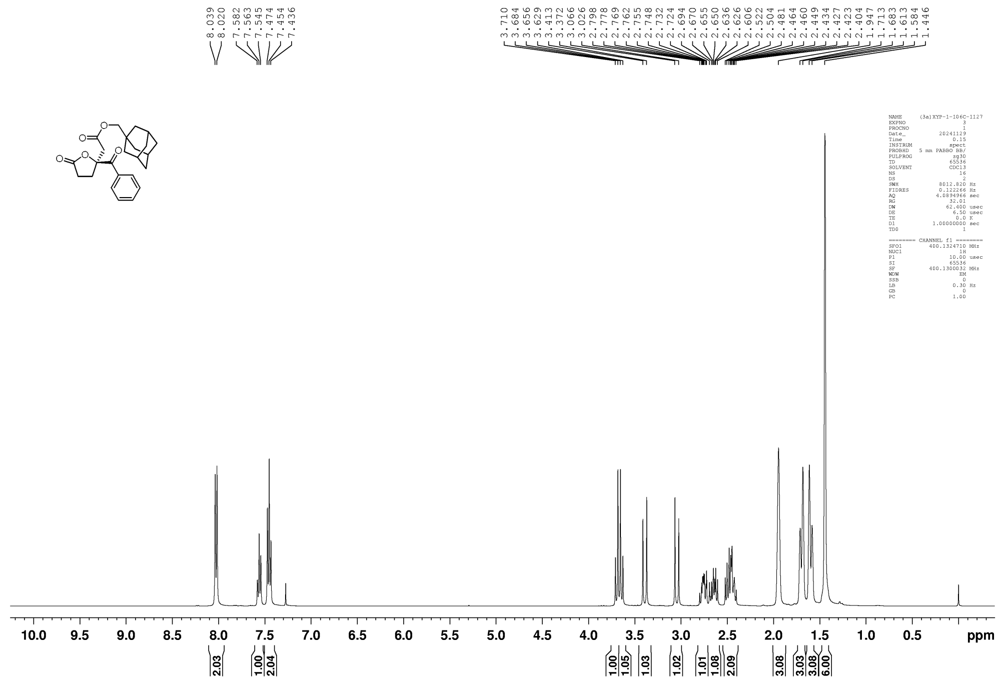

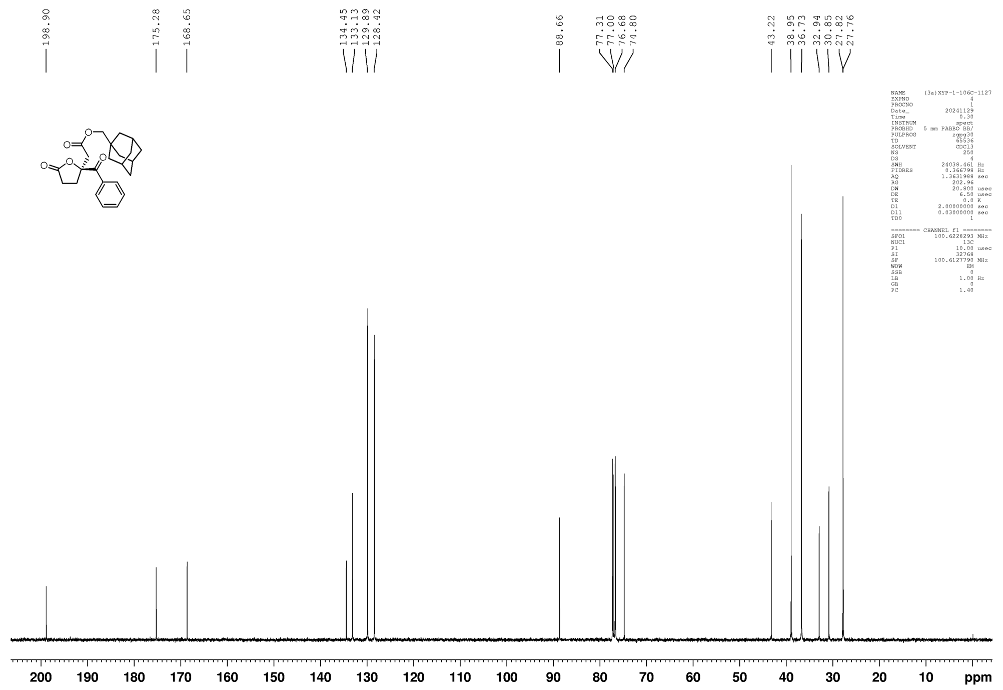

((1s,3R)-adamantan-1-yl)methyl 2-((S)-2-([1,1'-biphenyl]-4-carbonyl)-5-oxotetrahydrofuran-2-yl)acetate (3b)

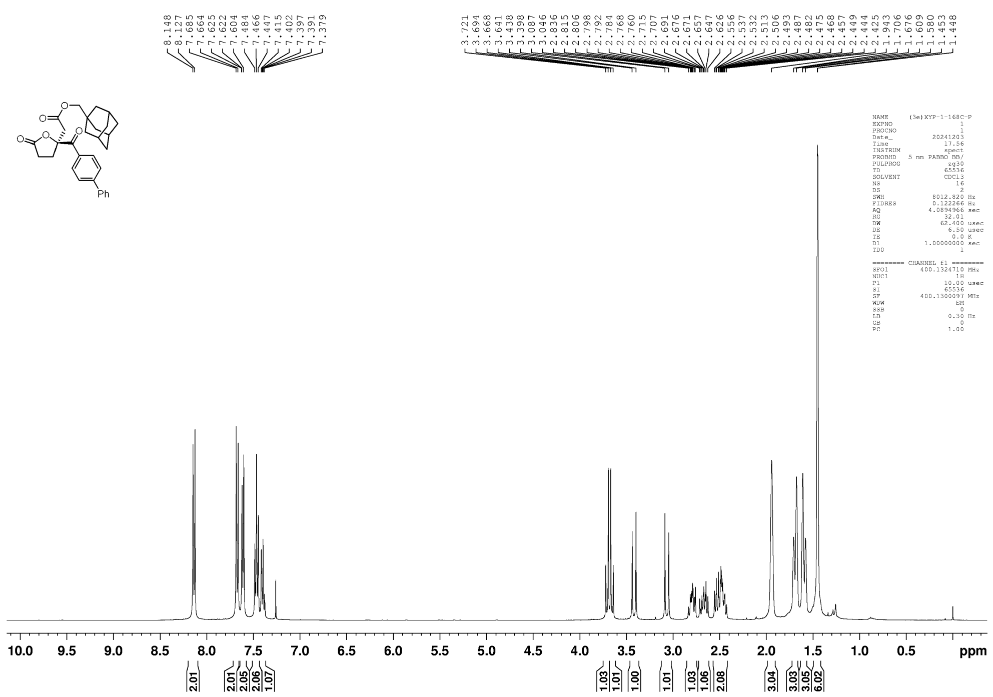

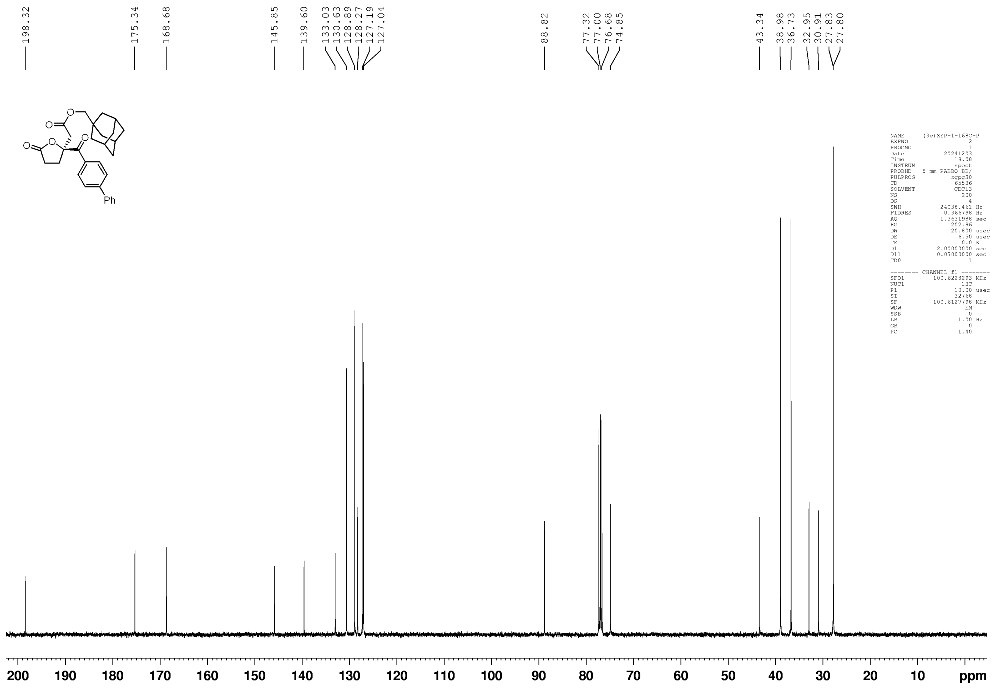

((1s,3R)-adamantan-1-yl)methyl 2-((S)-2-(4-methylbenzoyl)-5-oxotetrahydrofuran-2-yl)acetate (3c)

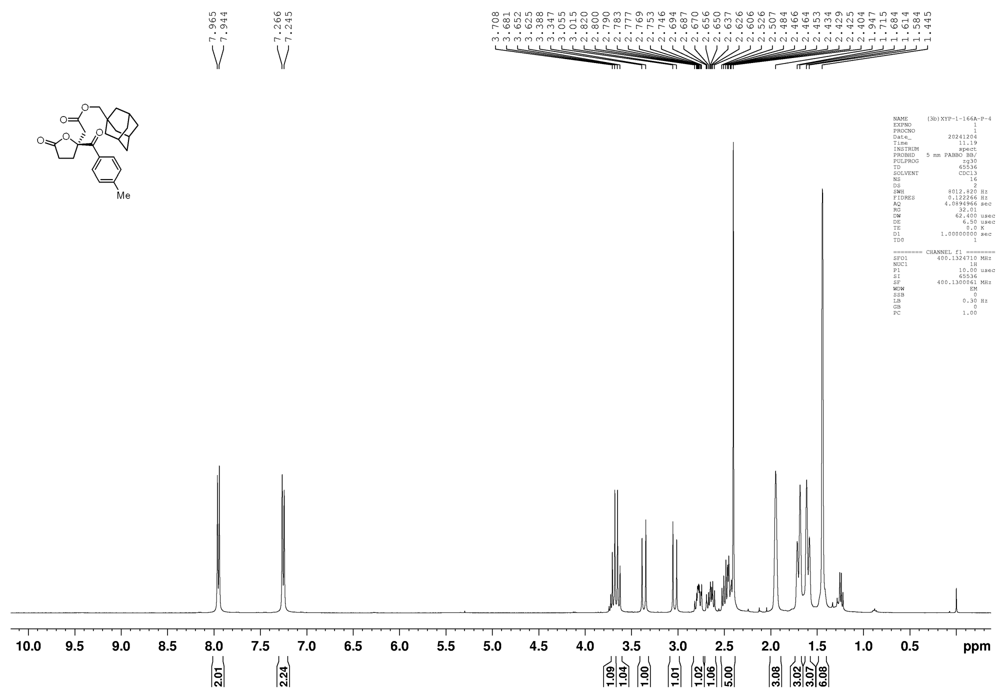

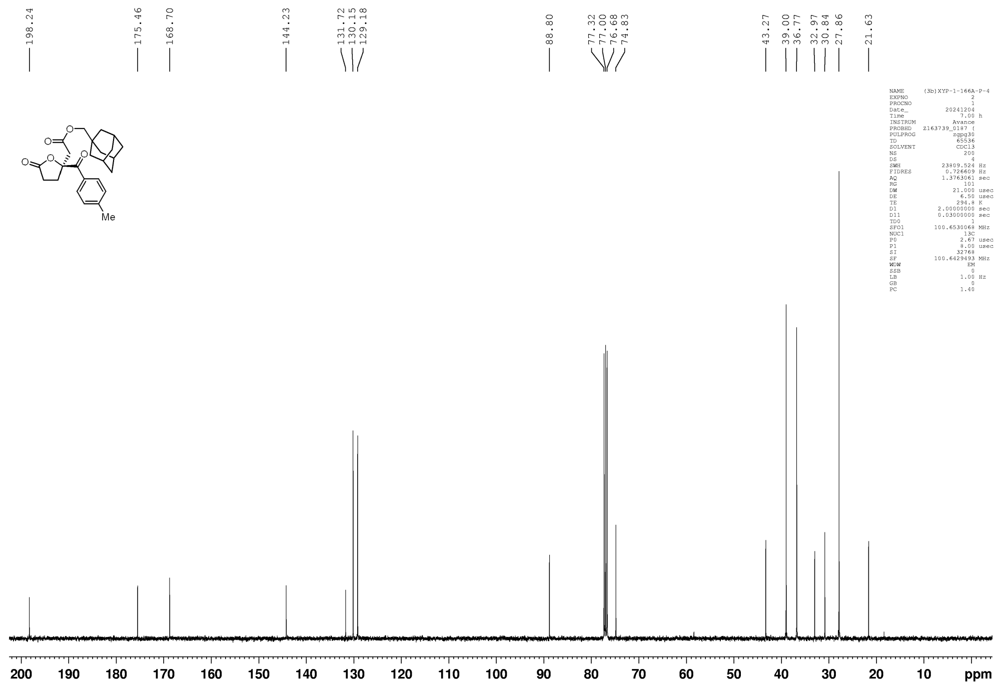

((1s,3R)-adamantan-1-yl)methyl 2-((S)-2-(4-methoxybenzoyl)-5-oxotetrahydrofuran-2-yl)acetate (3d)

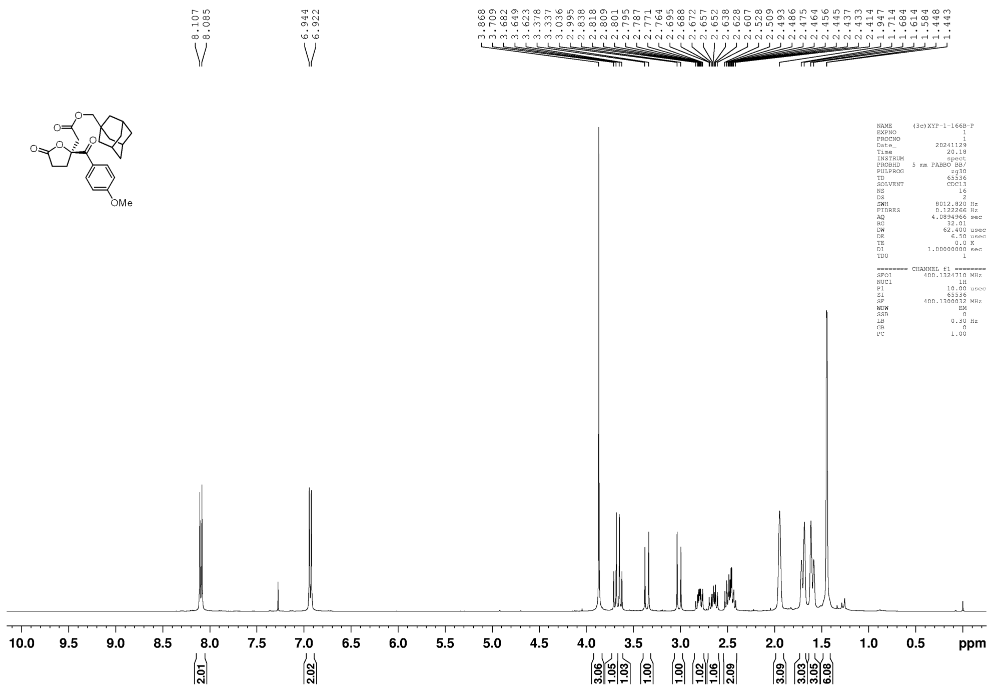

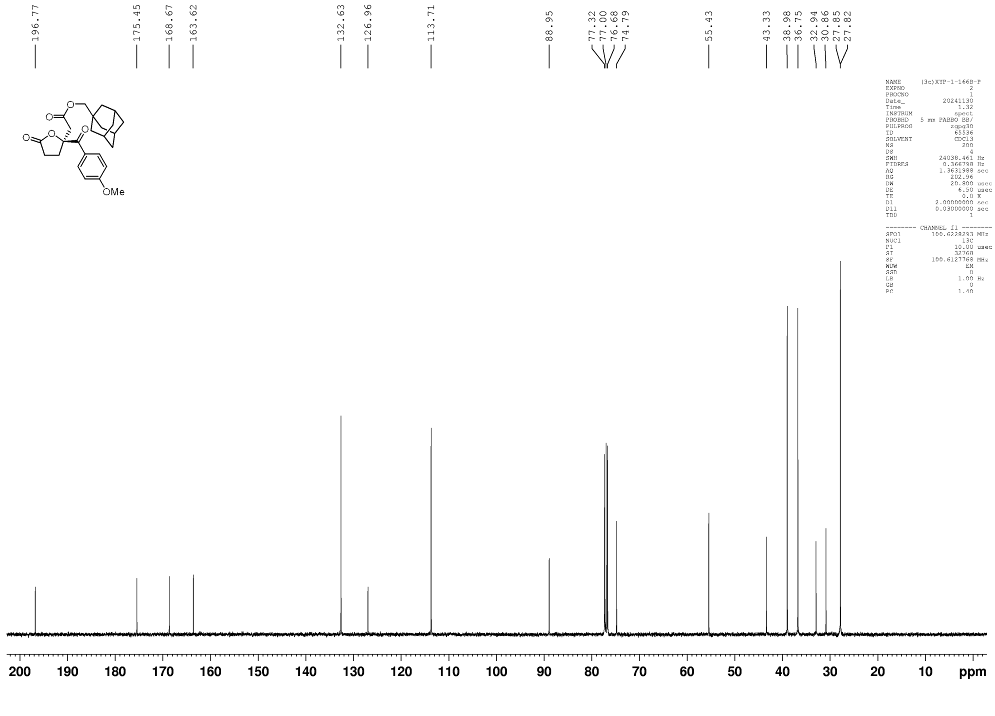

((1s,3R)-adamantan-1-yl)methyl 2-((S)-2-(4-(tert-butyl)benzoyl)-5-oxotetrahydrofuran-2-yl)acetate (3e)

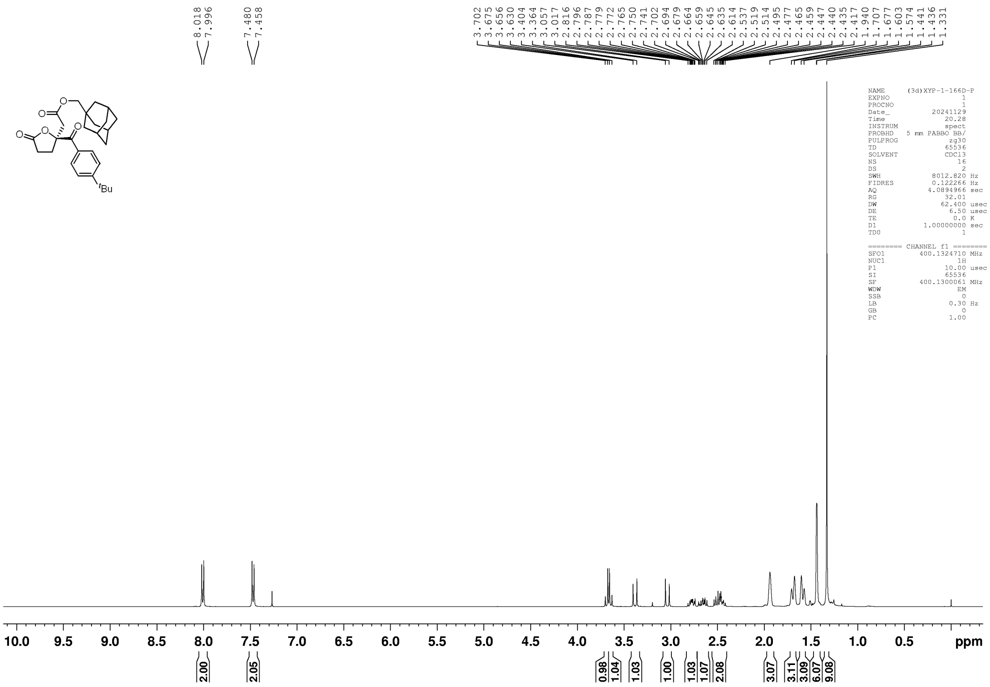

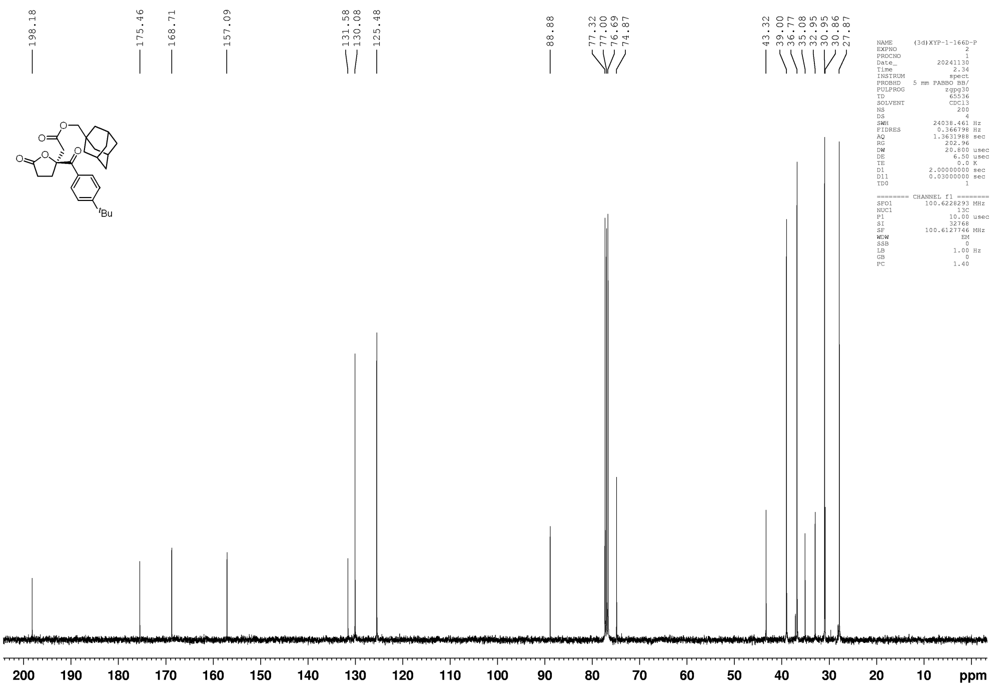

((1s,3R)-adamantan-1-yl)methyl 2-((S)-2-(4-(dimethylamino)benzoyl)-5-oxotetrahydrofuran-2-yl)acetate (3f)

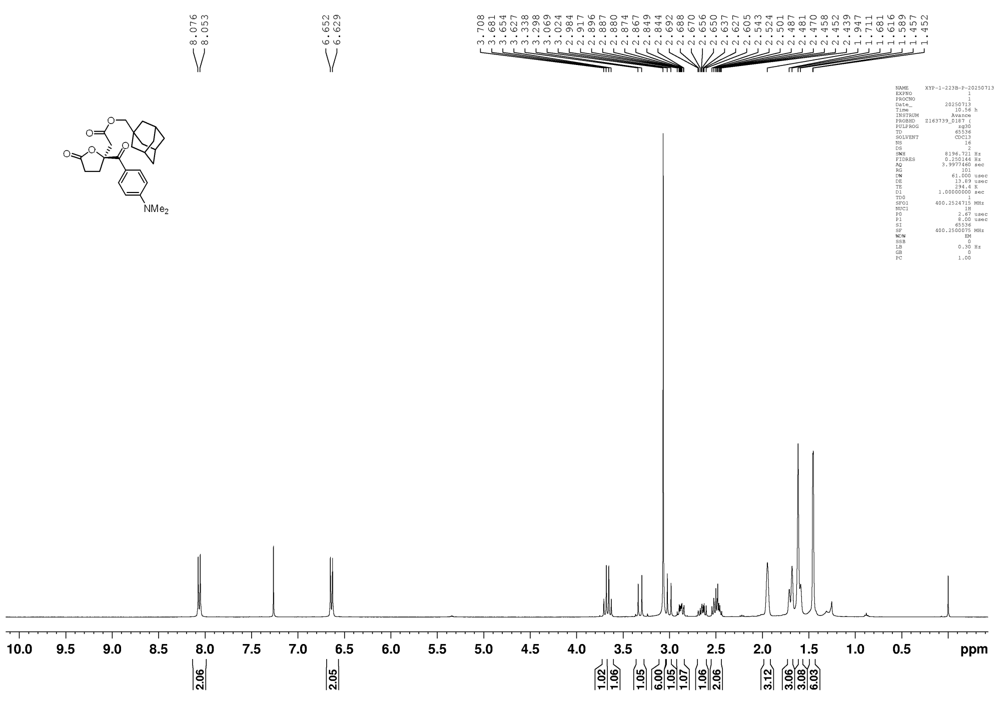

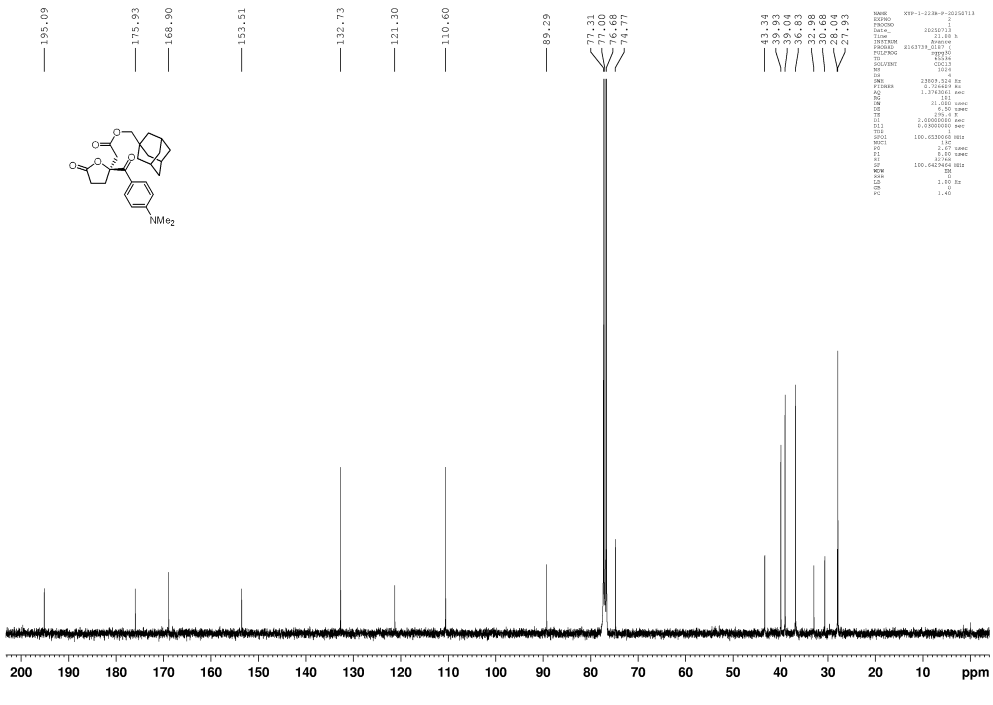

((1s,3R)-adamantan-1-yl)methyl 2-((S)-2-(4-fluorobenzoyl)-5-oxotetrahydrofuran-2-yl)acetate (3g)

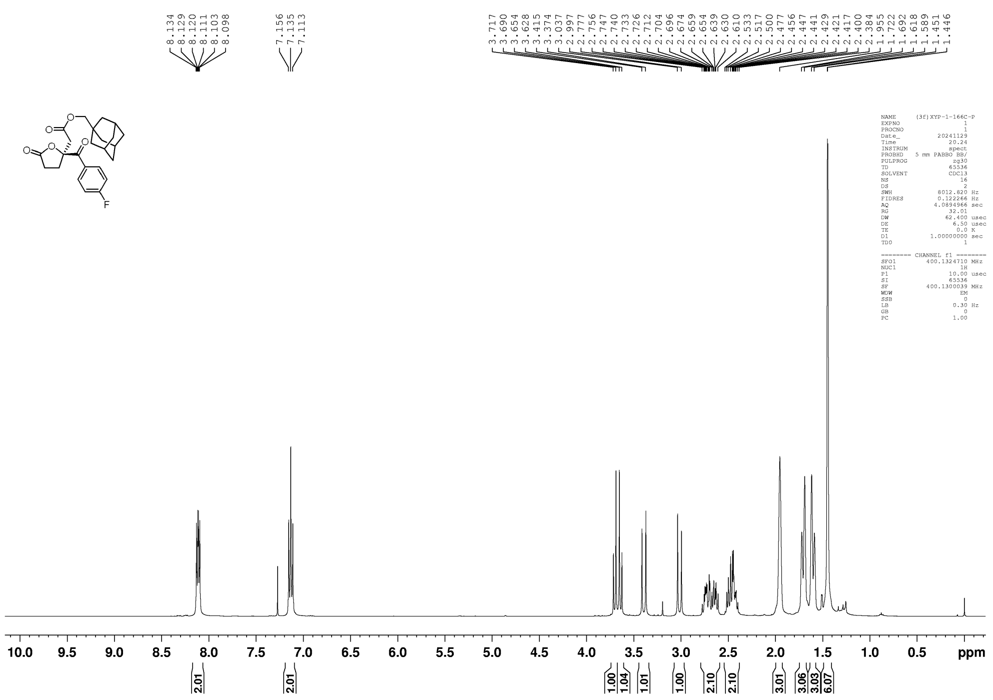

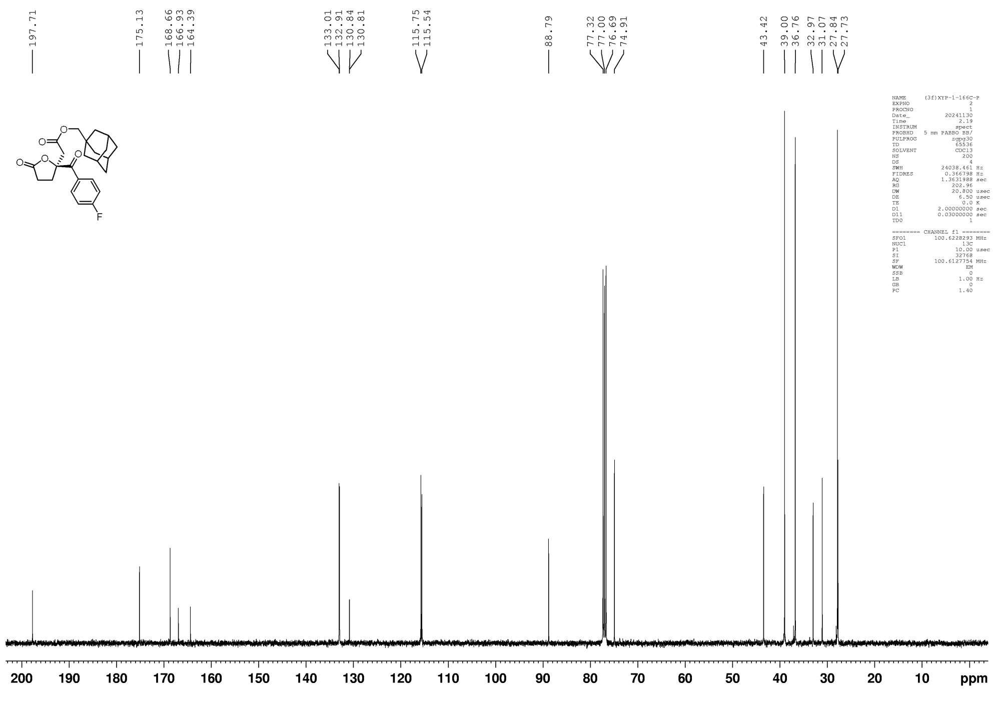

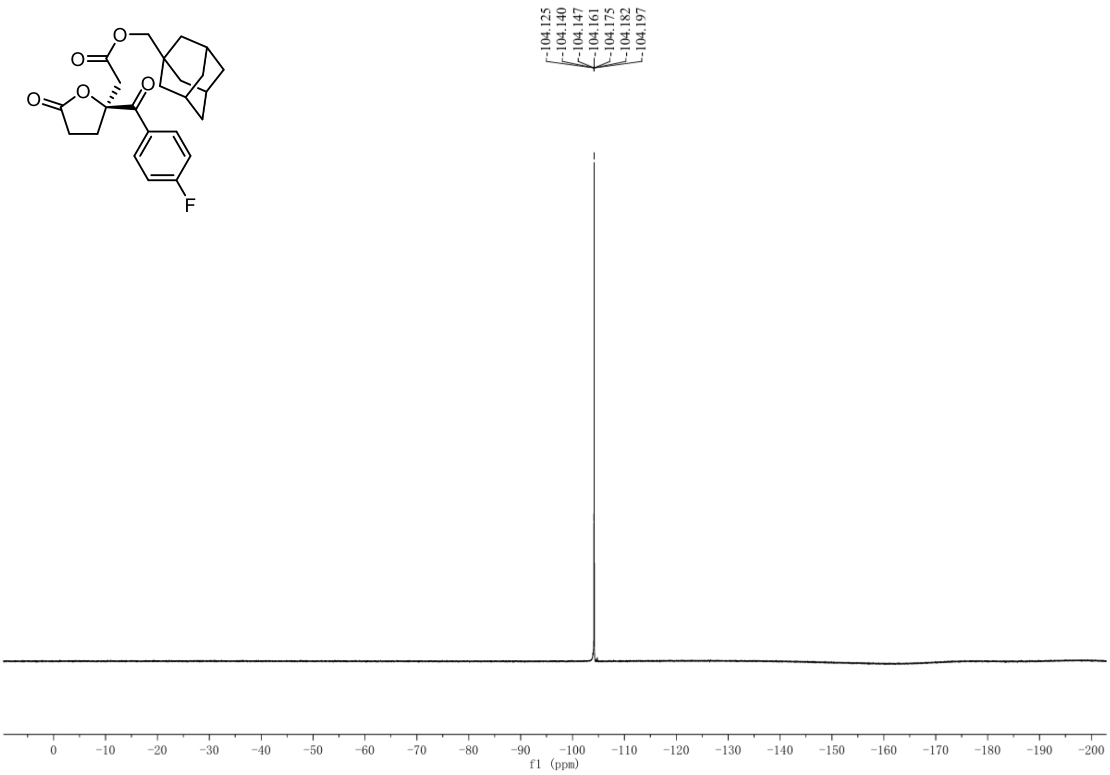
-104.125
-104.140
-104.147
-104.161
-104.175
-104.182
-104.197
0 −10 −20 −30 −40 −50 −60 −70 −80 −90 −100 −110 −120 −130 −140 −150 −160 −170 −180 −190 −200
f1 (ppm)

((1s,3R)-adamantan-1-yl)methyl 2-((S)-2-(4-chlorobenzoyl)-5-oxotetrahydrofuran-2-yl)acetate (3h)

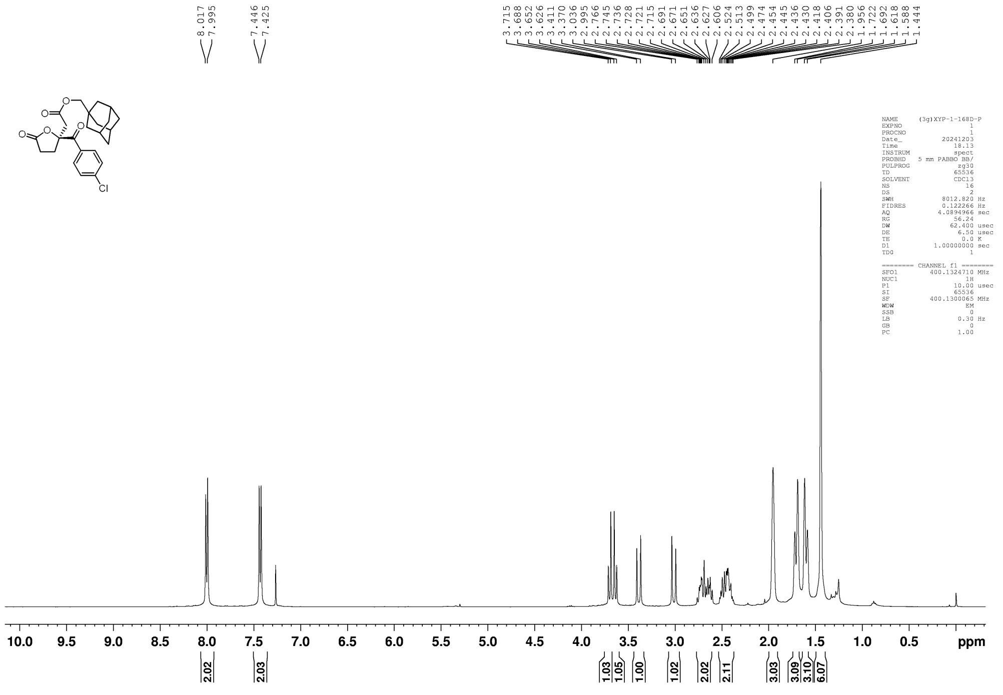

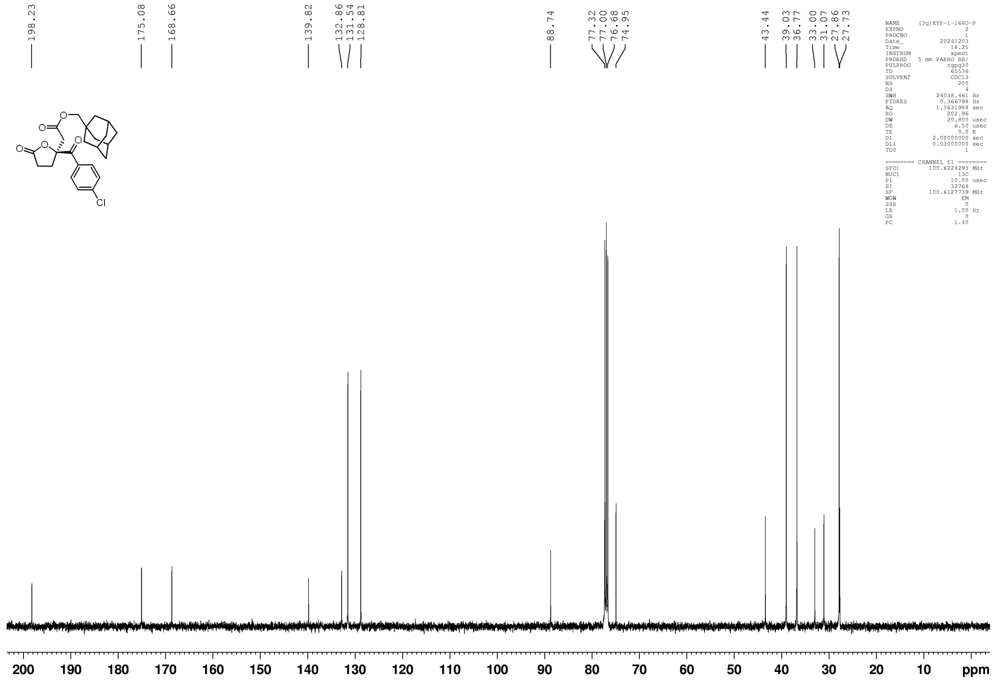

((1s,3R)-adamantan-1-yl)methyl 2-((S)-2-(4-bromobenzoyl)-5-oxotetrahydrofuran-2-yl)acetate (3i)

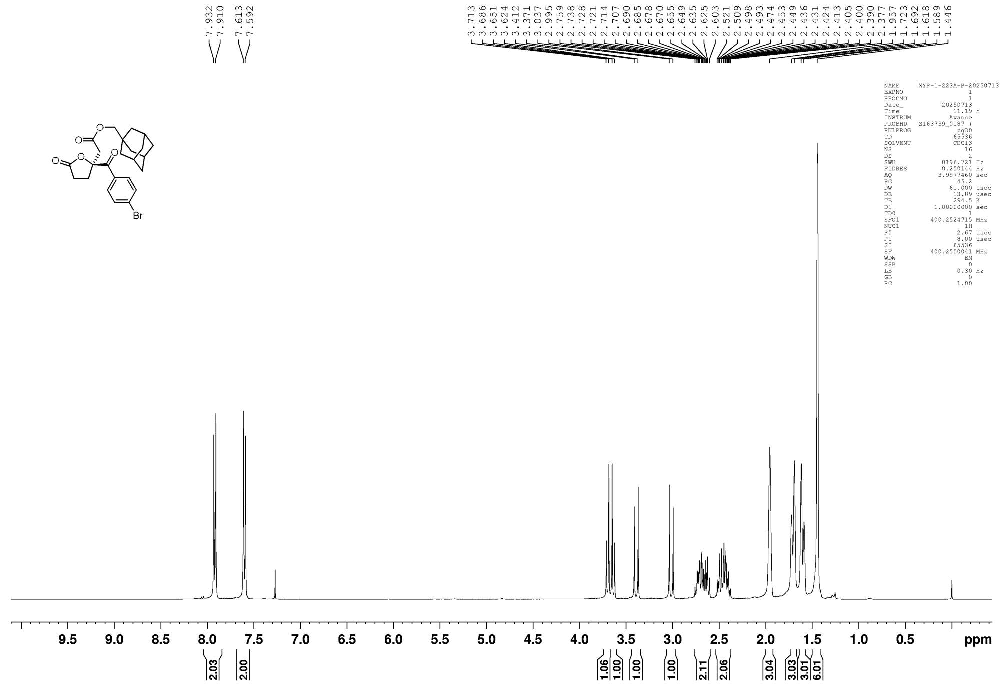

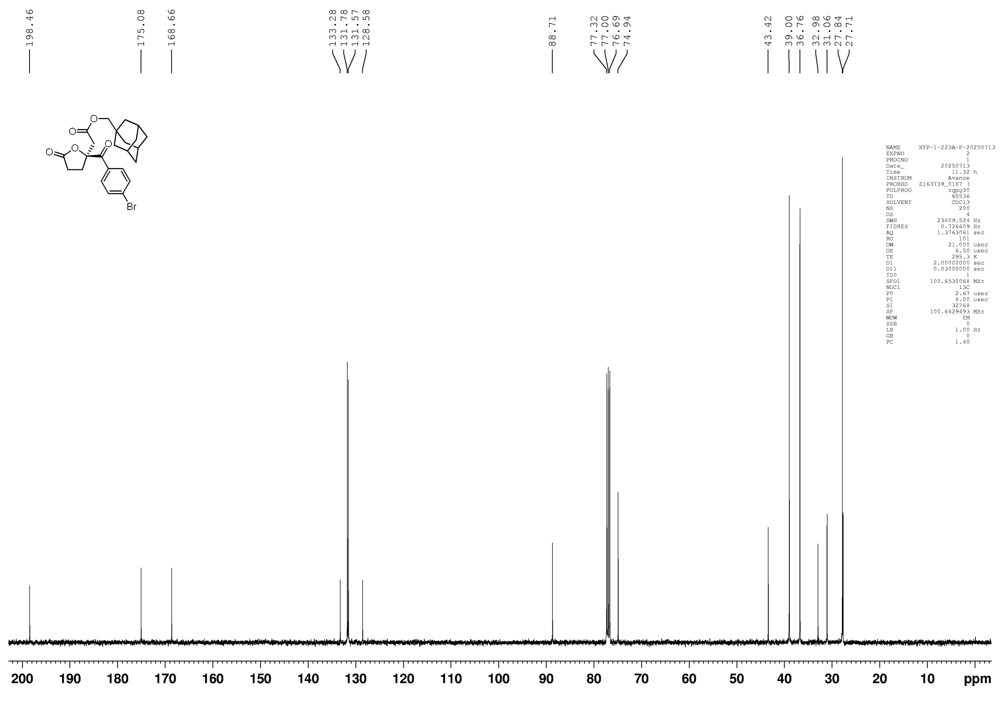

((1s,3R)-adamantan-1-yl)methyl 2-((S)-2-(4-cyanobenzoyl)-5-oxotetrahydrofuran-2-yl)acetate (3j)

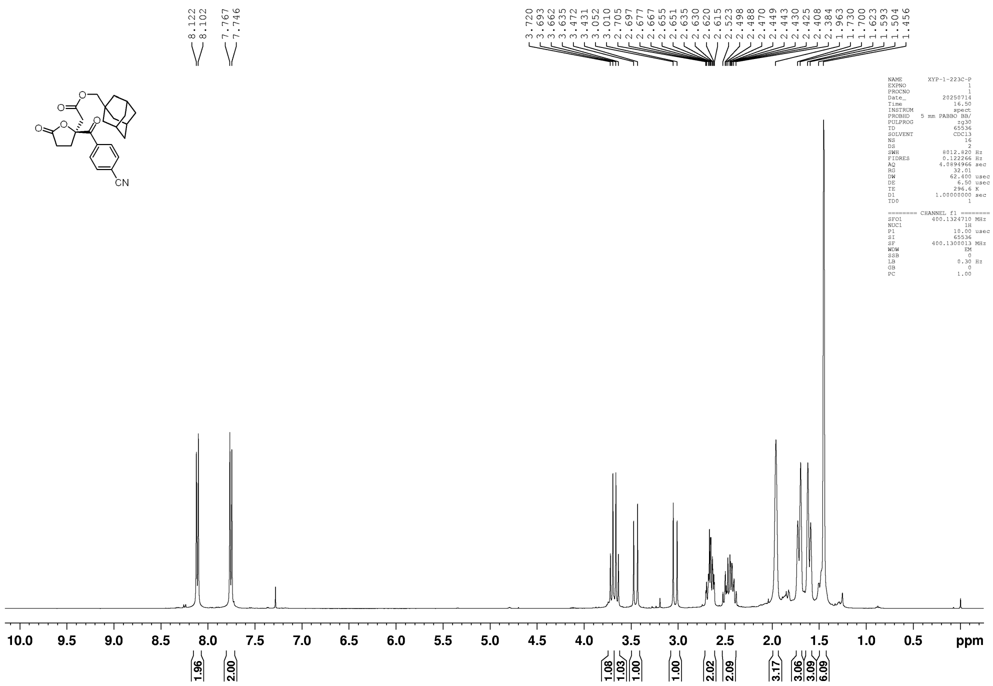

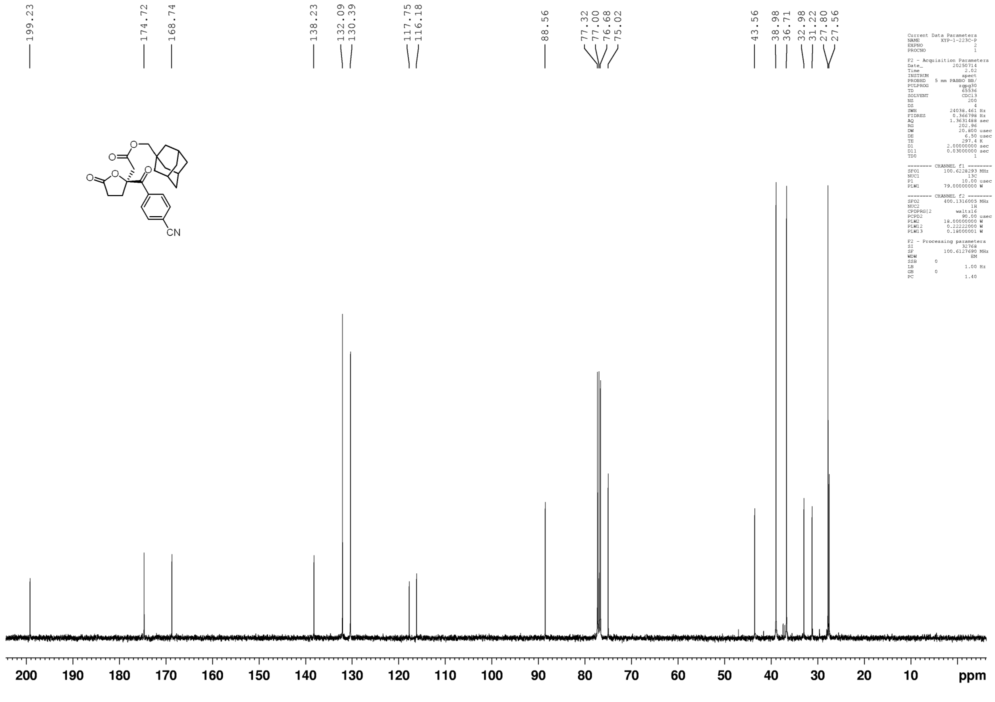

((1s,3R)-adamantan-1-yl)methyl 2-((S)-5-oxo-2-(4-(trifluoromethyl)benzoyl) tetrahydrofuran-2-yl)acetate (3k)

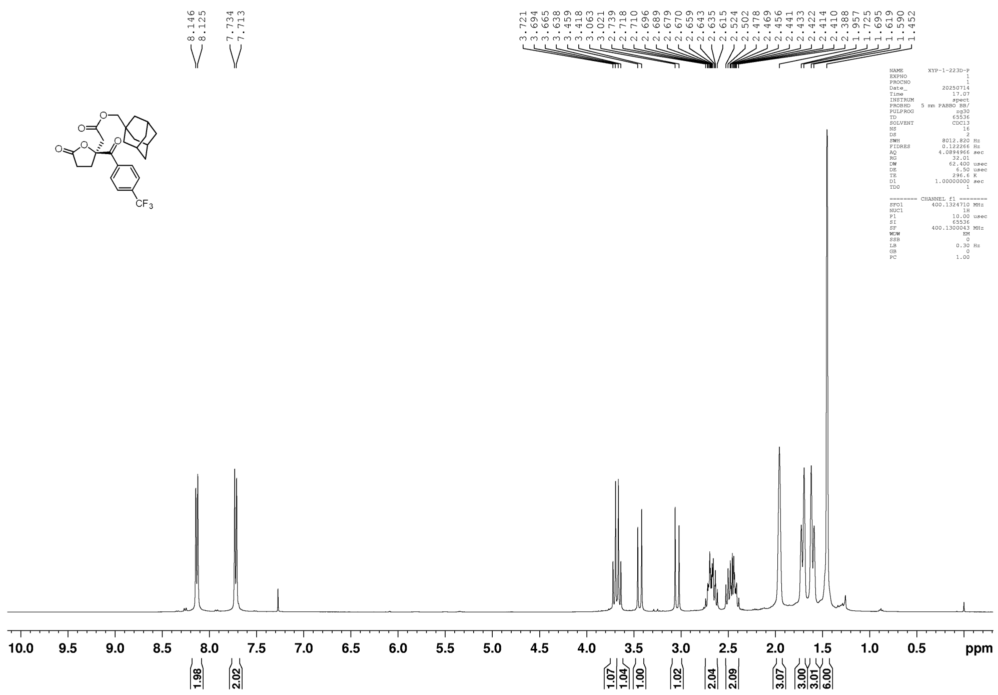

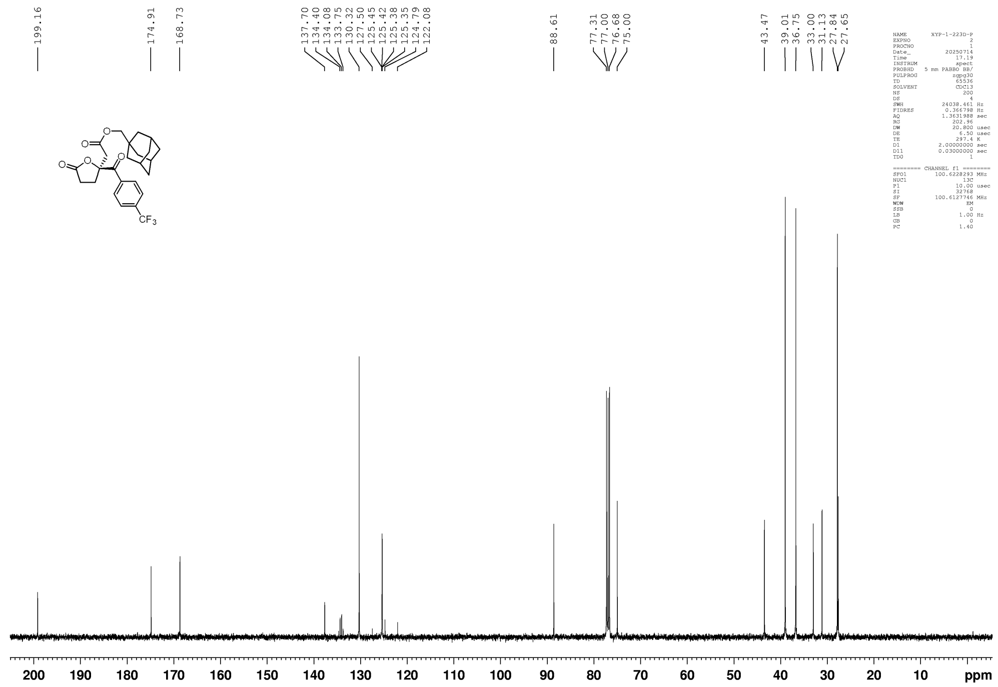

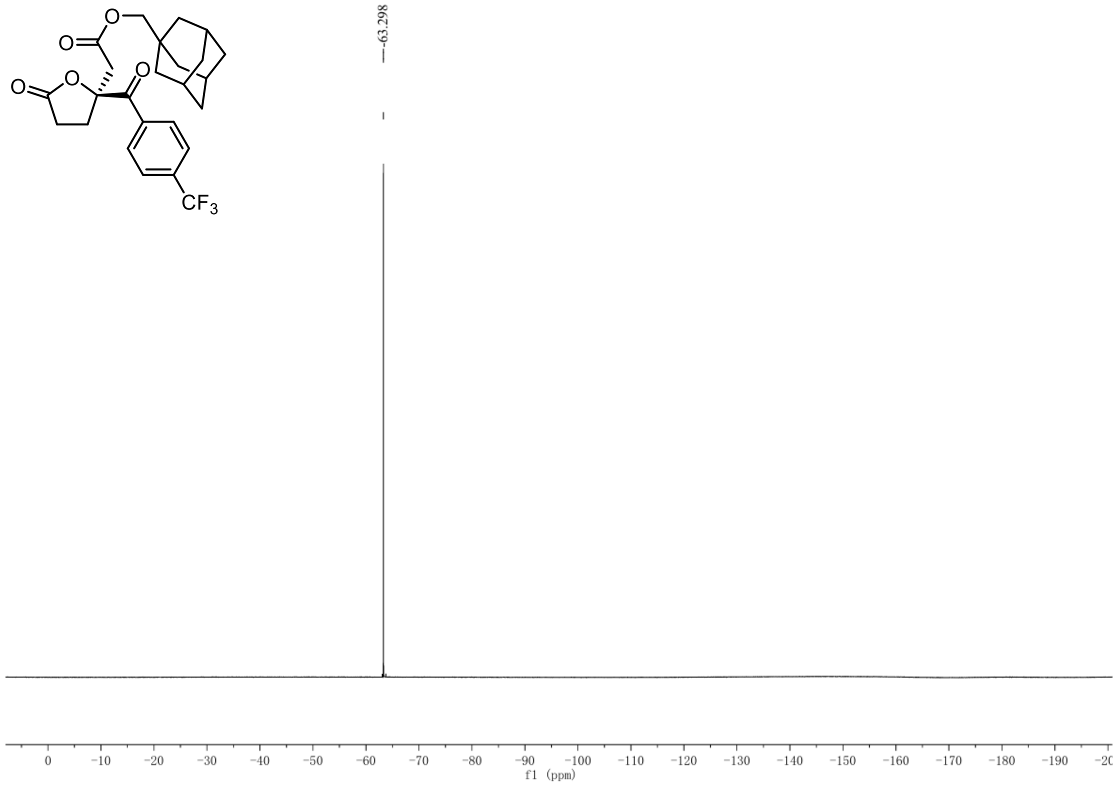

O
O
O
O
O
CF3
-63.298
0
-10
-20
-30
-40
-50
-60
-70
-80
-90
-100
-110
-120
-130
-140
-150
-160
-170
-180
-190
f1 (ppm)

((1s,3R)-adamantan-1-yl)methyl 2-((S)-2-(3-methylbenzoyl)-5-oxotetrahydrofuran-2-yl)acetate (3l)

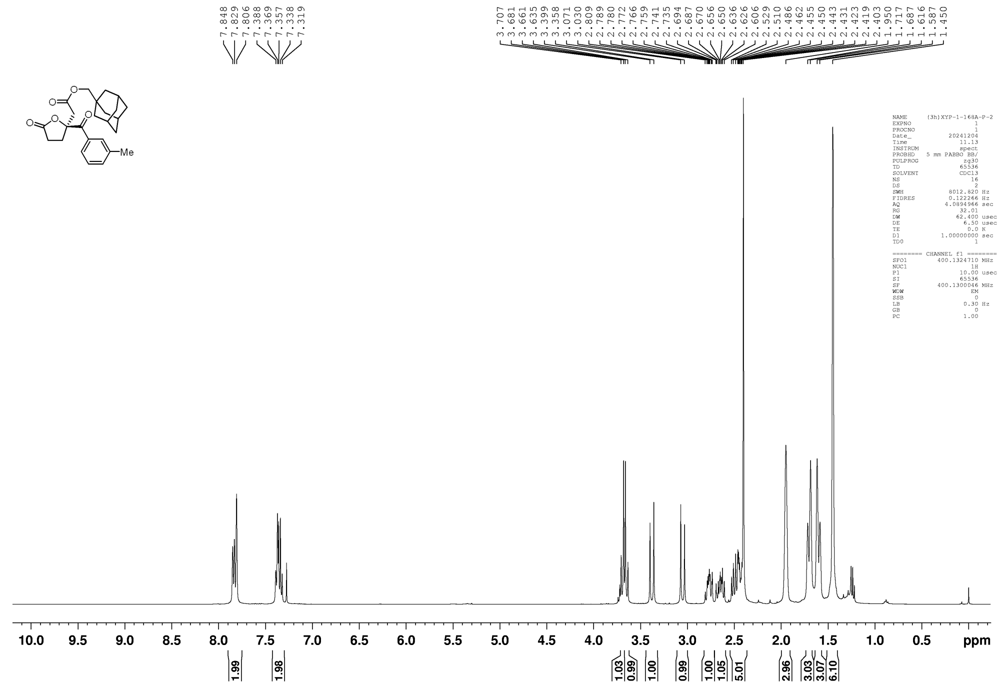

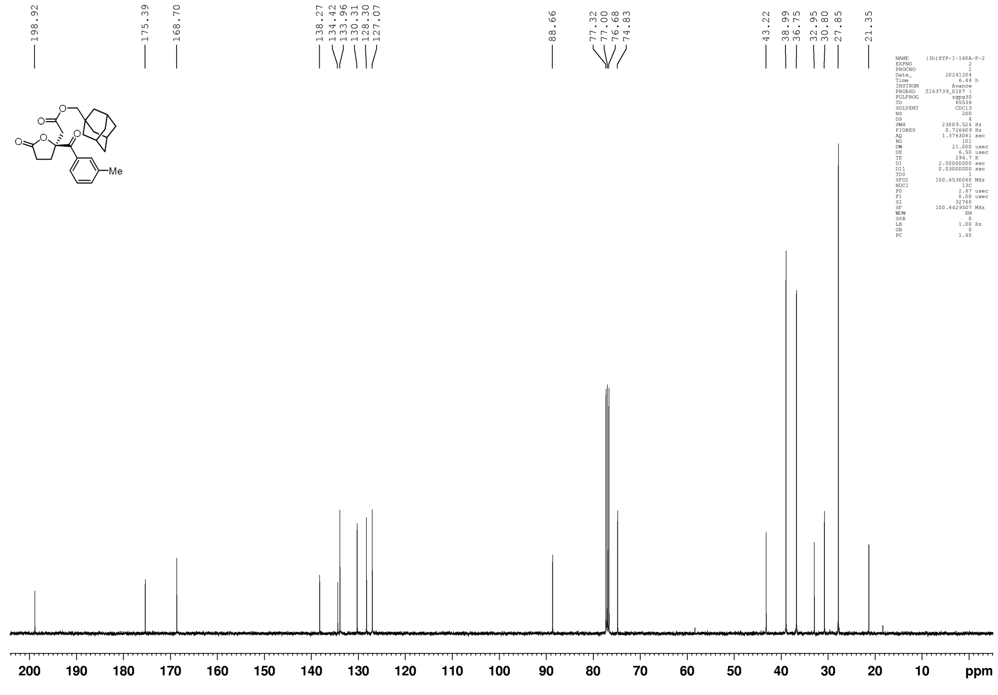

((1s,3R)-adamantan-1-yl)methyl 2-((S)-2-(3-methoxybenzoyl)-5-oxotetrahydrofuran-2-yl)acetate (3m)

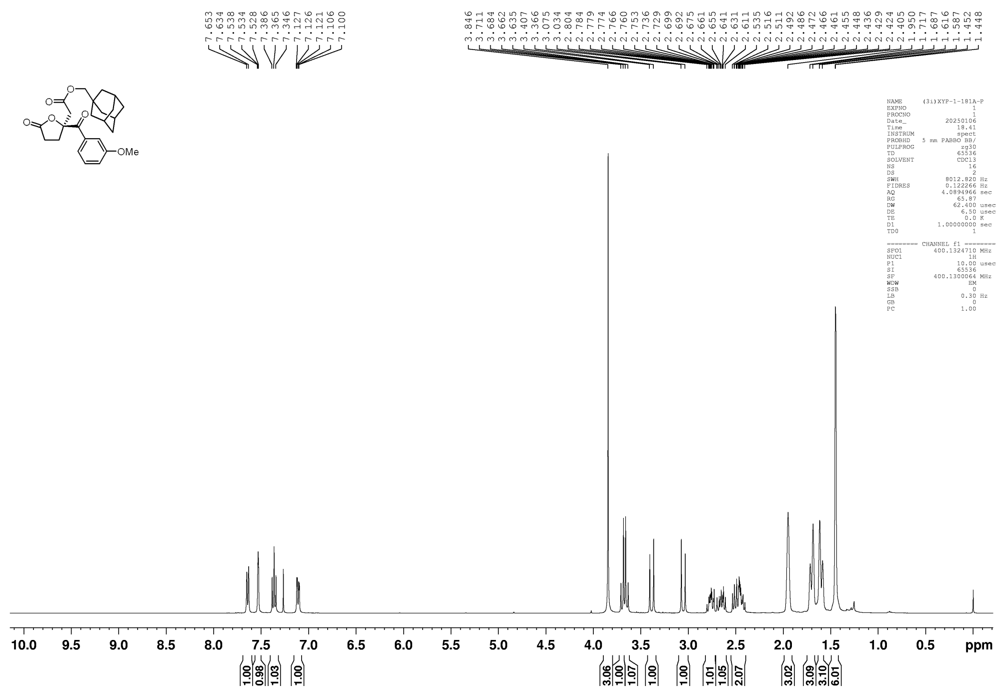

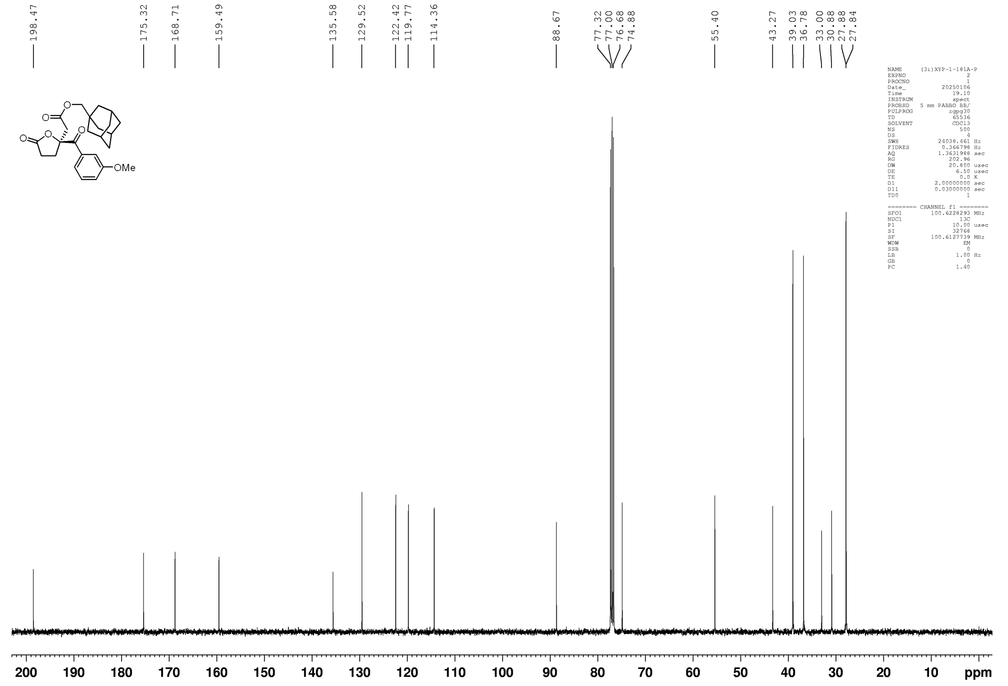

((1s,3R)-adamantan-1-yl)methyl 2-((S)-2-(2-methylbenzoyl)-5-oxotetrahydrofuran-2-yl)acetate (3n)

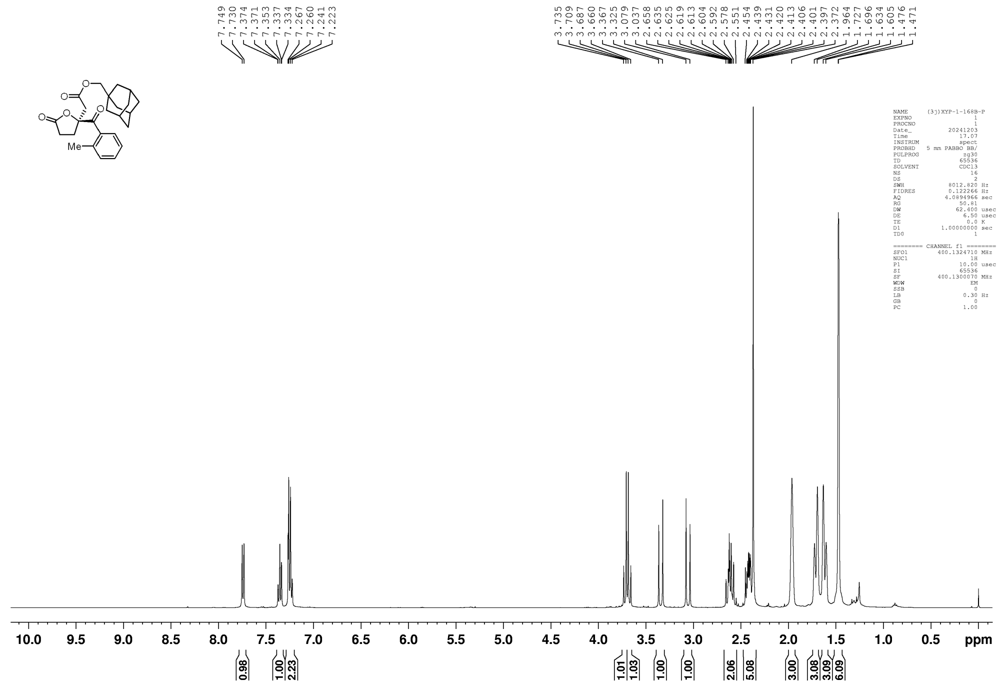

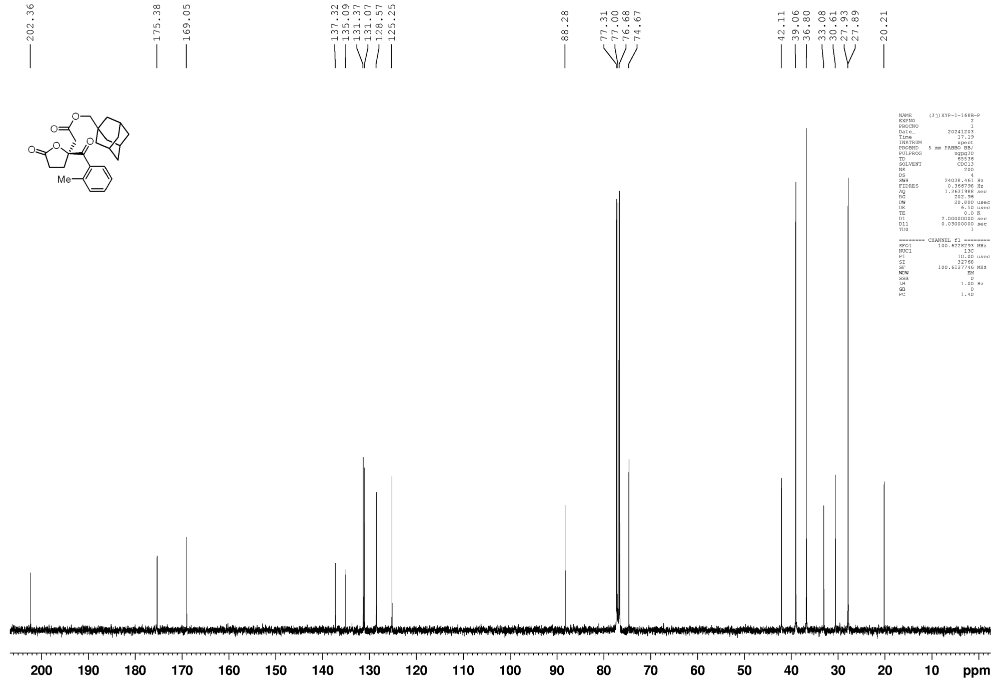

((1s,3R)-adamantan-1-yl)methyl 2-((S)-2-(2-naphthoyl)-5-oxotetrahydrofuran-2-yl)acetate (3o)

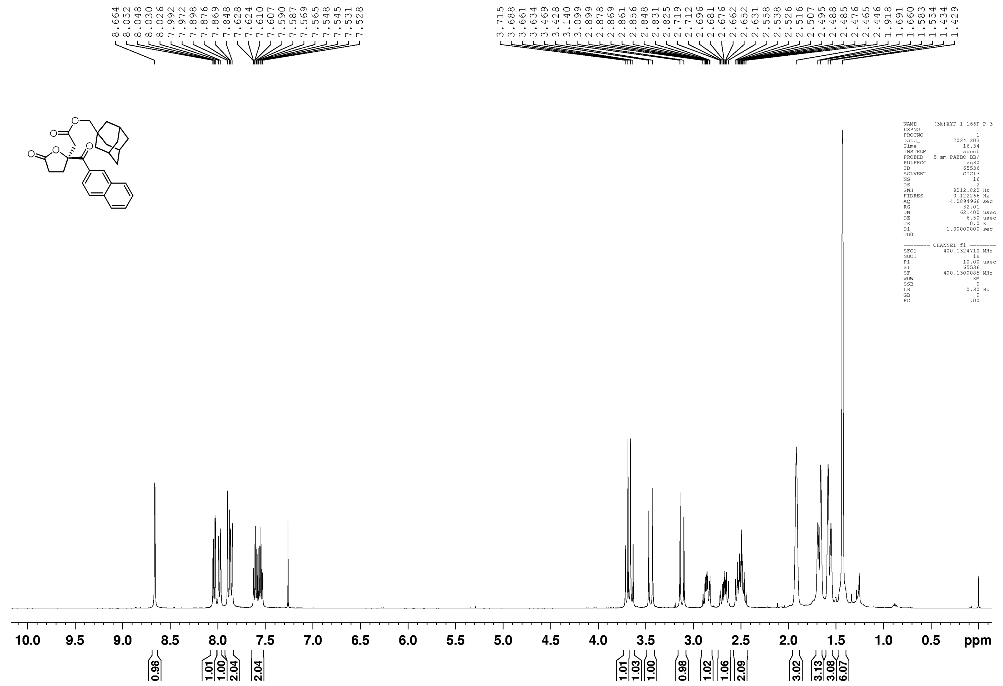

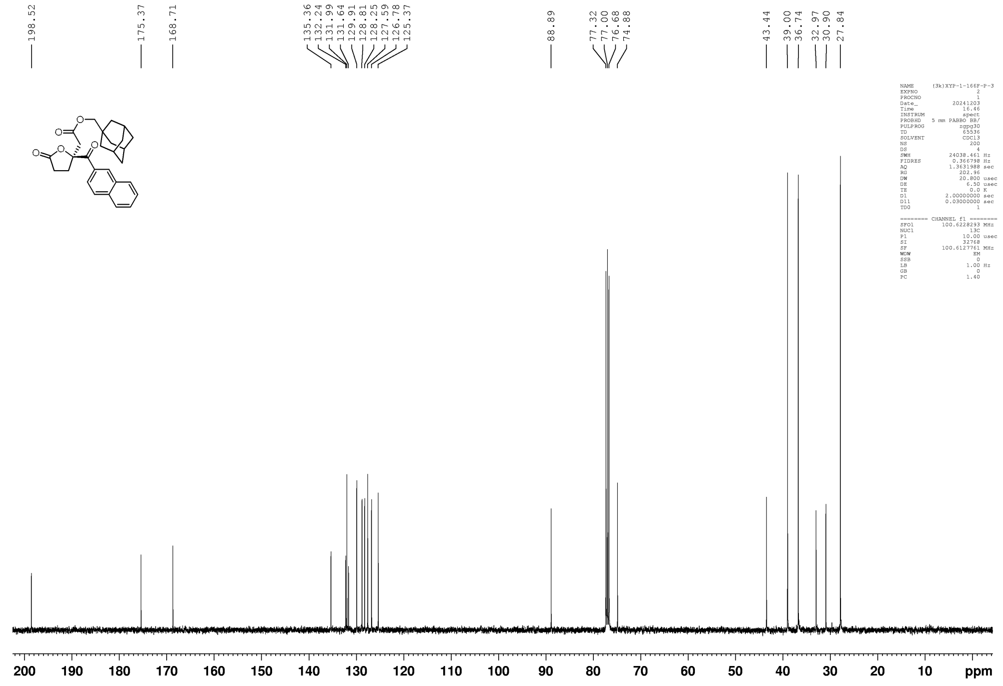

((1s,3R)-adamantan-1-yl)methyl 2-((S)-5-oxo-2-(thiophene-2-carbonyl)tetrahydrofuran-2-yl)acetate (3p)

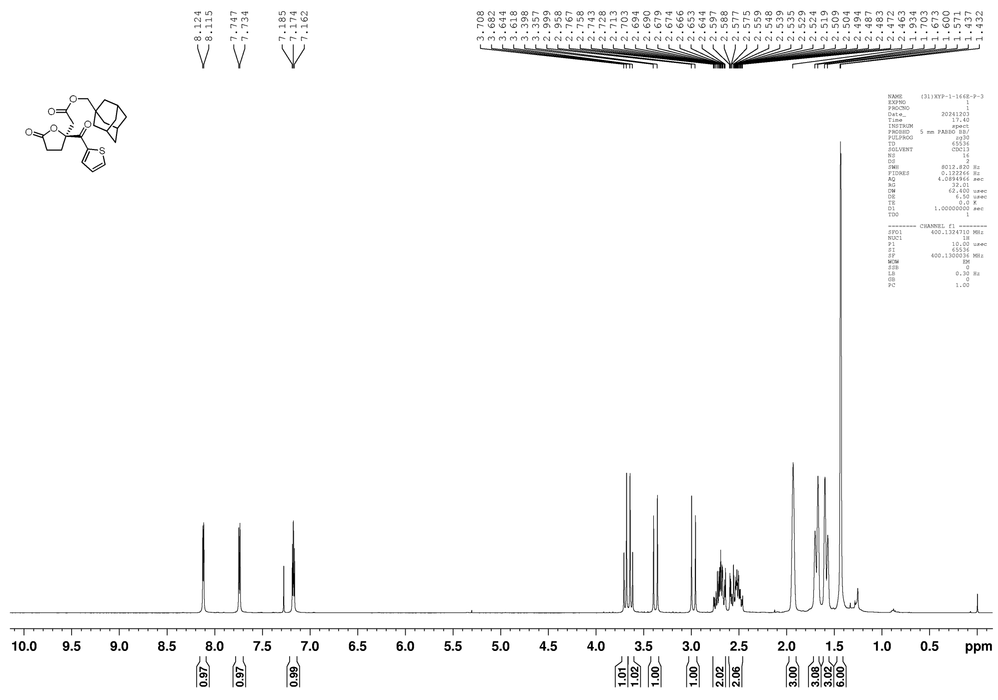

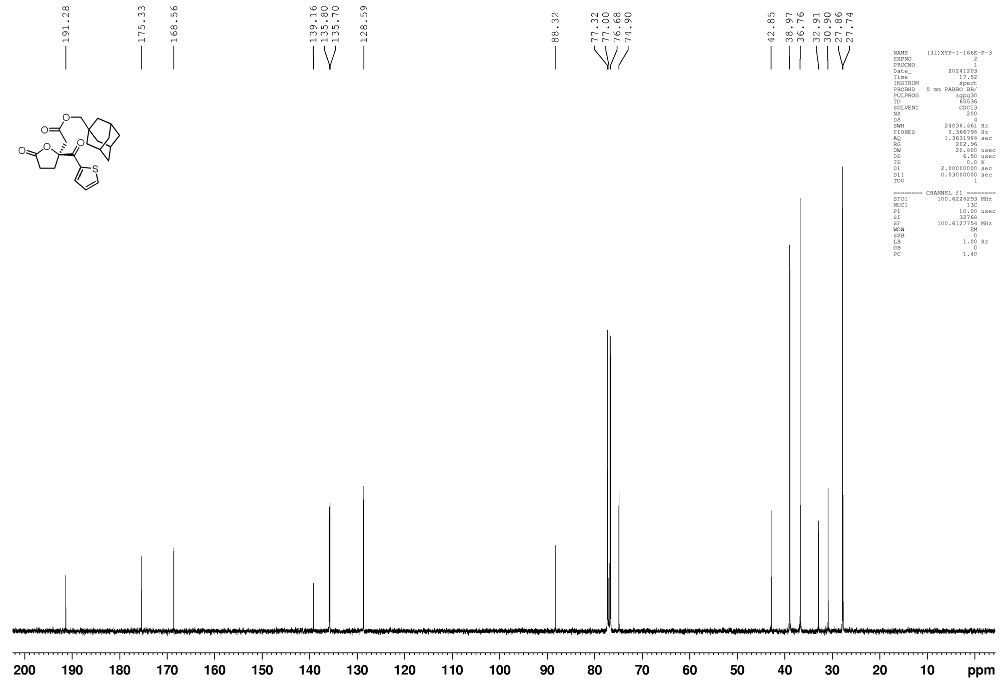

((1s,3R)-adamantan-1-yl)methyl 2-((S)-2-(furan-2-carbonyl)-5-oxotetrahydrofuran-2-yl)acetate (3q)

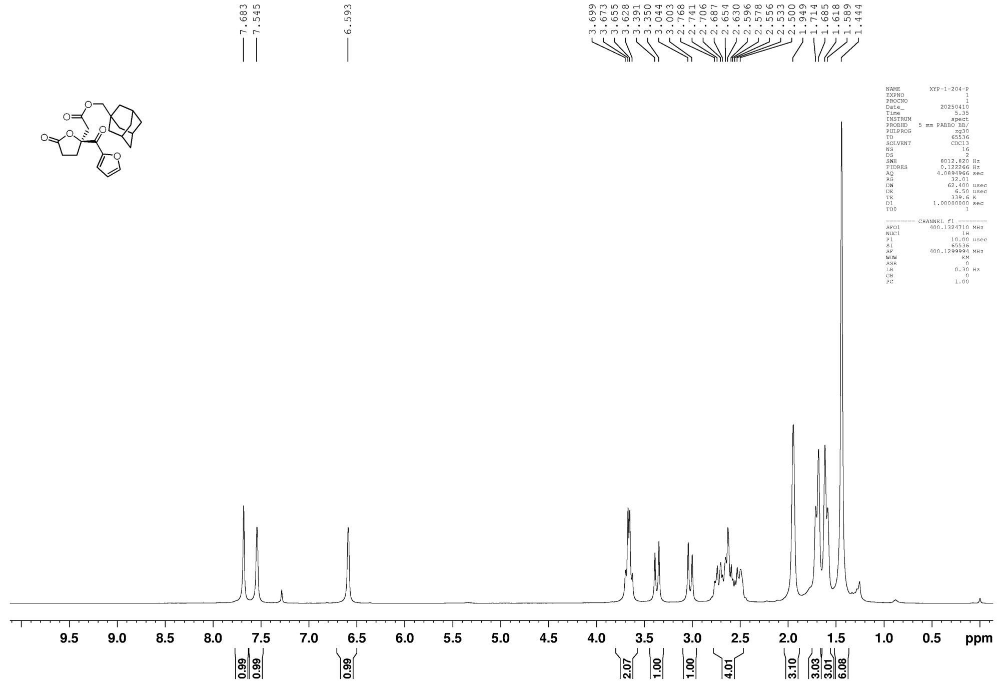

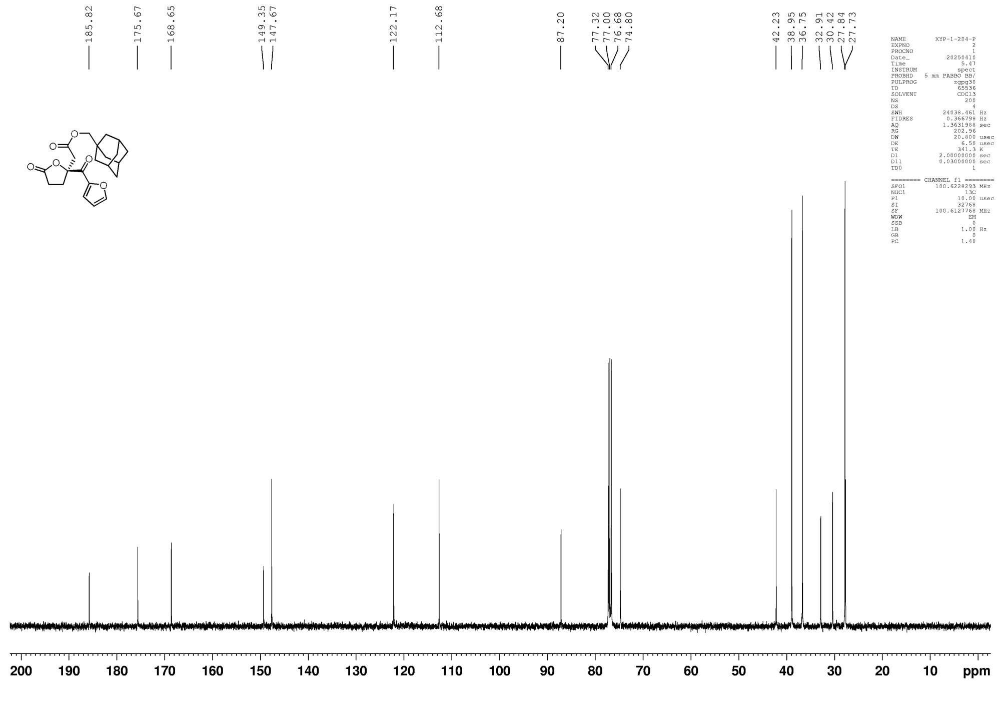

((1s,3R)-adamantan-1-yl)methyl 2-((S)-5-oxo-2-phenyltetrahydrofuran-2-yl)acetate (3r)

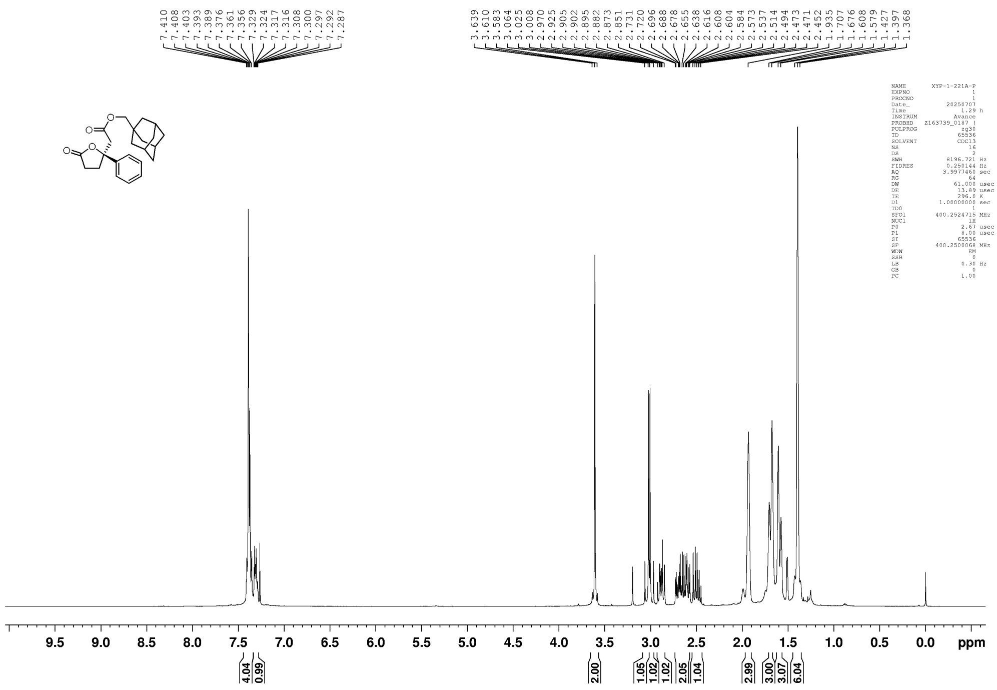

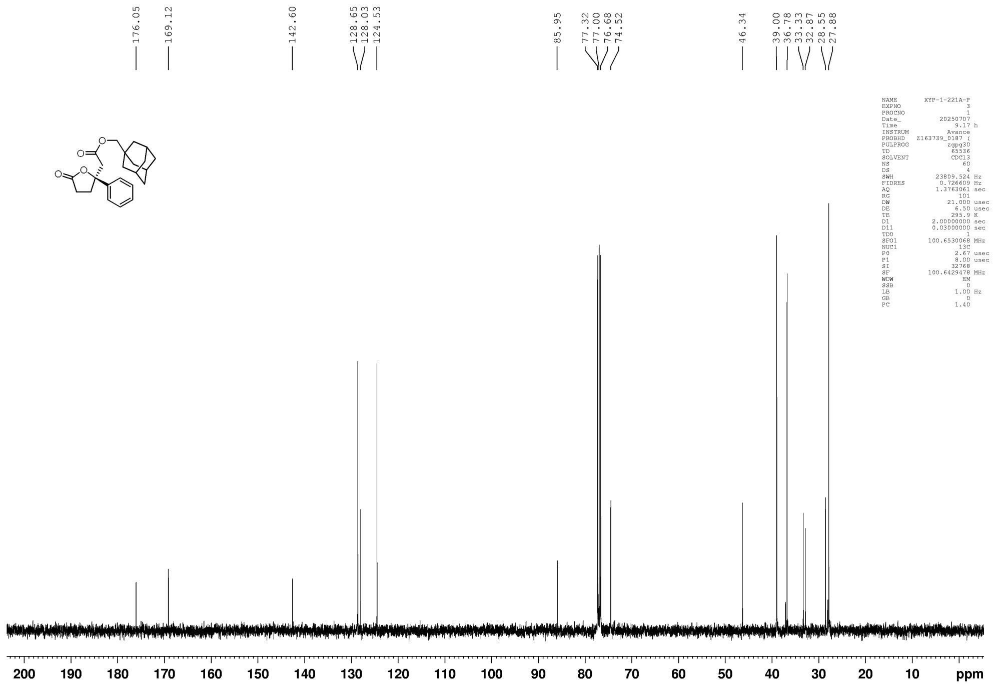

((1s,3R)-adamantan-1-yl)methyl 2-((S)-2-([1,1'-biphenyl]-4-yl)-5-oxotetrahydrofuran-2-yl)acetate (3s)

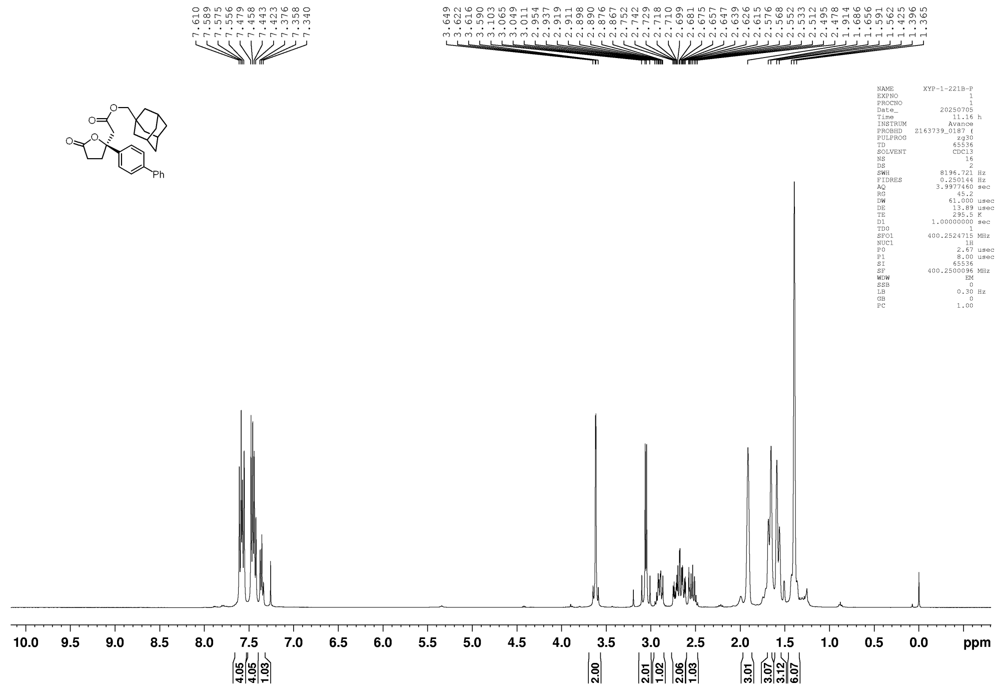

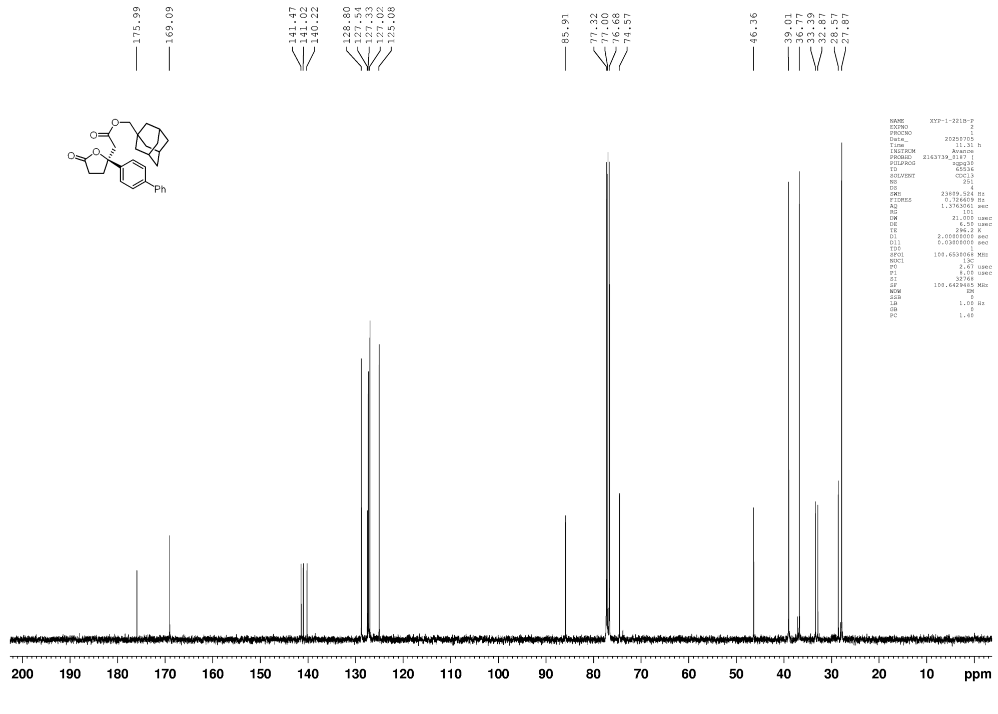

((1s,3R)-adamantan-1-yl)methyl 2-((S)-2-(4-chlorophenyl)-5-oxotetrahydrofuran-2-yl)acetate (3t)

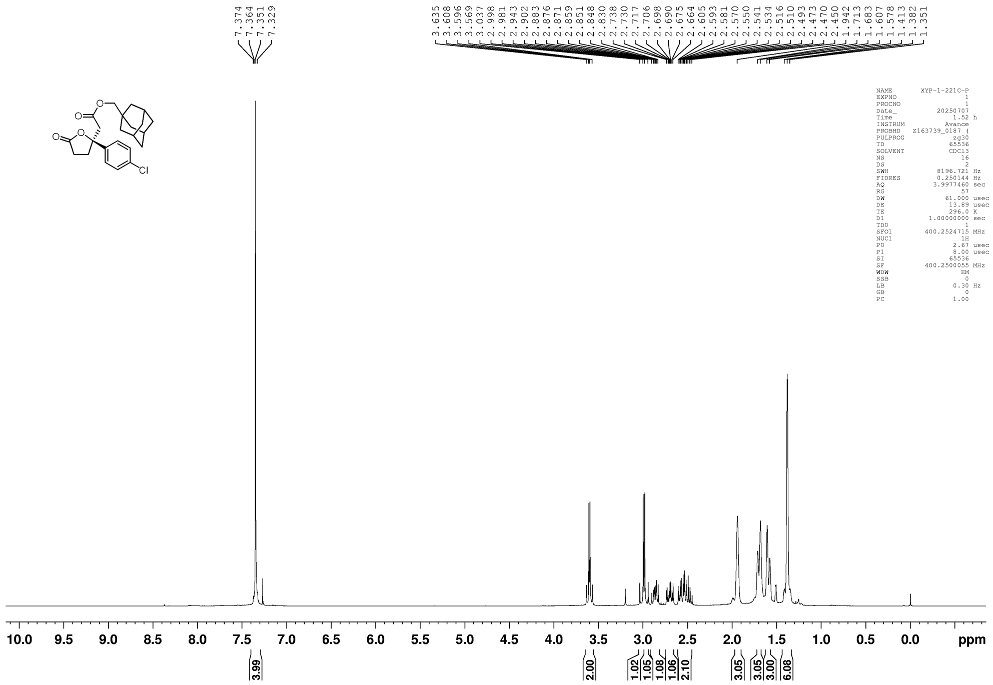

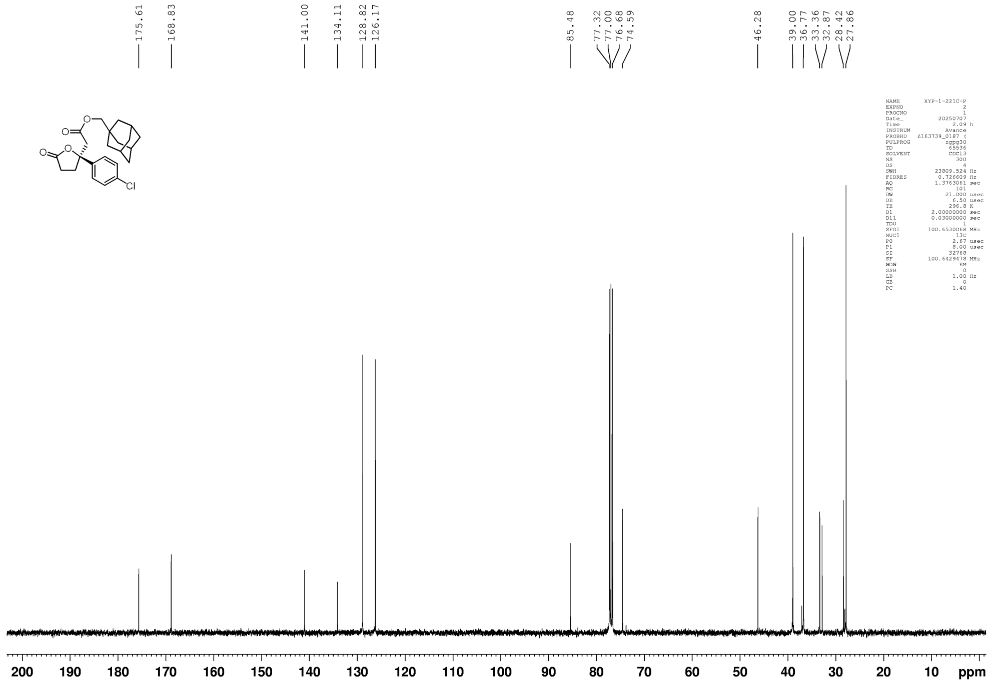

methyl (S)-2-(2-benzoyl-5-oxotetrahydrofuran-2-yl)acetate (3u)

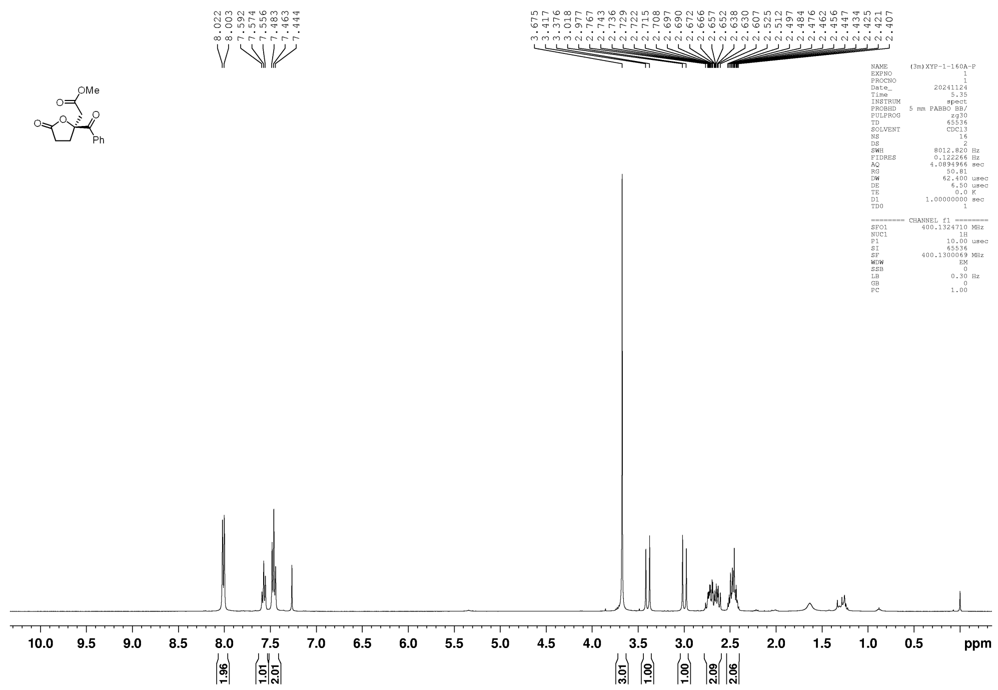

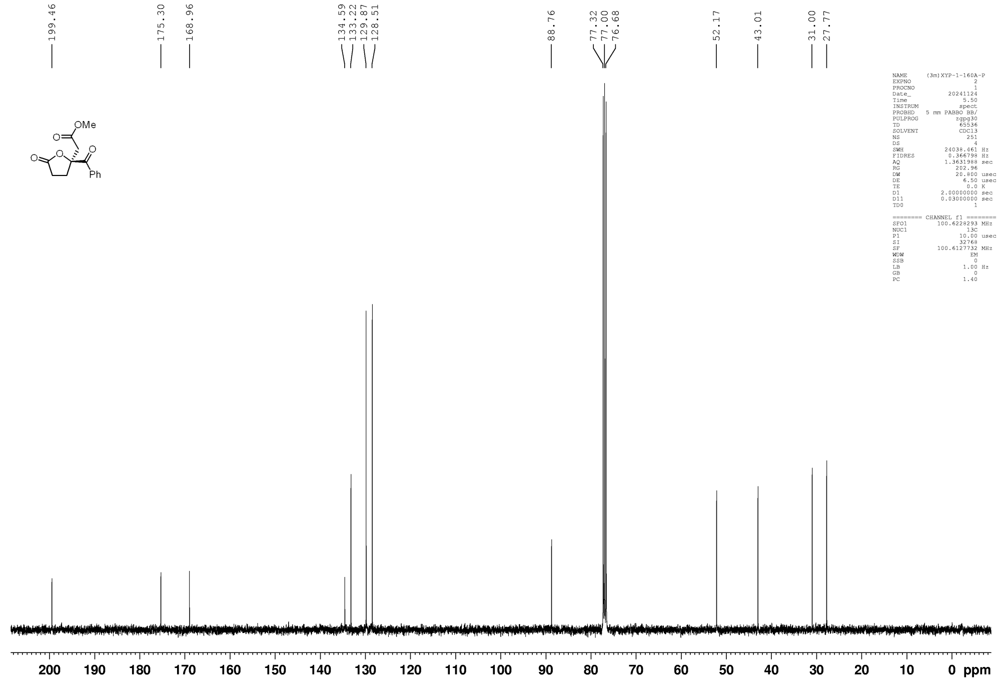

methyl-d3 (S)-2-(2-benzoyl-5-oxotetrahydrofuran-2-yl)acetate (3v)

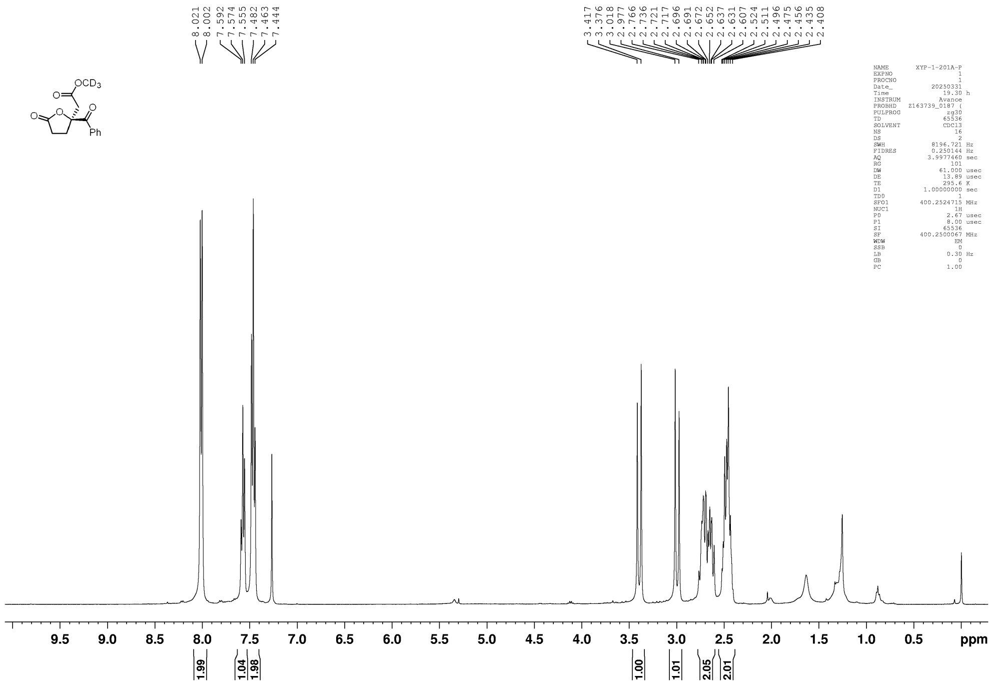

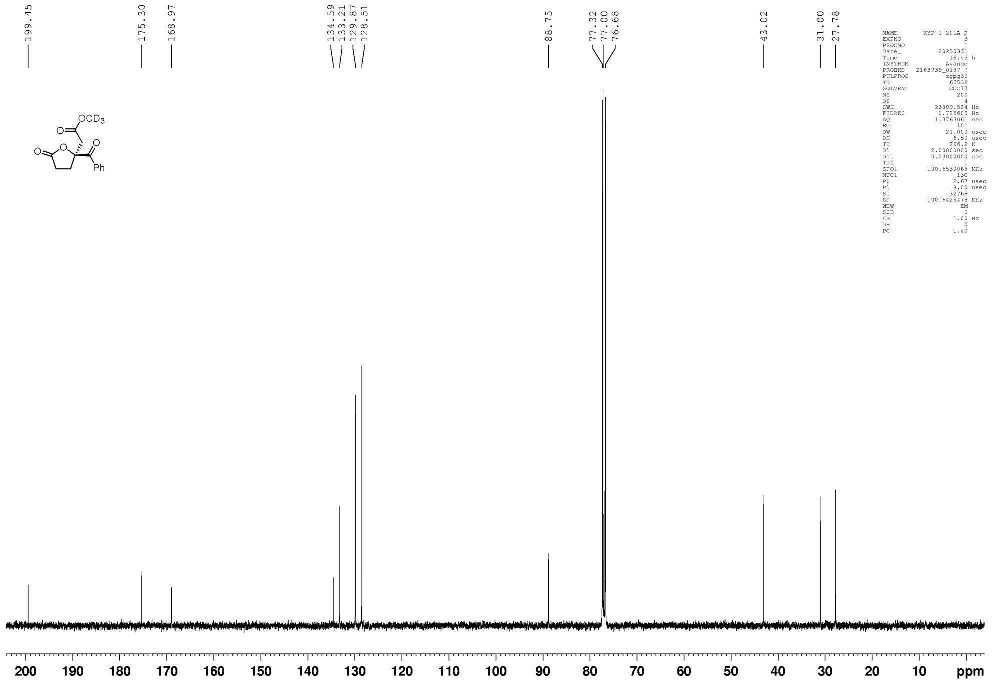

ethyl (S)-2-(2-benzoyl-5-oxotetrahydrofuran-2-yl)acetate (3w)

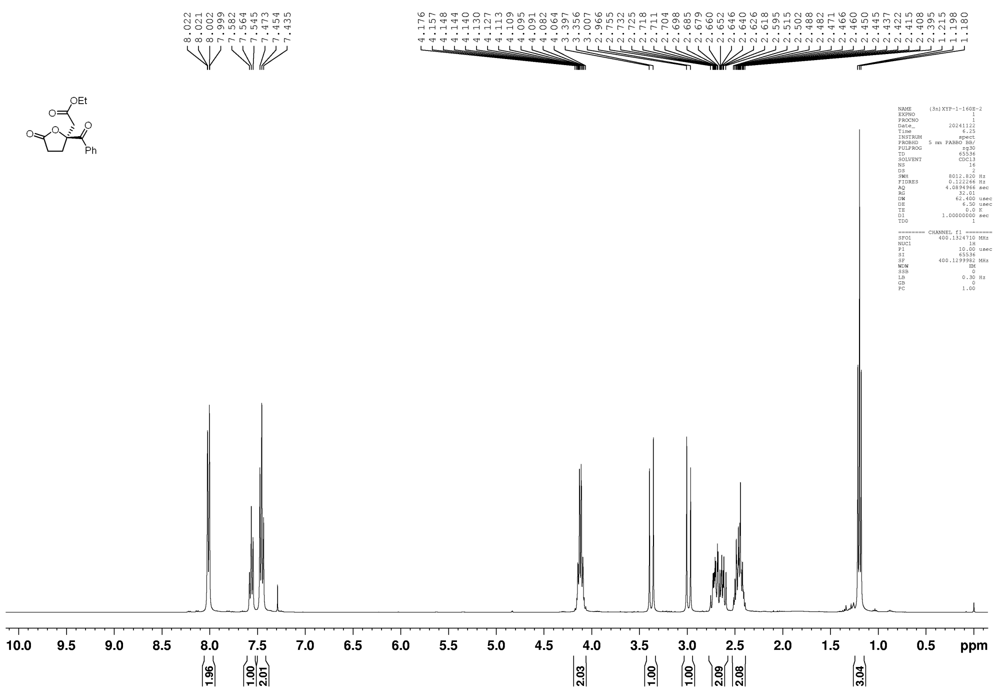

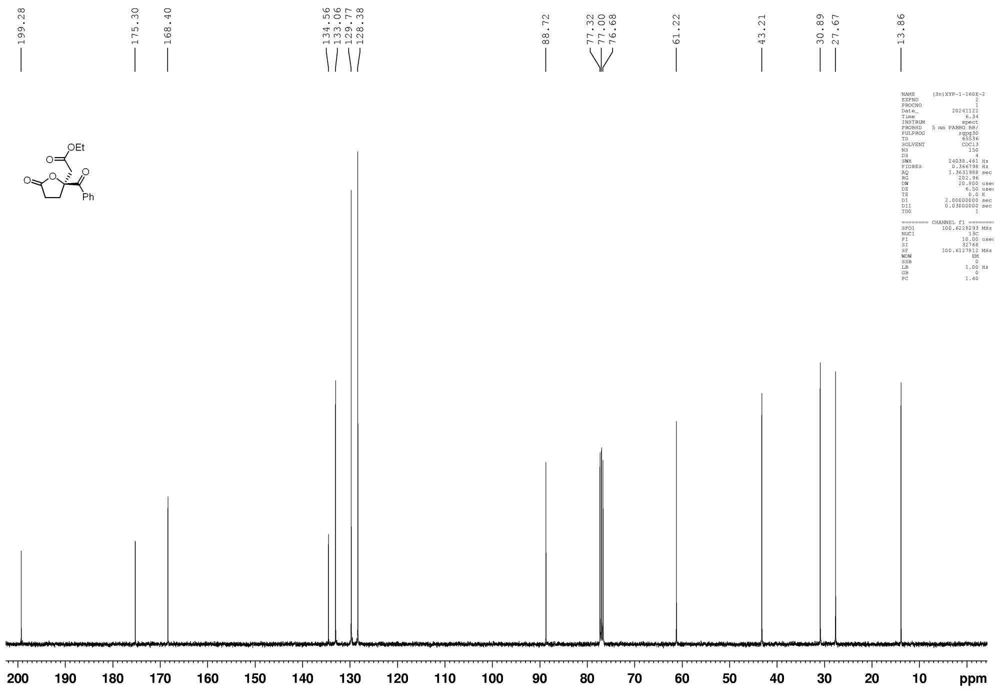

cyclopentylmethyl (S)-2-(2-benzoyl-5-oxotetrahydrofuran-2-yl)acetate (3x)

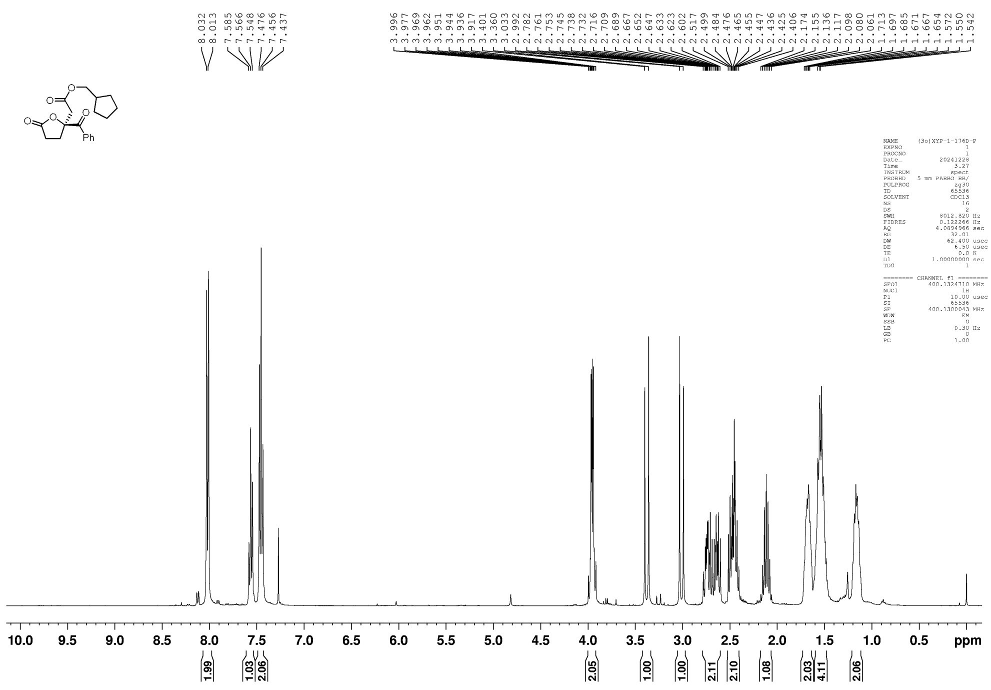

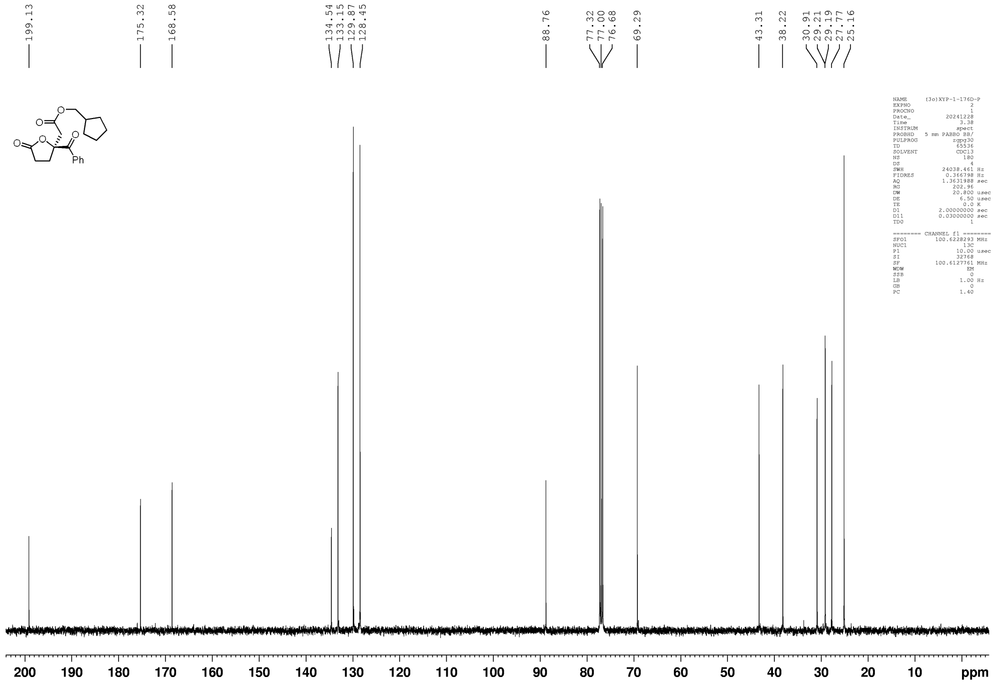

2-isopropoxyethyl (S)-2-(2-benzoyl-5-oxotetrahydrofuran-2-yl)acetate (3y)

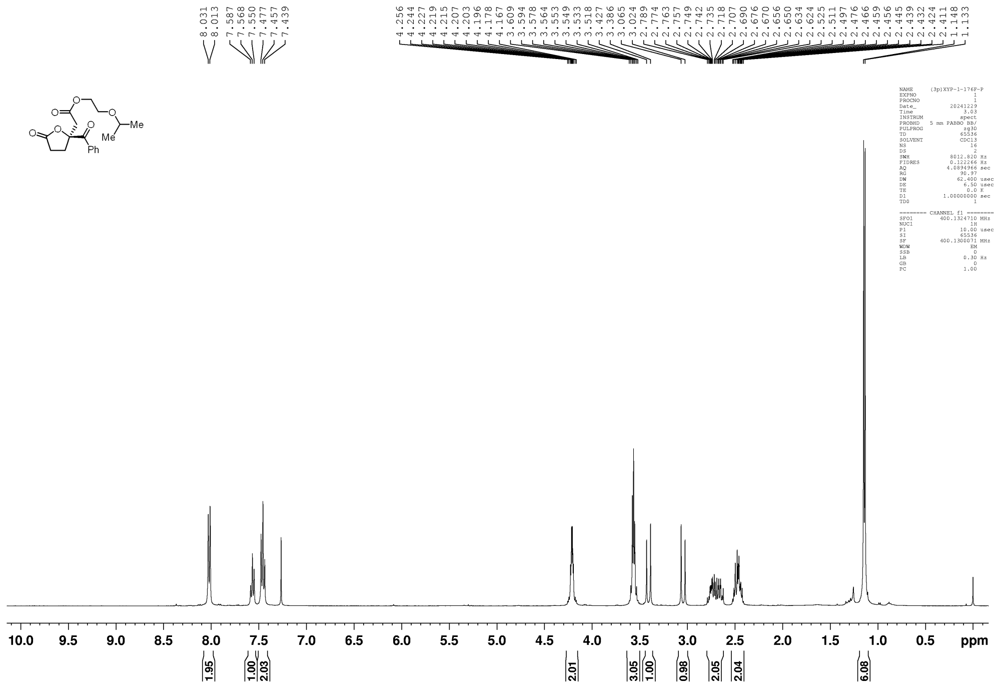

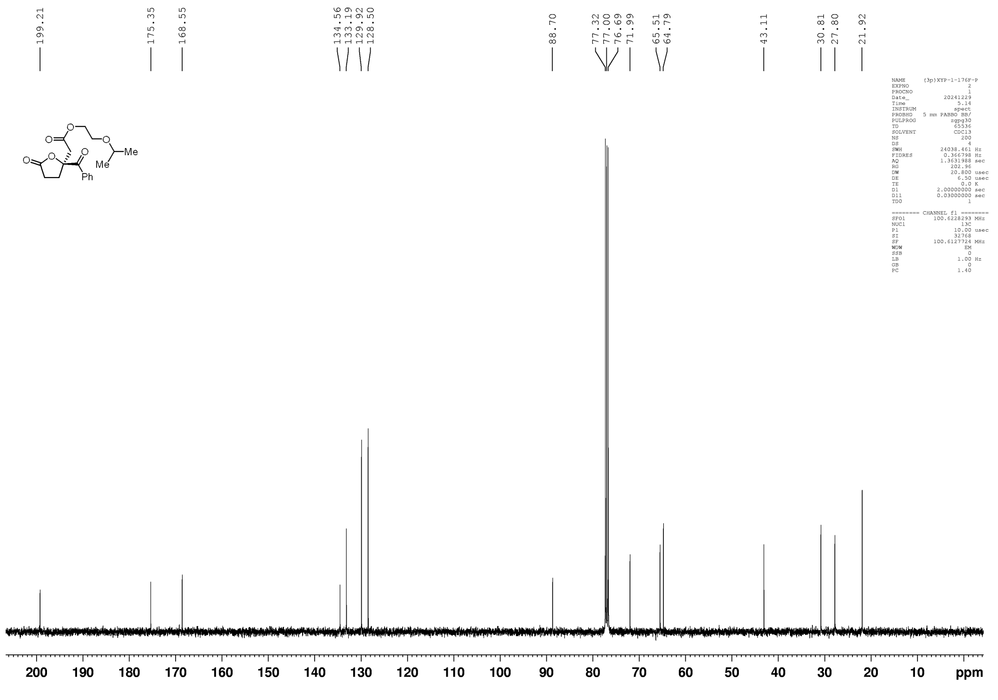

2-(trimethylsilyl)ethyl (S)-2-(2-benzoyl-5-oxotetrahydrofuran-2-yl)acetate (3z)

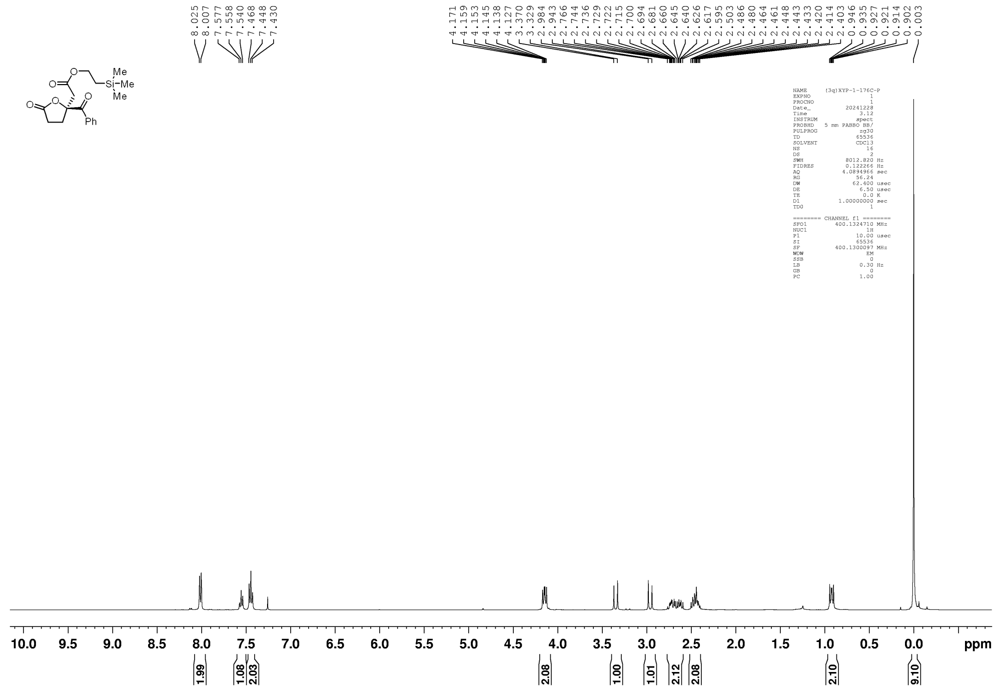

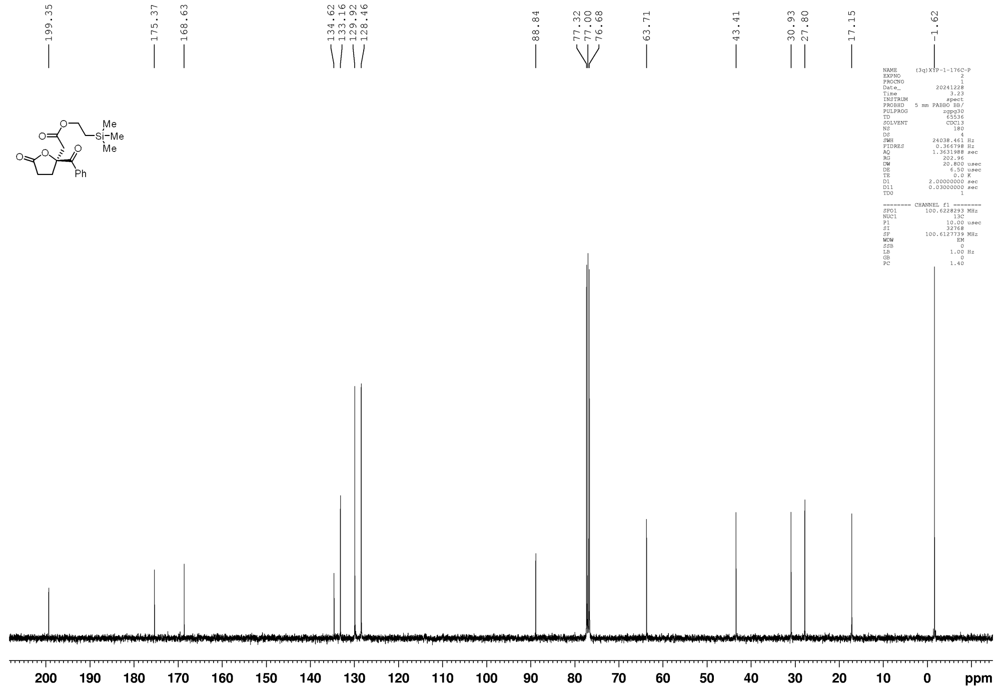

phenethyl (S)-2-(2-benzoyl-5-oxotetrahydrofuran-2-yl)acetate (3aa)

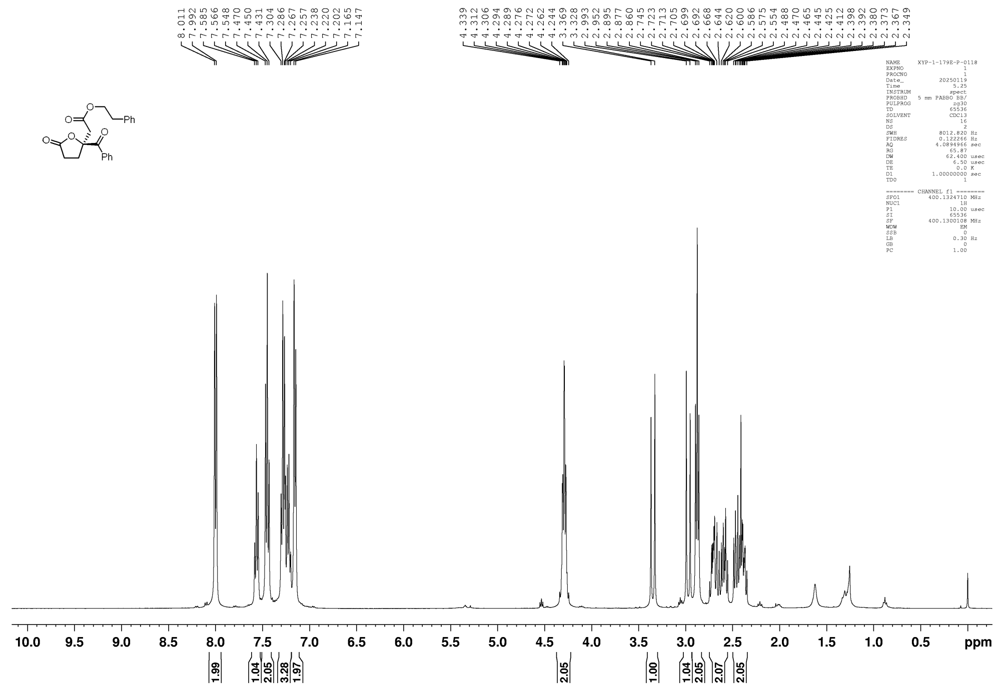

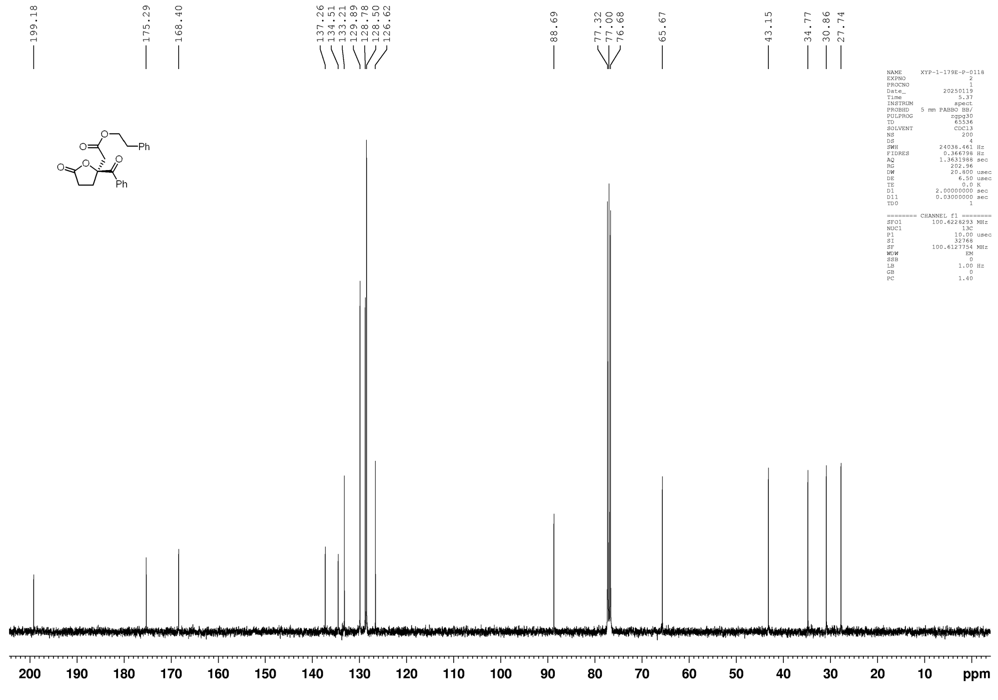

hex-5-en-1-yl (S)-2-(2-benzoyl-5-oxotetrahydrofuran-2-yl)acetate (3ab)

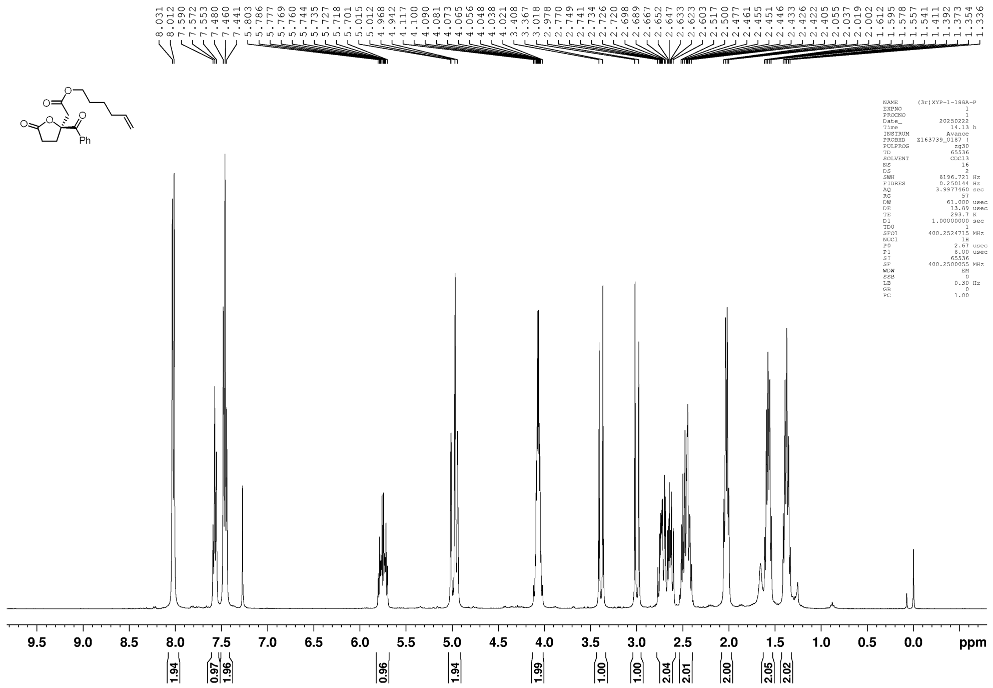

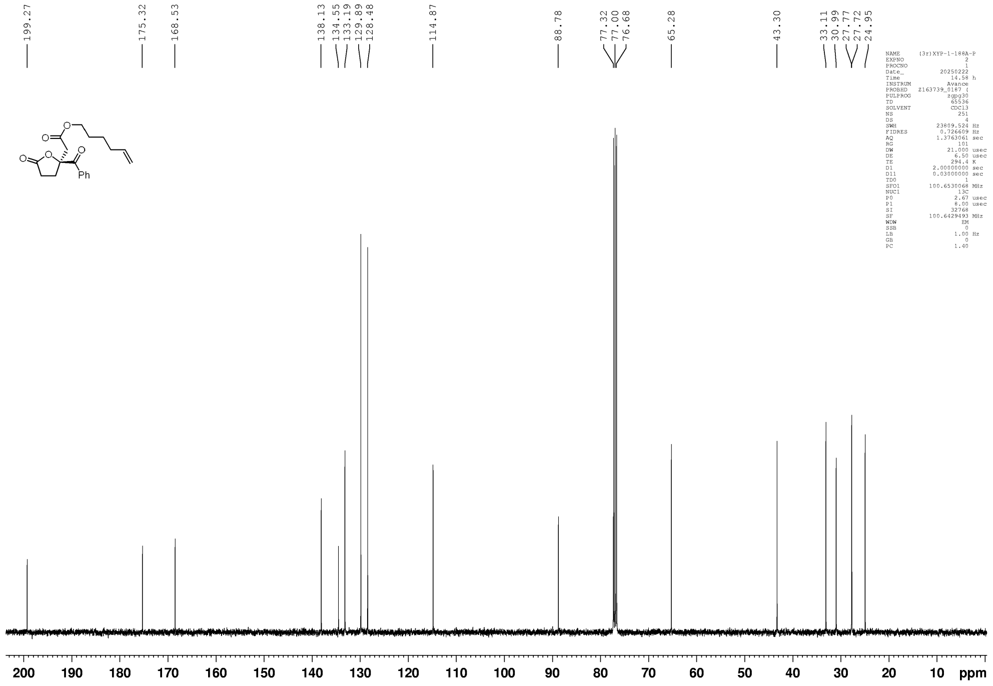

hept-6-yn-1-yl (S)-2-(2-benzoyl-5-oxotetrahydrofuran-2-yl)acetate (3ac)

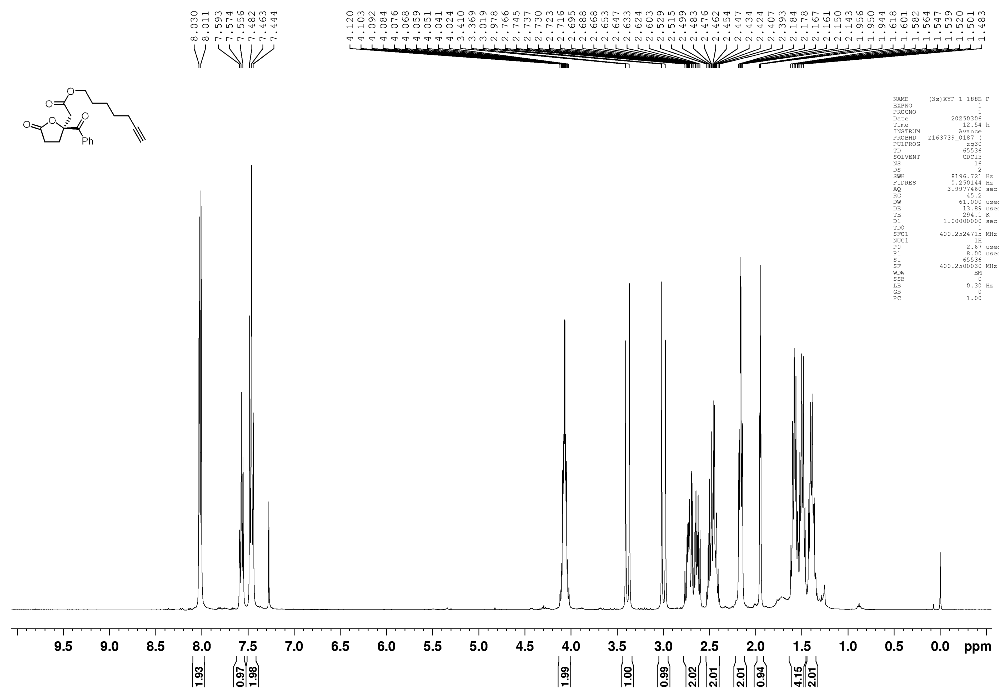

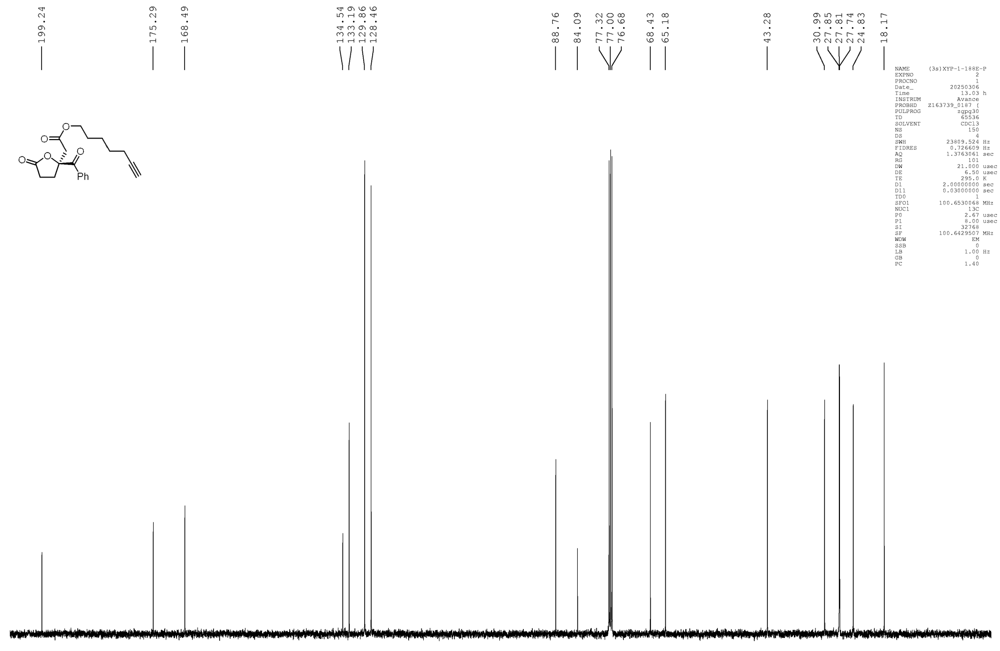

ethyl (S)-6-(2-(2-benzoyl-5-oxotetrahydrofuran-2-yl)acetoxy)hexanoate (3ad)

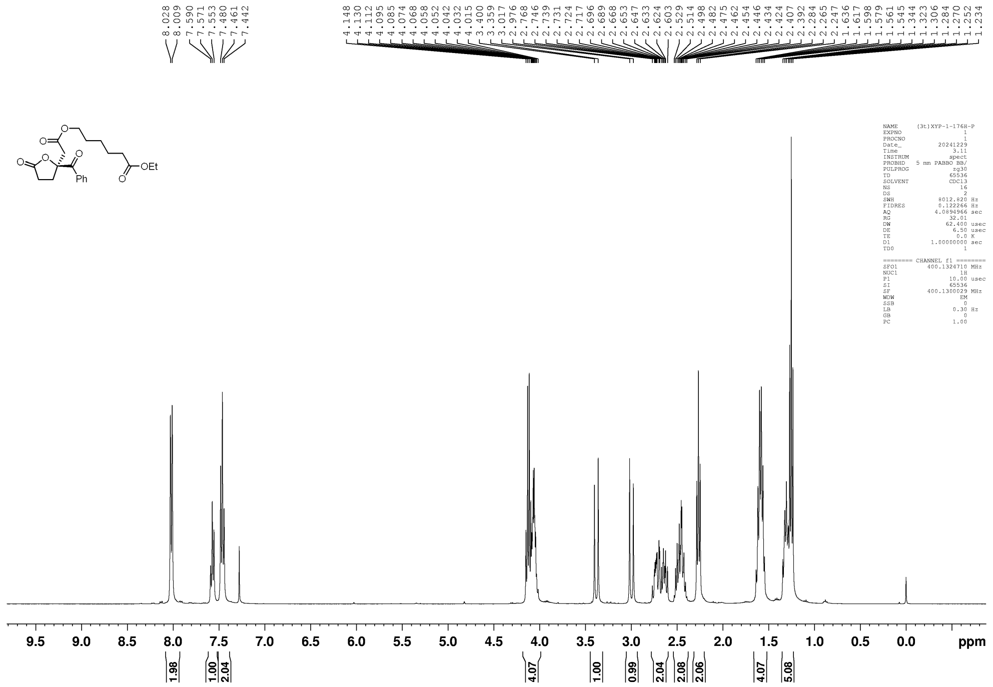

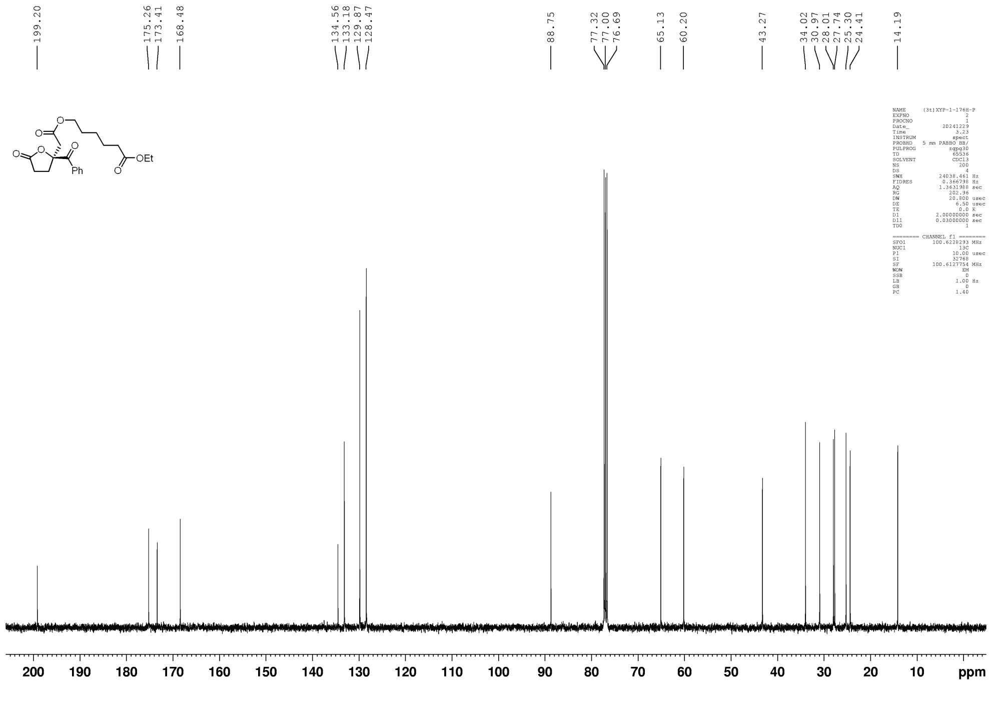

12-bromododecyl (S)-2-(2-benzoyl-5-oxotetrahydrofuran-2-yl)acetate (3ae)

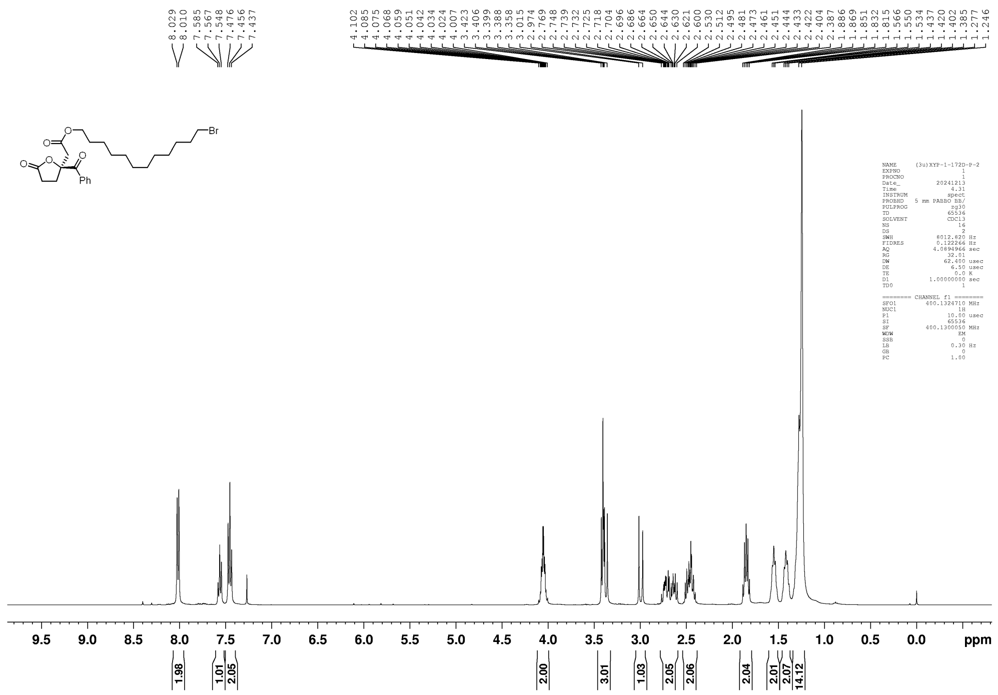

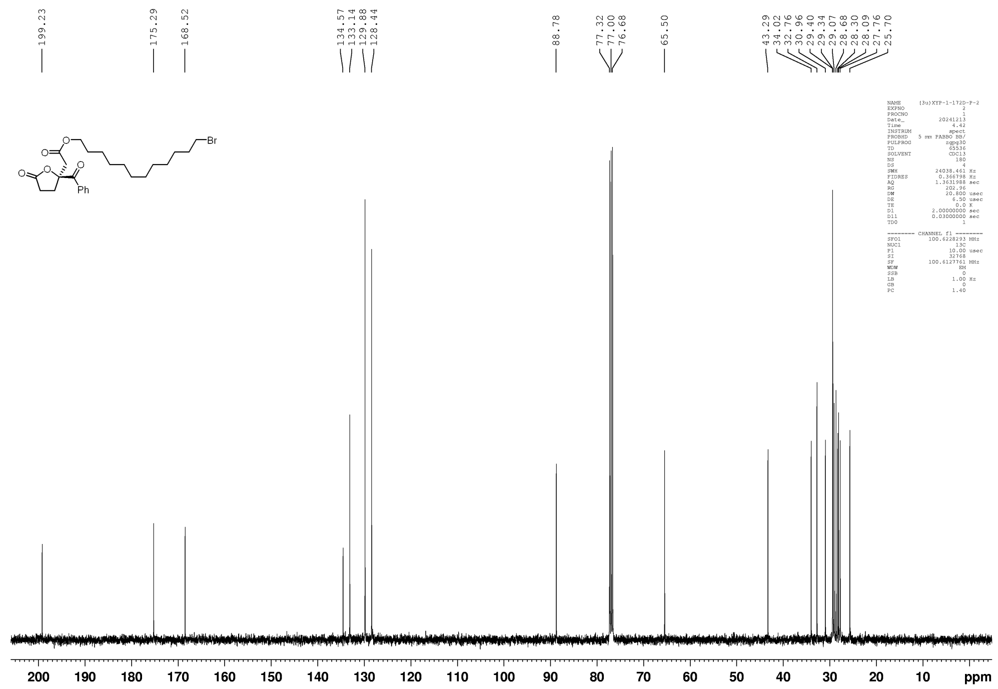

isopropyl (S)-2-(2-benzoyl-5-oxotetrahydrofuran-2-yl)acetate (3af)

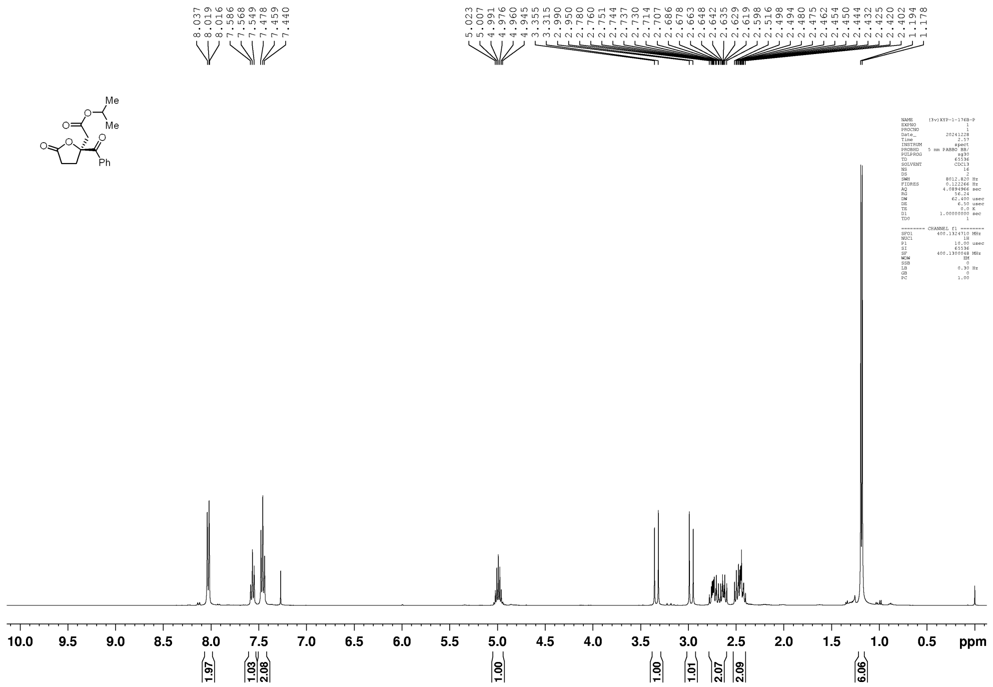

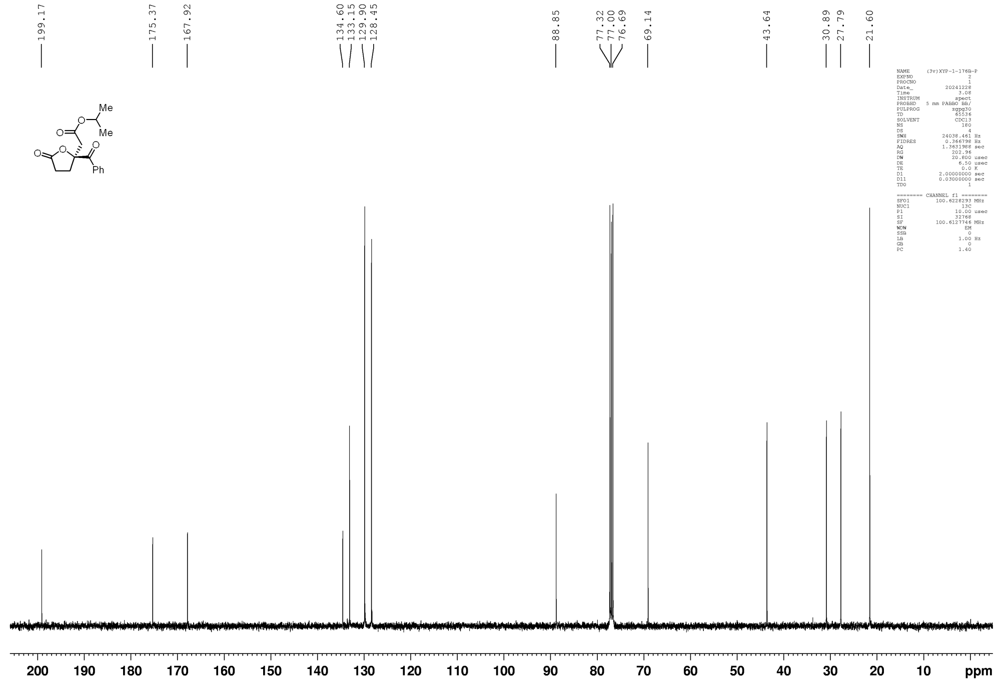

cyclopropyl (S)-2-(2-benzoyl-5-oxotetrahydrofuran-2-yl)acetate (3ag)

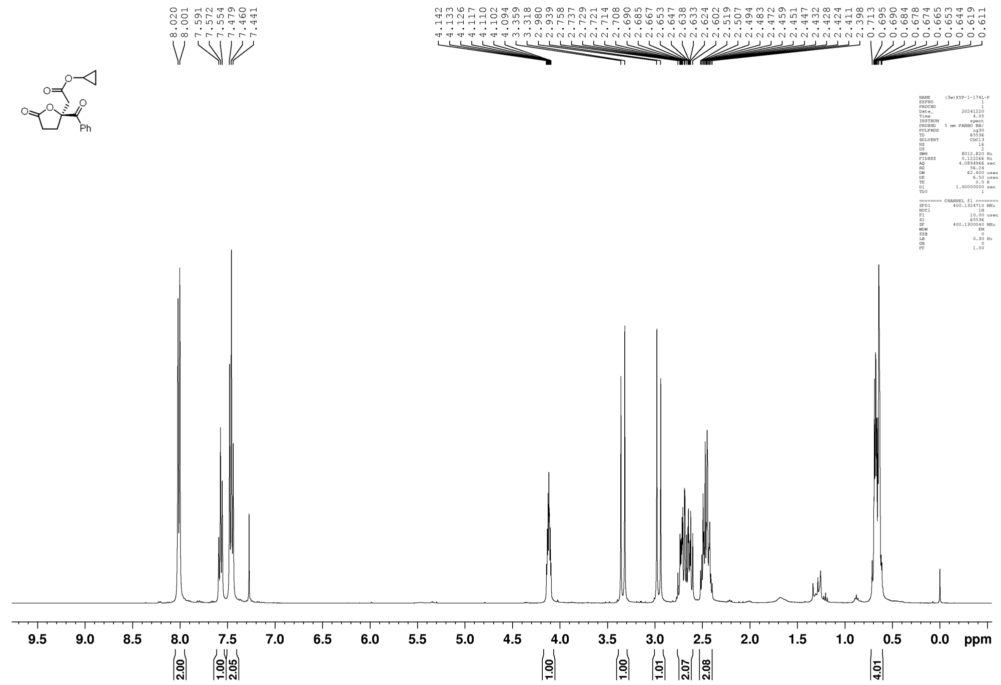

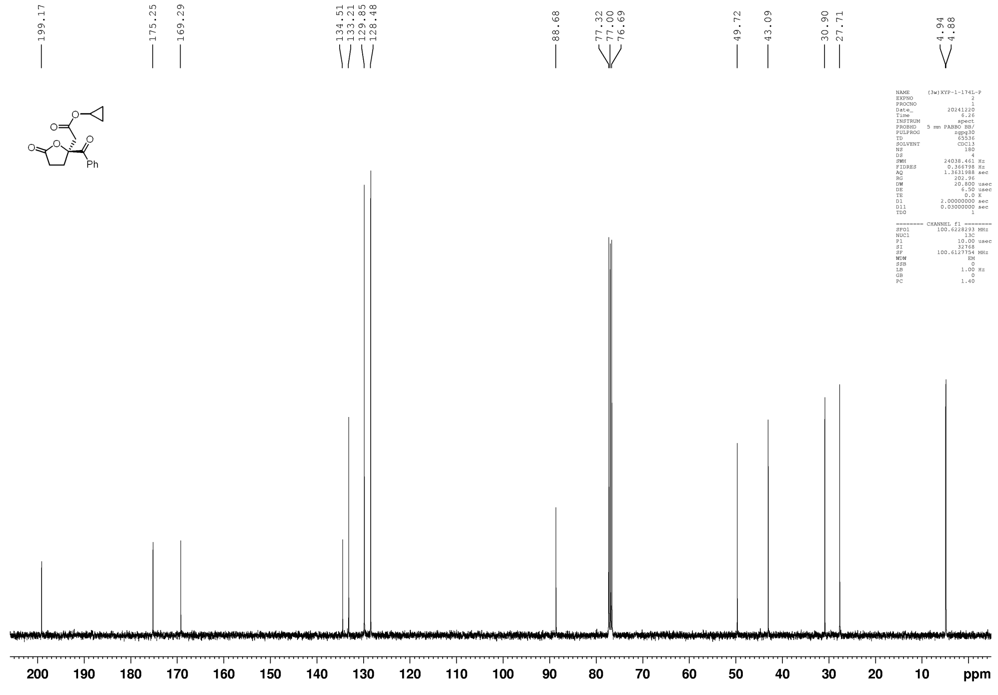

cyclobutyl (S)-2-(2-benzoyl-5-oxotetrahydrofuran-2-yl)acetate (3ah)

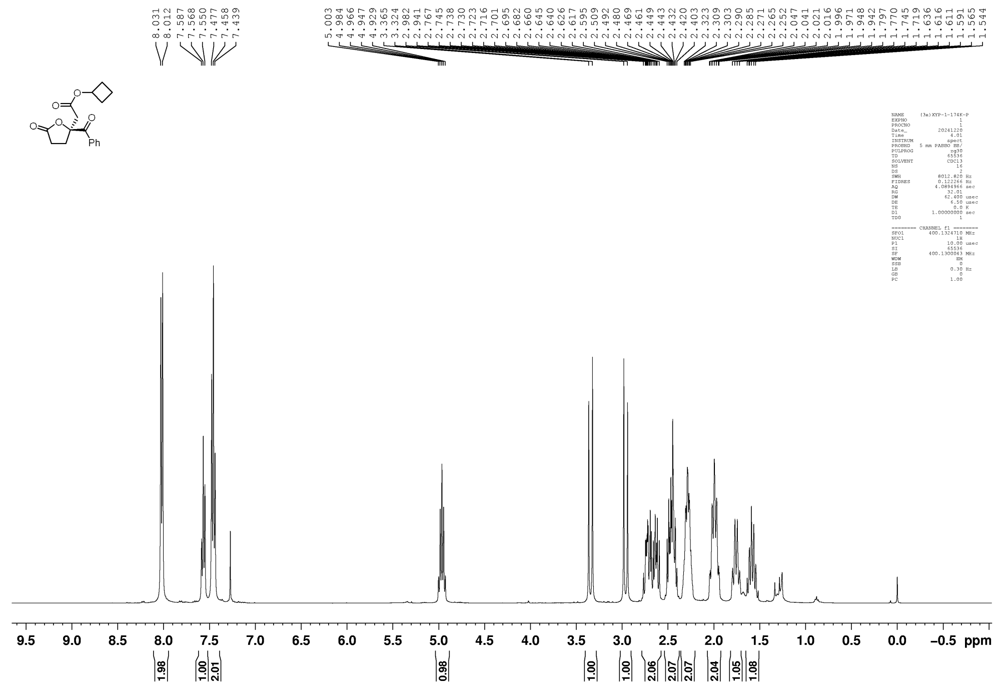

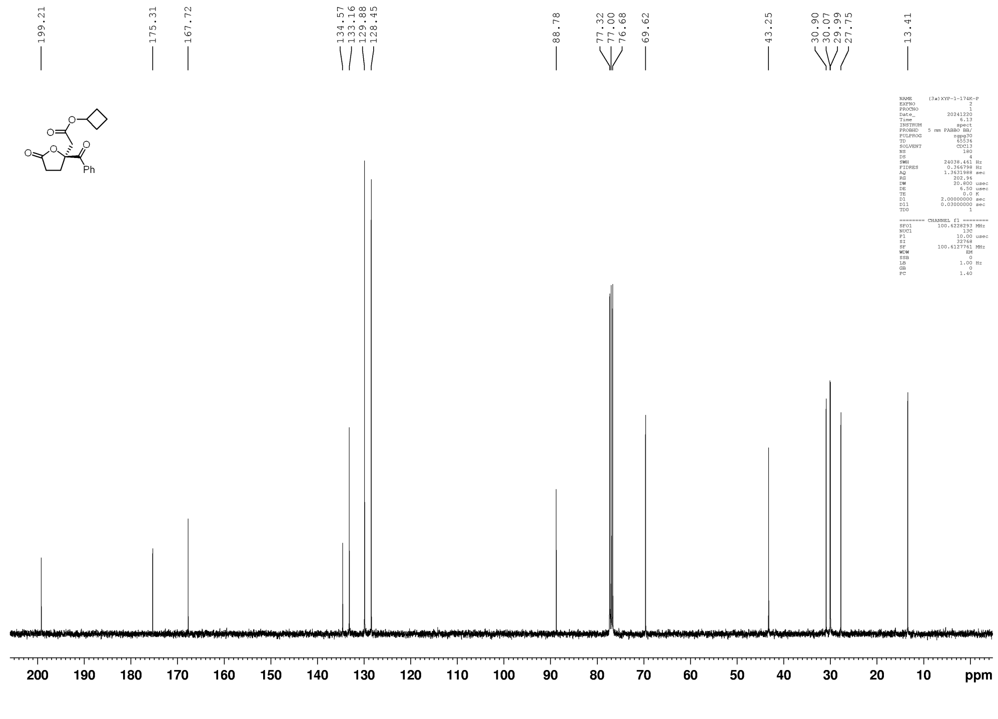

cyclopentyl (S)-2-(2-benzoyl-5-oxotetrahydrofuran-2-yl)acetate (3ai)

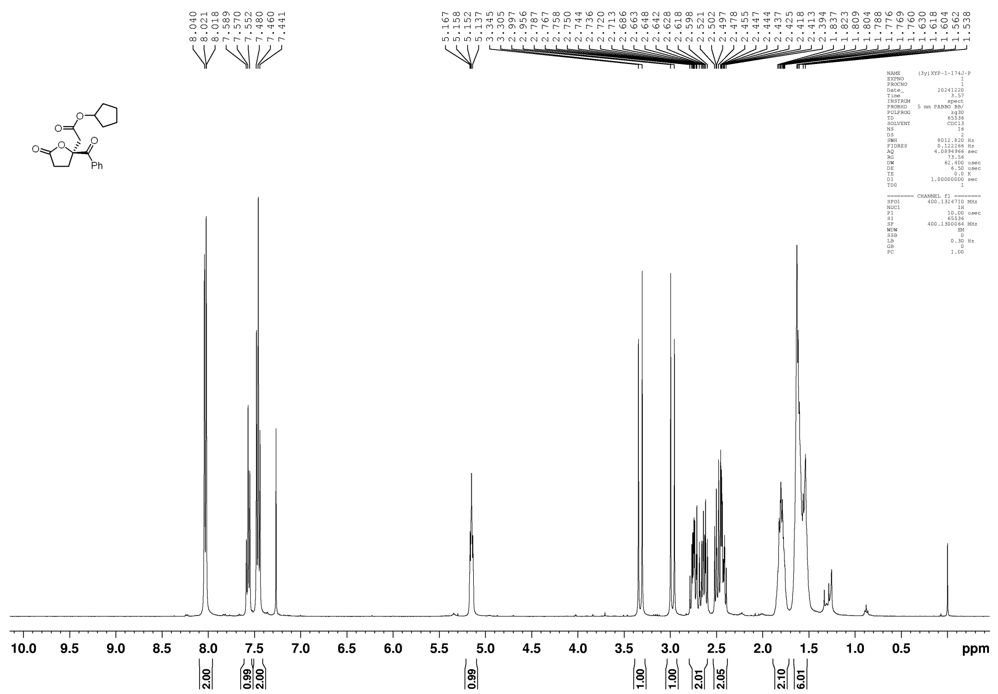

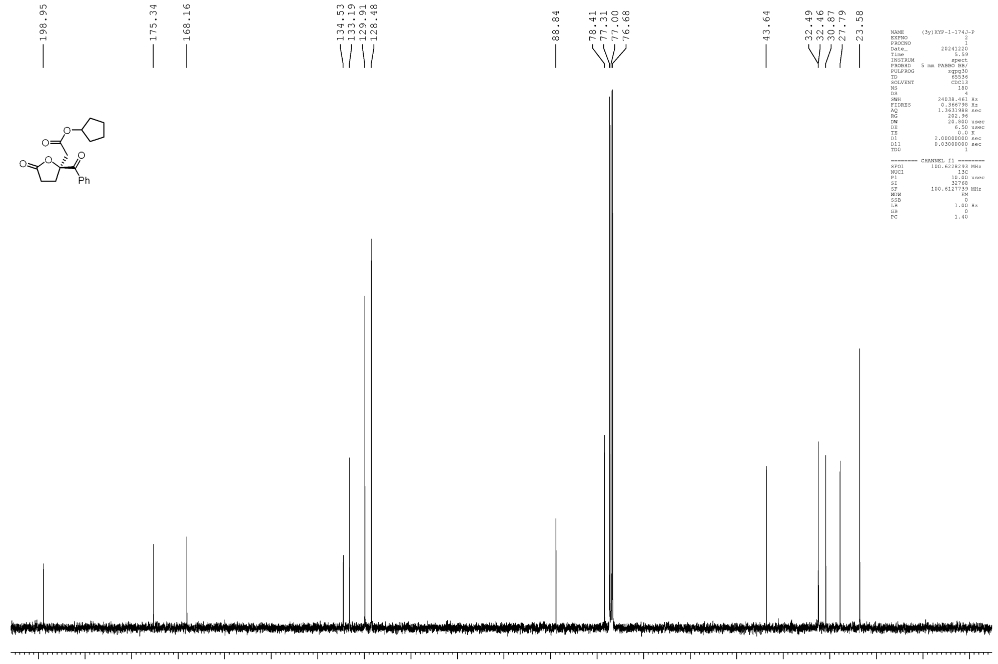

cyclohexyl (S)-2-(2-benzoyl-5-oxotetrahydrofuran-2-yl)acetate (3aj)

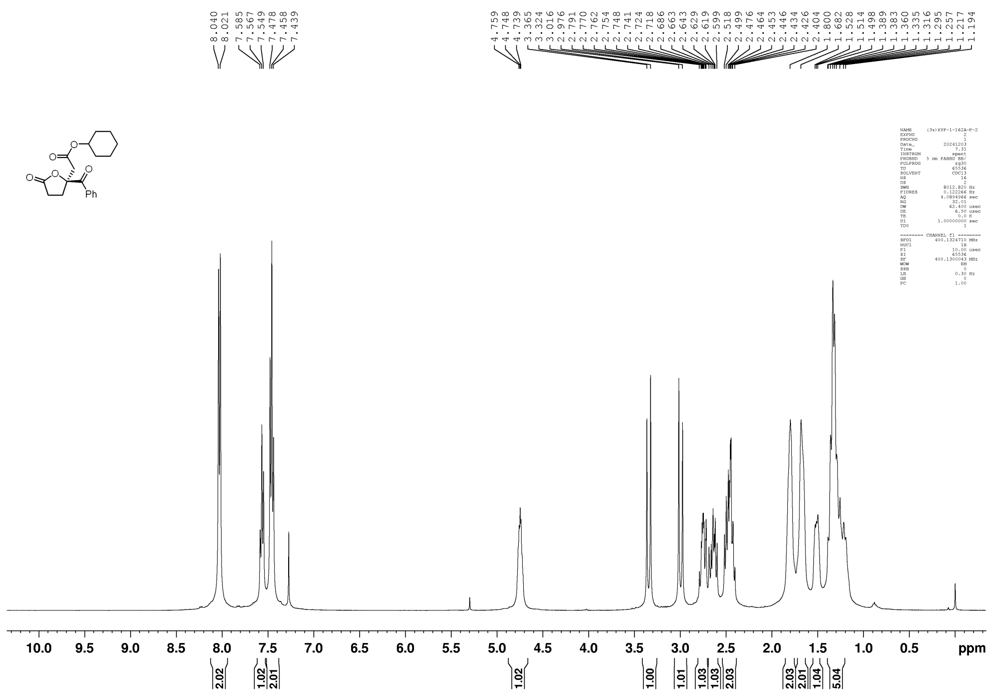

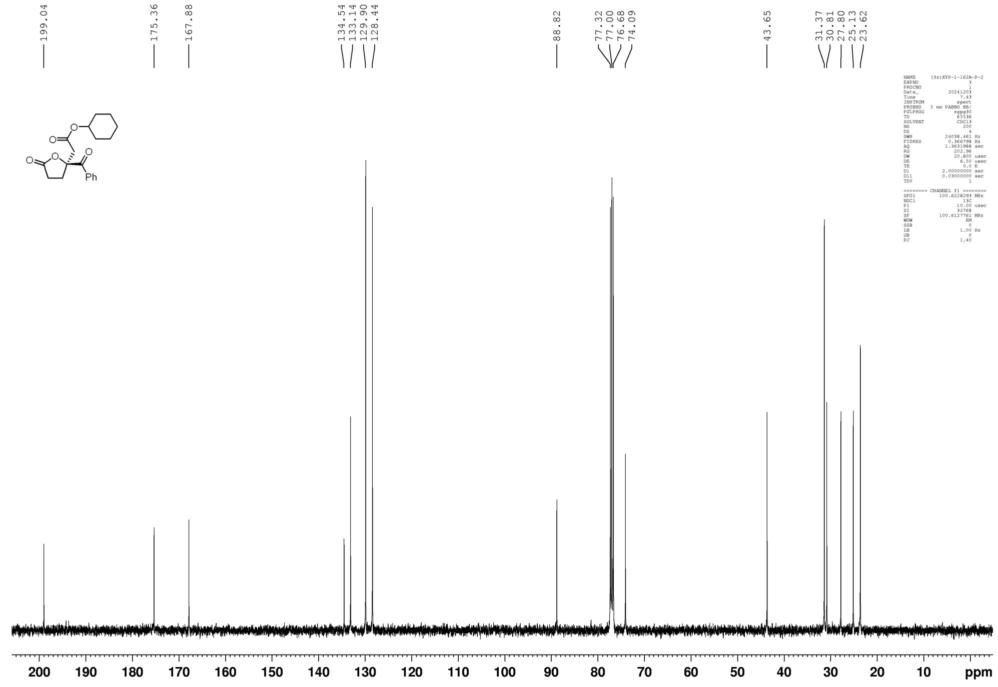

***tert*-butyl (S)-4-(2-(2-benzoyl-5-oxotetrahydrofuran-2-yl)acetoxy)piperidine-1-carboxylate (3ak)**

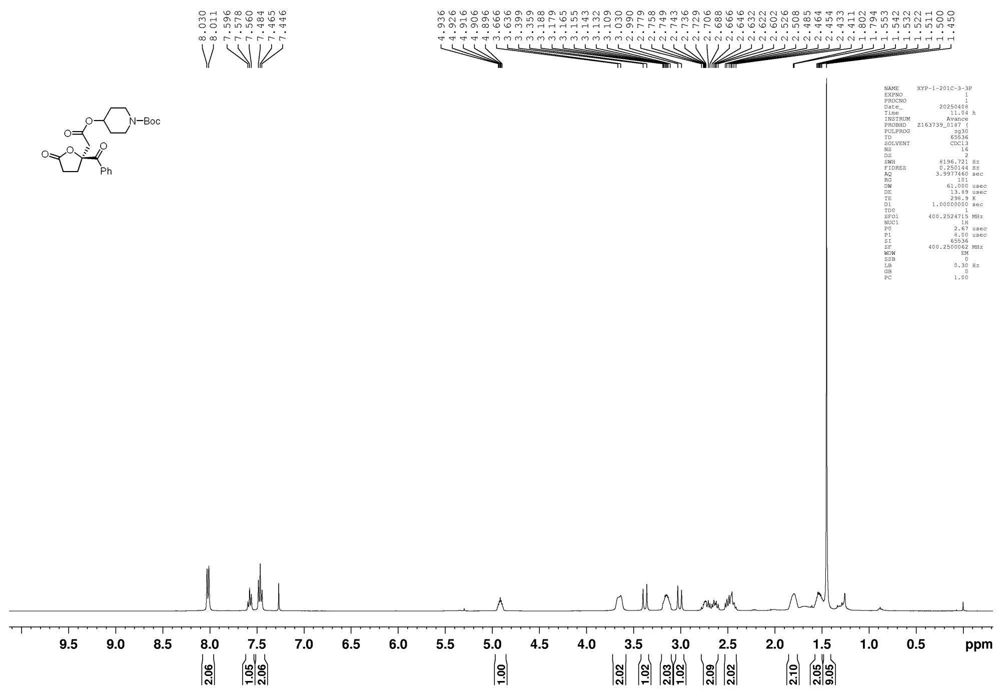

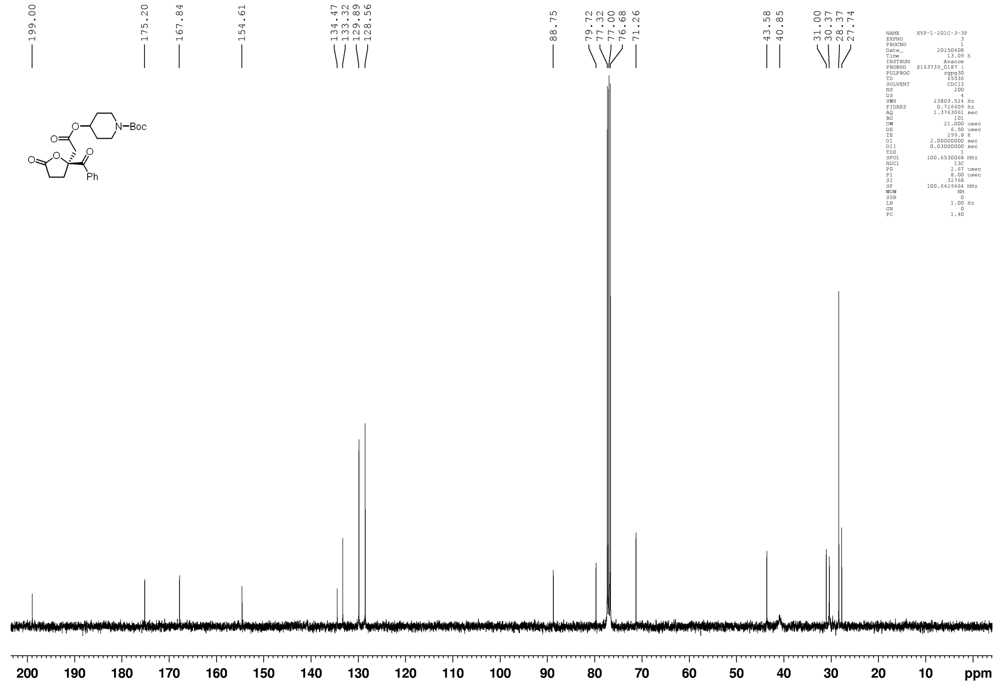

cyclododecyl (S)-2-(2-benzoyl-5-oxotetrahydrofuran-2-yl)acetate (3al)

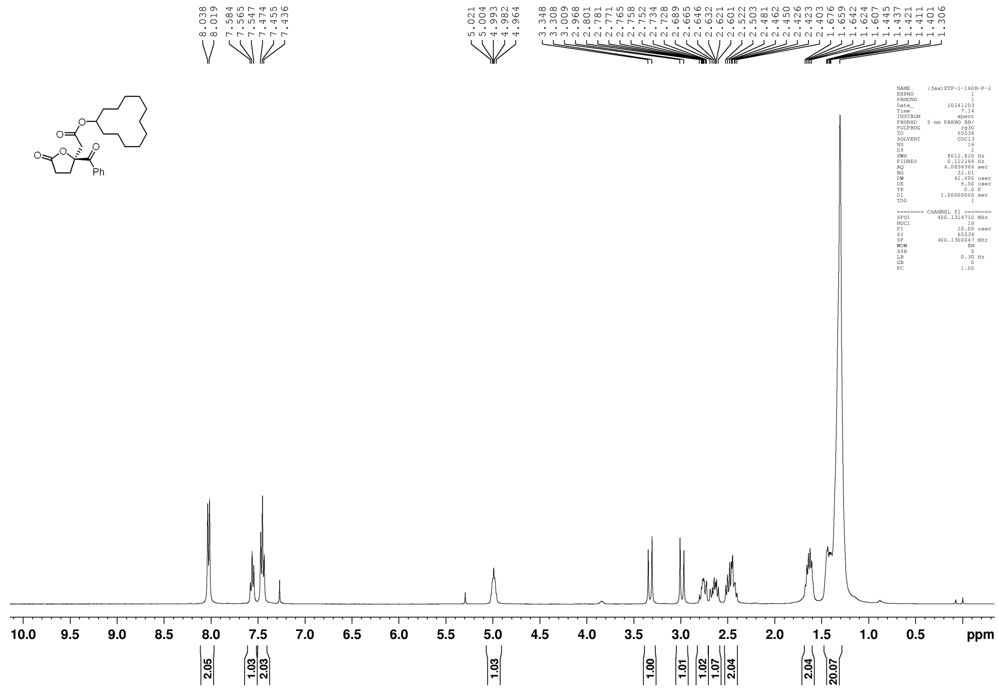

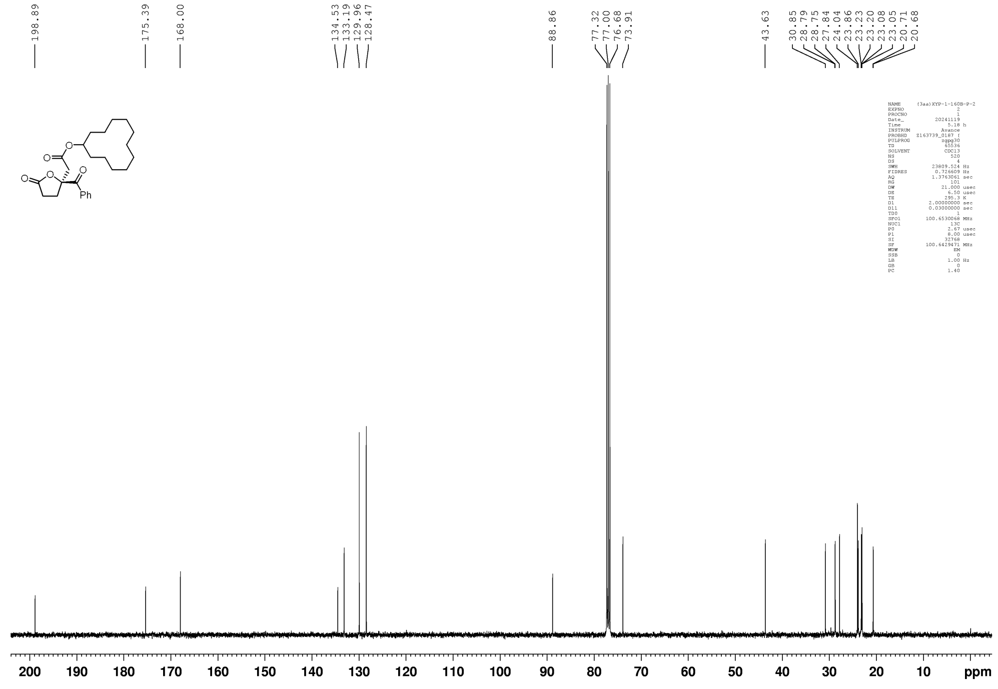

(1S,3S,5S,7S)-adamantan-2-yl 2-((S)-2-benzoyl-5-oxotetrahydrofuran-2-yl)acetate (3am)

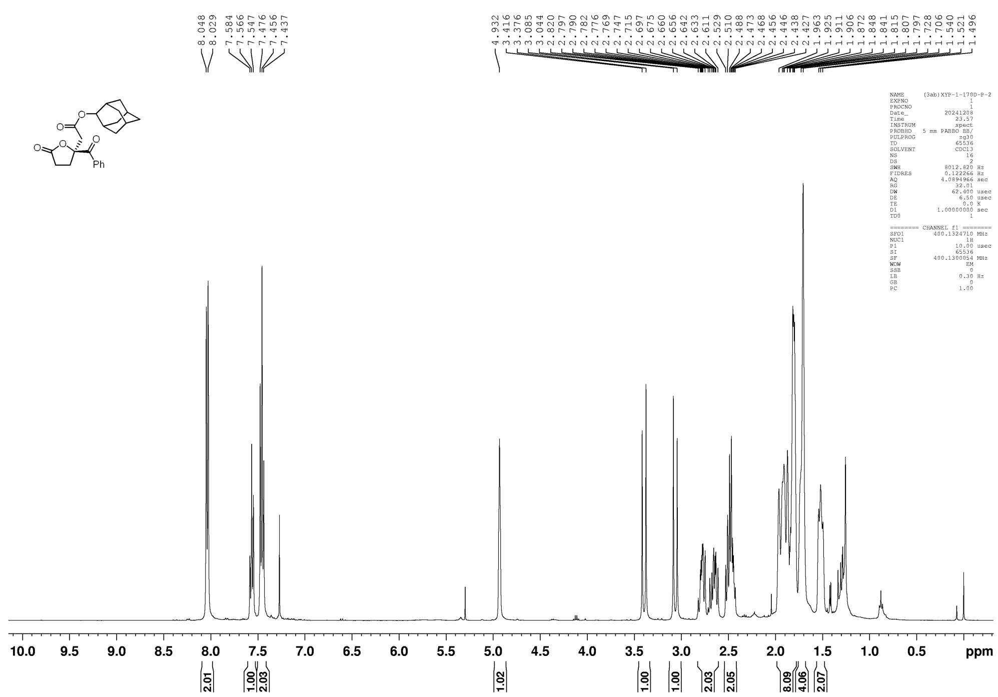

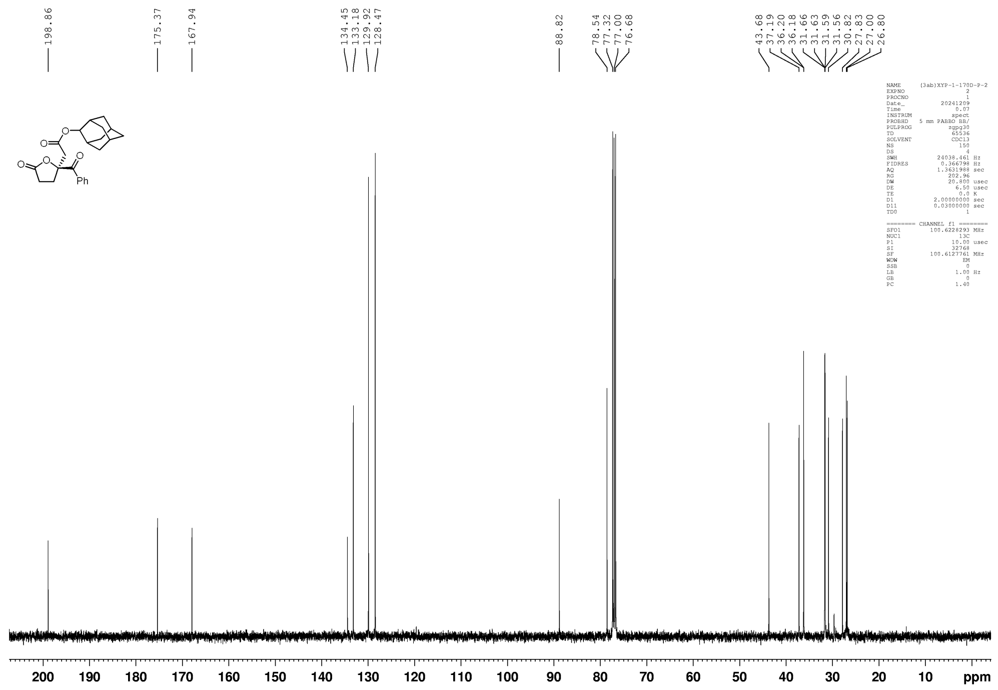

(3R,5R,7R)-adamantan-1-yl 2-((S)-2-benzoyl-5-oxotetrahydrofuran-2-yl)acetate (3an)

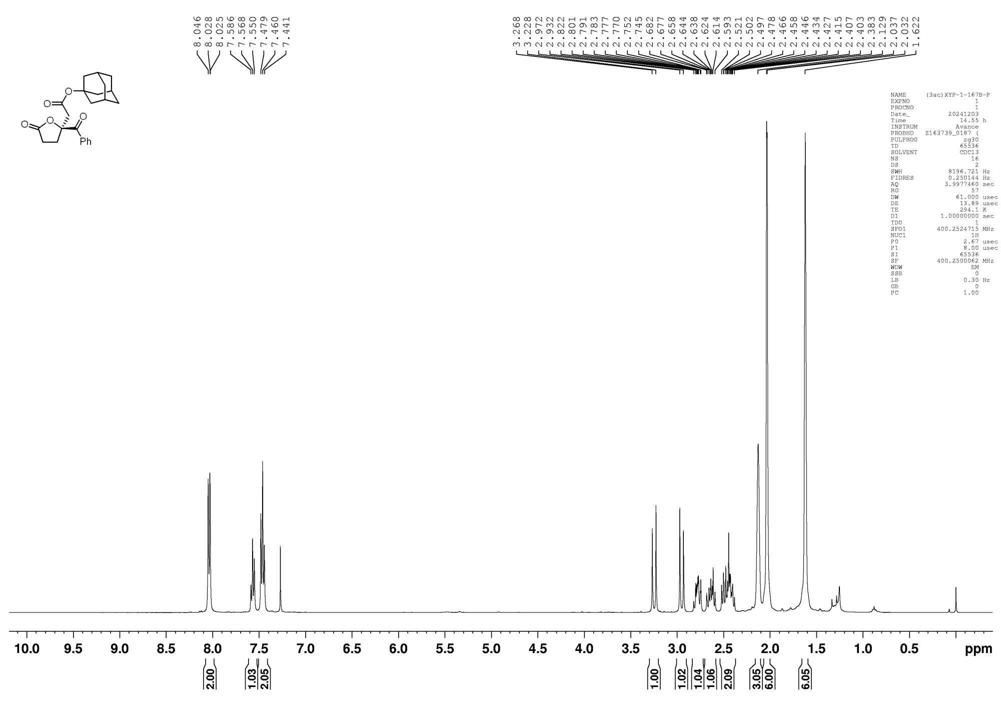

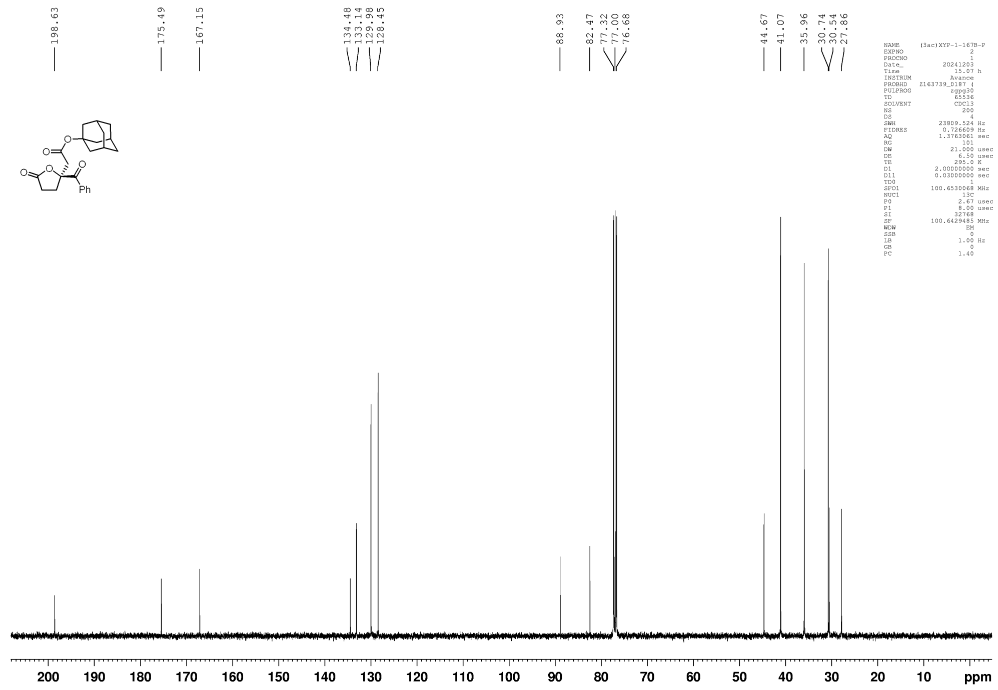

(S)-5-(((3S,5S,7S)-adamantan-1-yl)methyl)-5-benzoyldihydrofuran-2(3H)-one (3an')

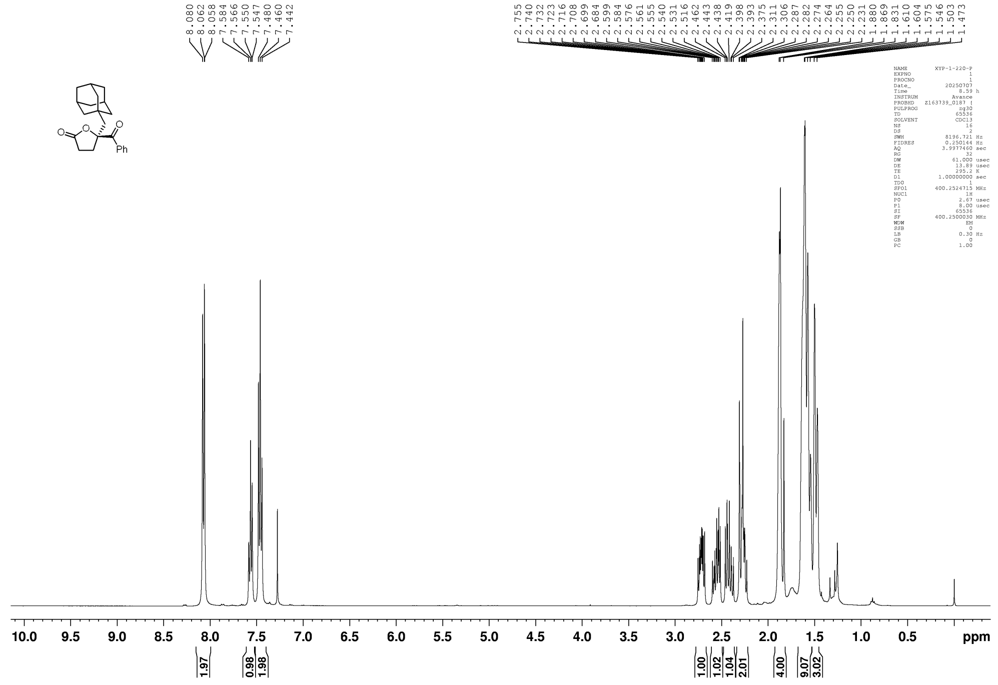

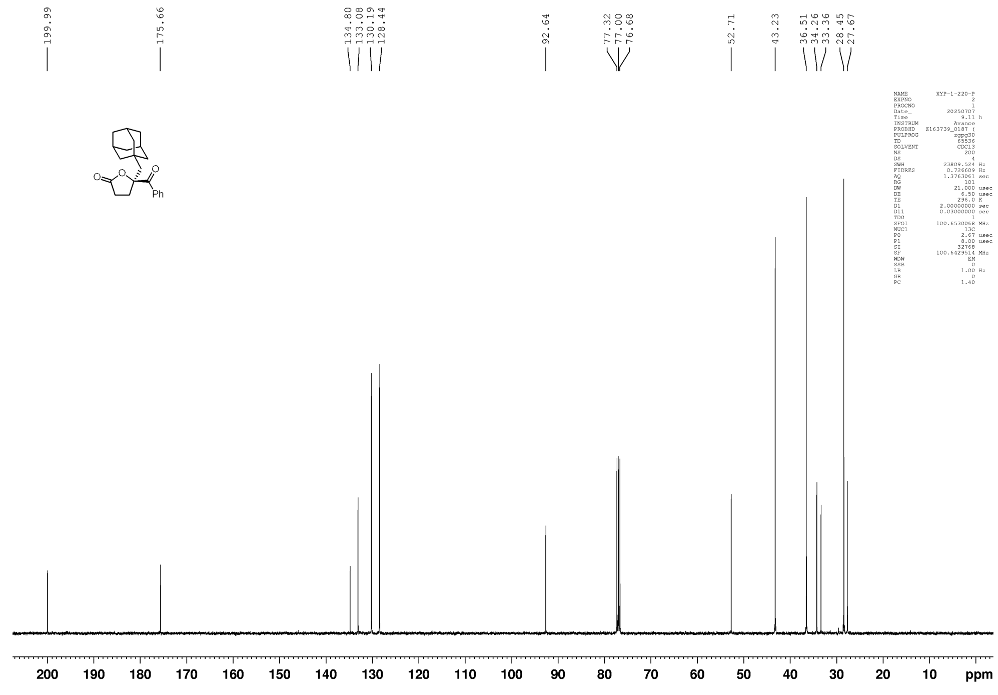

(1S,1's,4R,4'S)-4'-propyl-[1,1'-bi(cyclohexan)]-4-yl 2-((S)-2-benzoyl-5-oxotetrahydrofuran-2-yl)acetate (3ao)

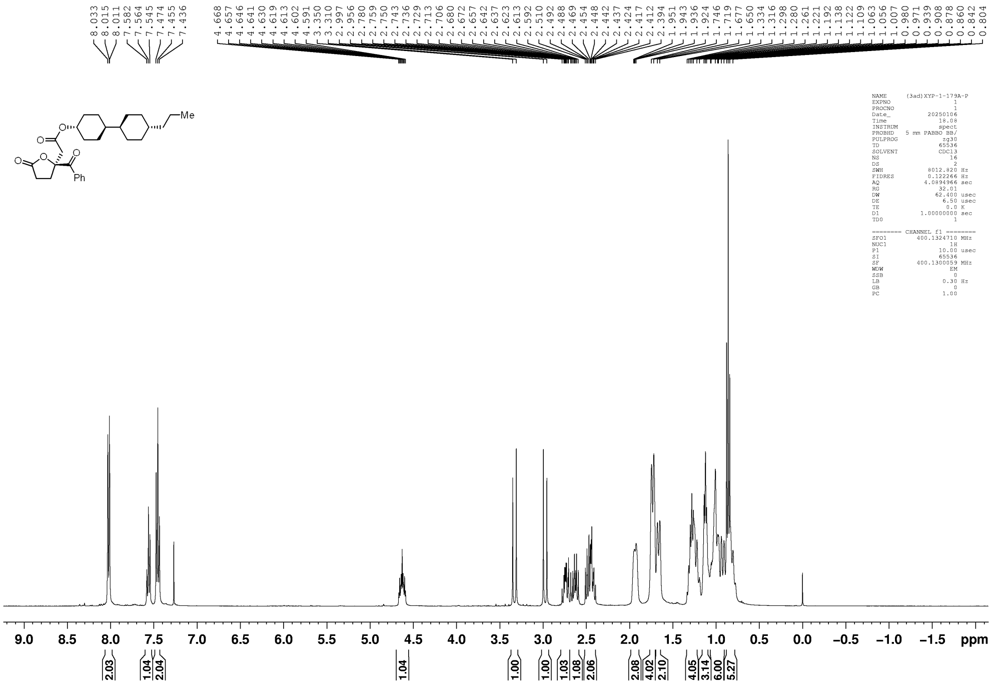

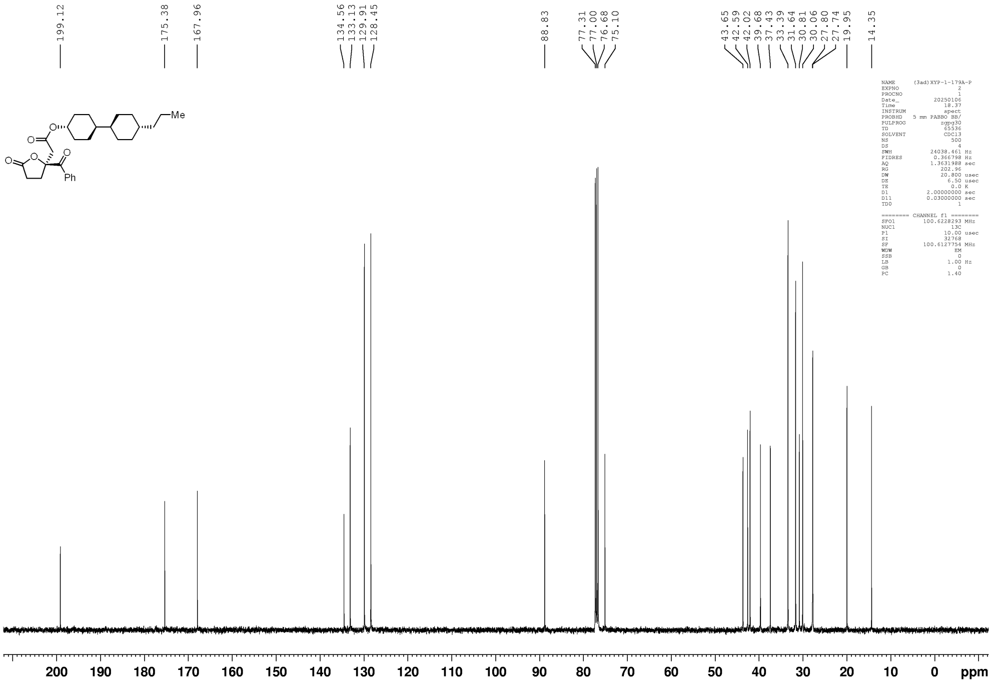

hexadecyl (S)-2-(2-benzoyl-5-oxotetrahydrofuran-2-yl)acetate (3ap)

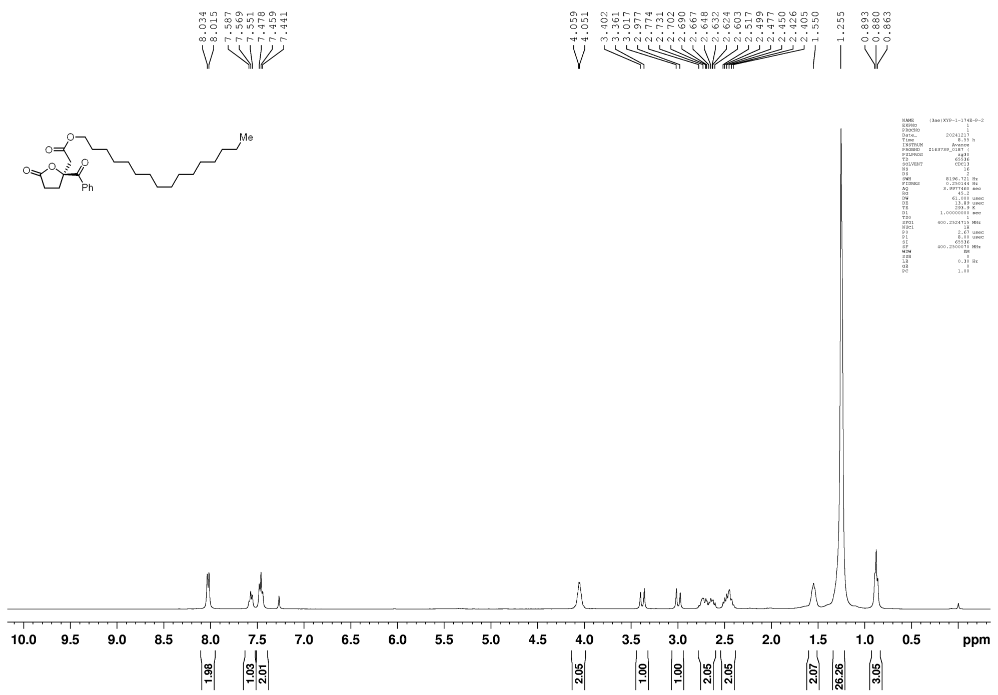

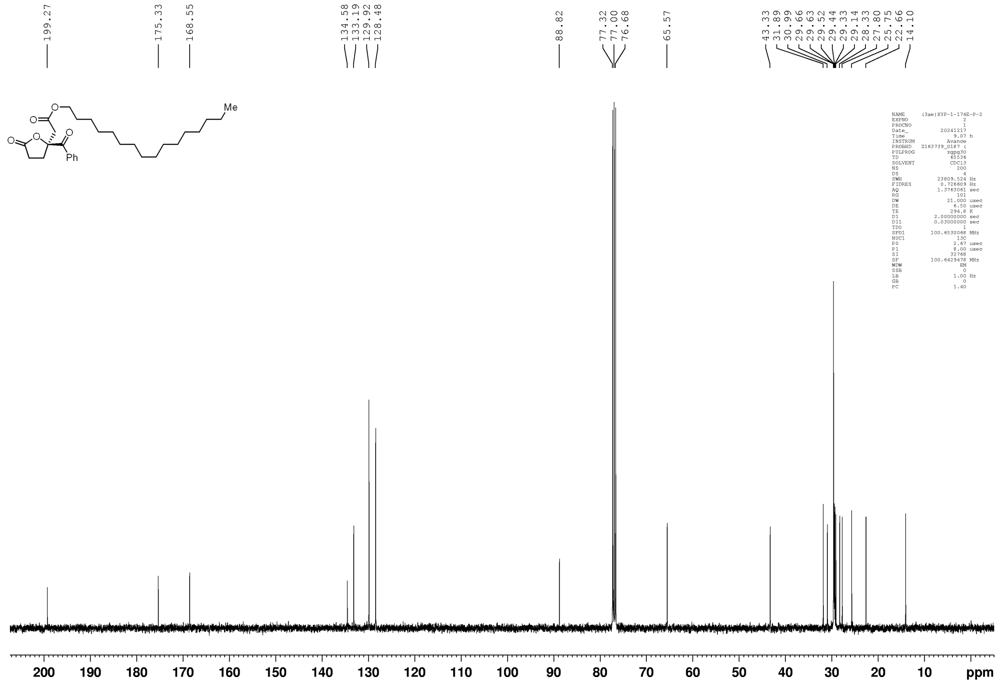

(S)-3,7-dimethyloct-6-en-1-yl 2-((S)-2-benzoyl-5-oxotetrahydrofuran-2-yl)acetate (3aq)

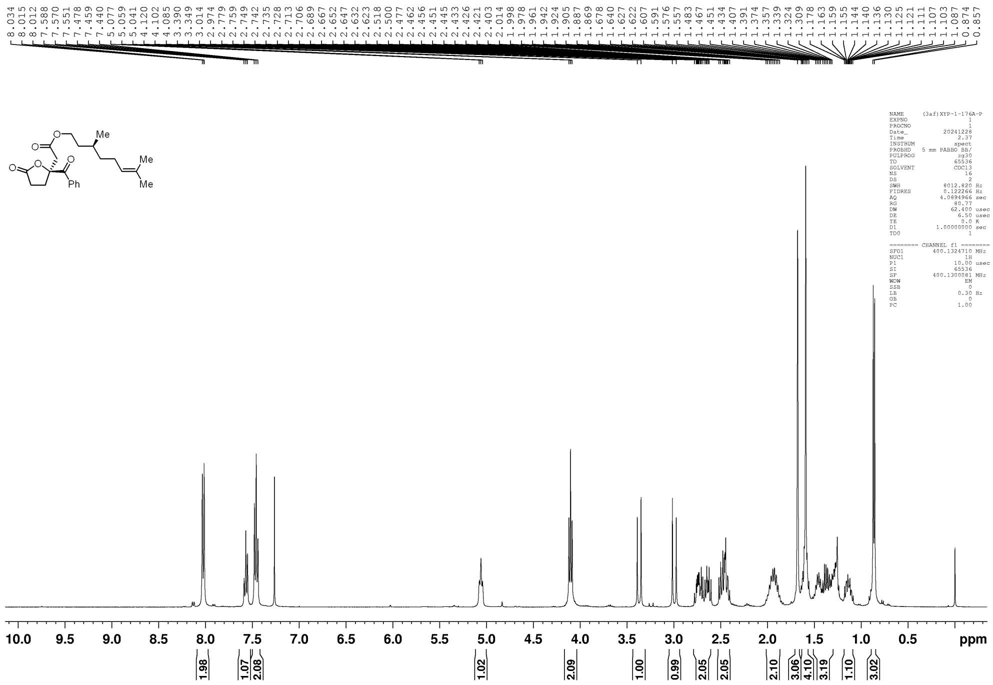

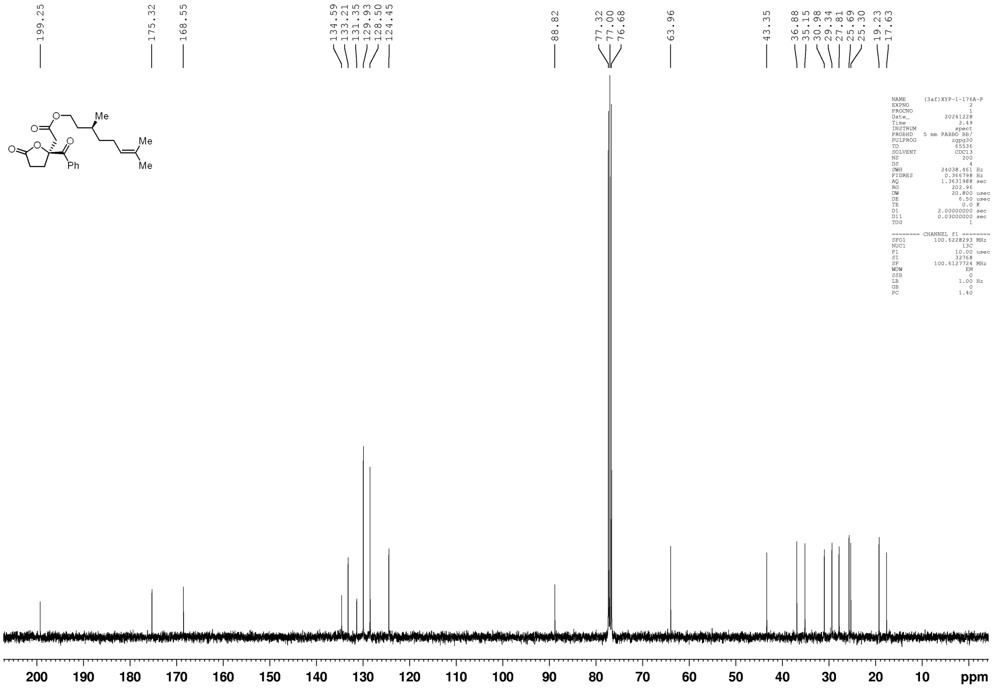

(2S,4R)-1,7,7-trimethylbicyclo[2.2.1]heptan-2-yl 2-((S)-2-benzoyl-5-oxotetrahydrofuran-2-yl)acetate (3ar)

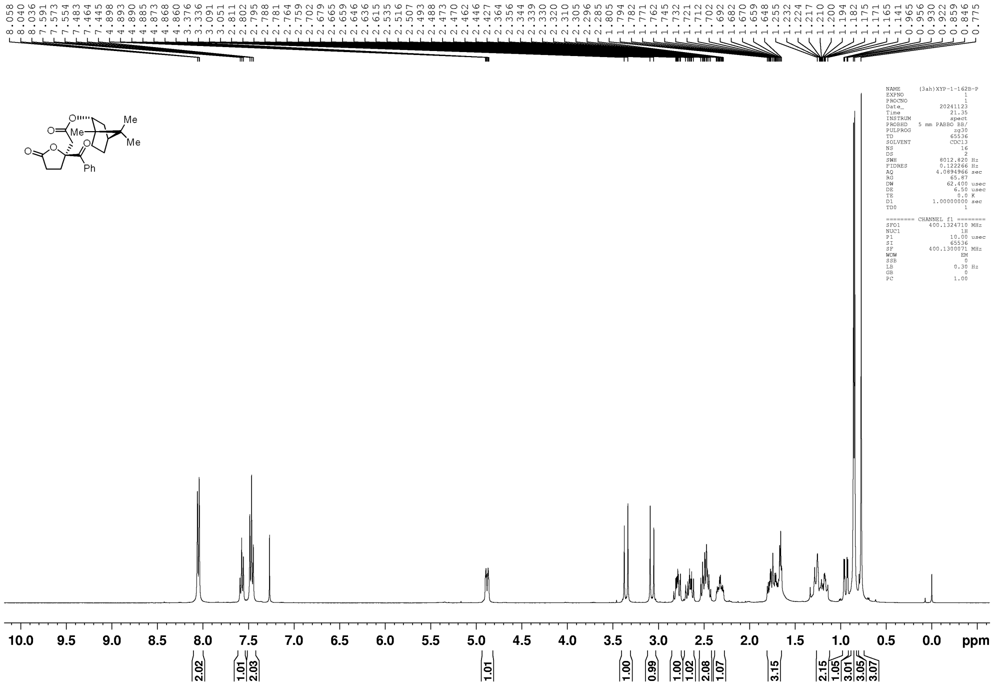

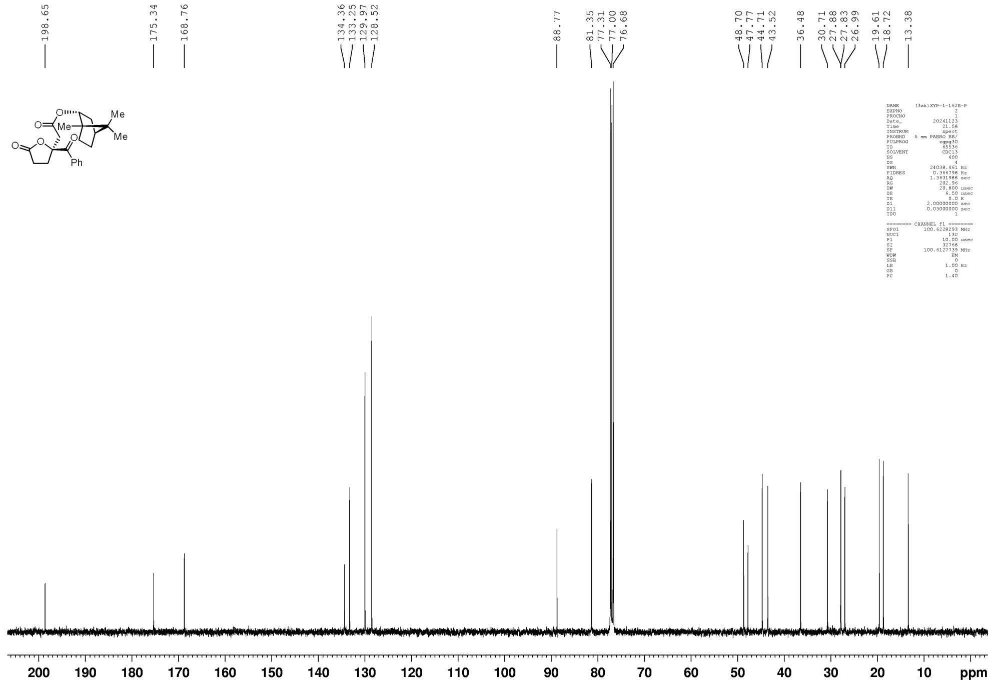

(1R,2R,4S)-1,3,3-trimethylbicyclo[2.2.1]heptan-2-yl 2-((S)-2-benzoyl-5-oxotetrahydrofuran-2-yl)acetate (3as)

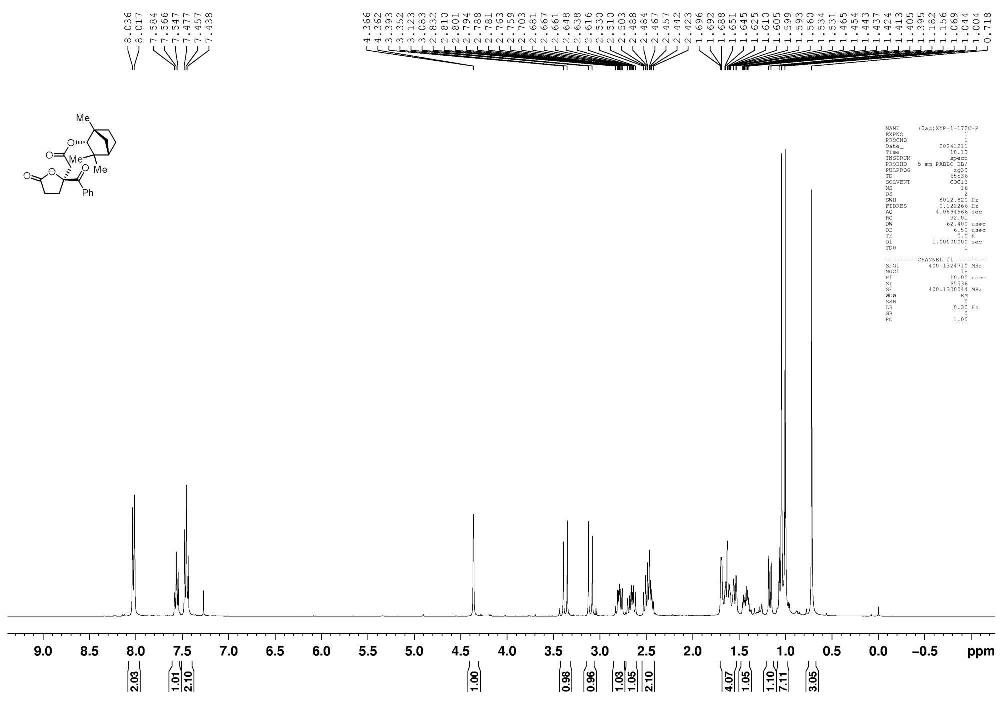

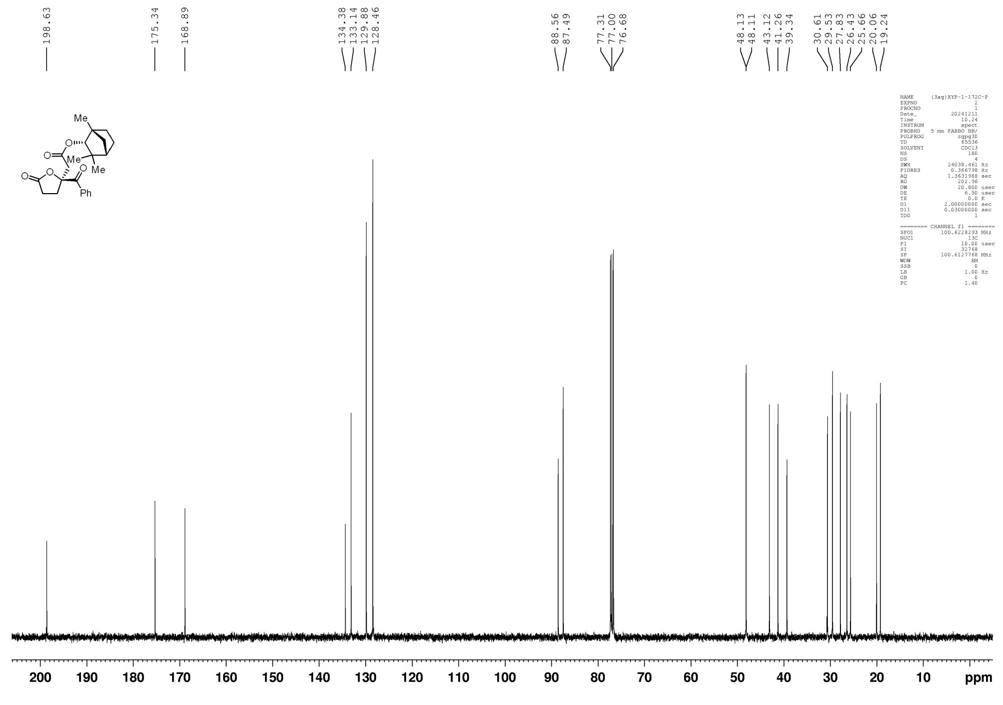

(1R,2S,5R)-2-isopropyl-5-methylcyclohexyl 2-((S)-2-benzoyl-5-oxotetrahydrofuran-2-yl)acetate (3at)

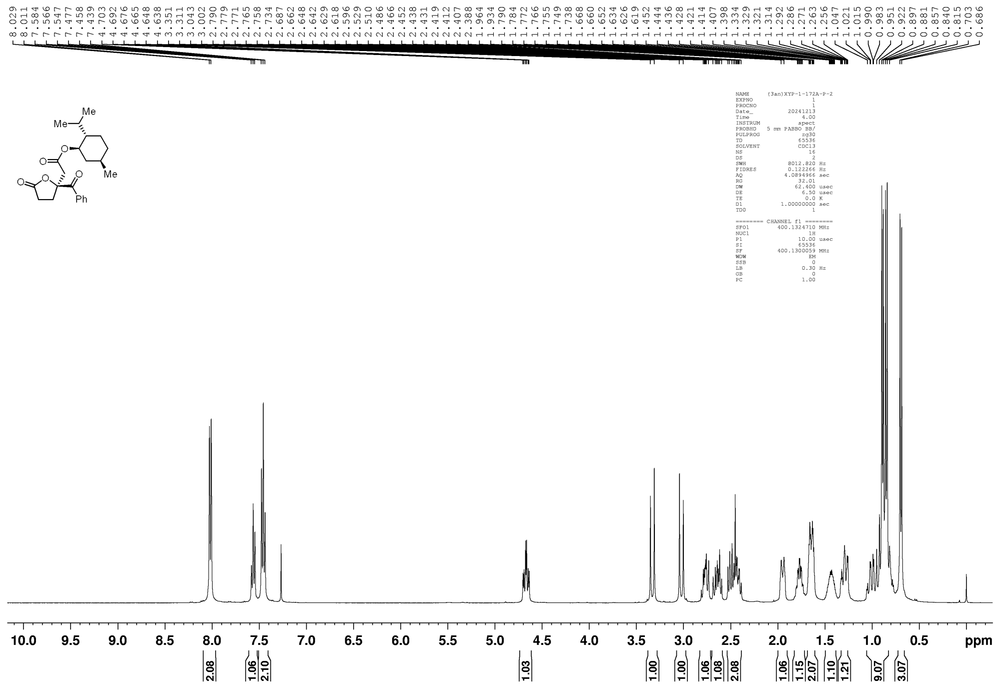

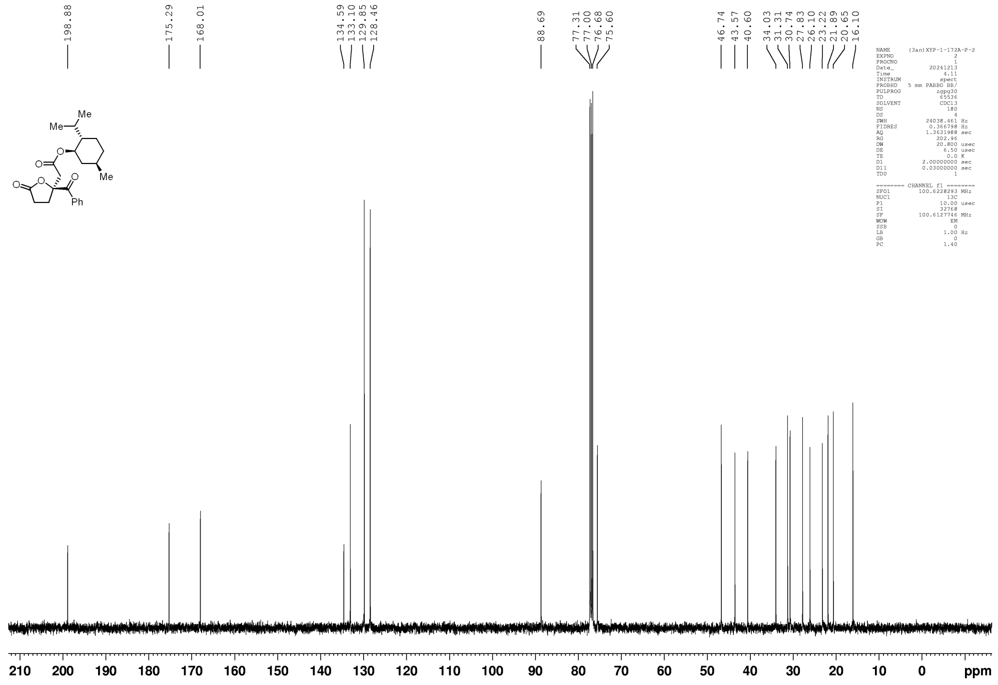

(1S,2R,5S)-2-isopropyl-5-methylcyclohexyl 2-((S)-2-benzoyl-5-oxotetrahydrofuran-2-yl)acetate (3au)

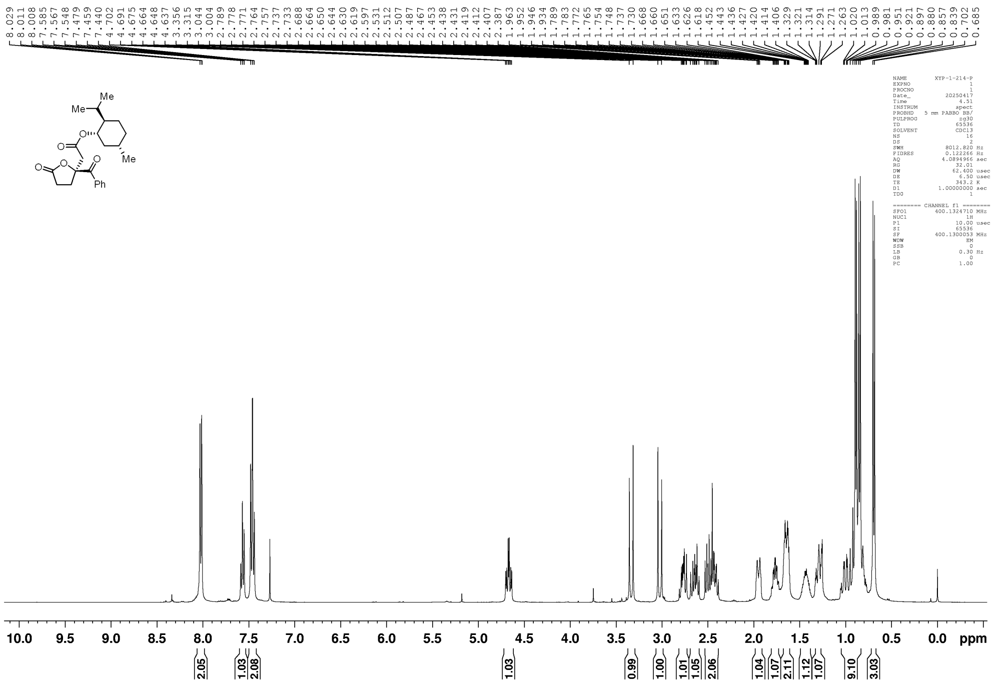

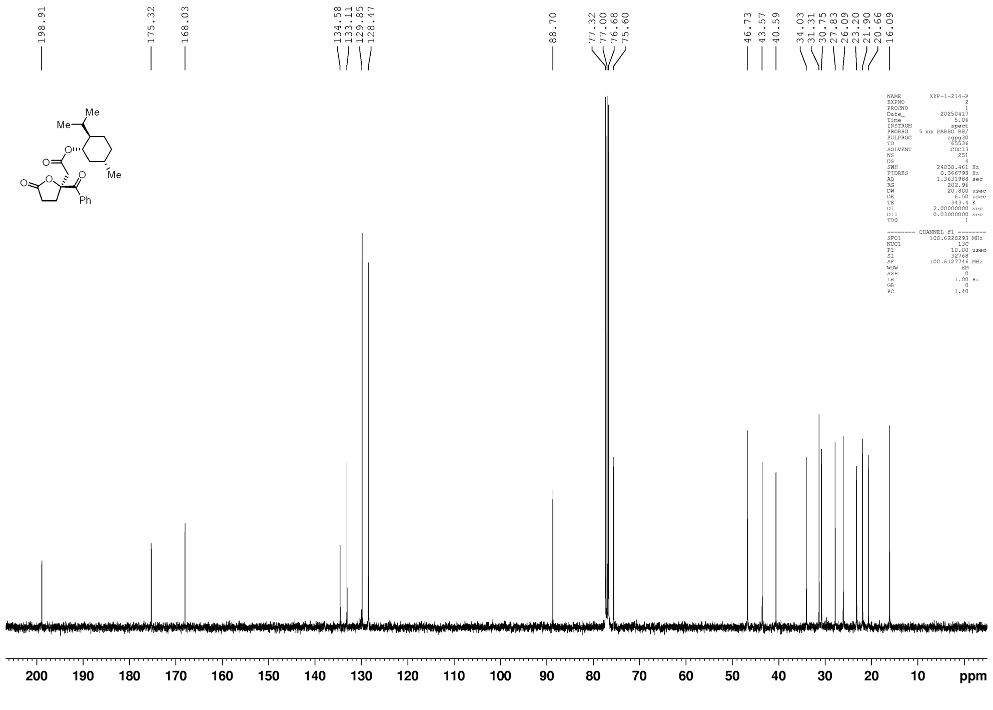

((3aR,5R,5aS,8aS,8bR)-2,2,7,7-tetramethyltetrahydro-5H-bis([1,3]dioxolo)[4,5-b:4',5'-d]pyran-5-yl)methyl 2-((S)-2-benzoyl-5-oxotetrahydrofuran-2-yl)acetate (3av)

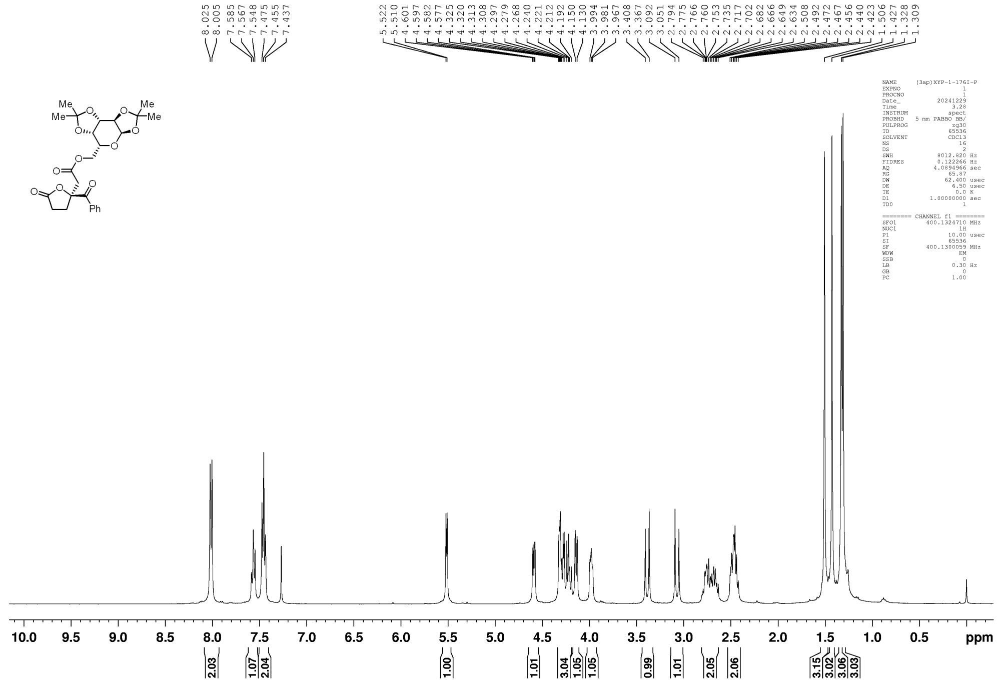

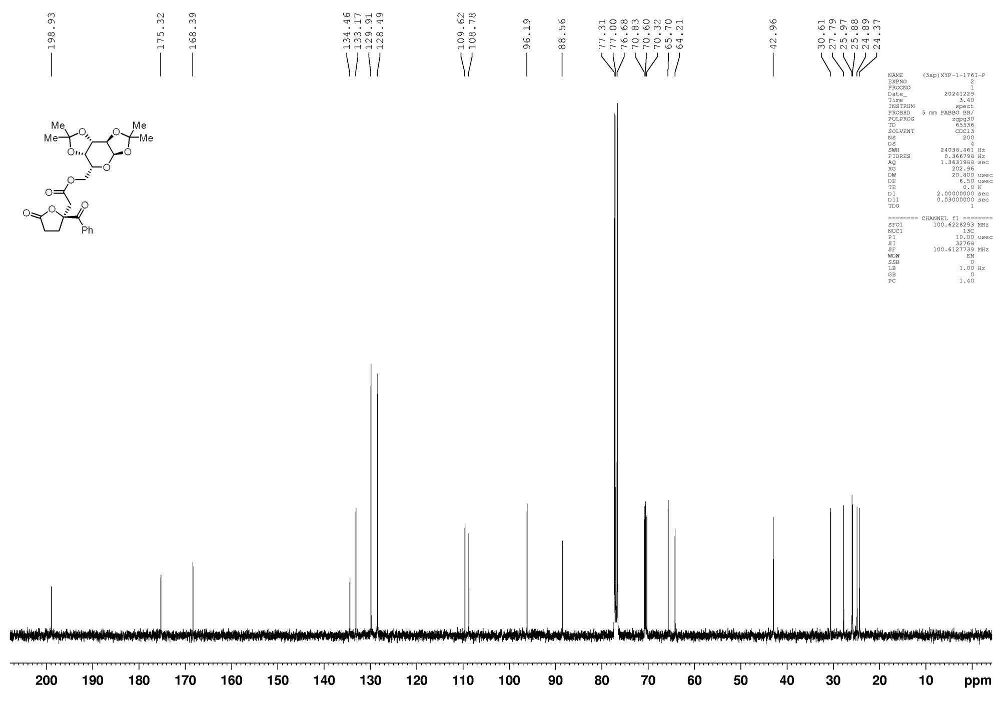

((3aS,5aR,8aR,8bS)-2,2,7,7-tetramethyltetrahydro-3aH-bis([1,3]dioxolo)[4,5-b:4',5'-d]pyran-3a-yl)methyl 2-((S)-2-benzoyl-5-oxotetrahydrofuran-2-yl)acetate (3aw)

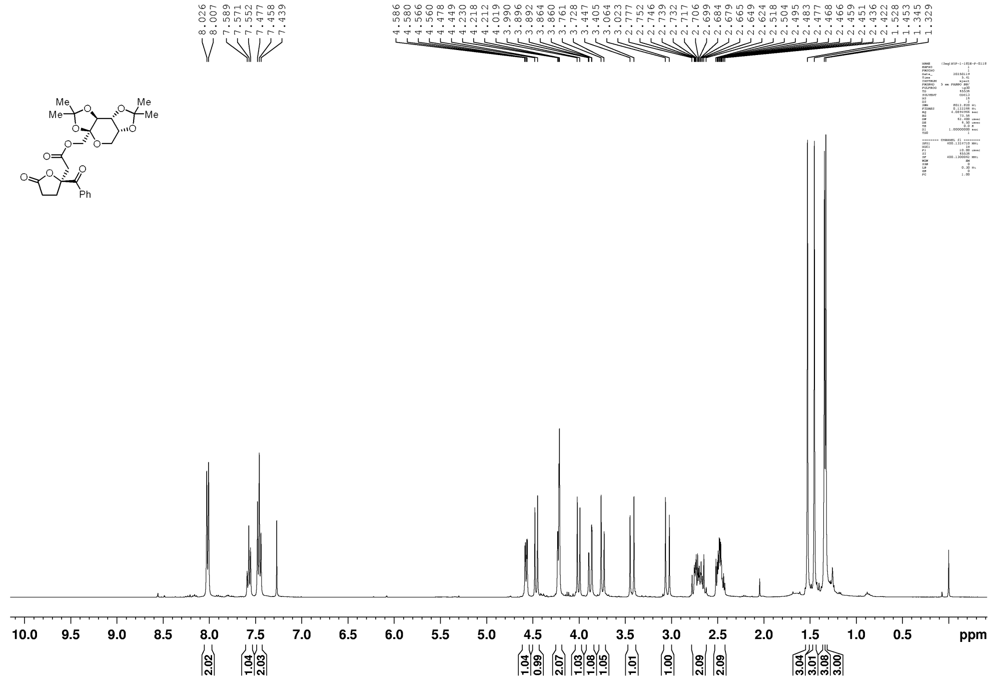

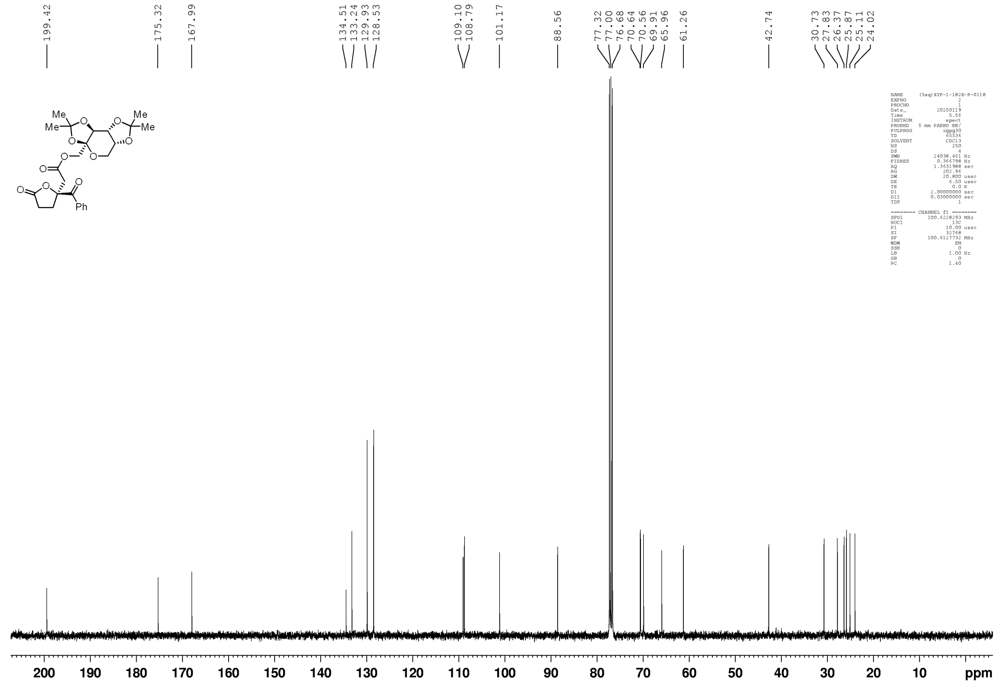

((3aR,4R,6R,6aR)-6-methoxy-2,2-dimethyltetrahydrofuro[3,4-d][1,3]dioxol-4-yl)methyl 2-((S)-2-benzoyl-5-oxotetrahydrofuran-2-yl)acetate (3ax)

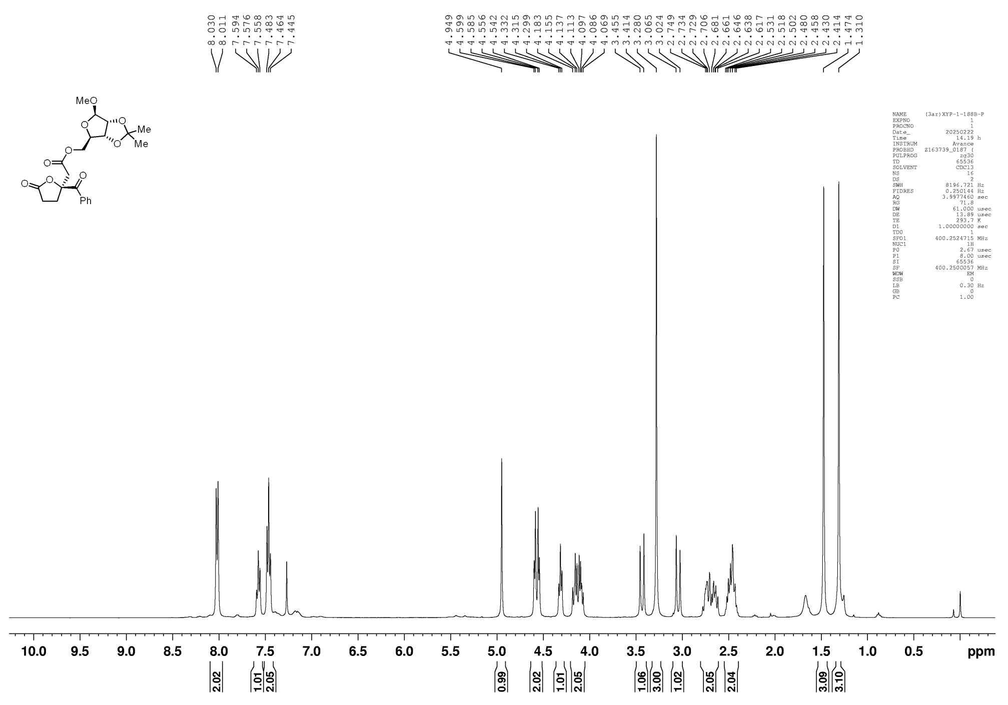

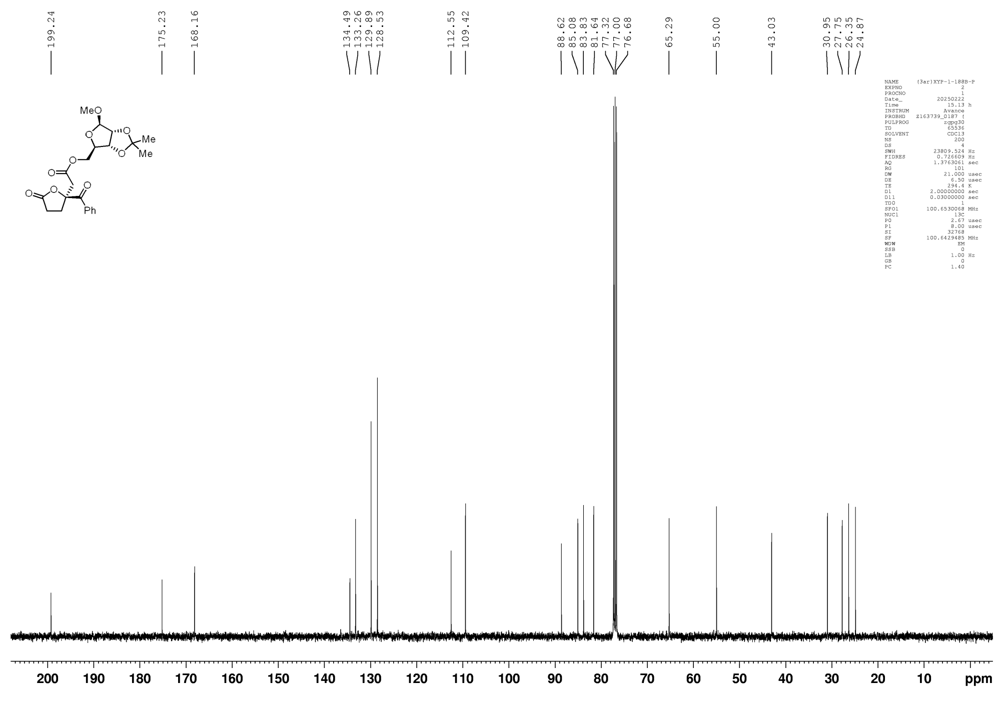

(3S,8S,9S,10R,13R,14S,17R)-10,13-dimethyl-17-((R)-6-methylheptan-2-yl)-2,3,4,7,8,9,10,11,12,13,14,15,16,17-tetradecahydro-1H-cyclopenta[a]phenanthren-3-yl 2-((S)-2-benzoyl-5-oxotetrahydrofuran-2-yl)acetate (3ay)

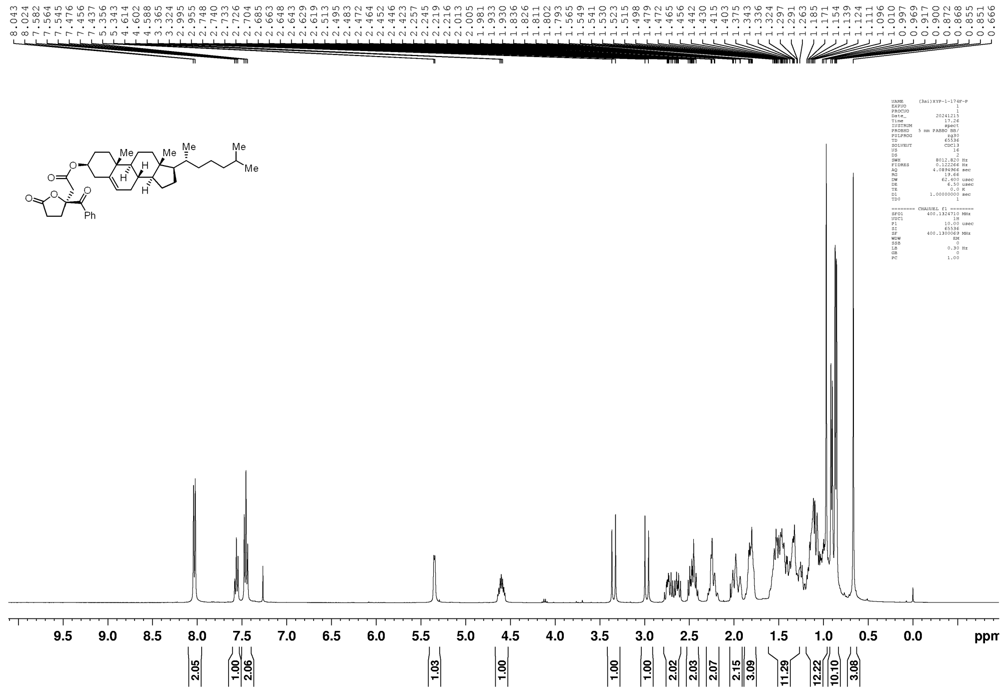

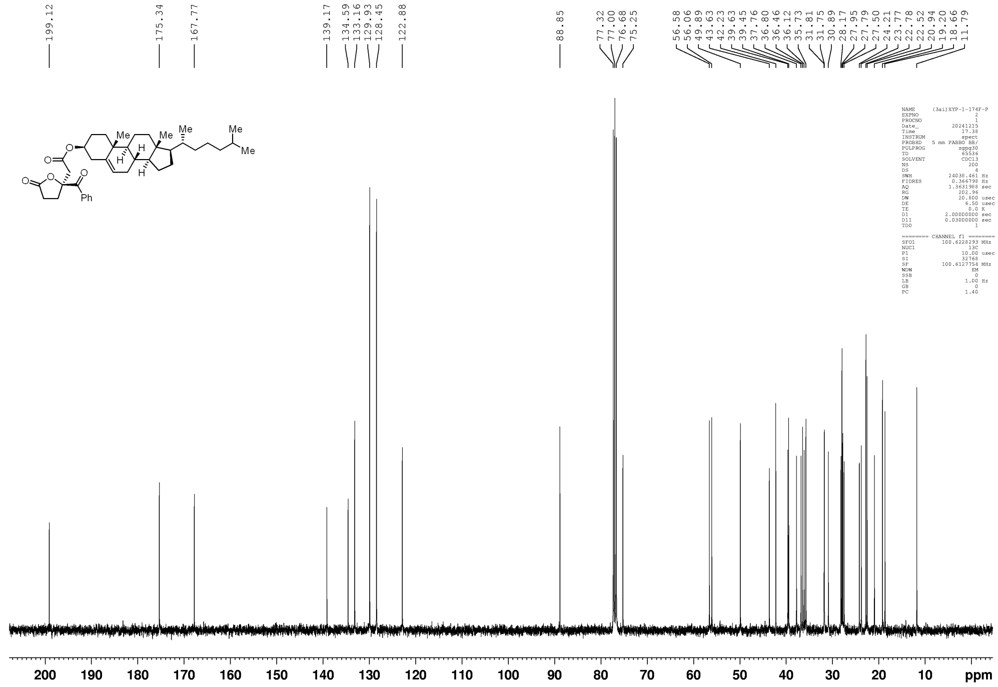

(3S,5S,8R,9S,10S,13R,14S,17R)-10,13-dimethyl-17-((R)-6-methylheptan-2-yl)hexadecahydro-1H-cyclopenta[a]phenanthren-3-yl 2-((S)-2-benzoyl-5-oxotetrahydrofuran-2-yl)acetate (3az)

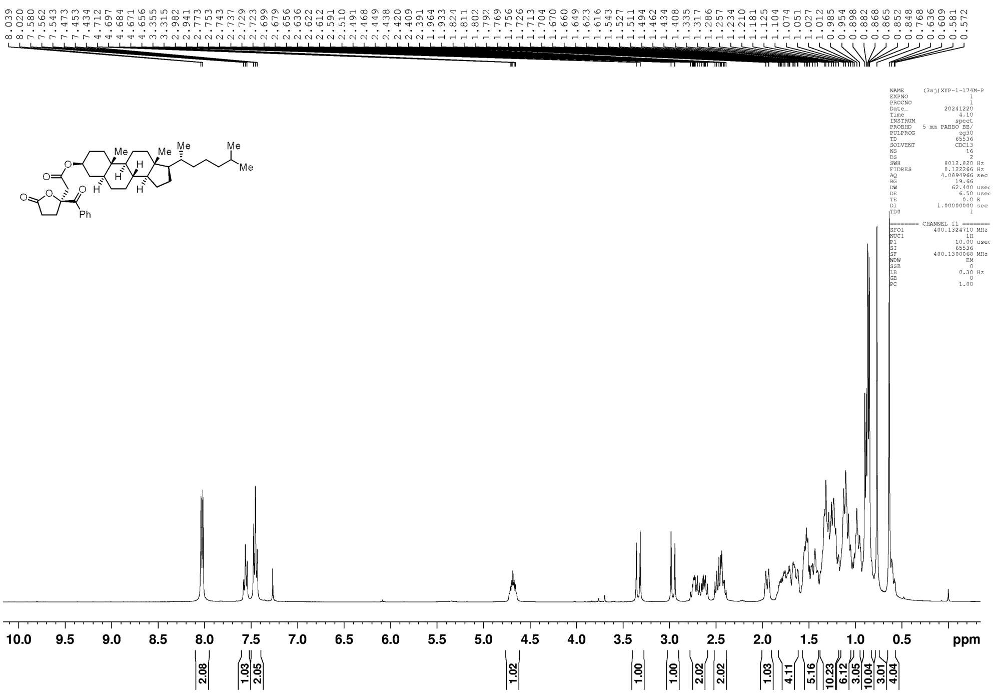

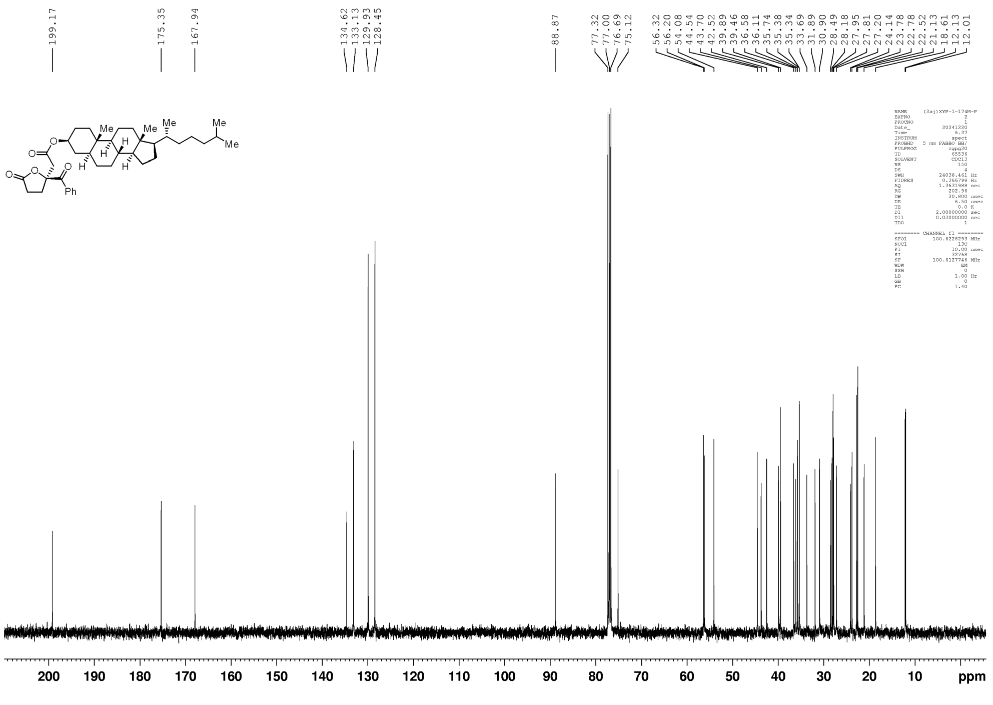

(4S,5'R,6aR,6bS,8aS,8bR,9S,10R,11aS,12aS,12bS)-5',6a,8a,9-tetramethyl-1,3,3',4,4',5,5',6,6a,6b,6',7,8,8a,8b,9,11a,12,12a,12b-icosahydrospiro[naphtho[2',1':4,5]indeno[2,1-b]furan-10,2'-pyran]-4-yl 2-((S)-2-benzoyl-5-oxotetrahydrofuran-2-yl)acetate (3ba)

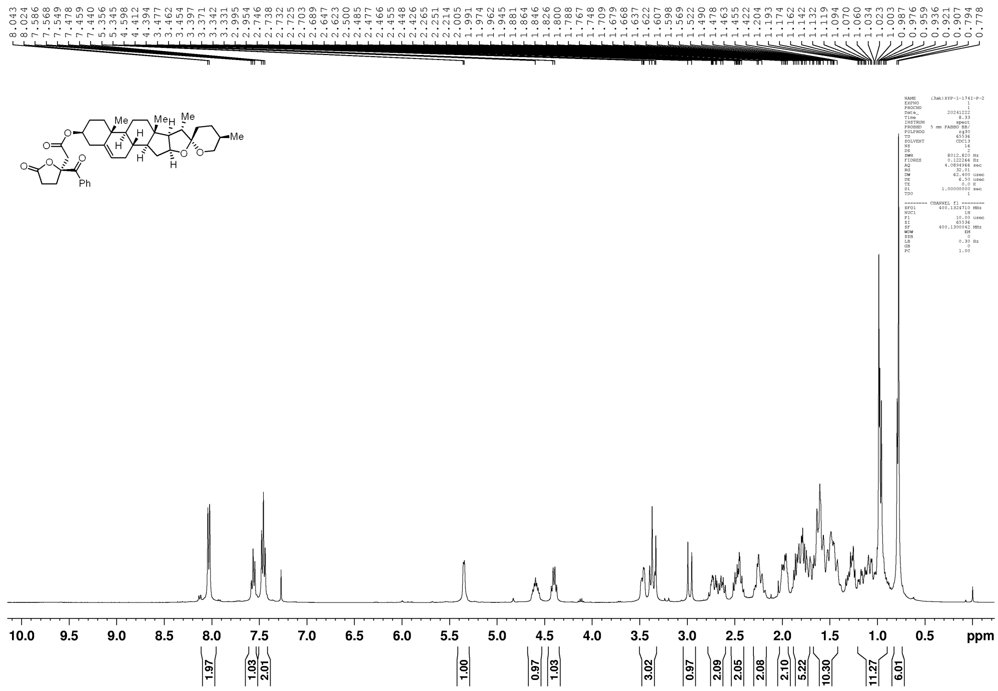

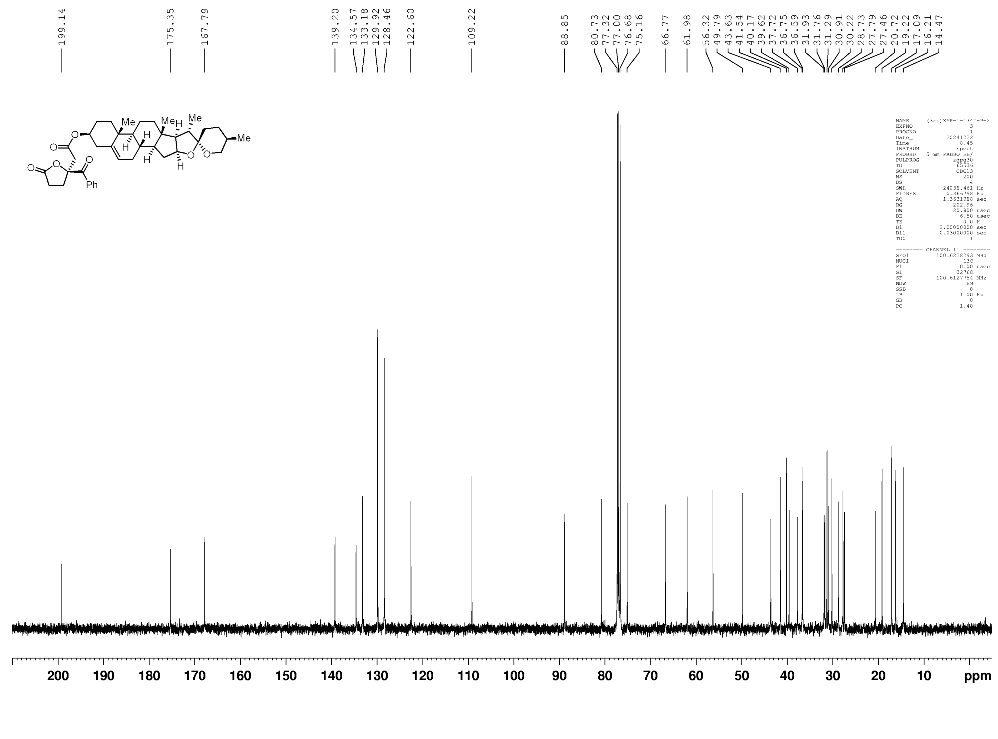

(3S,8S,9S,10R,13S,14S,17S)-17-acetyl-10,13-dimethyl-2,3,4,7,8,9,10,11,12,13,14,15,16,17-tetradecahydro-1H-cyclopenta[a]phenanthren-3-yl 2-((S)-2-benzoyl-5-oxotetrahydrofuran-2-yl)acetate (3bb)

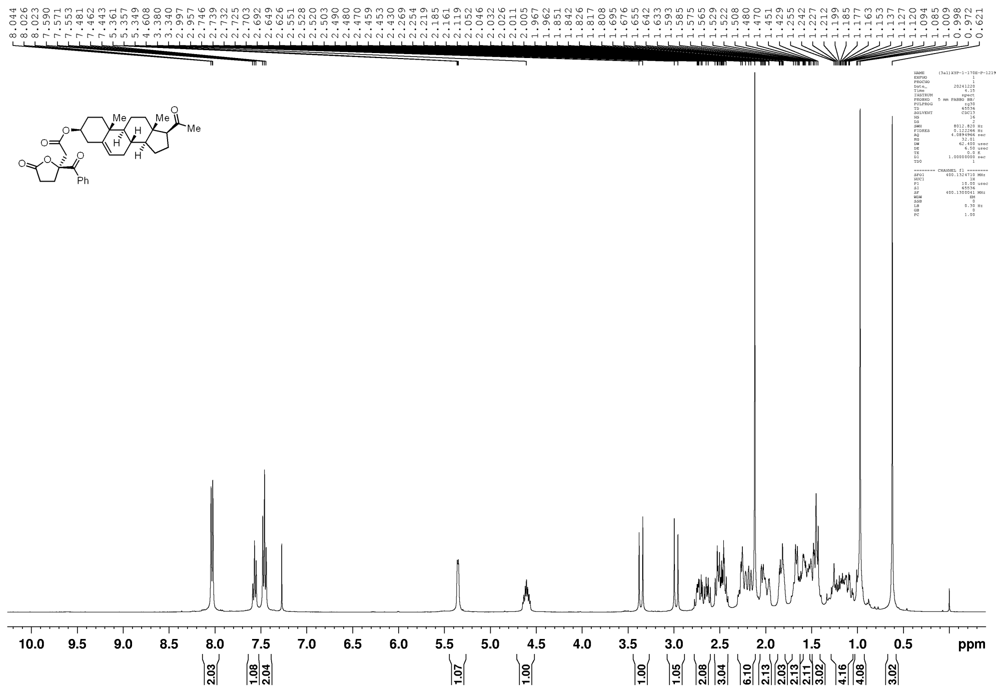

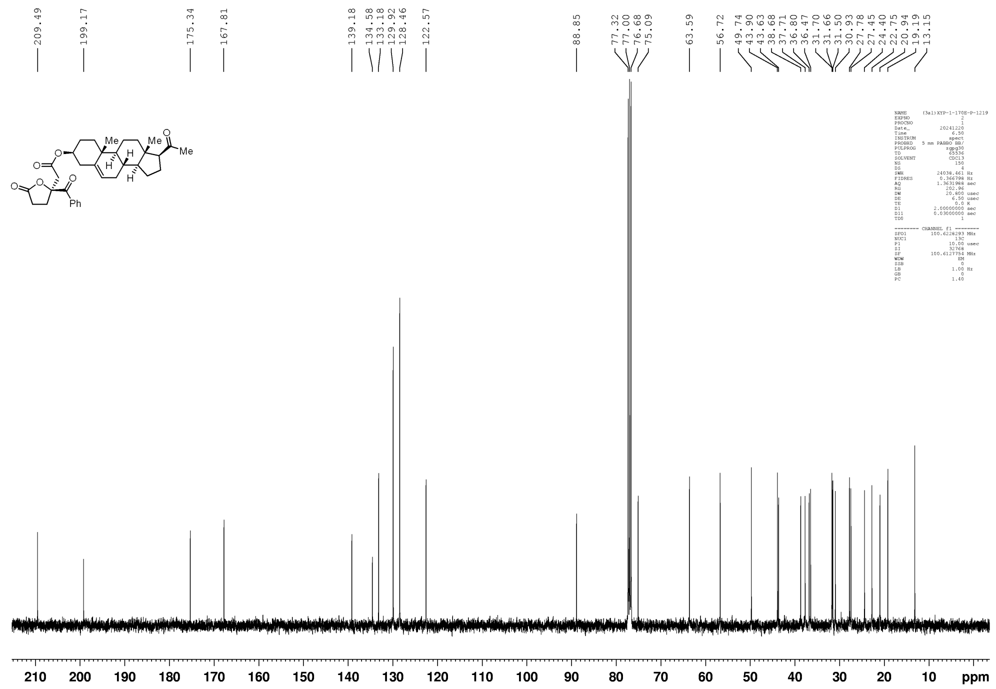

(3S,5S,8R,9S,10S,13S,14S)-10,13-dimethyl-17-oxohexadecahydro-1H-cyclopenta[a]phenanthren-3-yl 2-((S)-2-benzoyl-5-oxotetrahydrofuran-2-yl)acetate (3bc)

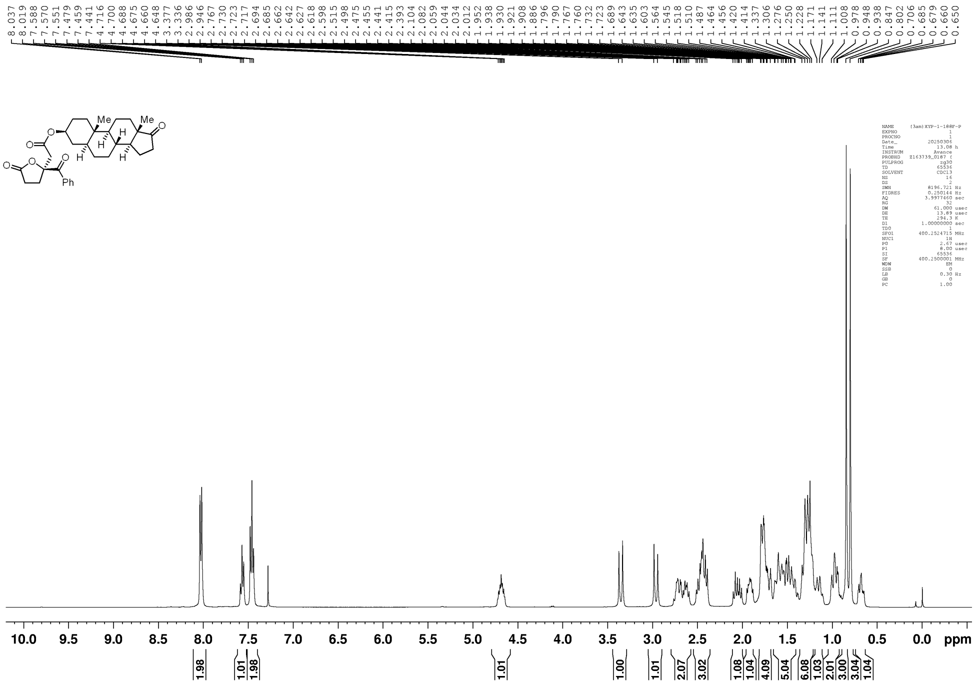

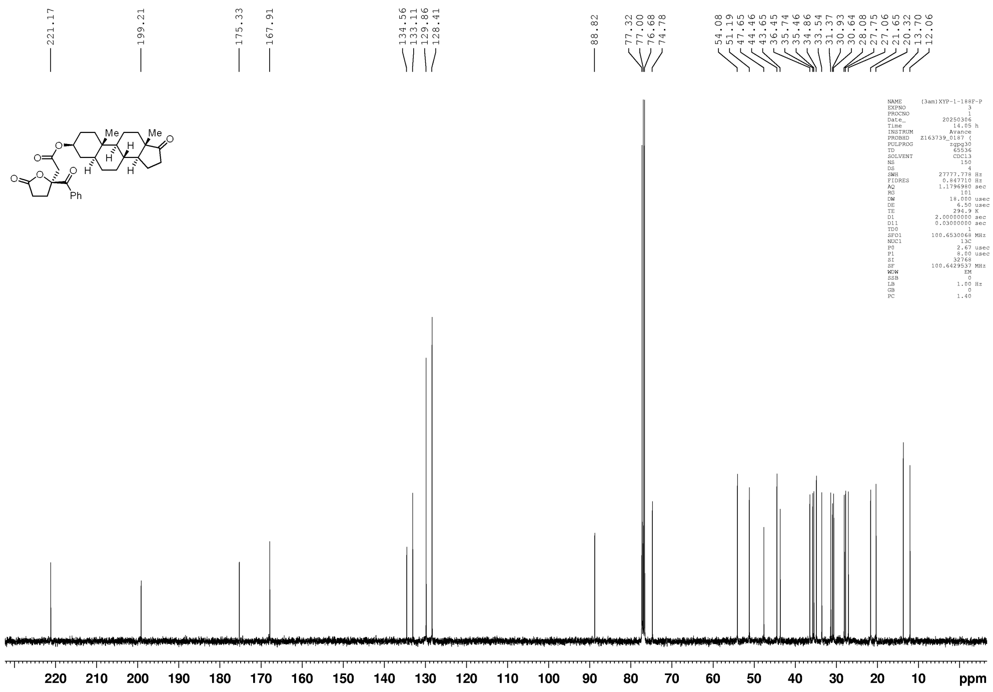

(1S,2R,5S)-2-isopropyl-5-methylcyclohexyl 2-((S)-2-benzoyl-5-oxotetrahydrofuran-2-yl)acetate and (1R,2S,5R)-2-isopropyl-5-methylcyclohexyl 2-((S)-2-benzoyl-5-oxotetrahydrofuran-2-yl)acetate (3at and 3au)

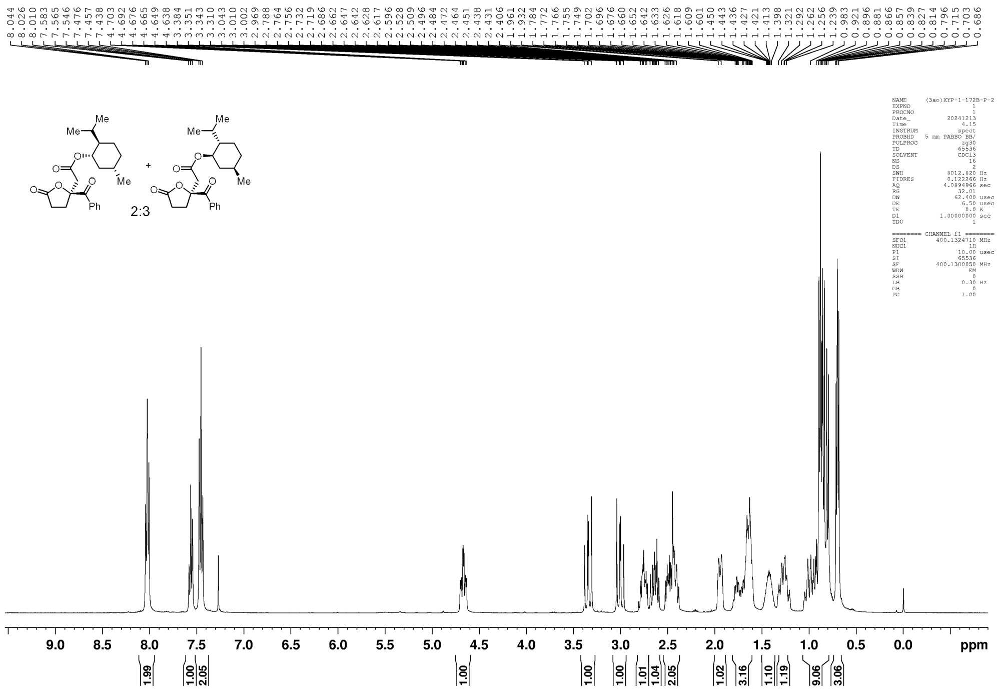

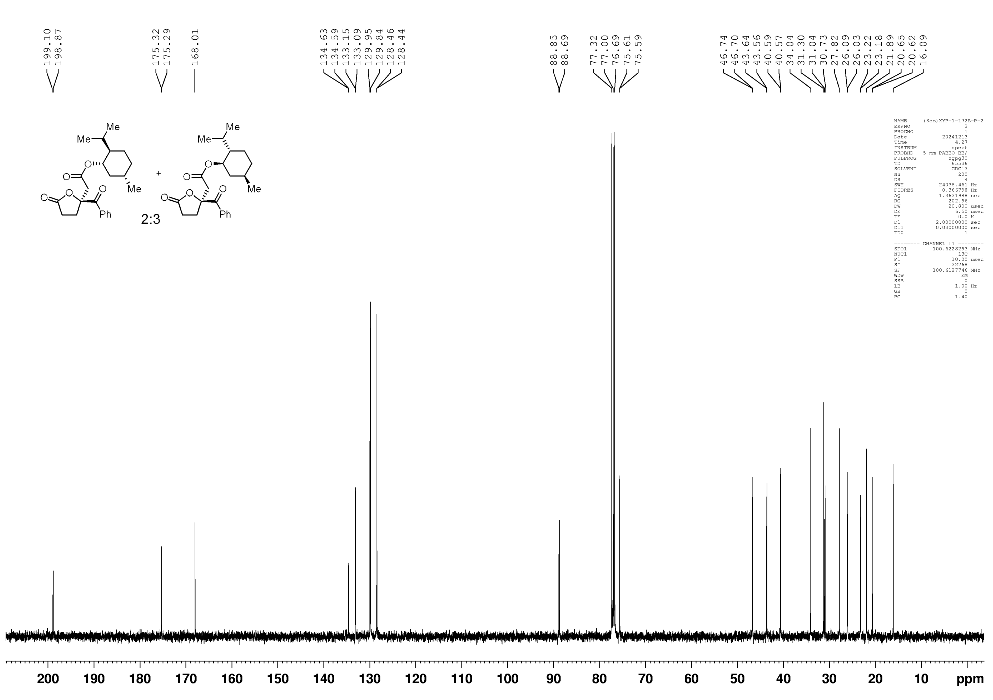

(4S)-6-(((3R)-adamantan-1-yl)methoxy)-4-benzoyl-4-hydroxy-6-oxohexanoic acid (3a-I)

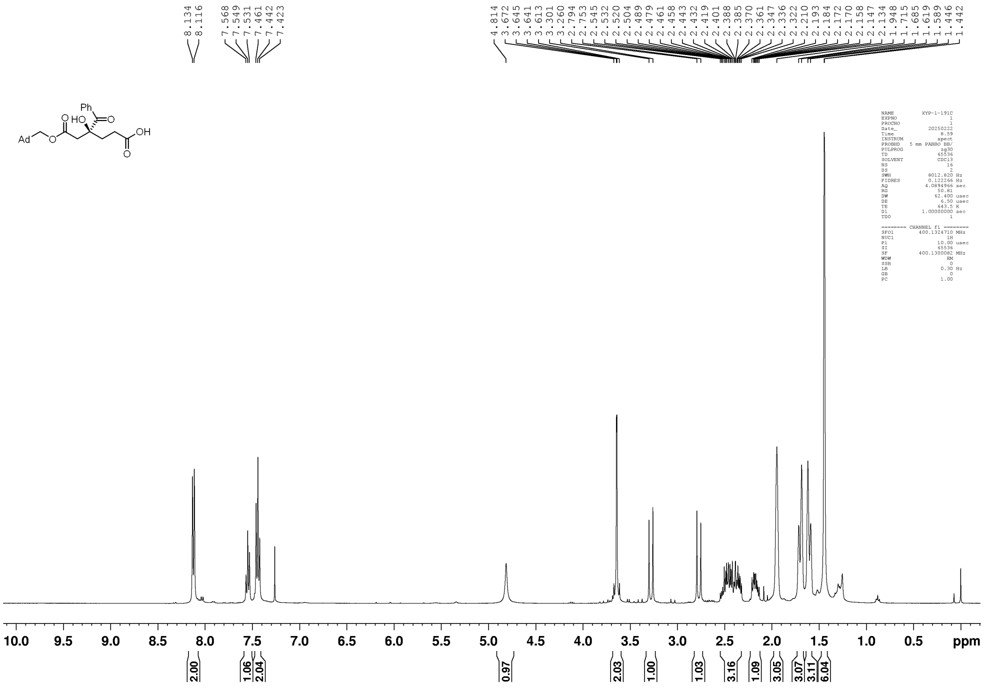

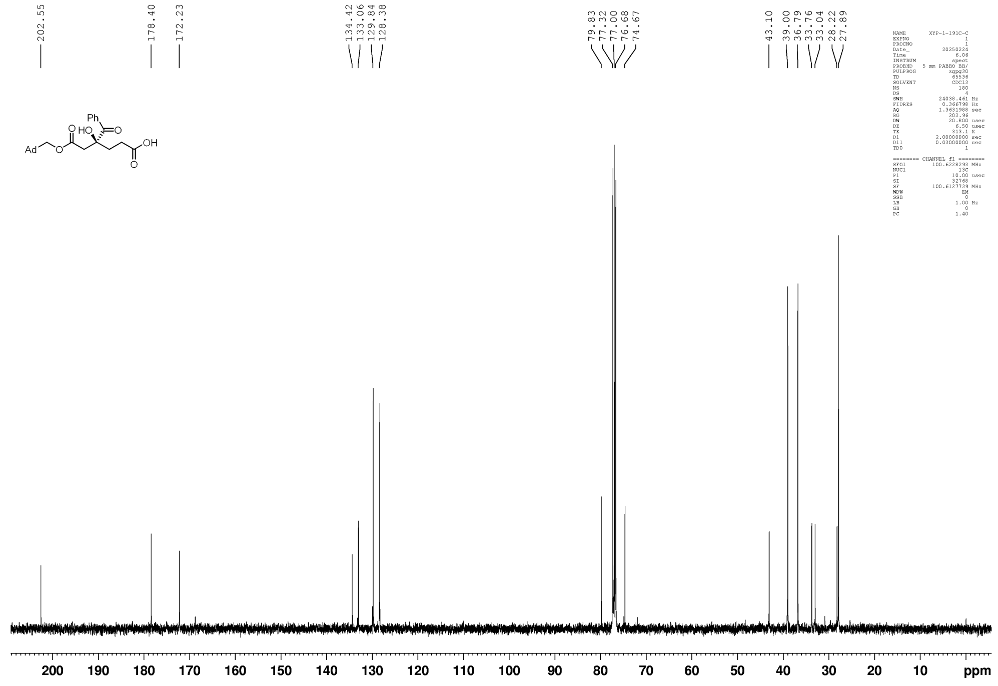

((3R)-adamantan-1-yl)methyl (3S)-3-benzoyl-3-hydroxy-6-oxo-6-(piperidin-1-yl)hexanoate (3a-II)

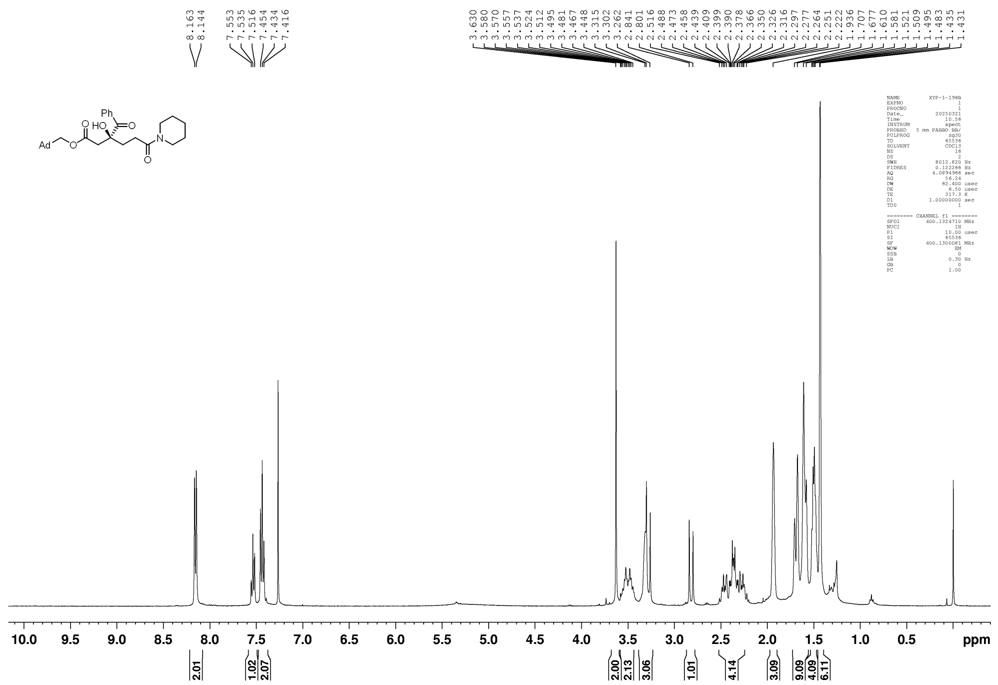

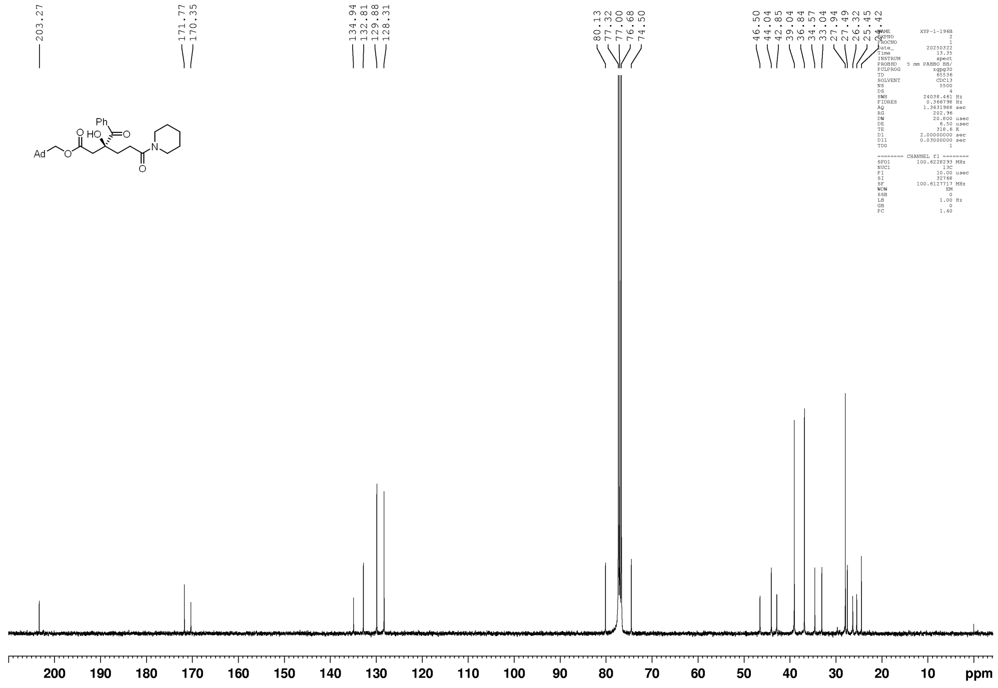

(2R)-2-(2-(((3S)-adamantan-1-yl)methoxy)-2-oxoethyl)-5-oxotetrahydrofuran-2-yl benzoate (3a-III)

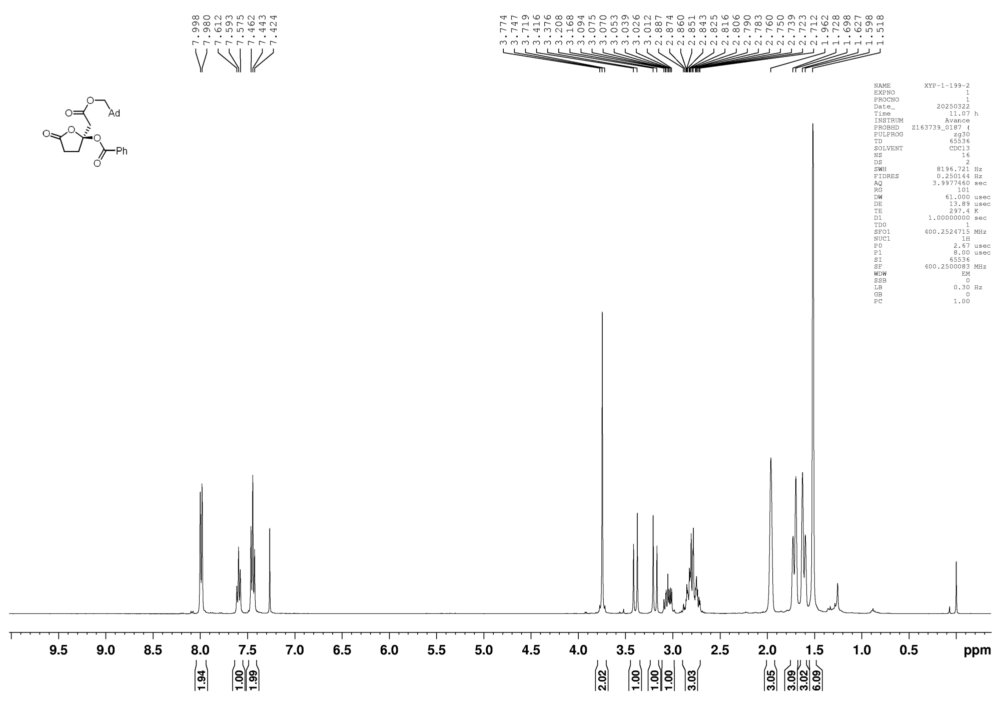

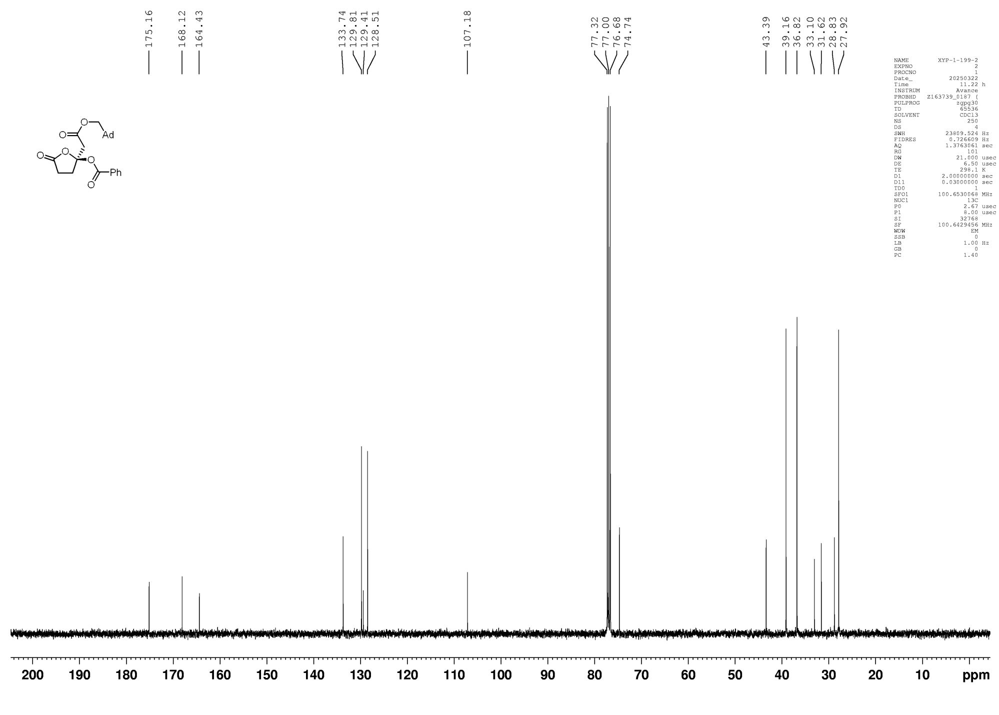

((3R)-adamantan-1-yl)methyl 2-((S)-2-((R)-hydroxy(phenyl)methyl)-5-oxotetrahydrofuran-2-yl)acetate (3a-IV)

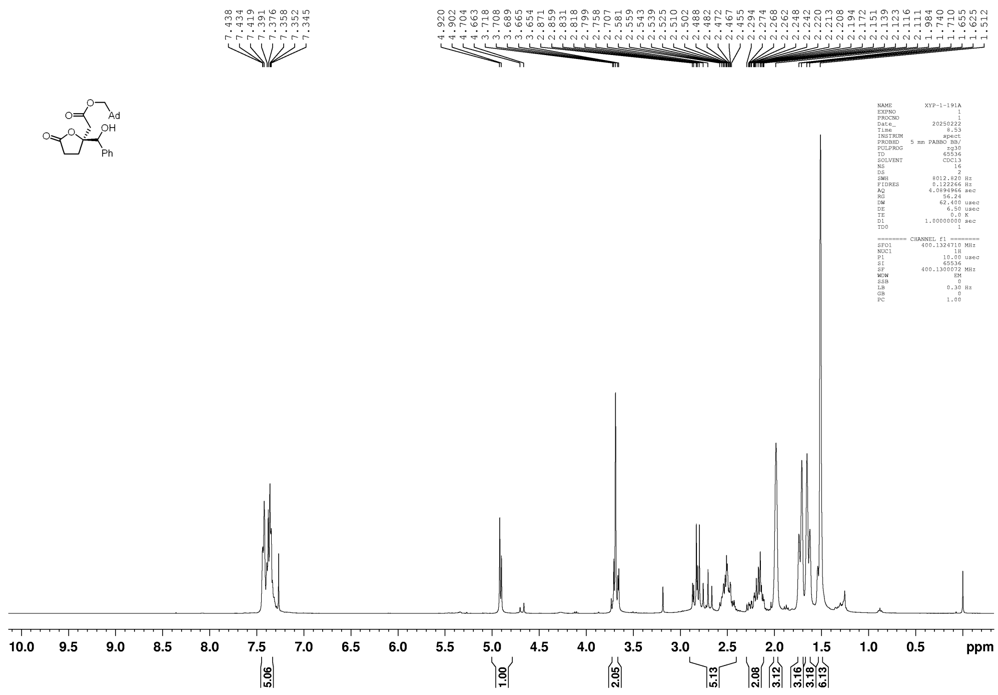

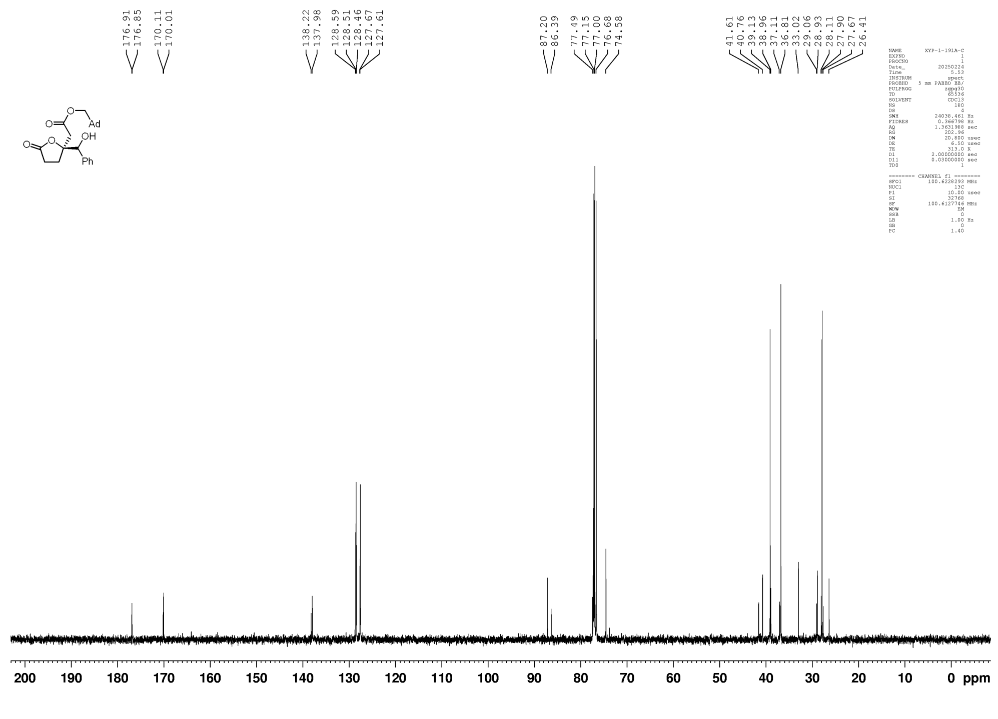

((3R)-adamantan-1-yl)methyl (3S)-3-benzoyl-6-(benzylamino)-3-hydroxy-6-oxohexanoate (3a-V)

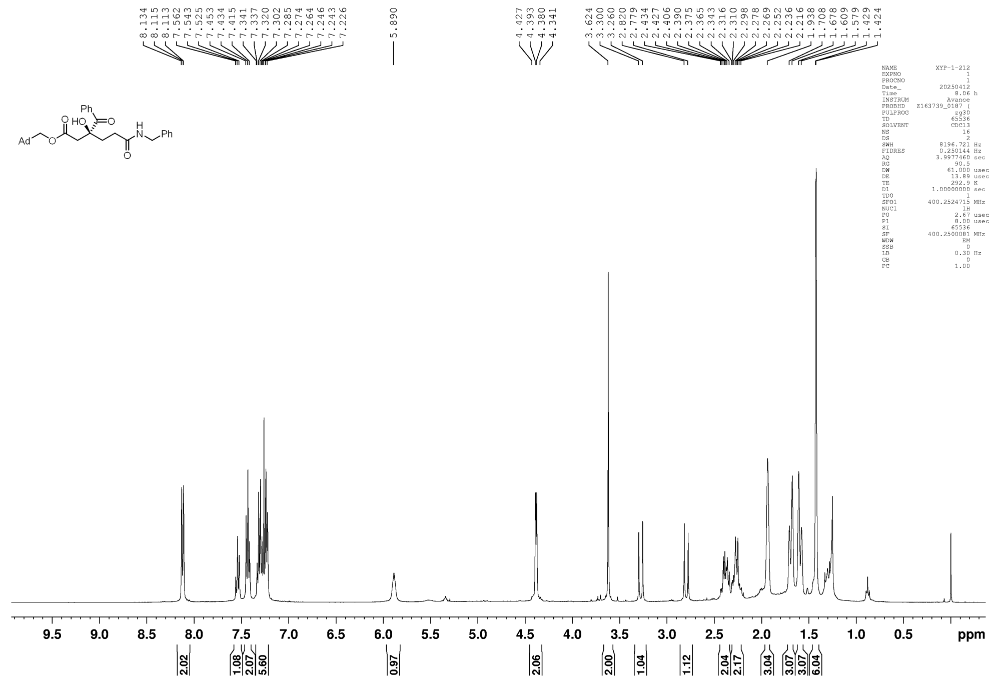

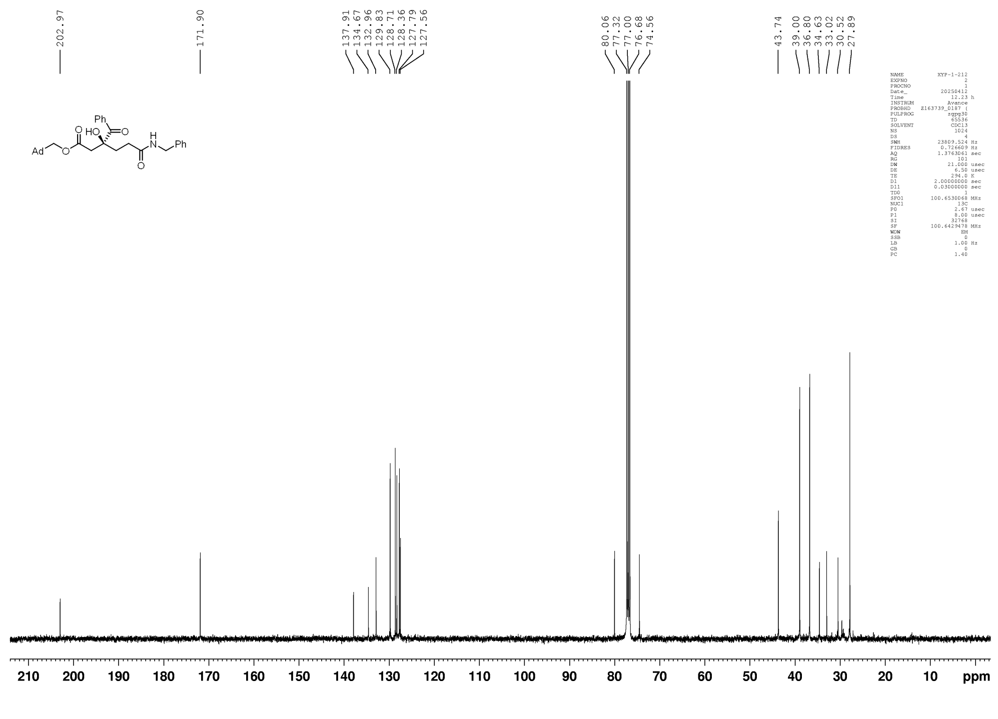

((3r,5r,7r)-adamantan-1-yl)methyl (2,2,6,6-tetramethylpiperidin-1-yl) carbonate (4)

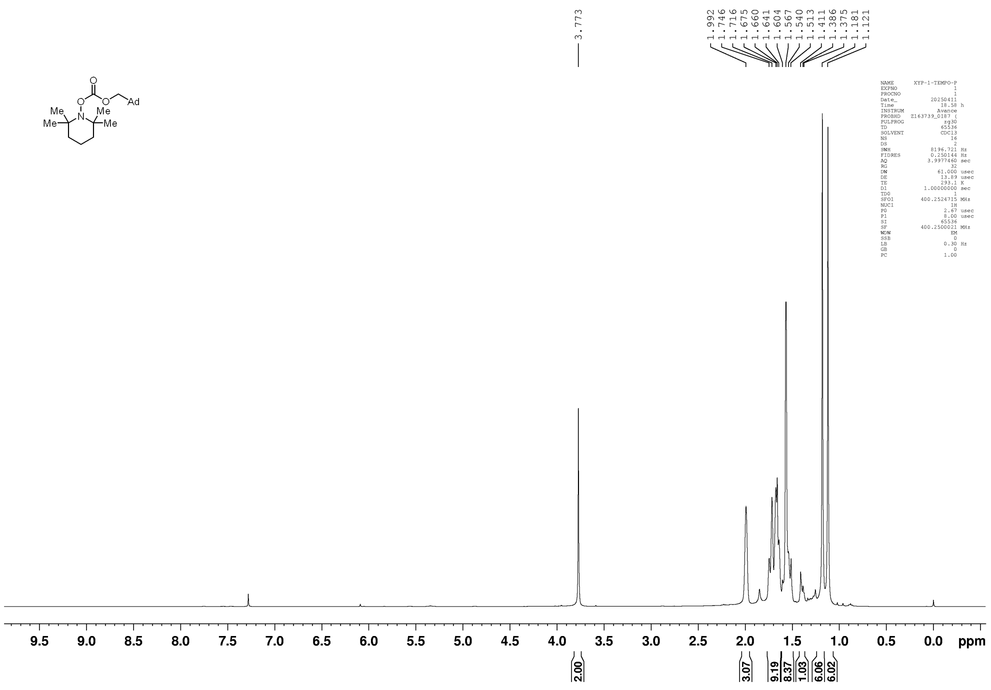

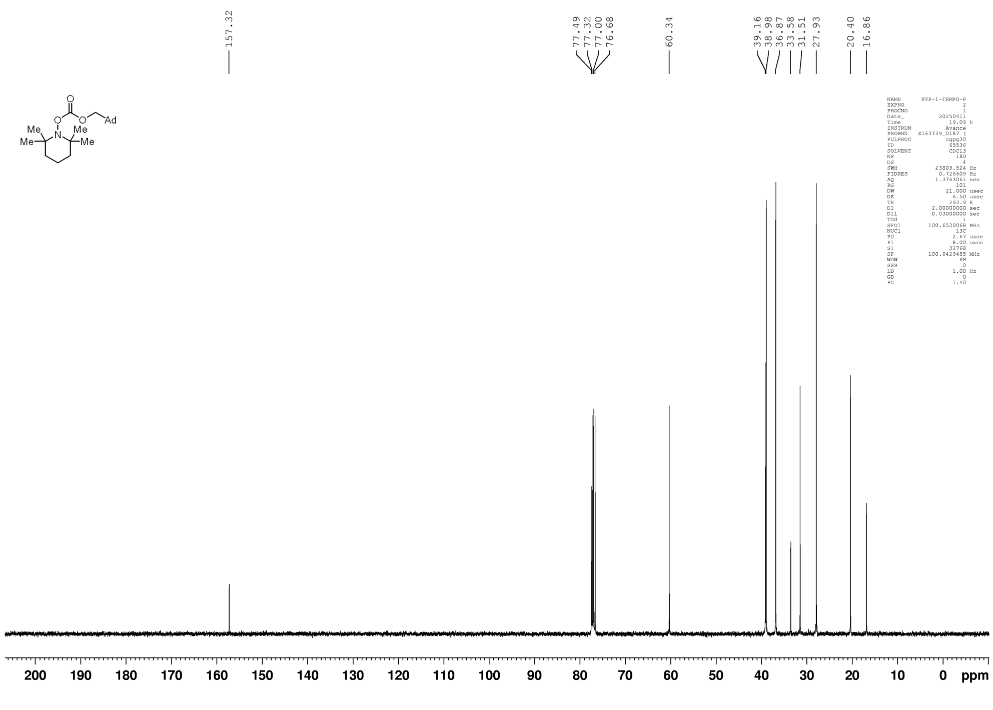

11. Supplementary references

1. (a) Hemric, B. N., Shen, K. & Wang, Q. Copper-catalyzed amino lactonization and amino oxygenation of alkenes using O-benzoylhydroxylamines. *J. Am. Chem. Soc.* **138**, 5813–5816 (2016). (b) Jiang, X., Liu, S., Yang, S., Jing, M., Xu, L., Yu, P., Wang, Y. & Yeung, Y.-Y. Enantioselective bromolactonization of deactivated olefinic acids. *Org. Lett.* **20**, 3259–3262 (2018). (c) Yang, X., Gao, H., Yan, J., Zhou, J. & Shi, L. Intramolecular chaperone-assisted dual-anchoring activation (ICDA): a suitable preorganization for electrophilic halocyclization. *Chem. Sci.* **15**, 6130–6140 (2024). (d) Lussier, T., Legault, C. Y. Iodine(III)-Mediated Enantioselective Oxidative Contraction of Dihydropyranones. *Org. Lett.* **25**, 2825–2829 (2023).
2. (a) Li, Y., Zhang, J., Li, D. & Chen, Y. Metal-Free C(sp^3)–H Allylation via Aryl Carboxyl Radicals Enabled by Donor–Acceptor Complex. *Org. Lett.* **20**, 3296–3299 (2018). (b) Slutskyy Y. & Overman, L. E. Generation of the methoxycarbonyl radical by visible-light photoredox catalysis and its conjugate addition with electron-deficient olefins. *Org. Lett.* **18**, 2564–2567 (2016). (c) Lackner, G. L., Quasdorf, K. W. & Overman, L. E. Direct construction of quaternary carbons from tertiary alcoholsvia photoredox-catalyzed fragmentation of tert-alkyl *N*-phthalimidoyl oxalates. *J. Am. Chem. Soc.* **135**, 15342−15345 (2013).
3. (a) Jin, Y., Fan, L.-F., Wang, E., Yu, L., Hirao, H. & Gong. L.-Z. Atom transfer radical coupling enables highly enantioselective carbo-oxygenation of alkenes with hydrocarbons. *J. Am. Chem. Soc.* **145**, 22031−22040 (2023). (b) Maji, U., Baidya, A., Das, S. & Guin, J. Bifunctional NHC-catalyzed asymmetric intramolecular conjugate addition via noncovalent interaction. *Org. Lett.* **27**, 2423–2428 (2025).
4. Gaussian 16, R. C. e., M. J. Frisch, G. W. Trucks, H. B. Schlegel, G. E. Scuseria, M. A. Robb, J. R. Cheeseman, G. Scalmani, V. Barone, G. A. Petersson, H. Nakatsuji, X. Li, M. Caricato, A. V. Marenich, J. Bloino, B. G. Janesko, R. Gomperts, B. Mennucci, H. P. Hratchian, J. V. Ortiz, A. F. Izmaylov, J. L. Sonnenberg, D. Williams-Young, F. Ding, F. Lipparini, F. Egidi, J. Goings, B. Peng, A. Petrone, T. Henderson, D. Ranasinghe, V. G. Zakrzewski, J. Gao, N. Rega, G. Zheng, W. Liang, M. Hada, M. Ehara, K. Toyota, R. Fukuda, J. Hasegawa, M. Ishida, T. Nakajima, Y. Honda, O. Kitao, H. Nakai, T. Vreven, K. Throssell, J. A. Montgomery, Jr., J. E. Peralta, F. Ogliaro, M. J. Bearpark, J. J. Heyd, E. N. Brothers, K. N. Kudin, V. N. Staroverov, T. A. Keith, R. Kobayashi, J. Normand, K.

Raghavachari, A. P. Rendell, J. C. Burant, S. S. Iyengar, J. Tomasi, M. Cossi, J. M. Millam, M. Klene, C. Adamo, R. Cammi, J. W. Ochterski, R. L. Martin, K. Morokuma, O. Farkas, J. B. Foresman, & D. J. Fox, Gaussian,Inc., Wallingford CT, 2016.

5. Harvey, J. N., Aschi, M., Schwarz, H. & Koch, W. The singlet and triplet states of phenyl cation. A hybrid approach for locating minimum energy crossing points between non-interacting potential energy surfaces. *Theor. Chem. Acc.* **99**, 95−99 (1998).
6. Grimme, S., Antony, J., Ehrlich, S. & Krieg, H. A consistent and accurate ab initio parametrization of density functional dispersion correction (DFT-D) for the 94 elements H-Pu. *J. Chem. Phys*. **132**, 154104 (2010).
7. Grimme, S., Ehrlich, S. & Goerigk, L. Effect of the damping function in dispersion corrected density functional theory. *J. Comput. Chem.* **32**, 1456−65 (2011).
8. Weigend, F. & Ahlrichs, R. Balanced basis sets of split valence, triple zeta valence and quadruple zeta valence quality for H to Rn: Design and assessment of accuracy. *Phys. Chem. Chem. Phys*. **7**, 3297−305 (2005).
9. Fukui, K. The path of chemical reactions-the IRC approach. *Acc. Chem. Res*. **14**, 363−368 (1981).
10. Weigend, F. Accurate Coulomb-fitting basis sets for H to Rn. *Phys. Chem. Chem. Phys*. **8**, 1057−1065 (2006).
11. Marenich, A. V., Cramer, C. J. & Truhlar, D. G. Universal Solvation Model Based on Solute Electron Density and on a Continuum Model of the Solvent Defined by the Bulk Dielectric Constant and Atomic Surface Tensions. *J. Phys. Chem*. *B* **113**, 6378−6396 (2009).
